# Supplementary material for: COVID-19 and common mental health symptoms in the early phase of the pandemic: An umbrella review of the evidence
Source: PLoS Med. 2023 Apr 25;20(4):e1004206. doi: 10.1371/journal.pmed.1004206 (PMC10129001; doi:10.1371/journal.pmed.1004206)
Supplement: S1 Text — Table A. Search strategies used to retrieve papers from different databases. Table B. List of excluded meta-analyses by full-text screening with exclusion reason.Table C. AMSTAR 2 ratings. (DOCX) [file pmed.1004206.s002.docx]

Contents

[**Table A***.* Search Strategies Used to Retrieve Papers from Different Databases 2](#_Toc128946052)

[**Table B.** List of excluded meta-analyses by full text screening with exclusion reason. 5](#_Toc128946053)

[**Table C**. Appendix AMSTAR 2 ratings 31](#_Toc128946054)

[References 38](#_Toc128946055)

### **Table A***.* Search Strategies Used to Retrieve Papers from Different Databases

| **Database** | **Date of search** | **No. of papers** | **Search terms used** |
| --- | --- | --- | --- |
| EMBASE (OVID) | October 6, 2021  August 12, 2022 | 10.242  9.160 | (('coronavirus'/exp OR 'corona virus'/exp OR 'coronavirus infect*' OR 'corona virus infect*' OR 'betacoronavirus*' OR 'beta coronavirus*' OR 'beta corona virus*') AND  or wuhan or hubei or AND 'severe acute respiratory syndrome coronavirus 2'/exp OR 'sars cov-2'/exp OR 'sarscov 2' OR 'sarscov2' OR 'cov2' OR 'sars 2' OR 'covid'/exp OR 'coronavirus 2' OR 'covid19'/exp OR 'covid-19'/exp OR 'covid 19 or ncov' OR 'new corona virus' OR 'new coronavirus' OR 'novel coronavirus' OR 'novel corona virus or ncp or corona' OR 'pandemic'/exp OR 'global pandemic' OR 'epidemic'/exp OR 'global epidemic or coronavirinae') AND  ('mental or psycholog*' OR 'distress'/exp OR psychiatr* OR 'social stigma'/exp OR 'stigma'/exp OR 'fear'/exp OR 'phobia'/exp OR 'anxiety or anxi*' OR 'stress'/exp OR 'worry'/exp OR 'physiological stress'/exp OR angst OR depress* OR 'mood or irritab*' OR sad* OR affect* OR 'fatigue'/exp OR hope* OR neurotic* OR 'grief or griev* or bereav*' OR 'loss'/exp OR 'burnout'/exp OR 'burned out' OR 'burnt out' OR 'trauma'/exp OR trauma* OR 'post-traumatic' OR 'post-trauma' OR 'ptsd'/exp OR 'post traumatic stress disorder'/exp OR 'post-traumatic stress disorder'/exp OR 'posttraumatic stress disorder'/exp OR 'anger'/exp OR 'substance abuse'/exp OR 'substance use'/exp OR 'substance use disorder'/exp OR 'substance dependence'/exp OR 'drug use'/exp OR 'drug abuse'/exp OR 'drug dependence'/exp) AND  (review:ab,ti OR 'systematic review':ab,ti OR 'narrative review':ab,ti OR 'meta-analysis':ab,ti OR 'metaanalysis':ab,ti OR 'meta analysis':ab,ti OR 'literature review or synthes*':ab,ti) |
| MEDLINE (OVID) | October 6, 2021  August 12, 2022 | 21.876  12.204 | ((coronavirus OR "corona virus" OR "coronavirus infect*" OR "corona virus infect*" OR "betacoronavirus*" OR "beta coronavirus*" OR "beta corona virus*" OR wuhan OR hubei OR "severe acute respiratory syndrome coronavirus 2" OR "SARS CoV-2" OR "SARSCoV 2" OR "SARSCoV2" OR "cov2" or "sars 2" OR COVID OR "coronavirus 2" OR "covid19" OR "Covid-19" OR "Covid 19" OR nCov OR "new corona virus" OR "new coronavirus" OR "novel coronavirus" OR "novel corona virus" OR ncp OR corona OR pandemic OR "global pandemic" OR epidemic OR "global epidemic" OR coronavirinae) AND  (mental OR psycholog* OR "distress" OR "psychiatr*" OR "social stigma" OR stigma OR fear OR phobia OR anxiety OR anxi* OR stress OR worry OR "physiological stress" OR angst OR depress* OR mood OR irritab* OR sad* OR affect* OR fatigue OR hope* OR neurotic* OR grief OR griev* OR bereav* OR loss OR burnout OR "burned out" OR "burnt out" OR trauma OR trauma* OR post-traumatic OR post-trauma OR PTSD OR "Post traumatic stress disorder" OR "post-traumatic stress disorder" OR "posttraumatic stress disorder" OR anger OR "substance abuse" OR "substance use" OR "substance use disorder" OR "substance dependence" OR "drug use" OR "drug abuse" OR "drug dependence")) AND  (review[Title/Abstract] OR "systematic review"[Title/Abstract] OR "narrative review"[Title/Abstract] OR "meta-analysis"[Title/Abstract] OR "metaanalysis"[Title/Abstract] OR "meta analysis"[Title/Abstract] OR "literature review"[Title/Abstract] OR synthes*[Title/Abstract]) |
| PsycInfo | October 6, 2021  August 12, 2022 | 688  2.373 | TX ( coronavirus OR "corona virus" OR "coronavirus infect*" OR "corona virus infect*" OR "betacoronavirus*" OR "beta coronavirus*" OR "beta corona virus*" OR wuhan OR hubei OR "severe acute respiratory syndrome coronavirus 2" OR "SARS CoV-2" OR "SARSCoV 2" OR "SARSCoV2" OR "cov2" or "sars 2" OR COVID OR "coronavirus 2" OR "covid19" OR "Covid-19" OR "Covid 19" OR nCov OR "new corona virus" OR "new coronavirus" OR "novel coronavirus" OR "novel corona virus" OR ncp OR corona OR pandemic OR "global pandemic" OR epidemic OR "global epidemic" OR coronavirinae ) AND  TX ( mental OR psycholog* OR "distress" OR "psychiatr*" OR "social stigma" OR stigma OR fear OR phobia OR anxiety OR anxi* OR stress OR worry OR "physiological stress" OR angst OR depress* OR mood OR irritab* OR sad* OR affect* OR fatigue OR hope* OR neurotic* OR grief OR griev* OR bereav* OR loss OR burnout OR "burned out" OR "burnt out" OR trauma OR trauma* OR post-traumatic OR post-trauma OR PTSD OR "Post traumatic stress disorder" OR "post-traumatic stress disorder" OR "posttraumatic stress disorder" OR anger OR "substance abuse" OR "substance use" OR "substance use disorder" OR "substance dependence" OR "drug use" OR "drug abuse" OR "drug dependence" ) AND  AB ( review OR "systematic review" OR "narrative review" OR "meta-analysis" OR "metaanalysis" OR "meta analysis" OR "literature review" OR synthes* ) |
| **CINAHL** | October 6, 2021  August 12, 2022 | 1.811  1.361 | TX ( TX ( coronavirus OR "corona virus" OR "coronavirus infect*" OR "corona virus infect*" OR "betacoronavirus*" OR "beta coronavirus*" OR "beta corona virus*" OR wuhan OR hubei OR "severe acute respiratory syndrome coronavirus 2" OR "SARS CoV-2" OR "SARSCoV 2" OR "SARSCoV2" OR "cov2" or "sars 2" OR COVID OR "coronavirus 2" OR "covid19" OR "Covid-19" OR "Covid 19" OR nCov OR "new corona virus" OR "new coronavirus" OR "novel coronavirus" OR "novel corona virus" OR ncp OR corona OR pandemic OR "global pandemic" OR epidemic OR "global epidemic" OR coronavirinae ) AND  TX ( mental OR psycholog* OR "distress" OR "psychiatr*" OR "social stigma" OR stigma OR fear OR phobia OR anxiety OR anxi* OR stress OR worry OR "physiological stress" OR angst OR depress* OR mood OR irritab* OR sad* OR affect* OR fatigue OR hope* OR neurotic* OR grief OR griev* OR bereav* OR loss OR burnout OR "burned out" OR "burnt out" OR trauma OR trauma* OR post-traumatic OR post-trauma OR PTSD OR "Post traumatic stress disorder" OR "post-traumatic stress disorder" OR "posttraumatic stress disorder" OR anger OR "substance abuse" OR "substance use" OR "substance use disorder" OR "substance dependence" OR "drug use" OR "drug abuse" OR "drug dependence" ) AND  AB ( review OR "systematic review" OR "narrative review" OR "meta-analysis" OR "metaanalysis" OR "meta analysis" OR "literature review" OR synthes* ) ) AND  TX ( mental OR psycholog* OR "distress" OR "psychiatr*" OR "social stigma" OR stigma OR fear OR phobia OR anxiety OR anxi* OR stress OR worry OR "physiological stress" OR angst OR depress* OR mood OR irritab* OR sad* OR affect* OR fatigue OR hope* OR neurotic* OR grief OR griev* OR bereav* OR loss OR burnout OR "burned out" OR "burnt out" OR trauma OR trauma* OR post-traumatic OR post-trauma OR PTSD OR "Post traumatic stress disorder" OR "post-traumatic stress disorder" OR "posttraumatic stress disorder" OR anger OR "substance abuse" OR "substance use" OR "substance use disorder" OR "substance dependence" OR "drug use" OR "drug abuse" OR "drug dependence" ) AND  AB ( review OR "systematic review" OR "narrative review" OR "meta-analysis" OR "metaanalysis" OR "meta analysis" OR "literature review" OR synthes* ) |
| Web of Science | October 6, 2021  August 12, 2022 | 11.361  6.229 | (( ALL=(coronavirus OR "corona virus" OR "coronavirus infect*" OR "corona virus infect*" OR "betacoronavirus*" OR "beta coronavirus*" OR "beta corona virus*" OR wuhan OR hubei OR "severe acute respiratory syndrome coronavirus 2" OR "SARS CoV-2" OR "SARSCoV 2" OR "sarscov" OR "cov2" or "sars 2" OR COVID OR "coronavirus 2" OR "covid19" OR "Covid-19" OR "Covid 19" OR nCov OR "new corona virus" OR "new coronavirus" OR "novel coronavirus" OR "novel corona virus" OR ncp OR corona OR pandemic OR "global pandemic" OR epidemic OR "global epidemic" OR coronaviridae) AND  ALL=(mental OR psycholog* OR "distress" OR "psychiatr*" OR "social stigma" OR stigma OR fear OR phobia OR anxiety OR anxi* OR stress OR worry OR "physiological stress" OR angst OR depress* OR mood OR irritab* OR sad* OR affect* OR fatigue OR hope* OR neurotic* OR grief OR griev* OR bereav* OR loss OR burnout OR "burned out" OR "burnt out" OR trauma OR trauma* OR post-traumatic OR post-trauma OR PTSD OR "Post traumatic stress disorder" OR "post-traumatic stress disorder" OR "posttraumatic stress disorder" OR anger OR "substance abuse" OR "substance use" OR "substance use disorder" OR "substance dependence" OR "drug use" OR "drug abuse" OR "drug dependence") AND  AB=(review OR "systematic review" OR "narrative review" OR "meta-analysis" OR "metaanalysis" OR "meta analysis" OR "literature review" OR synthes*))) |
| Cochrane | October 6, 2021  August 12, 2022 | 306  147 | (coronavirus OR "corona virus" OR "coronavirus infect*" OR "corona virus infect*" OR "betacoronavirus*" OR "beta coronavirus*" OR "beta corona virus*" OR wuhan OR hubei OR "severe acute respiratory syndrome coronavirus 2" OR "SARS CoV-2" OR "SARSCoV 2" OR "SARSCoV2" OR "cov2" or "sars 2" OR COVID OR "coronavirus 2" OR "covid19" OR "Covid-19" OR "Covid 19" OR nCov OR "new corona virus" OR "new coronavirus" OR "novel coronavirus" OR "novel corona virus" OR ncp OR corona OR pandemic OR "global pandemic" OR epidemic OR "global epidemic" OR coronavirinae) AND (mental OR psycholog* OR "distress" OR "psychiatr*" OR "social stigma" OR stigma OR fear OR phobia OR anxiety OR anxi* OR stress OR worry OR "physiological stress" OR angst OR depress* OR mood OR irritab* OR sad* OR affect* OR fatigue OR hope* OR neurotic* OR grief OR griev* OR bereav* OR loss OR burnout OR "burned out" OR "burnt out" OR trauma OR trauma* OR post-traumatic OR post-trauma OR PTSD OR "Post traumatic stress disorder" OR "post-traumatic stress disorder" OR "posttraumatic stress disorder" OR anger OR "substance abuse" OR "substance use" OR "substance use disorder" OR "substance dependence" OR "drug use" OR "drug abuse" OR "drug dependence") |

**Note.** All searches were restricted to time-period from December 31, 2019 onwards (filter).

### **Table B.** List of excluded meta-analyses by full text screening with exclusion reason.

| **Paper (first author, year)** | **Reason** |
| --- | --- |
| 1. a, P.K., et al., *Psychological and Behavioral Impact of Lockdown and Quarantine Measures for COVID-19 Pandemic on Children, Adolescents and Caregivers: A Systematic Review and Meta-Analysis.* Journal of Tropical Pediatrics, 2021. **67**(1): p. 1-13. | Wrong outcome |
| 2. Abd-Alrazaq, A., et al., *Overview of Technologies Implemented During the First Wave of the COVID-19 Pandemic: Scoping Review.* J Med Internet Res. **23**(9): p. e29136. | Wrong outcome |
| 3. Abdo, C., et al., *Domestic violence and substance abuse during COVID19: A systematic review.* INDIAN JOURNAL OF PSYCHIATRY. **62**(9): p. 337-342. | Wrong outcome |
| 4. Abraham, A., et al., *Depression among healthcare workers in the Eastern Mediterranean Region: a systematic review and meta-analysis.* Hum Resour Health. **19**(1): p. 81. | Wrong outcome |
| 5. Ahmad, M.S., et al., *“LONG COVID”: An insight.* European Review for Medical and Pharmacological Sciences, 2021. **25**(17): p. 5561-5577. | Wrong outcome |
| 6. Ahmed, H., et al., *LONG-TERM CLINICAL OUTCOMES IN SURVIVORS OF SEVERE ACUTE RESPIRATORY SYNDROME (SARS) AND MIDDLE EAST RESPIRATORY SYNDROME (MERS) CORONAVIRUS OUTBREAKS AFTER HOSPITALISATION OR ICU ADMISSION: A SYSTEMATIC REVIEW AND META-ANALYSIS.* Journal of Rehabilitation Medicine (Stiftelsen Rehabiliteringsinformation), 2020. **52**(5): p. 1-11. | Wrong outcome |
| 7. Al Falasi, B., et al., *Prevalence and Determinants of Immediate and Long-Term PTSD Consequences of Coronavirus-Related (CoV-1 and CoV-2) Pandemics among Healthcare Professionals: A Systematic Review and Meta-Analysis.* Int J Environ Res Public Health. **18**(4). | Wrong outcome |
| 8. Alex, et al., *The impact of COVID-19 on healthcare delivery for people who use opioids: a scoping review.* Substance abuse treatment, prevention, and policy, 2021. **16**(1): p. 60. | Wrong outcome |
| 9. Alimoradi, Z., et al., *Sleep problems during COVID-19 pandemic and its' association to psychological distress: A systematic review and meta-analysis.* ECLINICALMEDICINE. **36**. | Wrong outcome |
| 10. Alimoradi, Z., et al., *Gender-specific estimates of sleep problems during the COVID-19 pandemic: Systematic review and meta-analysis.* JOURNAL OF SLEEP RESEARCH. | Wrong outcome |
| 11. Allan, S.M., et al., *The prevalence of common and stress-related mental health disorders in healthcare workers based in pandemic-affected hospitals: a rapid systematic review and meta-analysis.* Eur J Psychotraumatol. **11**(1): p. 1810903. | Wrong outcome |
| 12. Almeda, N., C. Garcia-Alonso, and L. Salvador-Carulla, *Mental health planning at a very early stage of the COVID-19 crisis: a systematic review of online international strategies and recommendations.* BMC PSYCHIATRY. **21**(1). | Wrong outcome |
| 13. Alqahtani, J.S., et al., *Prevalence, severity and mortality associated with COPD and smoking in patients with COVID-19: A rapid systematic review and meta-analysis.* PLoS ONE, 2020. **15**(5). | Wrong outcome |
| 14. Amdal, C.D., et al., *Health-related quality of life issues, including symptoms, in patients with active COVID-19 or post COVID-19; a systematic literature review.* QUALITY OF LIFE RESEARCH. | Wrong outcome |
| 15. Ardekani, A., et al., *Student support systems for undergraduate medical students during the COVID-19 pandemic: a systematic narrative review of the literature.* BMC medical education, 2021. **21**(1): p. 352. | Wrong outcome |
| 16. Armendariz-Garcia, N.A. and D. Ctr Documentacion Invest, *COVID 19 and its Impact on Drug use: Systematic Review.* EUREKA-REVISTA CIENTIFICA DE PSICOLOGIA, 2020. **17**: p. 318-332. | Wrong outcome |
| 17. Astirbadi, D. and P. Lockwood, *COVID-19: A literature review of the impact on diagnostic radiography students.* Radiography (Lond). | Wrong outcome |
| 18. Atoofi, M.K., et al., *Requirements of Mental Health Services During the COVID-19 Outbreak: A Systematic Review.* IRANIAN JOURNAL OF PSYCHIATRY AND CLINICAL PSYCHOLOGY. **26**(3): p. 264-279. | Wrong outcome |
| 19. Barello, S., et al., *The psychosocial impact of flu influenza pandemics on healthcare workers and lessons learnt for the COVID-19 emergency: a rapid review.* INTERNATIONAL JOURNAL OF PUBLIC HEALTH. **65**(7): p. 1205-1216. | Wrong outcome |
| 20. Baskin, R.G. and R. Bartlett, *Healthcare worker resilience during the COVID-19 pandemic: An integrative review.* JOURNAL OF NURSING MANAGEMENT. | Wrong outcome |
| 21. Batista, P., et al., *Anxiety impact during COVID-19: a systematic review.* J Infect Dev Ctries. **15**(3): p. 320-325. | Wrong outcome |
| 22. Baumgart, J.G., et al., *The early impacts of the covid-19 pandemic on mental health facilities and psychiatric professionals.* International Journal of Environmental Research and Public Health, 2021. **18**(15). | Wrong outcome |
| 23. Bekele, F., et al., *Patterns and associated factors of COVID-19 knowledge, attitude, and practice among general population and health care workers: A systematic review.* SAGE Open Medicine, 2020. **8**. | Wrong outcome |
| 24. Bell, V. and D. Wade, *Mental health of clinical staff working in high-risk epidemic and pandemic health emergencies a rapid review of the evidence and living meta-analysis.* Social Psychiatry and Psychiatric Epidemiology: The International Journal for Research in Social and Genetic Epidemiology and Mental Health Services, 2020. | Wrong outcome |
| 25. Berger, E., et al., *Review: The mental health implications for children and adolescents impacted by infectious outbreaks – a systematic review.* Child & Adolescent Mental Health, 2021. **26**(2): p. 157-166. | Wrong outcome |
| 26. Bertuzzi, V., et al., *Psychological Support Interventions for Healthcare Providers and Informal Caregivers during the COVID-19 Pandemic: A Systematic Review of the Literature.* Int J Environ Res Public Health. **18**(13). | Wrong outcome |
| 27. Boden, M., et al., *Mental disorder prevalence among populations impacted by coronavirus pandemics: A multilevel meta-analytic study of COVID-19, MERS & SARS.* General Hospital Psychiatry, 2021. **70**: p. 124-133. | Wrong outcome |
| 28. Boldt, K., et al., *Interventions to Ameliorate the Psychosocial Effects of the COVID-19 Pandemic on Children-A Systematic Review.* Int J Environ Res Public Health. **18**(5). | Wrong outcome |
| 29. Brodeur, M., et al., *Gambling and the COVID-19 pandemic: A scoping review.* Progress in Neuro-Psychopharmacology and Biological Psychiatry, 2021. **111**. | Wrong outcome |
| 30. Brooks, S.K., D. Weston, and N. Greenberg, *Social and psychological impact of the COVID-19 pandemic on people with Parkinson's disease: a scoping review.* Public Health, 2021. **199**: p. 77-86. | Wrong outcome |
| 31. Brown, E., et al., *The potential impact of COVID-19 on psychosis: A rapid review of contemporary epidemic and pandemic research.* Schizophrenia Research, 2020. **222**: p. 79-87. | Wrong outcome |
| 32. Buselli, R., et al., *Mental health of Health Care Workers (HCWs): a review of organizational interventions put in place by local institutions to cope with new psychosocial challenges resulting from COVID-19.* PSYCHIATRY RESEARCH. **299**. | Wrong outcome |
| 33. C. Fong, V. and G. Iarocci, *Child and family outcomes following pandemics: A systematic review and recommendations on COVID-19 policies.* Journal of Pediatric Psychology, 2020. **45**(10): p. 1124-1143. | Wrong outcome |
| 34. Cabarkapa, S., et al., *Psychological impact of COVID-19 and other viral epidemics on frontline healthcare workers: A systematic review.* Asia-Pacific Psychiatry, 2021. **13**. | Wrong outcome |
| 35. Cabrera Martimbianco, A.L., et al., *Frequency, signs and symptoms, and criteria adopted for long COVID-19: A systematic review.* Int J Clin Pract. **75**(10): p. e14357. | Wrong outcome |
| 36. Cappa, C. and I. Jijon, *COVID-19 and violence against children: A review of early studies.* Child Abuse and Neglect, 2021. | Wrong outcome |
| 37. Carmassi, C., et al., *PTSD symptoms in healthcare workers facing the three coronavirus outbreaks: What can we expect after the COVID-19 pandemic.* PSYCHIATRY RESEARCH. **292**. | Wrong outcome |
| 38. Carotenuto, A., et al., *Tele-Neuropsychological Assessment of Alzheimer's Disease.* J Pers Med. **11**(8). | Wrong outcome |
| 39. Chaabane, S., et al., *The Impact of COVID-19 School Closure on Child and Adolescent Health: A Rapid Systematic Review.* Children, 2021. **8**(5): p. 1-17. | Wrong outcome |
| 40. Chiesa, V., et al., *COVID-19 pandemic: health impact of staying at home, social distancing and 'lockdown' measures-a systematic review of systematic reviews.* J Public Health (Oxf). **43**(3): p. e462-e481. | Wrong outcome |
| 41. Chmielewska, B., et al., *Effects of the COVID-19 pandemic on maternal and perinatal outcomes: a systematic review and meta-analysis.* The Lancet Global Health, 2021. **9**(6): p. e759-e772. | Wrong outcome |
| 42. Chow, K.M., et al., *A review of psychological issues among patients and healthcare staff during two major coronavirus disease outbreaks in China: Contributory factors and management strategies.* International Journal of Environmental Research and Public Health, 2020. **17**(18): p. 1-17. | Wrong outcome |
| 43. Chu, I.Y., et al., *Social consequences of mass quarantine during epidemics: a systematic review with implications for the COVID-19 response.* J Travel Med. **27**(7). | Wrong outcome |
| 44. Ch, et al., *Measuring the Impact of COVID-19 on Mental Health: A Scoping Review of the Existing Scales.* Indian J Psychol Med. **42**(5): p. 421-427. | Wrong outcome |
| 45. Cielo, F., R. Ulberg, and D. Di Giacomo, *Psychological impact of the covid-19 outbreak on mental health outcomes among youth: A rapid narrative review.* International Journal of Environmental Research and Public Health, 2021. **18**(11). | Wrong outcome |
| 46. Crook, H., et al., *Long covid - Mechanisms, risk factors, and management.* The BMJ, 2021. **374**. | Wrong outcome |
| 47. Daroische, R., et al., *Cognitive Impairment After COVID-19—A Review on Objective Test Data.* Frontiers in Neurology, 2021. **12**. | Wrong outcome |
| 48. Datta, K. and M. Tripathi, *Sleep and Covid-19.* Neurology India, 2021. **69**(1): p. 26-31. | Wrong outcome |
| 49. Davison, K.M., et al., *Interventions to Support Mental Health among Those with Health Conditions That Present Risk for Severe Infection from Coronavirus Disease 2019 (COVID-19): A Scoping Review of English and Chinese-Language Literature.* INTERNATIONAL JOURNAL OF ENVIRONMENTAL RESEARCH AND PUBLIC HEALTH. **18**(14). | Wrong outcome |
| 50. De Brier, N., et al., *Factors affecting mental health of health care workers during coronavirus disease outbreaks (SARS, MERS & COVID-19): A rapid systematic review.* PLoS ONE, 2020. **15**(12). | Wrong outcome |
| 51. de Carvalho, S.G., A.B.S. dos Santos, and I.M. Santos, *The pandemic in prison: interventions and overisolation.* CIENCIA & SAUDE COLETIVA. **25**(9): p. 3493-3502. | Wrong outcome |
| 52. Dean, E. and M.F. Olsen, *A health and lifestyle framework for management of post COVID-19 syndrome based on evidence-informed management of post-polio syndrome: a narrative review.* EUROPEAN JOURNAL OF PHYSIOTHERAPY. | Wrong outcome |
| 53. Dellazizzo, L., et al., *Systematic Review on the Mental Health and Treatment Impacts of COVID-19 on Neurocognitive Disorders.* JOURNAL OF PERSONALIZED MEDICINE. **11**(8). | Wrong outcome |
| 54. Di Giacomo, D., et al., *Mediator effect of affinity for e-learning on mental health: Buffering strategy for the resilience of university students.* International Journal of Environmental Research and Public Health, 2021. **18**(13). | Wrong outcome |
| 55. Drudi, L.M., et al., *The impact of the COVID-19 pandemic on wellness among vascular surgeons.* Seminars in Vascular Surgery, 2021. **34**(2): p. 43-50. | Wrong outcome |
| 56. Egbert, A.R., S. Cankurtaran, and S. Karpiak, *Brain abnormalities in COVID-19 acute/subacute phase: A rapid systematic review.* Brain, Behavior, and Immunity, 2020. **89**: p. 543-554. | Wrong outcome |
| 57. Fan, J., et al., *An Umbrella Review of the Work and Health Impacts of Working in an Epidemic/Pandemic Environment.* Int J Environ Res Public Health. **18**(13). | Wrong outcome |
| 58. Farsalinos, K., et al., *Current smoking, former smoking, and adverse outcome among hospitalized COVID-19 patients: a systematic review and meta-analysis.* Therapeutic Advances in Chronic Disease, 2020. **11**. | Wrong outcome |
| 59. Finstad, G.L., et al., *Resilience, Coping Strategies and Posttraumatic Growth in the Workplace Following COVID-19: A Narrative Review on the Positive Aspects of Trauma.* INTERNATIONAL JOURNAL OF ENVIRONMENTAL RESEARCH AND PUBLIC HEALTH. **18**(18). | Wrong outcome |
| 60. Firouzkouhi, M., et al., *Bereaved Families Views on the Death of Loved Ones Due to COVID 19: An Integrative Review.* OMEGA-JOURNAL OF DEATH AND DYING. | Wrong outcome |
| 61. Galanis, P., et al., *Nurses' burnout and associated risk factors during the COVID-19 pandemic: A systematic review and meta-analysis.* Journal of Advanced Nursing (John Wiley & Sons, Inc.), 2021. **77**(8): p. 3286-3302. | Wrong outcome |
| 62. Gianfredi, V., et al., *What can internet users' behaviours reveal about the mental health impacts of the COVID-19 pandemic? A systematic review.* Public Health (Elsevier), 2021. **198**: p. 44-52. | Wrong outcome |
| 63. Gibson, B., et al., *The Impact of Inequality on Mental Health Outcomes During the COVID-19 Pandemic: A Systematic Review.* Canadian Psychology, 2021. **62**(1): p. 101-126. | Wrong outcome |
| 64. Gross, J.V., J. Mohren, and T.C. Erren, *COVID-19 and healthcare workers: A rapid systematic review into risks and preventive measures.* BMJ Open, 2021. **11**(1). | Wrong outcome |
| 65. Gupta, S. and S. Sahoo, *Pandemic and mental health of the front-line healthcare workers: A review and implications in the Indian context amidst COVID-19.* General Psychiatry, 2020. **33**(5): p. 1-10. | Wrong outcome |
| 66. Hesary, F.B. and H. Salehiniya, *The Impact of the COVID-19 Epidemic on Diagnosis, Treatment, Concerns, Problems, and Mental Health in Patients with Gastric Cancer.* JOURNAL OF GASTROINTESTINAL CANCER. | Wrong outcome |
| 67. Hintermeier, M., et al., *SARS-CoV-2 among migrants and forcibly displaced populations: A rapid systematic review.* J Migr Health, 2021. **4**: p. 100056. | Wrong outcome |
| 68. Hodgins, D.C. and R.M.G. Stevens, *The impact of COVID-19 on gambling and gambling disorder: emerging data.* Current Opinion in Psychiatry, 2021. **34**(4): p. 332-343. | Wrong outcome |
| 69. Hou, H., et al., *Smoking is independently associated with an increased risk for COVID-19 mortality: A systematic review and meta-analysis based on adjusted effect estimates.* Nicotine & tobacco research : official journal of the Society for Research on Nicotine and Tobacco, 2021. | Wrong outcome |
| 70. Hughes, M.C., Y. Liu, and A. Baumbach, *Impact of COVID-19 on the Health and Well-being of Informal Caregivers of People with Dementia: A Rapid Systematic Review.* Gerontology and Geriatric Medicine, 2021. **7**. | Wrong outcome |
| 71. Ilmy, S.K., N. Noorhamdani, and H.D. Windarwati, *Family Burden of Schizophrenia in Pasung During COVID-19 Pandemic: A Scoping Review.* Indonesian Nursing Journal of Education & Clinic (INJEC), 2020. **5**(2): p. 152-163. | Wrong outcome |
| 72. Imran, N., et al., *Psychological burden of quarantine in children and adolescents: A rapid systematic review and proposed solutions.* Pakistan Journal of Medical Sciences, 2020. **36**(5): p. 1106-1116. | Wrong outcome |
| 73. Iwu, C.J., C.D. Iwu, and C.S. Wiysonge, *The occurrence of long COVID: A rapid review.* Pan African Medical Journal, 2021. **38**: p. 1-12. | Wrong outcome |
| 74. Jahrami, H., et al., *Sleep problems during the COVID-19 pandemic by population: A systematic review and meta-analysis.* Journal of Clinical Sleep Medicine, 2021. **17**(2): p. 299-313. | Wrong outcome |
| 75. Jammu, A.S., et al., *Systematic rapid living review of the impact of the COVID-19 pandemic on cancer survivors: update to August 27, 2020.* Supportive Care in Cancer, 2021. **29**(6): p. 2841-2850. | Wrong outcome |
| 76. Jesus, T.S., et al., *Lockdown-Related Disparities Experienced by People with Disabilities during the First Wave of the COVID-19 Pandemic: Scoping Review with Thematic Analysis.* Int J Environ Res Public Health. **18**(12). | Wrong outcome |
| 77. John, A., et al., *The impact of the COVID-19 pandemic on self-harm and suicidal behaviour: update of living systematic review.* F1000Res, 2020. **9**: p. 1097. | Wrong outcome |
| 78. Joo, J.Y. and M.F. Liu, *Nurses' barriers to caring for patients with COVID‐19: a qualitative systematic review.* International Nursing Review, 2021. **68**(2): p. 202-213. | Wrong outcome |
| 79. Jurecka, A., P. Skucinska, and A. Gadek, *Impact of the SARS-CoV-2 Coronavirus Pandemic on Physical Activity, Mental Health and Quality of Life in Professional Athletes-A Systematic Review.* INTERNATIONAL JOURNAL OF ENVIRONMENTAL RESEARCH AND PUBLIC HEALTH. **18**(17). | Wrong outcome |
| 80. Kar, S.K., et al., *Mental health research in the lower-middle-income countries of Africa and Asia during the COVID-19 pandemic: A scoping review.* Neurology Psychiatry and Brain Research, 2020. **38**: p. 54-64. | Wrong outcome |
| 81. Kirubarajan, A., et al., *The psychological impact of the COVID-19 pandemic on fertility care: a qualitative systematic review.* HUMAN FERTILITY. | Wrong outcome |
| 82. Klinger, C., et al., *Unintended health and societal consequences of international travel measures during the COVID-19 pandemic: A scoping review.* J Travel Med. | Wrong outcome |
| 83. Kotlar, B., et al., *The impact of the COVID-19 pandemic on maternal and perinatal health: a scoping review.* Reproductive Health, 2021. **18**(1): p. 1-39. | Wrong outcome |
| 84. Kourti, A., et al., *Domestic Violence During the COVID-19 Pandemic: A Systematic Review.* TRAUMA VIOLENCE & ABUSE. | Wrong outcome |
| 85. Kourti, A., et al., *Play Behaviors in Children during the COVID-19 Pandemic: A Review of the Literature.* Children, 2021. **8**(8): p. 1-18. | Wrong outcome |
| 86. Kraef, C., M. van der Meirschen, and C. Free, *Digital telemedicine interventions for patients with multimorbidity: a systematic review and meta-analysis.* BMJ Open. **10**(10): p. e036904. | Wrong outcome |
| 87. Kunzler, A.M., et al., *Mental health and psychosocial support strategies in highly contagious emerging disease outbreaks of substantial public concern: A systematic scoping review.* PLoS ONE, 2021. **16**(2). | Wrong outcome |
| 88. Kuroda, N. and A. Fujimoto, *Considering temporality in causal relationship between seizure worsening and psychological stress in patients with epilepsy during the COVID-19 pandemic: A systematic review.* Epilepsy and Behavior, 2021. **122**. | Wrong outcome |
| 89. Labrague, L.J., *Psychological resilience, coping behaviours and social support among health care workers during the COVID-19 pandemic: A systematic review of quantitative studies.* JOURNAL OF NURSING MANAGEMENT. | Wrong outcome |
| 90. Lateef, R., R. Alaggia, and D. Collin-Vezina, *A scoping review on psychosocial consequences of pandemics on parents and children: Planning for today and the future.* CHILDREN AND YOUTH SERVICES REVIEW. **125**. | Wrong outcome |
| 91. Lau, N., et al., *Telemental health for youth with chronic illnesses: Systematic review.* JMIR Mental Health, 2021. **8**(8). | Wrong outcome |
| 92. Lausi, G., et al., *Intimate Partner Violence during the COVID-19 Pandemic: A Review of the Phenomenon from Victims' and Help Professionals' Perspectives.* Int J Environ Res Public Health. **18**(12). | Wrong outcome |
| 93. Law, J., et al., *Tele-practice for children and young people with communication disabilities: Employing the COM-B model to review the intervention literature and inform guidance for practitioners.* INTERNATIONAL JOURNAL OF LANGUAGE & COMMUNICATION DISORDERS. **56**(2): p. 415-434. | Wrong outcome |
| 94. Lazzari, C. and M. Rabottini, *COVID-19, loneliness, social isolation and risk of dementia in older people: a systematic review and meta-analysis of the relevant literature.* INTERNATIONAL JOURNAL OF PSYCHIATRY IN CLINICAL PRACTICE. | Wrong outcome |
| 95. Lebrasseur, A., et al., *Impact of COVID-19 on people with physical disabilities: A rapid review.* DISABILITY AND HEALTH JOURNAL. **14**(1). | Wrong outcome |
| 96. Lebrasseur, A., et al., *Impact of the COVID-19 Pandemic on Older Adults: Rapid Review.* JMIR Aging. **4**(2): p. e26474. | Wrong outcome |
| 97. Li, H., et al., *Transition of Mental Health Service Delivery to Telepsychiatry in Response to COVID-19: A Literature Review.* Psychiatric Quarterly, 2021. | Wrong outcome |
| 98. Lin, Y.N., et al., *Burden of Sleep Disturbance During COVID-19 Pandemic: A Systematic Review.* Nat Sci Sleep, 2021. **13**: p. 933-966. | Wrong outcome |
| 99. Liu, L., et al., *Mental and neurological disorders and risk of COVID-19 susceptibility, illness severity and mortality: A systematic review, meta-analysis and call for action.* EClinicalMedicine. **40**: p. 101111. | Wrong outcome |
| 100. Liu, M., et al., *Drinking no-links to the severity of COVID-19: a systematic review and meta-analysis: Drinking and COVID-19.* Journal of Infection, 2020. **81**(2): p. e126-e127. | Wrong outcome |
| 101. Loreto, B.B.L., et al., *Well-being at work, productivity and coping with stress during the COVID-19 pandemic.* Trends Psychiatry Psychother. | Wrong outcome |
| 102. Lu, C., et al., *The perspective and need of health-care workers for COVID-19 on the frontline: A systematic review of qualitative research.* Global Advances in Health and Medicine, 2021. **10**: p. 25-26. | Wrong outcome |
| 103. Magklara, E., et al., *The role of medical students during COVID-19 era. A review.* Acta Biomedica, 2021. **92**(1). | Wrong outcome |
| 104. Magnavita, N., et al., *Sars/mers/sars-cov-2 outbreaks and burnout syndrome among healthcare workers. An umbrella systematic review.* International Journal of Environmental Research and Public Health, 2021. **18**(8). | Wrong outcome |
| 105. Majrashi, A., et al., *Stressors and Coping Strategies among Nursing Students during the COVID-19 Pandemic: Scoping Review.* NURSING REPORTS. **11**(2): p. 444-459. | Wrong outcome |
| 106. Masaeli, N. and H. Farhadi, *Prevalence of Internet-based addictive behaviors during COVID-19 pandemic: a systematic review.* JOURNAL OF ADDICTIVE DISEASES. | Wrong outcome |
| 107. McGowan, V.J., H.J. Lowther, and C. Meads, *Life under COVID-19 for LGBT+ people in the UK: systematic review of UK research on the impact of COVID-19 on sexual and gender minority populations.* BMJ Open. **11**(7): p. e050092. | Wrong outcome |
| 108. Medina-Ortiz, O., et al., *Sleep disorders as a result of the covid-19 pandemic.* Revista Peruana de Medicina Experimental y Salud Publica, 2020. **37**(4): p. 755-761. | Wrong outcome |
| 109. Menculini, G., et al., *The influence of the urban environment on mental health during the covid-19 pandemic: Focus on air pollution and migration—a narrative review.* International Journal of Environmental Research and Public Health, 2021. **18**(8). | Wrong outcome |
| 110. Min Ji, K. and P. Jeong Hun, *The effects of COVID-19 on physician's burnout: a systematic review.* Journal of the Korean Medical Association / Taehan Uisa Hyophoe Chi, 2021. **64**(9): p. 636-646. | Wrong outcome |
| 111. Monroy-Fraustro, D., et al., *Bibliotherapy as a Non-pharmaceutical Intervention to Enhance Mental Health in Response to the COVID-19 Pandemic: A Mixed-Methods Systematic Review and Bioethical Meta-Analysis.* FRONTIERS IN PUBLIC HEALTH. **9**. | Wrong outcome |
| 112. Morrish, N. and A. Medina-Lara, *Does unemployment lead to greater levels of loneliness? A systematic review.* Social Science & Medicine, 2021. **287**: p. N.PAG-N.PAG. | Wrong outcome |
| 113. Murphy, L., et al., *The impact of the COVID-19 pandemic and its related restrictions on people with pre-existent mental health conditions: A scoping review.* Arch Psychiatr Nurs. **35**(4): p. 375-394. | Wrong outcome |
| 114. Nowrouzi-Kia, B., et al., *Factors associated with work performance and mental health of healthcare workers during pandemics: a systematic review and meta-analysis.* Journal of public health (Oxford, England), 2021. | Wrong outcome |
| 115. Nursalam, N., et al., *Risk factors for psychological impact and social stigma among people facing COVID 19: A systematic review.* Systematic Reviews in Pharmacy, 2020. **11**(6): p. 1022-1028. | Wrong outcome |
| 116. Orfao, N.H., et al., *COVID-19: coping strategies and adaptive behaviors adopted by health professionals during the pandemic.* REVISTA DE EPIDEMIOLOGIA E CONTROLE DE INFECCAO, 2020. **10**(4). | Wrong outcome |
| 117. Pai, N. and S.L. Vella, *COVID-19 and loneliness: A rapid systematic review.* AUSTRALIAN AND NEW ZEALAND JOURNAL OF PSYCHIATRY. | Wrong outcome |
| 118. Paiano, M., et al., *Mental health of healthcare professionals in China during the new coronavirus pandemic: an integrative review.* Revista brasileira de enfermagem, 2020. **73**: p. e20200338. | Wrong outcome |
| 119. Pâmela Schultz, D., S.A. Cláudia Pinto da, and G.F. Miguel Nascimento, *Atuação do psicólogo na saúde mental da população diante da pandemia.* Journal of Nursing & Health, 2020. **10**(4): p. 1-14. | Wrong outcome |
| 120. Peters, E.M.J., et al., *Can Stress Interact with SARS-CoV-2? A Narrative Review with a Focus on Stress-Reducing Interventions that may Improve Defence against COVID-19.* PPmP Psychotherapie Psychosomatik Medizinische Psychologie, 2021. **71**(2): p. 61-71. | Wrong outcome |
| 121. Preston, A.J. and L. Rew, *Connectedness, Self-Esteem, and Prosocial Behaviors Protect Adolescent Mental Health Following Social Isolation: A Systematic Review.* ISSUES IN MENTAL HEALTH NURSING. | Wrong outcome |
| 122. Rahman, M., et al., *Mental Distress and Human Rights Violations During COVID-19: A Rapid Review of the Evidence Informing Rights, Mental Health Needs, and Public Policy Around Vulnerable Populations.* Frontiers in Psychiatry, 2020. **11**. | Wrong outcome |
| 123. Rapp, A., et al., *Child Maltreatment During the COVID-19 Pandemic: A Systematic Rapid Review.* Pediatric Clinics of North America, 2021. **68**(5): p. 991-1009. | Wrong outcome |
| 124. Reardon, M., et al., *Review article: Prevalence of burnout in paramedics: A systematic review of prevalence studies.* Emerg Med Australas. **32**(2): p. 182-189. | Wrong outcome |
| 125. Rieckert, A., et al., *How can we build and maintain the resilience of our health care professionals during COVID-19? Recommendations based on a scoping review.* BMJ OPEN, 2021. **11**(1). | Wrong outcome |
| 126. Rivera-Torres, S., et al., *Older Adults' Mental Health Through Leisure Activities During COVID-19: A Scoping Review.* Gerontol Geriatr Med. **7**: p. 23337214211036776. | Wrong outcome |
| 127. Rocha, D.D., et al., *Psychosocial effects of social distancing during coronavirus infections: integrative review.* ACTA PAULISTA DE ENFERMAGEM, 2021. **34**. | Wrong outcome |
| 128. Rogers, J.P., et al., *Neurology and neuropsychiatry of COVID-19: A systematic review and meta-analysis of the early literature reveals frequent CNS manifestations and key emerging narratives.* Journal of Neurology, Neurosurgery and Psychiatry, 2021. **92**(9): p. 932-941. | Wrong outcome |
| 129. Sahebi, A., et al., *The prevalence of insomnia among health care workers amid the COVID-19 pandemic: An umbrella review of meta-analyses.* Journal of Psychosomatic Research, 2021. **149**: p. N.PAG-N.PAG. | Wrong outcome |
| 130. Salari, N., et al., *The prevalence of sleep disturbances among physicians and nurses facing the COVID-19 patients: a systematic review and meta-analysis.* Globalization & Health, 2020. **16**(1): p. N.PAG-N.PAG. | Wrong outcome |
| 131. Sarangi, A., T. McMahon, and J. Gude, *Benzodiazepine Misuse: An Epidemic Within a Pandemic.* CUREUS. **13**(6). | Wrong outcome |
| 132. Schmidt, R.A., et al., *The early impact of COVID-19 on the incidence, prevalence, and severity of alcohol use and other drugs: A systematic review.* Drug Alcohol Depend. **228**: p. 109065. | Wrong outcome |
| 133. Schou, T.M., et al., *Psychiatric and neuropsychiatric sequelae of COVID-19 – A systematic review.* Brain, Behavior, and Immunity, 2021. **97**: p. 328-348. | Wrong outcome |
| 134. Schubert, M., et al., *Stigmatization from Work-Related COVID-19 Exposure: A Systematic Review with Meta-Analysis.* Int J Environ Res Public Health. **18**(12). | Wrong outcome |
| 135. Serrano-Ripoll, M.J., et al., *Insomnia and sleep quality in healthcare workers fighting against COVID-19: a systematic review of the literature and meta-analysis.* Actas Esp Psiquiatr. **49**(4): p. 155-179. | Wrong outcome |
| 136. Shankar, A., B.E. Yu, and M. Malvankar-Mehta, *The psychological impact of COVID-19 on socially isolated individuals – a systematic review.* Mental Health Review Journal, 2021. **26**(3): p. 247-257. | Wrong outcome |
| 137. Shiozawa, P. and R.R. Uchida, *An updated systematic review on the coronavirus pandemic: Lessons for psychiatry.* Brazilian Journal of Psychiatry, 2020. **42**(3): p. 330-331. | Wrong outcome |
| 138. Singh, K.P. and R. Agarwal, *Neurological Manifestations in COVID-19 Population: A Short Review.* Annals of Neurosciences, 2021. | Wrong outcome |
| 139. Singh, S., et al., *Impact of COVID-19 and lockdown on mental health of children and adolescents: A narrative review with recommendations.* Psychiatry Research, 2020. **293**. | Wrong outcome |
| 140. Sirois, F.M. and J. Owens, *Factors Associated With Psychological Distress in Health-Care Workers During an Infectious Disease Outbreak: A Rapid Systematic Review of the Evidence.* Frontiers in Psychiatry, 2020. **11**. | Wrong outcome |
| 141. Soltani, S., et al., *COVID-19 associated central nervous system manifestations, mental and neurological symptoms: A systematic review and meta-analysis.* Reviews in the Neurosciences, 2021. **32**(3): p. 351-361. | Wrong outcome |
| 142. Sousa Neto, A.R., et al., *Symptomatic manifestations of the disease caused by coronavirus (COVID-19) in adults: systematic review.* Rev Gaucha Enferm, 2021. **42**: p. e20200205. | Wrong outcome |
| 143. Stavridou, A., et al., *Psychosocial consequences of COVID-19 in children, adolescents and young adults: A systematic review.* Psychiatry and Clinical Neurosciences, 2020. **74**(11): p. 615-616. | Wrong outcome |
| 144. Szemik, S., M. Gajda, and M. Kowalska, *[The review of prospective studies on mental health and the quality of life of physicians and medical students].* Med Pr. **71**(4): p. 483-491. | Wrong outcome |
| 145. Talebi-Azar, N., B.C. Anzali, and R. Goli, *COVID-19 and its mental health effects on nurses and health workers ⇓ a narrative review.* Pakistan Journal of Medical and Health Sciences, 2020. **14**(4): p. 1453-1456. | Wrong outcome |
| 146. Tang, X.F., et al., *Psychological risk and protective factors associated with depressive symptoms among adolescents in secondary schools in China: A systematic review and meta-analysis.* CHILDREN AND YOUTH SERVICES REVIEW. **108**. | Wrong outcome |
| 147. Tang, X., et al., *Psychosocial risk factors associated with depressive symptoms among adolescents in secondary schools in mainland china: A systematic review and meta-analysis.* J Affect Disord. **263**: p. 155-165. | Wrong outcome |
| 148. Tibubos, A.N., et al., *A Systematic Review on Sex- and Gender-Sensitive Research in Public Mental Health During the First Wave of the COVID-19 Crisis.* Front Psychiatry, 2021. **12**: p. 712492. | Wrong outcome |
| 149. Tolsa, M.D. and O. Malas, *COVID-19: Psychological Impact, Risk Factors and Psychological Interventions in Healthcare Personnel. A Systematic Review.* REVISTA IBEROAMERICANA DE PSICOLOGIA Y SALUD. **12**(2): p. 58-75. | Wrong outcome |
| 150. Troglio da Silva, F.C. and M.L.R. Neto, *Psychiatric disorders in health professionals during the COVID-19 pandemic: A systematic review with meta-analysis.* J Psychiatr Res. **140**: p. 474-487. | Wrong outcome |
| 151. Troglio da Silva, F.C. and M.L.R. Neto, *The impact of the COVID-19 pandemic in an intensive care unit (ICU): Psychiatric symptoms in healthcare professionals – A systematic review.* Journal of Psychiatric Research, 2021. **140**: p. 474-487. | Wrong outcome |
| 152. Usher, K., et al., *Pandemic‐related behaviours and psychological outcomes; A rapid literature review to explain COVID‐19 behaviours.* International Journal of Mental Health Nursing, 2020. **29**(6): p. 1018-1034. | Wrong outcome |
| 153. Venegas Tresierra, C.E. and A.C. Leyva Pozo, *[Fatigue and mental workload among workers: about social distancing.].* Rev Esp Salud Publica. **94**. | Wrong outcome |
| 154. Winwood, J.J., et al., *Exploring the Social Impacts of the COVID-19 Pandemic on People Living with HIV (PLHIV): A Scoping Review.* AIDS and behavior, 2021. | Wrong outcome |
| 155. Yamamoto, V., et al., *COVID-19: Review of a 21st Century Pandemic from Etiology to Neuro-psychiatric Implications.* Journal of Alzheimer's Disease, 2020. **77**(2): p. 459-504. | Wrong outcome |
| 156. Yılmaz, B., M. Azak, and N. Şahin, *Mental health of parents of children with autism spectrum disorder during COVID-19 pandemic: A systematic review.* World J Psychiatry. **11**(7): p. 388-402. | Wrong outcome |
| 157. Yuan, K., et al., *Prevalence of posttraumatic stress disorder after infectious disease pandemics in the twenty-first century, including COVID-19: a meta-analysis and systematic review.* Molecular Psychiatry, 2021. | Wrong outcome |
| 158. Zhang, Y., et al., *Anxiety and Depression in Chinese Students During the COVID-19 Pandemic: A Meta-Analysis.* Frontiers in public health, 2021. **9**: p. 697642. | Wrong outcome |
| 159. Fond G, et al. Association Between Mental Health Disorders and Mortality Among Patients With COVID-19 in 7 Countries: A Systematic Review and Meta-analysis. JAMA Psychiatry. 2021 Nov 1;78(11):1208-1217. doi: 10.1001/jamapsychiatry.2021.2274. | Wrong outcome |
| 160. Ghahramani S, et al. A Systematic Review and Meta-Analysis of Burnout Among Healthcare Workers During COVID-19. Front Psychiatry. 2021 Nov 10;12:758849. doi: 10.3389/fpsyt.2021.758849. | Wrong outcome |
| 161. Jin Y, Sun T, Zheng P, An J. Mass quarantine and mental health during COVID-19: A meta-analysis. J Affect Disord. 2021 Dec 1;295:1335-1346. doi: 10.1016/j.jad.2021.08.067. Epub 2021 Sep 2. | Wrong outcome |
| 162. Lee, Y., Jeon, Y.J., Kang, S. et al. Social media use and mental health during the COVID-19 pandemic in young adults: a meta-analysis of 14 cross-sectional studies. BMC Public Health 22, 995 (2022). | Wrong outcome |
| 163. Liu, L. et al. Mental and neurological disorders and risk of COVID-19 susceptibility, illness severity and mortality: A systematic review, meta-analysis and call for action 2021 | Wrong outcome |
| 164. Luo, Y. and Zhang, K. and Huang, M. and Qiu, C. Risk factors for depression and anxiety in pregnant women during the COVID-19 pandemic: Evidence from meta-analysis 2022 | Wrong outcome |
| 165. Malik, P. and Patel, K. and Pinto, C. and Jaiswal, R. and Tirupathi, R. and Pillai, S. and Patel, U. Post-acute COVID-19 syndrome (PCS) and health-related quality of life (HRQoL)-A systematic review and meta-analysis 2022 | Wrong outcome |
| 166. Mermerkaya, S. and Çinar, F. The effects on mental health of nurses during the COVID-19 pandemic: A systematic review and metaanalysis 2022 | Wrong outcome |
| 167. Metin, A. and Erbiçer, E. S. and Şen, S. and Çetinkaya, A. Gender and COVID-19 related fear and anxiety: A meta-analysis 2022 | Wrong outcome |
| 168. Nam, S. H. and Nam, J. H. and Kwon, C. Y. Comparison of the Mental Health Impact of COVID-19 on Vulnerable and Non-Vulnerable Groups: A Systematic Review and Meta-Analysis of Observational Studies 2021 | Wrong outcome |
| 169. Negahi, A. and Nafissi, N. and Eghbali, F. and Nouri, B. and Nassiri, S. and Nasiri, M. A Literature Review about Impact of COVID-19 on Operative Activity, Educational Process and Mental Health of Surgical Residents 2021 | Wrong outcome |
| 170. Nyberg, A. and Rajaleid, K. and Demmelmaier, I. The Work Environment during Coronavirus Epidemics and Pandemics: A Systematic Review of Studies Using Quantitative, Qualitative, and Mixed-Methods Designs 2022 | Wrong outcome |
| 171. Oostrom, T. G. A. and Cullen, P. and Peters, S. A. E. The indirect health impacts of the COVID-19 pandemic on children and adolescents: A review | Wrong outcome |
| 172. Pabis, P. and Smolarczyk- Kosowska, J. and Piegza, M. The impact of the COVID-19 pandemic on the schizophrenia: A literature review 2021 | Wrong outcome |
| 173. Pai, N. and Vella, S. L. COVID-19 and loneliness: A rapid systematic review 2021 | Wrong outcome |
| 174. Panchal, Urvashi and Salazar de Pablo, Gonzalo and Franco, Macarena and Moreno, Carmen and Parellada, Mara and Arango, Celso and Fusar-Poli, Paolo The impact of covid-19 lockdown on child and adolescent mental health: Systematic review 2021 | Wrong outcome |
| 175. Rajkumar, E. and Rajan, A. M. and Daniel, M. and Lakshmi, R. and John, R. and George, A. J. and Abraham, J. and Varghese, J. The psychological impact of quarantine due to COVID-19: A systematic review of risk, protective factors and interventions using socio-ecological model framework 2022 | Wrong outcome |
| 176. Roy, Adrija and Singh, Arvind Kumar and Mishra, Shree and Chinnadurai, Aravinda and Mitra, Arun and Bakshi, Ojaswini Mental health implications of COVID-19 pandemic and its response in India 2021 | Wrong outcome |
| 177. Roy, Devlina and Ghosh, Ritwik and Dubey, Souvik and Dubey, Mahua Jana and Benito-León, Julián and Kanti Ray, Biman Neurological and neuropsychiatric impacts of COVID-19 pandemic 2021 | Wrong outcome |
| 178. Alimoradi Z, Ohayon MM, Griffiths MD, Lin CY, Pakpour AH. Fear of COVID-19 and its association with mental health-related factors: systematic review and meta-analysis. BJPsych Open. 2022 Mar 21;8(2):e73. doi: 10.1192/bjo.2022.26. PMID: 35307051; PMCID: PMC8943231. | Wrong outcome |
| 179. Caruso R, Annaloro C, Arrigoni C, Ghizzardi G, Dellafiore F, Magon A, Maga G, Nania T, Pittella F, Villa G. Burnout and post-traumatic stress disorder in frontline nurses during the COVID-19 pandemic: a systematic literature review and meta-analysis of studies published in 2020: COVID-19, burnout, and PTSD in nurses . Acta Biomed [Internet]. 2021 Dec. 21 [cited 2023 Jan. 11];92(S2):e2021428. | Wrong outcome |
| 180. Behrmann JT, Blaabjerg J, Jordansen J, Jensen de López KM. Systematic Review: Investigating the Impact of COVID-19 on Mental Health Outcomes of Individuals With ADHD. J Atten Disord. 2022 May;26(7):959-975. doi: 10.1177/10870547211050945. Epub 2021 Oct 15. PMID: 34654341. | Wrong outcome |
| 181. Berger E, Jamshidi N, Reupert A, Jobson L, Miko A. Review: The mental health implications for children and adolescents impacted by infectious outbreaks - a systematic review. Child Adolesc Ment Health. 2021 May;26(2):157-166. doi: 10.1111/camh.12453. Epub 2021 Mar 17. PMID: 33733620. | Wrong outcome |
| 182. Ciuffreda G, Cabanillas-Barea S, Carrasco-Uribarren A, Albarova-Corral MI, Argüello-Espinosa MI, Marcén-Román Y. Factors Associated with Depression and Anxiety in Adults ≥60 Years Old during the COVID-19 Pandemic: A Systematic Review. Int J Environ Res Public Health. 2021 Nov 12;18(22):11859. doi: 10.3390/ijerph182211859. PMID: 34831615; PMCID: PMC8621514. | Wrong outcome |
| 183. Dellazizzo L, Léveillé N, Landry C, Dumais A. Systematic Review on the Mental Health and Treatment Impacts of COVID-19 on Neurocognitive Disorders. J Pers Med. 2021 Jul 29;11(8):746. doi: 10.3390/jpm11080746. PMID: 34442390; PMCID: PMC8401453. | Wrong outcome |
| 184. Đurđević Sanja, Conde Ghigliazza Ileana, Dukanac Vesna et al.Anxiety and depressive symptomatology among children and adolescents exposed to the COVID-19 pandemic: A systematic review  PY - 2022 Vojnosanit Pregl 2022; 79(4): 389–399. | Wrong outcome |
| 185. Uzbay, T. (2021). "Effects of the Covid-19 pandemic on brain and behavior." Journal of Research in Pharmacy 25(6): 785-798. | Wrong outcome |
| 186. Wilkialis, L., et al. (2021). "Social Isolation, Loneliness and Generalized Anxiety: Implications and Associations during the COVID-19 Quarantine." BRAIN SCIENCES 11(12). | Wrong outcome |
| 187. Windarwati, H. D., et al. (2022). "A narrative review into the impact of covid‐19 pandemic on senior high school adolescent mental health." JOURNAL OF CHILD AND ADOLESCENT PSYCHIATRIC NURSING. | Wrong outcome |
| 188. Zhou, Y., et al. (2021). "The prevalence of PTSS under the influence of public health emergencies in last two decades: A systematic review and meta-analysis." Clinical Psychology Review 83 | Wrong outcome |
| 189. Salehiniya, H., et al. (2022). "Mental health status of dentists during COVID-19 pandemic: A systematic review and meta-analysis." Health Sci Rep 5(3): e617. | Wrong outcome |
| 190. Soltani, S., et al. (2021). "COVID-19 associated central nervous system manifestations, mental and neurological symptoms: A systematic review and meta-analysis." Reviews in the Neurosciences 32(3): 351-361. | Wrong outcome |
| 1. Alves, G.S., et al., *A Systematic Review of Home-Setting Psychoeducation Interventions for Behavioral Changes in Dementia: Some Lessons for the COVID-19 Pandemic and Post-Pandemic Assistance.* Frontiers in Psychiatry, 2020. 11. | Wrong Study Duration |
| 2. Amro, M., A. Mohamed, and M. Alawna, *Effects of increasing aerobic capacity on improving psychological problems seen in patients with COVID-19: A review.* European Review for Medical and Pharmacological Sciences, 2021. 25(6): p. 2808-2821. | Wrong Study Duration |
| 3. Barnett, P., et al., *Tele-mental health services: a rapid umbrella review of pre-COVID-19 literature.* Journal of Medical Internet Research, 2021. 23(7): p. N.PAG-N.PAG. | Wrong Study Duration |
| 4. Bennett, C.B., et al., *eHealth to redress psychotherapy access barriers both new and old: A review of reviews and meta-analyses.* Journal of Psychotherapy Integration, 2020. 30(2): p. 188-207. | Wrong Study Duration |
| 5. Brooks, S.K., D. Weston, and N. Greenberg, *Psychological impact of infectious disease outbreaks on pregnant women: rapid evidence review.* Public Health (Elsevier), 2020. 189: p. 26-36. | Wrong Study Duration |
| 6. Brooks, S.K., et al., *The psychological impact of quarantine and how to reduce it: rapid review of the evidence.* Lancet, 2020. 395(10227): p. 912-920. | Wrong Study Duration |
| 7. Cavicchioli, M., et al., *What Will Be the Impact of the Covid-19 Quarantine on Psychological Distress? Considerations Based on a Systematic Review of Pandemic Outbreaks.* Healthcare (Basel). 9(1). | Wrong Study Duration |
| 8. Chew, Q.H., et al., *Psychological and Coping Responses of Health Care Workers Toward Emerging Infectious Disease Outbreaks: A Rapid Review and Practical Implications for the COVID-19 Pandemic.* The Journal of clinical psychiatry, 2020. 81(6). | Wrong Study Duration |
| 9. Chigwedere, O.C., et al., *The impact of epidemics and pandemics on the mental health of healthcare workers: A systematic review.* International Journal of Environmental Research and Public Health, 2021. 18(13). | Wrong Study Duration |
| 10. Dahiya, A.V., et al., *A systematic review of technological approaches for autism spectrum disorder assessment in children: Implications for the COVID-19 pandemic.* Res Dev Disabil. 109: p. 103852. | Wrong Study Duration |
| 11. Dixit, S. and G. akumar, *Promoting healthy lifestyles using information technology during the COVID-19 pandemic.* Reviews in Cardiovascular Medicine, 2021. 22(1): p. 115-125. | Wrong Study Duration |
| 12. Fiest, K.M., et al., *Experiences and management of physician psychological symptoms during infectious disease outbreaks: A rapid review.* BMC Psychiatry, 2021. 21. | Wrong Study Duration |
| 13. Fischer, R., et al., *Rapid Review and Meta-Meta-Analysis of Self-Guided Interventions to Address Anxiety, Depression, and Stress During COVID-19 Social Distancing.* FRONTIERS IN PSYCHOLOGY. 11. | Wrong Study Duration |
| 14. Gagliardi, A.R., et al., *The psychological burden of waiting for procedures and patient‐centred strategies that could support the mental health of wait‐listed patients and caregivers during the COVID‐19 pandemic: A scoping review.* Health Expectations, 2021. 24(3): p. 978-990. | Wrong Study Duration |
| 15. Galli, F., et al., *A Systematic Review and Provisional Metanalysis on Psychopathologic Burden on Health Care Workers of Coronavirus Outbreaks.* Frontiers in Psychiatry, 2020. 11. | Wrong Study Duration |
| 16. Gomez-Duran, E.L., C. Martin-Fumado, and C.G. Forero, *Psychological impact of quarantine on healthcare workers.* OCCUPATIONAL AND ENVIRONMENTAL MEDICINE. 77(10): p. 666-674. | Wrong Study Duration |
| 17. Hooper, J.J., et al., *Addressing the psychological impact of COVID-19 on healthcare workers: Learning from a systematic review of early interventions for frontline responders.* BMJ Open, 2021. 11(5). | Wrong Study Duration |
| 18. Hossain, M.M., A. Sultana, and N. Purohit, *Mental health outcomes of quarantine and isolation for infection prevention: a systematic umbrella review of the global evidence.* Epidemiology & Health, 2020. 42: p. e2020038-e2020038. | Wrong Study Duration |
| 19. Jones, C., et al., *Virtual Trauma-Focused Therapy for Military Members, Veterans, and Public Safety Personnel With Posttraumatic Stress Injury: Systematic Scoping Review.* JMIR MHEALTH AND UHEALTH. 8(9). | Wrong Study Duration |
| 20. Kisely, S., et al., *Occurrence, prevention, and management of the psychological effects of emerging virus outbreaks on healthcare workers: rapid review and meta-analysis.* BMJ (Clinical research ed.), 2020. 369: p. m1642. | Wrong Study Duration |
| 21. Kuek, J.T.Y., et al., *The impact of caring for dying patients in intensive care units on a physician's personhood: a systematic scoping review.* Philos Ethics Humanit Med. 15(1): p. 12. | Wrong Study Duration |
| 22. Kulkarni, M.S., et al., *Combating the psychological impact of COVID-19 pandemic through yoga: Recommendation from an overview.* Journal of Ayurveda and Integrative Medicine, 2021. | Wrong Study Duration |
| 23. Laufs, J. and Z. Waseem, *Policing in pandemics: A systematic review and best practices for police response to COVID-19.* Int J Disaster Risk Reduct. 51: p. 101812. | Wrong Study Duration |
| 24. Lenferink, L.I.M., K. Meyerbröker, and P.A. Boelen, *PTSD treatment in times of COVID-19: A systematic review of the effects of online EMDR.* Psychiatry Research, 2020. 293. | Wrong Study Duration |
| 25. Liu, D., R.F. Baumeister, and Y. Zhou, *Mental health outcomes of coronavirus infection survivors: A rapid meta-analysis.* Journal of Psychiatric Research, 2021. 137: p. 542-553. | Wrong Study Duration |
| 26. Loades, M.E., et al., *Rapid Systematic Review: The Impact of Social Isolation and Loneliness on the Mental Health of Children and Adolescents in the Context of COVID-19.* Journal of the American Academy of Child & Adolescent Psychiatry, 2020. 59(11): p. 1218-1218. | Wrong Study Duration |
| 27. Luo, Y., et al., *A Systematic Review of the Impact of Viral Respiratory Epidemics on Mental Health: An Implication on the Coronavirus Disease 2019 Pandemic.* Frontiers in Psychiatry, 2020. 11. | Wrong Study Duration |
| 28. M, T. and A. Annamalai, *Telepsychiatry and the Role of Artificial Intelligence in Mental Health in Post-COVID-19 India: A Scoping Review on Opportunities.* Indian J Psychol Med. 42(5): p. 428-434. | Wrong Study Duration |
| 29. McGrath, M., et al., *Effectiveness of community interventions for protecting and promoting the mental health of working-age adults experiencing financial uncertainty: a systematic review.* J Epidemiol Community Health. 75(7): p. 665-73. | Wrong Study Duration |
| 30. Morina, N., et al., *Potential impact of physical distancing on physical and mental health: a rapid narrative umbrella review of meta-analyses on the link between social connection and health.* BMJ Open. 11(3): p. e042335. | Wrong Study Duration |
| 31. O'Donohue, K., et al., *Psychological outcomes for young adults after disastrous events: A mixed-methods scoping review.* Soc Sci Med. 276: p. 113851. | Wrong Study Duration |
| 32. Simonovich, S.D., et al., *Meta-Analysis Of Antenatal Depression And Adverse Birth Outcomes In US Populations, 2010-20.* Health Aff (Millwood). 40(10): p. 1560-1565. | Wrong Study Duration |
| 33. Toscano, M., et al., *Prevalence of Depression or Anxiety During Antepartum Hospitalizations for Obstetric Complications: A Systematic Review and Meta-analysis.* Obstet Gynecol. 137(5): p. 881-891. | Wrong Study Duration |
| 34. van der Kruk, S.R., et al., *Psychosocial well-being and supportive care needs of cancer patients and survivors living in rural or regional areas: a systematic review from 2010 to 2021.* Support Care Cancer: p. 1-44. | Wrong Study Duration |
| 35. Vasileva, M., et al., *Research review: A meta-analysis of the international prevalence and comorbidity of mental disorders in children between 1 and 7 years.* J Child Psychol Psychiatry. 62(4): p. 372-381. | Wrong Study Duration |
| 36. Henssler J, Stock F, van Bohemen J, Walter H, Heinz A, Brandt L. Mental health effects of infection containment strategies: quarantine and isolation-a systematic review and meta-analysis. Eur Arch Psychiatry Clin Neurosci. 2021 Mar;271(2):223-234. doi: 10.1007/s00406-020-01196-x. Epub 2020 Oct 6. | Wrong Study Duration |
| 37. Saragih, I. D., et al. (2021). "Global prevalence of mental health problems among healthcare workers during the Covid-19 pandemic: A systematic review and meta-analysis." INTERNATIONAL JOURNAL OF NURSING STUDIES 121: N.PAG-N.PAG. | Wrong Study Duration |
| 38. Zhou, Y., et al. (2021). "The prevalence of PTSS under the influence of public health emergencies in last two decades: A systematic review and meta-analysis." Clinical Psychology Review 83. | Wrong Study Duration |
| 39. Sousa, L., et al. (2021). "Psychological impact of COVID-19 on healthcare workers: prevalence systematic review." Acta Paulista de Enfermagem 34(4): 1-7. | Wrong Study Duration |
| 40. Zhang, L., et al. (2022). "The effect of the COVID-19 pandemic on health care workers' anxiety levels: a meta-analysis." PEERJ 10: e13225. | Wrong Study Duration |
| 41. Zürcher, S. J., et al. (2022). "Post-viral mental health sequelae in infected persons associated with COVID-19 and previous epidemics and pandemics: Systematic review and meta-analysis of prevalence estimates." J Infect Public Health 15(5): 599-608. | Wrong Study Duration |
| 1. Abila, S.S. and I.L. Acejo, *Mental health of Filipino seafarers and its implications for seafarers' education.* Int Marit Health, 2021. **72**(3): p. 183-192. | Wrong publication type |
| 2. Agarwal, V., L. Ganesh, and B.K. Sunitha, *Impact of COVID-19 on the mental health among children in China with specific reference to emotional and behavioral disorders.* INTERNATIONAL JOURNAL OF HUMAN RIGHTS IN HEALTH CARE. **14**(2): p. 182-188. | Wrong publication type |
| 3. Ahmad, M., et al., *Psychological impact on health workers of Covid-19 outbreak in the early emergency period: a brief and quick systematic review.* RIVISTA DI PSICOLOGIA DELL EMERGENZA E DELL ASSISTENZA UMANITARIA, 2021(25): p. 6-27. | Wrong publication type |
| 4. Ahmad, M. and L. Vismara, *The psychological impact of COVID-19 pandemic on women’s mental health during pregnancy: A rapid evidence review.* International Journal of Environmental Research and Public Health, 2021. **18**(13). | Wrong publication type |
| 5. Aiyegbusi, O.L., et al., *Symptoms, complications and management of long COVID: a review.* Journal of the Royal Society of Medicine, 2021. **114**(9): p. 428-442. | Wrong publication type |
| 6. Akbarialiabad, H., et al., *Long COVID, a comprehensive systematic scoping review.* INFECTION. | Wrong publication type |
| 7. Al Mamun, F., et al., *Mental Disorders of Bangladeshi Students During the COVID-19 Pandemic: A Systematic Review.* Psychol Res Behav Manag, 2021. **14**: p. 645-654. | Wrong publication type |
| 8. Amanullah, S. and R. Ramesh Shankar, *The Impact of COVID-19 on Physician Burnout Globally: A Review.* Healthcare (Basel). **8**(4). | Wrong publication type |
| 9. Ammouni, A. and J. Hayden, *The psychological impact of viral epidemics on medical students: A systematic review.* Irish Journal of Medical Science, 2021. **190**: p. S142-S143. | Wrong publication type |
| 10. Anaya, J.M., et al., *Post-COVID syndrome. A case series and comprehensive review.* Autoimmunity Reviews, 2021. | Wrong publication type |
| 11. Arifin, S. and N.A. Hassan, *The Psychological Impact of COVID-19 on Antenatal Women: A Scoping Review.* IIUM MEDICAL JOURNAL MALAYSIA. **19**(2): p. 9-+. | Wrong publication type |
| 12. Bachilo, E.V., *Mental health of population during the covid-19 pandemic.* Zhurnal Nevrologii i Psihiatrii imeni S.S. Korsakova, 2020. **120**(10): p. 130-136. | Wrong publication type |
| 13. Banerjee, D., et al., *Impact of the COVID-19 pandemic on psychosocial health and well-being in South-Asian (World Psychiatric Association zone 16) countries: A systematic and advocacy review from the Indian Psychiatric Society.* Indian J Psychiatry. **62**: p. S343-s353. | Wrong publication type |
| 14. Banerjee, D., H.G. Vijayakumar, and T.S.S. Rao, *"Watching the watchmen:" Mental health needs and solutions for the health-care workers during the coronavirus disease 2019 pandemic.* INTERNATIONAL JOURNAL OF HEALTH AND ALLIED SCIENCES. **9**: p. 51-54. | Wrong publication type |
| 15. Barros, K.C.C., et al., *Estresse ocupacional em ambiente hospitalar no cenário da COVID-19: revisão das estratégias de enfrentamento dos trabalhadores de enfermagem.* Enfermagem Brasil, 2021. **20**(3): p. 412-428. | Wrong publication type |
| 16. Bekele, F. and M. Hajure, *Magnitude and determinants of the psychological impact of COVID-19 among health care workers: A systematic review.* SAGE Open Medicine, 2021. **9**. | Wrong publication type |
| 17. Bekele, F., D.F. Mechessa, and B. Sefera, *Prevalence and associated factors of the psychological impact of COVID-19 among communities, health care workers and patients in Ethiopia: A systematic review.* Annals of Medicine and Surgery, 2021. **66**. | Wrong publication type |
| 18. Benfante, A., et al., *Traumatic Stress in Healthcare Workers During COVID-19 Pandemic: A Review of the Immediate Impact.* Front Psychol, 2020. **11**: p. 569935. | Wrong publication type |
| 19. Bohlken, J., et al., *COVID-19 Pandemic: Stress Experience of Healthcare Workers: A Short Current Review.* Psychiatrische Praxis, 2020. **47**(4): p. 190-197. | Wrong publication type |
| 20. Boulkrane, M.S., et al., *The impact of SARS-Cov-2 on the Nervous system and Mental Health.* Curr Neuropharmacol. | Wrong publication type |
| 21. Cabrera, M.A., L. Karamsetty, and S.A. Simpson, *Coronavirus and Its Implications for Psychiatry: A Rapid Review of the Early Literature.* Psychosomatics, 2020. **61**(6): p. 607-615. | Wrong publication type |
| 22. Chamaa, F., et al., *PTSD in the COVID-19 Era.* Current neuropharmacology, 2021. | Wrong publication type |
| 23. Chawla, N., et al., *Psychological Impact of COVID-19 on Children and Adolescents: A Systematic Review.* Indian J Psychol Med. **43**(4): p. 294-299. | Wrong publication type |
| 24. Clemente-Suárez, V.J., et al., *The impact of the covid-19 pandemic on mental disorders. A critical review.* International Journal of Environmental Research and Public Health, 2021. **18**(19). | Wrong publication type |
| 25. Coombes, A., et al., *Prevalence of anxiety, depression, post-traumatic stress, and burnout in healthcare workers during the covid-19 pandemic: A systematic review and meta-analysis.* Irish Journal of Medical Science, 2021. **190**: p. S145. | Wrong publication type |
| 26. Cuadra-Martínez, D., et al., *[COVID-19 and psychological behavior: a systematic review of the psychological effects of 21st century pandemics].* Rev Med Chil. **148**(8): p. 1139-1154. | Wrong publication type |
| 27. Cunning, C. and M. Hodes, *The COVID-19 pandemic and obsessive-compulsive disorder in young people: Systematic review.* CLINICAL CHILD PSYCHOLOGY AND PSYCHIATRY. | Wrong publication type |
| 28. D'Silva, K.M. and Z.S. Wallace, *COVID-19 and rheumatoid arthritis.* Current Opinion in Rheumatology, 2021. **33**(3): p. 255-261. | Wrong publication type |
| 29. da Silva, F.C.T. and C.P. Barbosa, *The impact of the COVID-19 pandemic in an intensive care unit (ICU): Psychiatric symptoms in healthcare professionals.* Progress in Neuro-Psychopharmacology and Biological Psychiatry, 2021. **110**. | Wrong publication type |
| 30. da Silva, F.C.T. and M.L.R. Neto, *Psychiatric disorders in health professionals during the COVID-19 pandemic: A systematic review with meta-analysis.* JOURNAL OF PSYCHIATRIC RESEARCH. **140**: p. 474-487. | Wrong publication type |
| 31. Danet Danet, A., *Psychological impact of COVID-19 pandemic in Western frontline healthcare professionals. A systematic review.* Medicina Clinica, 2021. **156**(9): p. 449-458. | Wrong publication type |
| 32. De Kock, J.H., et al., *A rapid review of the impact of COVID-19 on the mental health of healthcare workers: implications for supporting psychological well-being.* BMC Public Health. **21**(1): p. 104. | Wrong publication type |
| 33. de Macêdo Rocha, D., et al., *Psychosocial effects of social distancing during coronavirus infections: integrative review.* Acta Paulista de Enfermagem, 2021. **34**(1): p. 1-9. | Wrong publication type |
| 34. de Macedo Rocha, K.P., et al., *Vivências de luto e saúde mental da enfermagem na pandemia da COVID-19: o que nos diz a literatura?* Saude Coletiva, 2021. **11**(62): p. 5092-5096. | Wrong publication type |
| 35. de Oliveira, W.A., et al., *Adolescents' health in times of COVID-19: a scoping review.* CADERNOS DE SAUDE PUBLICA, 2020. **36**(8). | Wrong publication type |
| 36. Della Monica, A., et al., *The impact of Covid-19 healthcare emergency on the psychological well-being of health professionals: a review of literature.* Annali di igiene : medicina preventiva e di comunita, 2021. | Wrong publication type |
| 37. D’ettorre, G., et al., *Post-traumatic stress symptoms in healthcare workers dealing with the covid-19 pandemic: A systematic review.* International Journal of Environmental Research and Public Health, 2021. **18**(2): p. 1-16. | Wrong publication type |
| 38. Fern, et al., *Anxiety and depression among healthcare workers during the COVID-19 pandemic: a systematic umbrella review of the global evidence.* BMJ Open. **11**(9): p. e054528. | Wrong publication type |
| 39. Garcia-Iglesias, J.J., et al., *Impact of SARS-CoV-2 (Covid-19) on the mental health of healthcare professionals: a systematic review.* REVISTA ESPANOLA DE SALUD PUBLICA. **94**. | Wrong publication type |
| 40. Giorgi, G., et al., *COVID-19-related mental health effects in the workplace: A narrative review.* International Journal of Environmental Research and Public Health, 2020. **17**(21): p. 1-22. | Wrong publication type |
| 41. Gualano, M.R., et al., *The burden of burnout among healthcare professionals of intensive care units and emergency departments during the covid-19 pandemic: A systematic review.* International Journal of Environmental Research and Public Health, 2021. **18**(15). | Wrong publication type |
| 42. Haan, R., et al., *Health and Well-Being of Athletes During the Coronavirus Pandemic: A Scoping Review.* FRONTIERS IN PUBLIC HEALTH. **9**. | Wrong publication type |
| 43. Hekmat, A., R. Divanbeigi, and S.A.F. Yegane, *Effects of COVID-19 on student’s mental health: A systematic review.* Pakistan Journal of Medical and Health Sciences, 2021. **15**(4): p. 1543-1550. | Wrong publication type |
| 44. Hong, S., H. Kim, and M.K. Park, *Impact of COVID‐19 on post‐traumatic stress symptoms in the general population: An integrative review.* International Journal of Mental Health Nursing, 2021. **30**(4): p. 834-846. | Wrong publication type |
| 45. Huarcaya-Victoria, J., *Mental health considerations about the COVID-19 pandemic.* Revista Peruana de Medicina Experimental y Salud Publica, 2020. **37**(2): p. 327-334. | Wrong publication type |
| 46. Iyengar, U., et al., *One Year Into the Pandemic: A Systematic Review of Perinatal Mental Health Outcomes During COVID-19.* Frontiers in Psychiatry, 2021. **12**. | Wrong publication type |
| 47. Jephtha, M.C. and M. Jagadeesan, *Insomnia, depression and anxiety among healthcare workers during covid-19 pandemic-an evidencebased review.* International Journal of Pharmaceutical Research, 2021. **13**(2): p. 605-612. | Wrong publication type |
| 48. Johnson, L., et al., *Scoping review of mental health in prisons through the COVID-19 pandemic.* BMJ Open, 2021. **11**(5). | Wrong publication type |
| 49. Jones, E.A.K., A.K. Mitra, and A.R. Bhuiyan, *Impact of covid-19 on mental health in adolescents: A systematic review.* International Journal of Environmental Research and Public Health, 2021. **18**(5): p. 1-9. | Wrong publication type |
| 50. Jordan, V., *Coronavirus (COVID-19): implementation facilitators to supporting programmes for improving the resilience and mental health of frontline workers during and after an epidemic.* Journal of Primary Health Care, 2020. **12**(4): p. 395-396. | Wrong publication type |
| 51. Jothishanmugam, A., et al., *Psychological impact of covid-19 outbreak on nurses: A systematic review.* International Journal of Research in Pharmaceutical Sciences, 2020. **11**: p. 1905-1913. | Wrong publication type |
| 52. Junaid, K., H. Ali, and R. Nazim, *Depression Among Healthcare Workers During the COVID-19 Pandemic in Low and Middle-Income Countries: A Systematic Review.* ANNALS OF KING EDWARD MEDICAL UNIVERSITY LAHORE PAKISTAN, 2020. **26**: p. 252-258. | Wrong publication type |
| 53. Kan, F.P., et al., *A systematic review of the prevalence of anxiety among the general population during the COVID-19 pandemic.* JOURNAL OF AFFECTIVE DISORDERS. **293**: p. 391-398. | Wrong publication type |
| 54. Kang, M.J. and J.H. Park, *The effects of COVID-19 on physician's burnout: a systematic review.* JOURNAL OF THE KOREAN MEDICAL ASSOCIATION. **64**(9): p. 636-646. | Wrong publication type |
| 55. Keskin, S. and B. Özkan, *Mental statuses of nursing students in the covid-19 pandemic period: A systematic review.* Annals of Clinical and Analytical Medicine, 2021. **12**: p. 245-249. | Wrong publication type |
| 56. Khaffaf, E.S. and Z.T. Madalah, *The relation between mental health and covid-19 pandemic: A systematic review study.* Pakistan Journal of Medical and Health Sciences, 2021. **15**(4): p. 1155-1157. | Wrong publication type |
| 57. Khan, M.A., *COVID-19's Impact on Higher Education: A Rapid Review of Early Reactive Literature.* EDUCATION SCIENCES. **11**(8). | Wrong publication type |
| 58. Khanizadeh, T., V. Omranifard, and S. Khanizadeh, *The outcomes of COVID-19 epidemic crisis on the elderly: A review study.* Journal of Isfahan Medical School, 2021. **39**(622): p. 292-302. | Wrong publication type |
| 59. Khoundabi, B., A. Soltani, and M.A. Marzaleh, *Psychological problems of the nursing staff in covid-19 pandemic: A systematic review.* Iranian Red Crescent Medical Journal, 2021. **23**(6). | Wrong publication type |
| 60. Killikelly, C., et al., *Rapid Systematic Review of Psychological Symptoms in Health Care Workers COVID-19.* JOURNAL OF LOSS & TRAUMA. **26**(7): p. 638-655. | Wrong publication type |
| 61. Kunz, M., M. Strasser, and A. Hasan, *Impact of the coronavirus disease 2019 pandemic on healthcare workers: systematic comparison between nurses and medical doctors.* Current Opinion in Psychiatry, 2021. **34**(4): p. 413-419. | Wrong publication type |
| 62. Lakhan, R., A. Agrawal, and M. Sharma, *Prevalence of Depression, Anxiety, and Stress during COVID-19 Pandemic.* Journal of Neurosciences in Rural Practice, 2020. **11**(4): p. 519-525. | Wrong publication type |
| 63. Latuapo, A., M. Farid, and Z. Ab Rahman, *Pharmaceutical and nonpharmaceutical use of music and al-quran therapy in preventing the spread of pandemics (covid-19): A systematic review.* Systematic Reviews in Pharmacy, 2020. **11**(12): p. 1171-1179. | Wrong publication type |
| 64. Lee, V., et al., *The impact of COVID-19 on the mental health and wellbeing of caregivers of autistic children and youth: A scoping review.* Autism Res. | Wrong publication type |
| 65. Lestari, R. and F.E.B. Setyawan, *Mental health policy: protecting community mental health during the COVID-19 pandemic.* JOURNAL OF PUBLIC HEALTH RESEARCH, 2021. **10**(2). | Wrong publication type |
| 66. Lin, C.Y. and Y.L. Lin, *Anxiety and depression of general population in the early phase of COVID-19 pandemic: A systematic review of cross-sectional studies.* Revista de Psiquiatria Clinica, 2020. **47**(6): p. 199-208. | Wrong publication type |
| 67. Lin, Q. and Y. Zheng, *Nurses' Emotional Stress Levels When Caring for COVID-19 Patients in an Intensive Care Unit.* Alternative therapies in health and medicine, 2021. | Wrong publication type |
| 68. Liu, W.Q.H., H.T. Zhang, and Y. He, *Variation in Obsessive-Compulsive Disorder Symptoms and Treatments: A Side Effect of COVID-19.* INTERNATIONAL JOURNAL OF ENVIRONMENTAL RESEARCH AND PUBLIC HEALTH. **18**(14). | Wrong publication type |
| 69. Luo, X., et al., *The psychological impact of quarantine on coronavirus disease 2019 (COVID-19).* Psychiatry Research, 2020. **291**. | Wrong publication type |
| 70. Lupieri, S., *Refugee health during the covid-19 pandemic: A review of global policy responses.* Risk Management and Healthcare Policy, 2021. **14**: p. 1373-1378. | Wrong publication type |
| 71. Makara-Studzinska, M., M. Zaluski, and J. Lickiewicz, *Are the COVID-19 survivors future patients of psychiatrists and psychologists A rapid literature review.* Psychiatria, 2021. **18**(2): p. 140-151. | Wrong publication type |
| 72. Malini and Amarendiran, *Systamatic investigation of mental stress during covid-19.* European Journal of Molecular and Clinical Medicine, 2020. **7**(7): p. 5542-5550. | Wrong publication type |
| 73. Manca, R., M. De Marco, and A. Venneri, *The Impact of COVID-19 Infection and Enforced Prolonged Social Isolation on Neuropsychiatric Symptoms in Older Adults With and Without Dementia: A Review.* Frontiers in Psychiatry, 2020. **11**. | Wrong publication type |
| 74. Marchi, J., et al., *The Impact of the COVID-19 Pandemic and Societal Infection Control Measures on Children and Adolescents' Mental Health: A Scoping Review.* Frontiers in Psychiatry, 2021. **12**. | Wrong publication type |
| 75. Meherali, S., et al., *Mental Health of Children and Adolescents Amidst COVID-19 and Past Pandemics: A Rapid Systematic Review.* Int J Environ Res Public Health. **18**(7). | Wrong publication type |
| 76. Mehraeen, E., et al., *COVID-19 in pediatrics: The current knowledge and practice.* Infect Disord Drug Targets. | Wrong publication type |
| 77. Meloni, S., G. de Girolamo, and R. Rossi, *[COVID-19 and mental health services in Europe].* Epidemiol Prev. **44**(5): p. 383-393. | Wrong publication type |
| 78. Mihai, E.C., *Mood disorders in children due to COVID-19 pandemic.* JOURNAL OF EDUCATIONAL SCIENCES & PSYCHOLOGY, 2020. **10**(2): p. 147-152. | Wrong publication type |
| 79. Minozzi, S., et al., *[Impact of social distancing for covid-19 on young people: type and quality of the studies found through a systematic review of the literature.].* Recenti Prog Med. **112**(5): p. 51e-67e. | Wrong publication type |
| 80. Minozzi, S., et al., *Impact of social distancing for covid-19 on the psychological well-being of youths: A systematic review of the literature.* Recenti Progressi in Medicina, 2021. **112**(5): p. 360-370. | Wrong publication type |
| 81. Mir, M.d., et al., *How is COVID-19 pandemic impacting mental health of children and adolescents?* Int J Disaster Risk Reduct. **51**: p. 101845. | Wrong publication type |
| 82. Mobasseri, K., et al., *The Main Issues and Challenges Older Adults Face in the SARS-CoV-2 Pandemic: A Scoping Review of Literature.* Iran J Public Health. **49**(12): p. 2295-2307. | Wrong publication type |
| 83. Moitra, M., et al., *Mental Health Consequences for Healthcare Workers During the COVID-19 Pandemic: A Scoping Review to Draw Lessons for LMICs.* Frontiers in Psychiatry, 2021. **12**. | Wrong publication type |
| 84. Mol, S. and S. Monisha, *Systamatic review on depression during covid-19.* European Journal of Molecular and Clinical Medicine, 2020. **7**(7): p. 5551-5560. | Wrong publication type |
| 85. Moreira, J.L.D., et al., *The psychiatric and neuropsychiatric repercussions associated with severe infections of COVID-19 and other coronaviruses.* PROGRESS IN NEURO-PSYCHOPHARMACOLOGY & BIOLOGICAL PSYCHIATRY. **106**. | Wrong publication type |
| 86. Mousavizadeh, S.N., P.G. Merdasi, and M. Safari, *Psychological Challenges of Nurses in Pandemic Covid-19.* PAKISTAN JOURNAL OF MEDICAL & HEALTH SCIENCES. **15**(1): p. 448-455. | Wrong publication type |
| 87. Muehlschlegel, P.A., et al., *Learning from previous lockdown measures and minimising harmful biopsychosocial consequences as they end: A systematic review.* Journal of global health, 2021. **11**: p. 05008. | Wrong publication type |
| 88. Mukhtar, S., *Psychological health during the coronavirus disease 2019 pandemic outbreak.* International Journal of Social Psychiatry, 2020. **66**(5): p. 512-516. | Wrong publication type |
| 89. Mukona, D.M. and M. Zvinavashe, *Diabetes mellitus and depression amid the COVID-19 pandemic: Possible solutions for resource limited settings.* AFRICAN JOURNAL OF DIABETES MEDICINE. **28**(1). | Wrong publication type |
| 90. Mulfinger, N., et al., *Psychological stress caused by epidemics among health care workers and implications for coping with the corona crisis: a literature review.* ZEITSCHRIFT FUR PSYCHOSOMATISCHE MEDIZIN UND PSYCHOTHERAPIE, 2020. **66**(3): p. 220-242. | Wrong publication type |
| 91. Muller, A.E., et al., *The mental health impact of the covid-19 pandemic on healthcare workers, and interventions to help them: A rapid systematic review.* PSYCHIATRY RESEARCH. **293**. | Wrong publication type |
| 92. Munich, J., et al., *Impact of Pandemics/Epidemics on Emergency Department Utilization for Mental Health and Substance Use: A Rapid Review.* Frontiers in Psychiatry, 2021. **12**. | Wrong publication type |
| 93. Murphy, J.K., et al., *Needs, gaps and opportunities for standard and e-mental health care among at-risk populations in the Asia Pacific in the context of COVID-19: a rapid scoping review.* INTERNATIONAL JOURNAL FOR EQUITY IN HEALTH. **20**(1). | Wrong publication type |
| 94. Nassar, M., et al., *Current systematic reviews and meta-analyses of COVID-19.* World J Virol. **10**(4): p. 182-208. | Wrong publication type |
| 95. Nearchou, F., et al., *Exploring the Impact of COVID-19 on Mental Health Outcomes in Children and Adolescents: A Systematic Review.* Int J Environ Res Public Health. **17**(22). | Wrong publication type |
| 96. Nepal, S., et al., *Impact of covid-19 on mental dimension of health: A sensitive issue to be addressed at the earliest.* Current Psychiatry Research and Reviews, 2020. **16**(3): p. 158-166. | Wrong publication type |
| 97. Neubeck, L., et al., *Delivering healthcare remotely to cardiovascular patients during COVID-19: A rapid review of the evidence.* European Journal of Cardiovascular Nursing, 2020. **19**(6): p. 486-494. | Wrong publication type |
| 98. O'Reilly, A., et al., *A rapid review investigating the potential impact of a pandemic on the mental health of young people aged 12–25 years.* Irish Journal of Psychological Medicine, 2020. | Wrong publication type |
| 99. Oakman, J., et al., *A rapid review of mental and physical health effects of working at home: how do we optimise health?* BMC Public Health, 2020. **20**(1): p. 1-13. | Wrong publication type |
| 100. Okuyama, J., et al., *Mental health and physical activity among children and adolescents during the COVID-19 pandemic.* Tohoku Journal of Experimental Medicine, 2021. **253**(3): p. 203-215. | Wrong publication type |
| 101. Oliveira, W.A., et al., *Adolescents' health in times of COVID-19: a scoping review.* Cad Saude Publica, 2020. **36**(8): p. e00150020. | Wrong publication type |
| 102. Oliveira, W., et al., *Impactos psicológicos e ocupacionais das sucessivas ondas recentes de pandemias em profissionais da saúde: Revisão integrativa e lições aprendidas = Psychological and occupational impacts of the recent successive pandemic waves on health workers: An integrative review and lessons learned.* Estudos de Psicologia, 2020. **37**. | Wrong publication type |
| 103. Pacheco, F., et al., *Breastfeeding during COVID-19: A Narrative Review of the Psychological Impact on Mothers.* BEHAVIORAL SCIENCES. **11**(3). | Wrong publication type |
| 104. Panchal, U., et al., *The impact of COVID-19 lockdown on child and adolescent mental health: systematic review.* EUROPEAN CHILD & ADOLESCENT PSYCHIATRY. | Wrong publication type |
| 105. Pappa, S., et al., *Corrigendum to "Prevalence of depression, anxiety, and insomnia among healthcare workers during the COVID-19 pandemic: A systematic review and meta-analysis" [Brain Behav. Immun. 88 (2020) 901-907].* Brain Behav Immun. **92**: p. 247. | Wrong publication type |
| 106. Parlapani, E., et al., *A review on the COVID-19-related psychological impact on older adults: vulnerable or not?* Aging Clinical & Experimental Research, 2021. **33**(6): p. 1729-1743. | Wrong publication type |
| 107. Pashazadeh Kan, F., et al., *A systematic review of the prevalence of anxiety among the general population during the COVID-19 pandemic.* Journal of Affective Disorders, 2021. **293**: p. 391-398. | Wrong publication type |
| 108. Pavani, F.M., et al., *Covid-19 and repercussions in mental health: a narrative review of literature.* Revista gaucha de enfermagem, 2021. **42**: p. e20200188. | Wrong publication type |
| 109. Perez-Rodriguez, P., et al., *Impact of Social Isolation due to COVID-19 in older people: Mental and physical effects and recommendations.* European Geriatric Medicine, 2020. **11**: p. S47. | Wrong publication type |
| 110. Petrova, N.N. and D.A. Khvostikova, *Prevalence, structure, and risk factors for mental disorders in older adults.* Advances in gerontology = Uspekhi gerontologii, 2021. **34**(1): p. 152-159. | Wrong publication type |
| 111. Philip, J. and V. Cherian, *Factors Affecting the Psychological Well-being of Health Care Workers During an Epidemic: A Thematic Review.* Indian J Psychol Med. **42**(4): p. 323-333. | Wrong publication type |
| 112. Pimenta, I.D.S.F., et al., *Media and scientific communication about the COVID-19 pandemic and the repercussions on the population's mental health: A protocol for a systematic review and meta-analysis.* Medicine, 2020. **99**(50): p. e23298. | Wrong publication type |
| 113. Pinho, L.G., et al., *Patient-Centered Care for People with Depression and Anxiety: An Integrative Review Protocol.* J Pers Med. **11**(5). | Wrong publication type |
| 114. Poletti, B., et al., *Telepsychotherapy: a leaflet for psychotherapists in the age of COVID-19. A review of the evidence.* COUNSELLING PSYCHOLOGY QUARTERLY. | Wrong publication type |
| 115. Pollock, A., et al., *Interventions to support the resilience and mental health of frontline health and social care professionals during and after a disease outbreak, epidemic or pandemic: a mixed methods systematic review.* Cochrane Database of Systematic Reviews, 2020(11). | Wrong publication type |
| 116. Pourdehghan, P., is, and S.-A. Mostafavi, *The Most Psychological Impacts of Coronavirus Epidemics: A Protocol for Systematic Review and Meta-Analysis.* Iranian Journal of Psychiatry, 2020. **15**(3): p. 248-251. | Wrong publication type |
| 117. Preti, E., et al., *The Psychological Impact of Epidemic and Pandemic Outbreaks on Healthcare Workers: Rapid Review of the Evidence.* Current Psychiatry Reports, 2020. **22**(8): p. 1-22. | Wrong publication type |
| 118. Priyantini, D., N. Nursalam, and T. Sukartini, *The first psychological management intervention towards community resilience facing covid-19 infection.* International Journal of Pharmaceutical Research, 2021. **13**(1): p. 4485-4492. | Wrong publication type |
| 119. Purnama, A., et al., *Mental health in health students during coronavirus disease-19: Systematic review.* Open Access Macedonian Journal of Medical Sciences, 2021. **9**: p. 205-210. | Wrong publication type |
| 120. Qi, Z., et al., *The impact of COVID-19 on sexual behaviors of young women and men: A protocol for systematic review and meta analysis.* Medicine, 2021. **100**(8): p. 1-5. | Wrong publication type |
| 121. Qian, Y., et al., *Influence of life intervention on anxiety, depression, and quality of life of COVID-19 patients: A protocol for systematic review and meta-analysis.* Medicine (Baltimore). **100**(18): p. e25391. | Wrong publication type |
| 122. Qiu, D., et al., *Policies to Improve the Mental Health of People Influenced by COVID-19 in China: A Scoping Review.* FRONTIERS IN PSYCHIATRY. **11**. | Wrong publication type |
| 123. Qiu, G.R., et al., *The effects of exercise therapy on the prognosis of patients with COVID-19 A protocol for systematic review.* MEDICINE. **99**(51). | Wrong publication type |
| 124. Racine, N., et al., *Child and adolescent mental illness during COVID-19: A rapid review.* Psychiatry Research, 2020. **292**. | Wrong publication type |
| 125. Radfar, A., et al., *Overcoming the Challenges of the Mental Health Care System in United States in the Aftermath of COVID-19.* CNS Spectrums: The International Journal of Neuropsychiatric Medicine, 2021. **26**(2): p. 176-176. | Wrong publication type |
| 126. Rahimi, R., et al., *A Systematic Review of the Prevalence of Mental Health Disorders in Pregnant Women during the COVID-19 Pandemic.* INTERNATIONAL JOURNAL OF PEDIATRICS-MASHHAD. **8**(11): p. 12397-12407. | Wrong publication type |
| 127. Rahman, A.A., et al., *Healthcare Workers Issues and COVID-19 Pandemic: A Review.* GAZI MEDICAL JOURNAL, 2020. **31**(2): p. 303-308. | Wrong publication type |
| 128. Raihan, M.M.H., *Mental health consequences of COVID-19 pandemic on adult population: a systematic review.* Mental Health Review Journal, 2021. **26**(1): p. 42-54. | Wrong publication type |
| 129. Rains, L.S., et al., *Early impacts of the COVID-19 pandemic on mental health care and on people with mental health conditions: framework synthesis of international experiences and responses.* SOCIAL PSYCHIATRY AND PSYCHIATRIC EPIDEMIOLOGY. **56**(1): p. 13-24. | Wrong publication type |
| 130. Rajkumar, R.P., *COVID-19 and mental health: A review of the existing literature.* Asian Journal of Psychiatry, 2020. **52**. | Wrong publication type |
| 131. Rajmil, L., et al., *Impact of lockdown and school closure on children's health and well-being during the first wave of COVID-19: a narrative review.* BMJ PAEDIATRICS OPEN. **5**(1). | Wrong publication type |
| 132. Rao, S., et al., *Psychosocial aspects of COVID-19 in the context of palliative care – A quick review.* Indian Journal of Palliative Care, 2020. **26**: p. 116-120. | Wrong publication type |
| 133. Raphael, J., R. Winter, and K. Berry, *Adapting practice in mental healthcare settings during the COVID-19 pandemic and other contagions: Systematic review.* BJPsych Open, 2021. **7**(2). | Wrong publication type |
| 134. Renjun, G., et al., *Psychological intervention on COVID-19: A protocol for systematic review and meta-analysis.* Medicine, 2020. **99**(21): p. e20335. | Wrong publication type |
| 135. Restauri, N. and A.D. Sheridan, *Burnout and Posttraumatic Stress Disorder in the Coronavirus Disease 2019 (COVID-19) Pandemic: Intersection, Impact, and Interventions.* Journal of the American College of Radiology, 2020. **17**(7): p. 921-926. | Wrong publication type |
| 136. Riedel-Heller, S. and D. Richter, *COVID-19-Pandemie trifft auf Psyche der Bevölkerung: Gibt es einen Tsunami psychischer Störungen? = COVID-19 pandemic and mental health of the general public: Is there a Tsunami of mental disorders?* Psychiatrische Praxis, 2020. **47**(8): p. 452-456. | Wrong publication type |
| 137. Robertson, L.J., et al., *Mental health of healthcare workers during the COVID-19 outbreak: A rapid scoping review to inform provincial guidelines in South Africa.* South African Medical Journal, 2020. **110**(10): p. 1010-1019. | Wrong publication type |
| 138. Rodrigues da Silva, R., et al., *Efeitos do isolamento social na pandemia da covid-19 na saúde mental da população.* Avances en Enfermeria, 2021. **39**: p. 1-13. | Wrong publication type |
| 139. Rodriguez, T.R., et al., *Protocol of psychological intervention facing COVID-19 in healthcare centers. Cienfuegos. Cuba.* MEDISUR-REVISTA DE CIENCIAS MEDICAS DE CIENFUEGOS. **18**(3): p. 368-380. | Wrong publication type |
| 140. Rodríguez-Fernández, P., et al., *Psychological Effects of Home Confinement and Social Distancing Derived from COVID-19 in the General Population-A Systematic Review.* INTERNATIONAL JOURNAL OF ENVIRONMENTAL RESEARCH AND PUBLIC HEALTH. **18**(12). | Wrong publication type |
| 141. Rohr, S., et al., *Psychosocial Impact of Quarantine Measures During Serious Coronavirus Outbreaks: A Rapid Review.* PSYCHIATRISCHE PRAXIS. **47**(4): p. 179-189. | Wrong publication type |
| 142. Russo, B. and M. Iudici, *Interventions for healthcare professionals caring for COVID-19 patients (beyond vaccines): A systematic review.* Infection Control and Hospital Epidemiology, 2021. **42**(5): p. 634-635. | Wrong publication type |
| 143. Ruta, F., et al., *[Covid-19 and front-line nurses' mental health: a literature review].* Prof Inferm. **74**(1): p. 41-47. | Wrong publication type |
| 144. Sahebi, A., et al., *The prevalence of anxiety and depression among healthcare workers during the COVID-19 pandemic: An umbrella review of meta-analyses.* Progress in Neuro-Psychopharmacology and Biological Psychiatry, 2021. **107**. | Wrong publication type |
| 145. Sahoo, K.C., et al., *Challenges in Maternal and Child Health Services Delivery and Access during Pandemics or Public Health Disasters in Low-and Middle-Income Countries: A Systematic Review.* Healthcare (Basel). **9**(7). | Wrong publication type |
| 146. Saint, S.A. and D.A. Moscovitch, *Effects of mask-wearing on social anxiety: An exploratory review.* Anxiety, Stress & Coping: An International Journal, 2021. | Wrong publication type |
| 147. Salmanian, M., M. Salehi, and Z. Hooshyari, *Global Prevalence of Posttraumatic Stress Disorder (PTSD) during and after Coronavirus Pandemic: A Study Protocol for a Systematic Review and Meta-Analysis.* Iranian Journal of Psychiatry, 2020. **15**(3): p. 252-255. | Wrong publication type |
| 148. Samantaray, N.N., et al., *Psychological management of mental health concerns related to COVID-19: A review of guidelines and recommendations.* Ind Psychiatry J. **29**(1): p. 12-21. | Wrong publication type |
| 149. Samji, H., et al., *Review: Mental health impacts of the COVID-19 pandemic on children and youth - a systematic review.* CHILD AND ADOLESCENT MENTAL HEALTH. | Wrong publication type |
| 150. Sanghera, J., et al., *The impact of SARS-CoV-2 on the mental health of healthcare workers in a hospital setting-A Systematic Review.* Journal of occupational health, 2020. **62**(1): p. e12175. | Wrong publication type |
| 151. Sasseville, M., et al., *Digital health interventions for the management of mental health in people with chronic diseases: a rapid review.* BMJ OPEN, 2021. **11**(4). | Wrong publication type |
| 152. Schneider, J., et al., *Factors mediating the psychological well-being of healthcare workers responding to global pandemics: A systematic review.* JOURNAL OF HEALTH PSYCHOLOGY. | Wrong publication type |
| 153. Seaborn, K., M. Chignell, and J. Gwizdka, *Psychological resilience during COVID-19: a meta-review protocol.* BMJ Open. **11**(6): p. e051417. | Wrong publication type |
| 154. Selick, A., et al., *Virtual health care for adult patients with intellectual and developmental disabilities: A scoping review.* Disabil Health J. **14**(4): p. 101132. | Wrong publication type |
| 155. Sergeant, A., et al., *Impact of COVID-19 and other pandemics and epidemics on people with pre-existing mental disorders: A systematic review protocol and suggestions for clinical care.* BMJ Open, 2020. **10**(9). | Wrong publication type |
| 156. SeyedAlinaghi, S., et al., *Psychological Symptoms of COVID-19 Epidemic: A Systematic Review of Current Evidence.* PSIHOLOGIJA, 2021. **54**(2): p. 173-192. | Wrong publication type |
| 157. Shanbehzadeh, S., et al., *Physical and mental health complications post-COVID-19: Scoping review.* Journal of Psychosomatic Research, 2021. **147**: p. N.PAG-N.PAG. | Wrong publication type |
| 158. Sharifi, M., A.A. Asadi-Pooya, and R.S. Mousavi-Roknabadi, *Burnout among Healthcare Providers of COVID-19; a Systematic Review of Epidemiology and Recommendations.* Archives of Academic Emergency Medicine, 2020. **9**(1): p. 1-17. | Wrong publication type |
| 159. Shaukat, N., D.M. Ali, and J. Razzak, *Physical and mental health impacts of COVID-19 on healthcare workers: a scoping review.* International Journal of Emergency Medicine, 2020. **13**(1): p. 1-8. | Wrong publication type |
| 160. Shelef, L., M. Schiff, and G. Zalsman, *[THE PSYCHOLOGICAL IMPACT OF A PANDEMIC OUTBREAK ON MEDICAL STAFF - LITERATURE REVIEW].* Harefuah. **159**(5): p. 326-331. | Wrong publication type |
| 161. Shelef, L. and G. Zalsman, *[THE PSYCHOLOGICAL IMPACT OF COVID-19 ON MENTAL HEALTH - LITERATURE REVIEW].* Harefuah. **159**(5): p. 320-325. | Wrong publication type |
| 162. Shi, J., et al., *Prevalence of delirium, depression, anxiety, and post-traumatic stress disorder among COVID-19 patients: protocol for a living systematic review.* Systematic Reviews, 2020. **9**(1). | Wrong publication type |
| 163. Shorey, S., et al., *Families With Children With Neurodevelopmental Disorders During COVID-19: A Scoping Review.* J Pediatr Psychol. **46**(5): p. 514-525. | Wrong publication type |
| 164. Shreffler, J., M. Huecker, and J. Petrey, *The Impact of COVID-19 on Healthcare Worker Wellness: A Scoping Review.* WESTERN JOURNAL OF EMERGENCY MEDICINE. **21**(5): p. 1059-1066. | Wrong publication type |
| 165. Shukla, J. and R. Manohar Singh, *Psychological Health amidst COVID-19: A Review of existing literature in the Indian Context.* Clinical Epidemiology and Global Health, 2021. **11**. | Wrong publication type |
| 166. Silva Junior, F., et al., *Suicidal behaviour in adults during the COVID-19 pandemic: protocol for systematic review of observational studies.* BMJ Open. **11**(8): p. e045313. | Wrong publication type |
| 167. Silva Junior, F., et al., *Impact of COVID-19 pandemic on mental health of young people and adults: a systematic review protocol of observational studies.* BMJ Open. **10**(7): p. e039426. | Wrong publication type |
| 168. Şimşir, Z., et al., *The relationship between fear of COVID-19 and mental health problems: A meta-analysis.* Death studies, 2021: p. 1-9. | Wrong publication type |
| 169. Singh, S., et al., *Impact of COVID-19 Pandemic on Mental Health of General Population and University Students Across the World: A Review.* Online Journal of Health & Allied Sciences, 2021. **20**(2): p. 1-8. | Wrong publication type |
| 170. Smith-East, M., N.E. Conner, and D.F. Neff, *Access to Mental Healthcare in the 21st Century: An Evolutionary Concept Analysis.* J Am Psychiatr Nurses Assoc: p. 10783903211011672. | Wrong publication type |
| 171. Soares de Almeida, V.R., et al., *IMPACTO PSICOSSOCIAL CAUSADO PELA PANDEMIA DA COVID-19 NOS PROFISSIONAIS DE SAÚDE.* Revista Baiana de Enfermagem, 2021. **35**: p. 1-8. | Wrong publication type |
| 172. Solehati, T., et al., *The Psychological and Sleep-Related Impact of Coronavirus Disease 2019 (COVID-19): A Systematic Review.* Kesmas: National Public Health Journal, 2021. **16**: p. 65-74. | Wrong publication type |
| 173. Sosnowska, M., *Review of research on impact of epidemic on healthcare workers, quarantined and socially isolated persons.* Psychiatria, 2020. **17**(4): p. 229-236. | Wrong publication type |
| 174. Spoorthy, M.S., S.K. Pratapa, and S. Mahant, *Mental health problems faced by healthcare workers due to the COVID-19 pandemic–a review.* Asian Journal of Psychiatry, 2020. **51**. | Wrong publication type |
| 175. Sriharan, A., et al., *Women in healthcare experiencing occupational stress and burnout during COVID-19: A rapid review.* BMJ Open, 2021. **11**(4). | Wrong publication type |
| 176. Sriharan, A., et al., *COVID-19-Related Occupational Burnout and Moral Distress among Nurses: A Rapid Scoping Review.* Nursing Leadership (1910-622X), 2021. **34**(1): p. 7-19. | Wrong publication type |
| 177. Sterina, E., et al., *Emotional Resilience of Older Adults during COVID-19: A Systematic Review of Studies of Stress and Well-Being.* CLINICAL GERONTOLOGIST. | Wrong publication type |
| 178. Stroebe, M. and H. Schut, *Bereavement in Times of COVID-19: A Review and Theoretical Framework.* Omega: Journal of Death & Dying, 2021. **82**(3): p. 500-522. | Wrong publication type |
| 179. Stuijfz, et al., *Psychological impact of an epidemic/pandemic on the mental health of healthcare professionals: a rapid review.* BMC public health, 2020. **20**(1): p. 1230. | Wrong publication type |
| 180. Su, Z., et al., *Technology-based Health Solutions for Cancer Caregivers to Better Shoulder the Impact of COVID-19: A Systematic Review Protocol.* Res Sq. | Wrong publication type |
| 181. Suwalska, J., et al., *Perinatal Mental Health during COVID-19 Pandemic: An Integrative Review and Implications for Clinical Practice.* JOURNAL OF CLINICAL MEDICINE. **10**(11). | Wrong publication type |
| 182. Szczesniak, D., et al., *The SARS-CoV-2 and mental health: From biological mechanisms to social consequences.* PROGRESS IN NEURO-PSYCHOPHARMACOLOGY & BIOLOGICAL PSYCHIATRY. **104**. | Wrong publication type |
| 183. Talevi, D., et al., *Mental health outcomes of the CoViD-19 pandemic.* Rivista di Psichiatria, 2020. **55**(3): p. 137-144. | Wrong publication type |
| 184. Tariq, M.J., et al., *Medicine resident wellness during the COVID-19 pandemic-a systematic review.* Journal of General Internal Medicine, 2021. **36**: p. S163. | Wrong publication type |
| 185. Tettamanti, M., et al., *[Impact of the Covid-19 pandemic on psychological disorders in adolescents and young adults].* Rev Med Suisse. **17**(751): p. 1593-1596. | Wrong publication type |
| 186. Thapar, S. and L. Mah, *Mental health outcomes during COVID-19: A scoping review & recommendations for geriatrics research.* American Journal of Geriatric Psychiatry, 2021. **29**(4): p. S54-S58. | Wrong publication type |
| 187. Thatrimontrichai, A., D.J. Weber, and A. Apisarnthanarak, *Mental health among healthcare personnel during COVID-19 in Asia: A systematic review.* Journal of the Formosan Medical Association, 2021. **120**(6): p. 1296-1304. | Wrong publication type |
| 188. Thombs, B.D., et al., *Curating evidence on mental health during COVID-19: A living systematic review.* Journal of Psychosomatic Research, 2020. **133**: p. N.PAG-N.PAG. | Wrong publication type |
| 189. Tian, H., et al., *Humanistic care and psychological counseling on psychological disorders in medical students after COVID-19 outbreak: A protocol of systematic review.* Medicine (Baltimore). **99**(33): p. e21484. | Wrong publication type |
| 190. Torrente, F.M., et al., *Remote non-pharmacologic interventions for sleep problems in healthcare workers during the COVID-19 pandemic.* Cochrane Database of Systematic Reviews, 2021. **2021**(5). | Wrong publication type |
| 191. Tranquilino, A.R.A., et al., *Impacts on the mental health of health professionals in front of the covid-19 pandemic.* CULTURA DE LOS CUIDADOS, 2021. **25**(60): p. 5-19. | Wrong publication type |
| 192. Uphoff, E.P., et al., *Mental health among healthcare workers and other vulnerable groups during the COVID-19 pandemic and other coronavirus outbreaks: A rapid systematic review.* PLoS ONE, 2021. **16**(8). | Wrong publication type |
| 193. Viner, R., et al., *Associations of School Closures with and without Social Lockdown on Physical and Mental Health of Children and Young People during the First COVID-19 Wave: A Systematic Review.* JAMA Pediatrics, 2021. | Wrong publication type |
| 194. Wang, F.F. and S. Boros, *Mental and physical health in general population during COVID-19: Systematic review and narrative synthesis.* BALTIC JOURNAL OF HEALTH AND PHYSICAL ACTIVITY, 2021. **13**(1): p. 91-99. | Wrong publication type |
| 195. Xiong, J., et al., *Impact of COVID-19 pandemic on mental health in the general population: A systematic review.* Journal of Affective Disorders, 2020. **277**: p. 55-64. | Wrong publication type |
| 196. Yahya, S., et al., *Covid 19 and its effect on the mental health of people in the community and pregnant women separately (a review of existing studies).* Turkish Journal of Physiotherapy and Rehabilitation, 2021. **32**(3): p. 9422-9440. | Wrong publication type |
| 197. Zafar, M. and F. Magdi, *Mental health consequence of covid 19 pandemic among health care workers: Systematic review.* International Journal of Pharmaceutical Research, 2020. **12**(4): p. 4405-4414. | Wrong publication type |
| 198. Zahmatkesh, M.R.R., et al., *The Impact of COVID-19 Outbreak on the Mental Health of the Pregnant Women: A Systematic Review.* INTERNATIONAL JOURNAL OF PEDIATRICS-MASHHAD. **9**(3): p. 13185-13192. | Wrong publication type |
| 199. Zurcher, S.J., et al., *Prevalence of Mental Health Problems During Virus Epidemics in the General Public, Health Care Workers and Survivors: A Rapid Review of the Evidence.* FRONTIERS IN PUBLIC HEALTH. **8**. | Wrong publication type |
| 200. Giannopoulou I, Galinaki S, Kollintza E, Adamaki M, Kympouropoulos S, Alevyzakis E, Tsamakis K, Tsangaris I, Spandidos DA, Siafakas N, Zoumpourlis V, Rizos E. COVID-19 and post-traumatic stress disorder: The perfect 'storm' for mental health (Review). Exp Ther Med. 2021 Oct;22(4):1162. doi: 10.3892/etm.2021.10596. | Wrong publication type |
| 201. Gerstein, L. H., & Rami, F. (2022). International Psychology and the COVID-19 Pandemic. International Perspectives in Psychology. | Wrong publication type |
| 202. Fiest, K.M., Parsons Leigh, J., Krewulak, K.D. et al. Experiences and management of physician psychological symptoms during infectious disease outbreaks: a rapid review. BMC Psychiatry 21, 91 (2021). <https://doi.org/10.1186/s12888-021-03090-9>  203. Gaigher JM, Lacerda IB, Dourado MCN. Dementia and Mental Health During the COVID-19 Pandemic: A Systematic Review. Front Psychiatry. 2022 Jul 7;13:879598. doi: 10.3389/fpsyt.2022.879598. | Wrong publication type |
| 204. Gayatri, M., & Irawaty, D. K. (2022). Family Resilience during COVID-19 Pandemic: A Literature Review. The Family Journal, 30(2), 132–138. https://doi.org/10.1177/10664807211023875 | Wrong publication type |
| 205. Gianfredi, Vincenza & Sandro, Provenzano & Santangelo, Omar. (2021). What can internet users’ behaviours reveal about the mental health impacts of the COVID-19 pandemic? A systematic review. Public Health. 198. | Wrong publication type |
| 206. Gibson, B., Schneider, J., Talamonti, D., & Forshaw, M. (2021). The impact of inequality on mental health outcomes during the COVID-19 pandemic: A systematic review. Canadian Psychology / Psychologie canadienne, 62(1), 101–126. | Wrong publication type |
| 207. Granek L, Nakash O. Oncology Healthcare Professionals' Mental Health during the COVID-19 Pandemic. Curr Oncol. 2022 Jun 2;29(6):4054-4067. doi: 10.3390/curroncol29060323. PMID: 35735432; PMCID: PMC9222050. | Wrong publication type |
| 208. Gray KL, Birtles H, Reichelt K, James IA. The experiences of care home staff during the COVID-19 pandemic: A systematic review. Aging Ment Health. 2022 Oct;26(10):2080-2089. doi: 10.1080/13607863.2021.2013433. | Wrong publication type |
| 209. Gryksa K, Neumann ID. Consequences of pandemic-associated social restrictions: Role of social support and the oxytocin system. Psychoneuroendocrinology. 2022 Jan;135:105601. doi: 10.1016/j.psyneuen.2021.105601. | Wrong publication type |
| 210. Haddad JM, Macenski C, Mosier-Mills A, Hibara A, Kester K, Schneider M, Conrad RC, Liu CH. The Impact of Social Media on College Mental Health During the COVID-19 Pandemic: a Multinational Review of the Existing Literature. Curr Psychiatry Rep. 2021 Oct 6;23(11):70. doi: 10.1007/s11920-021-01288-y. | Wrong publication type |
| 211. Haig-Ferguson A, Cooper K, Cartwright E, Loades ME, Daniels J. Practitioner review: health anxiety in children and young people in the context of the COVID-19 pandemic. Behav Cogn Psychother. 2021 Mar;49(2):129-143. | Wrong publication type |
| 212. Hannemann J, et al. The impact of the COVID-19 pandemic on the mental health of medical staff considering the interplay of pandemic burden and psychosocial resources-A rapid systematic review. PLoS One. 2022 Feb 22;17(2):e0264290. | Wrong publication type |
| 213. Hassan MHMO, Abdelhafez IHE, Ouanes S, et alMental health research in the Arab region in response to the COVID-19 pandemic: a scoping reviewGeneral Psychiatry 2022;35:e100590. doi: 10.1136/gpsych-2021-100590 | Wrong publication type |
| 214. Hernández-Díaz Y, Genis-Mendoza AD, Ramos-Méndez MÁ, Juárez-Rojop IE, Tovilla-Zárate CA, González-Castro TB, López-Narváez ML, Nicolini H. Mental Health Impact of the COVID-19 Pandemic on Mexican Population: A Systematic Review. Int J Environ Res Public Health. 2022 Jun 6;19(11):6953. doi: 10.3390/ijerph19116953. | Wrong publication type |
| 215. Huerta-González S, Selva-Medrano D, López-Espuela F, Caro-Alonso PÁ, Novo A, Rodríguez-Martín B. The Psychological Impact of COVID-19 on Front Line Nurses: A Synthesis of Qualitative Evidence. Int J Environ Res Public Health. 2021 Dec 9;18(24):12975. doi: 10.3390/ijerph182412975. PMID: 34948584; PMCID: PMC8701954. | Wrong publication type |
| 216. Husky MM, Pietrzak RH, Marx BP, Mazure CM. Research on Posttraumatic Stress Disorder in the Context of the COVID-19 Pandemic: A Review of Methods and Implications in General Population Samples. Chronic Stress (Thousand Oaks). 2021 Nov 2;5:24705470211051327. doi: 10.1177/24705470211051327. PMID: 34765850; PMCID: PMC8576091. | Wrong publication type |
| 217. Ilesanmi O, Afolabi A, Kwaghe A. A scope review on the global impact of COVID-19 lockdown on adolescents' health. Afr Health Sci. 2021 Dec;21(4):1518-1526. doi: 10.4314/ahs.v21i4.4. PMID: 35283972; PMCID: PMC8889810. | Wrong publication type |
| 218. Iyengar U, Jaiprakash B, Haitsuka H, Kim S. One Year Into the Pandemic: A Systematic Review of Perinatal Mental Health Outcomes During COVID-19. Front Psychiatry. 2021 Jun 24;12:674194. | Wrong publication type |
| 219. J Devoe D, Han A, Anderson A, Katzman DK, Patten SB, Soumbasis A, Flanagan J, Paslakis G, Vyver E, Marcoux G, Dimitropoulos G. The impact of the COVID-19 pandemic on eating disorders: A systematic review. Int J Eat Disord. 2022 Apr 5:10.1002/eat.23704. doi: 10.1002/eat.23704. | Wrong publication type |
| 220. Rhythm Joshi, Nidhi.B. Agarwal, Dinesh Bhurani, Mohd Ashif Khan. Frontline Healthcare workers suffering from psychosomatic disorders during COVID-19 (a pandemic) – A Systematic review. https://doi.org/10.1101/2021.11. 09.21266105 | Wrong publication type |
| 221. Jurecka, A.; Skucińska, P.; Gądek, A. Impact of the SARS-CoV-2 Coronavirus Pandemic on Physical Activity, Mental Health and Quality of Life in Professional Athletes—A Systematic Review. Int. J. Environ. Res. Public Health 2021, 18, 9423. https://doi.org/10.3390/ijerph18179423 | Wrong publication type |
| 222. The effects of COVID-19 on physician's burnout: A systematic review  Kang, M. J.; Park, J. H.. Journal of the Korean Medical Association ; 64(9):636-646, 2021. | Wrong publication type |
| 223. Kessler, Ronald & Chiu, Wai & Hwang, Irving & Puac Polanco, Victor & Sampson, Nancy & Ziobrowski, Hannah & Zaslavsky, Alan. (2021). Changes in Prevalence of Mental Illness Among US Adults During Compared with Before the COVID-19 Pandemic. Psychiatric Clinics of North America. 45. 10.1016/j.psc.2021.11.013. | Wrong publication type |
| 224. Khazaei P, Ramezanifar S, Gharari N, Jalali Farahani A, Salehi Sahlabadi A. Investigating the Prevalence of COVID-19 Among the Armed Forces and Its Impact on Their Mental Health: Systematic Review. J Mil Med 2022; 24 (2) :1086-1095 | Wrong publication type |
| 225. Khoundabi, B., Soltani, A., & Ahmadi Marzaleh, M. (2021). Psychological Problems of the Nursing Staff in COVID-19 Pandemic: A Systematic Review. Iranian Red Crescent Medical Journal, 23(6). | Wrong publication type |
| 226. Clare Killikelly, Lonneke I. M. Lenferink, Hanzhang Xie & Andreas Maercker (2021) Rapid Systematic Review of Psychological Symptoms in Health Care Workers COVID-19, Journal of Loss and Trauma, 26:7, 638-655 | Wrong publication type |
| 227. Koszalinski RS, Olmos B. Communication challenges in social isolation, subjective cognitive decline, and mental health status in older adults: A scoping review (2019-2021). Perspect Psychiatr Care. 2022 Oct;58(4):2741-2755. | Wrong publication type |
| 228. Kotlar B, Gerson E, Petrillo S, Langer A, Tiemeier H. The impact of the COVID-19 pandemic on maternal and perinatal health: a scoping review. Reprod Health. 2021 Jan 18;18(1):10. doi: 10.1186/s12978-021-01070-6. PMID: 33461593; PMCID: PMC7812564. | Wrong publication type |
| 229. Kunz M, Strasser M, Hasan A. Impact of the coronavirus disease 2019 pandemic on healthcare workers: systematic comparison between nurses and medical doctors. Curr Opin Psychiatry. 2021 Jul 1;34(4):413-419. | Wrong publication type |
| 230. Kuodi P, Gorelik Y, Edelstein M. Characterisation of the long-term physical and mental health consequences of SARS-CoV-2 infection: A systematic review and meta-analysis protocol. PLoS One. 2022 Apr 5;17(4):e0266232. | Wrong publication type |
| 231. Daphnée Lamarre-Choinière, Sarah Lippé. Impact de la Covid-19 sur les symptômes de dépression et d’anxiété des femmes enceintes : une revue de littérature. Revue de neuropsychologie. 2021;13(1):15-28. doi:10.1684/nrp.2021.0615 | Wrong publication type |
| 232. N. Lavenne-Collot, P. Ailliot, S. Badic, A. Favé, G. François, S. Saint-André, A. Thierry, G. Bronsard,  Les enfants suivis en psychiatrie infanto-juvénile ont ils connu la dégradation redoutée pendant la période de confinement liée à la pandémie COVID-19 ? Neuropsychiatrie de l'Enfance et de l'Adolescence, Volume 69, Issue 3,  2021, Pages 121-131, ISSN 0222-9617, https://doi.org/10.1016/j.neurenf.2021.02.006. | Wrong publication type |
| 233. Lee V, Albaum C, Tablon Modica P, Ahmad F, Gorter JW, Khanlou N, McMorris C, Lai J, Harrison C, Hedley T, Johnston P, Putterman C, Spoelstra M, Weiss JA. The impact of COVID-19 on the mental health and wellbeing of caregivers of autistic children and youth: A scoping review. Autism Res. 2021 Dec;14(12):2477-2494. | Wrong publication type |
| 234. Lieneck, C. and Bosworth, M. and Weaver, E. and Heinemann, K. and Patel, J. Protective and Non-Protective Factors of Mental Health Distress in the United States during the COVID-19 Pandemic: A Systematic Review 2021 | Wrong publication type |
| 235. Linardon, Jake and Messer, Mariel and Rodgers, Rachel F. and Fuller-Tyszkiewicz, Matthew. A systematic scoping review of research on COVID‐19 impacts on eating disorders: A critical appraisal of the evidence and recommendations for the field 2022 | Wrong publication type |
| 236. Linde, E. S. and Varga, T. V. and Clotworthy, A. Obsessive-Compulsive Disorder During the COVID-19 Pandemic-A Systematic Review 2022 | Wrong publication type |
| 237. Lindert, J. and Jakubauskiene, M. and Bilsen, J. The COVID-19 disaster and mental health-assessing, responding and recovering. 2021 | Wrong publication type |
| 238. Liu, W. and Xu, Y. B. and Ma, D. N. Work-Related Mental Health Under COVID-19 Restrictions: A Mini Literature Review. 2021 | Wrong publication type |
| 239. Łoś, K. and Kulikowska, J. and Waszkiewicz, N. The Impact of the COVID-19 Virus Pandemic on the Incidence of First Psychotic Spectrum Disorders 2022 | Wrong publication type |
| 240. Lulli, L. G. and Giorgi, G. and olfi, C. and Foti, G. and Finstad, G. L. and Arcangeli, G. and Mucci, N. Identifying Psychosocial Risks and Protective Measures for Workers' Mental Wellbeing at the Time of COVID-19: A Narrative Review 2021 | Wrong publication type |
| 241. Lund, Emily M. and Gabrielli, Joy The role of pediatric psychologists in mitigating disability-specific barriers among youth during the COVID-19 pandemic 2021 | Wrong publication type |
| 242. Lynch, S. and McDonnell, T. and Leahy, D. and Gavin, B. and McNicholas, F. PREVALENCE OF MENTAL ILLNESS IN CHILDREN AND ADOLESCENTS IN THE REPUBLIC OF IRELAND 2022 | Wrong publication type |
| 243. Machado Pavan, Fabiane and Basso da Silva, Aline and Olschowsky, Agnes and Wetze, Christine and Kenes Nunes, Cristiane and Bohnen Souza, Luíza. Covid-19 and repercussions in mental health: a narrative review of literature 2021 | Wrong publication type |
| 244. Mahira Putra R. A, Grispenjas Sumartono and Nursalam, Nursalam and Arief, Yuni Sufyanti Caused Anxiety and Coping Strategies among Nursing Staff during COVID-19 Outbreak: A Systematic Review 2021 | Wrong publication type |
| 245. Maizel, J. L. and Dixon, B. N. and Walker, A. F. Psychological Outcomes of the COVID-19 Pandemic on People with Type 1 Diabetes Globally: A Scoping Review 2022 | Wrong publication type |
| 246. Maladkar, Manish and Tekch and ani, Chitra and Bajaj, Raveena COVID-19: Impact on Mental Health 2021 | Wrong publication type |
| 247. Manchia, M. and Gathier, A. W. and Yapici-Eser, H. and Schmidt, M. V. and de Quervain, D. and van Amelsvoort, T. and Bisson, J. I. and Cryan, J. F. and Howes, O. D. and Pinto, L. and van der Wee, N. J. and Domschke, K. and Branchi, I. and Vinkers, C. H. The impact of the prolonged COVID-19 pandemic on stress resilience and mental health: A critical review across waves 2022 | Wrong publication type |
| 248. Marchi, J. and Johansson, N. and Sarkadi, A. and Warner, G. The Impact of the COVID-19 Pandemic and Societal Infection Control Measures on Children and Adolescents' Mental Health: A Scoping Review 2021 | Wrong publication type |
| 249. Marconcin, P. and Werneck, A. O. and Peralta, M. and Ihle, A. and Gouveia, ÉR and Ferrari, G. and Sarmento, H. and Marques, A. The association between physical activity and mental health during the first year of the COVID-19 pandemic: a systematic review 2022 | Wrong publication type |
| 250. Marel, Christina and Mills, Katherine L. and Teesson, Maree Substance use, mental disorders and COVID-19: a volatile mix 2021 | Wrong publication type |
| 251. Maresca, G. and Latella, D. and Carnazza, L. and Corallo, F. and Formica, C. Neuropsychological effects of COVID-19: A review | Wrong publication type |
| 252. Marquini, G. V. and Martins, S. B. and Oliveira, L. M. and Dias, M. M. and Takano, C. C. and Sartori, M. G. F. Effects of the COVID-19 Pandemic on Gynecological Health: An Integrative Review 2022 | Wrong publication type |
| 253. Martin, A. and Blom, I. M. and Whyatt, G. and Shaunak, R. and Viva, M. I. F. and Banerjee, L. A Rapid Systematic Review Exploring the Involvement of Medical Students in Pandemics and Other Global Health Emergencies 2022 | Wrong publication type |
| 254. Martin Delawalla, M. L. and Tiwari, R. and Evans, Y. N. and Rhew, I. C. and Enquobahrie, D. A. 182. The Impact of the COVID-19 Pandemic on Adolescent Social Media Use, Substance Use, and Depressive Symptoms: A Scoping Review 2022 | Wrong publication type |
| 255. Masaeli, Nassim and Farhadi, Hadi. Prevalence of Internet-based addictive behaviors during COVID-19 pandemic: a systematic review 2021 | Wrong publication type |
| 256. Mauz, E. and Eicher, S. and Peitz, D. and Junker, S. and Hölling, H. and Thom, J. Mental health of the adult population in Germany during the COVID-19 pandemic. Rapid Review 2021 | Wrong publication type |
| 257. Maye, C. E. and Wojcik, K. D. and elari, A. E. and Goodman, W. K. and Storch, E. A. Obsessive compulsive disorder during the COVID-19 pandemic: A brief review of course, psychological assessment and treatment considerations 2022 | Wrong publication type |
| 258. Mazurkiewicz, D. W. and Strzelecka, J. and Piechocka, D. I. Adverse Mental Health Sequelae of COVID-19 Pandemic in the Pregnant Population and Useful Implications for Clinical Practice 2022 | Wrong publication type |
| 259. Mazza, M. G. and Palladini, M. and Poletti, S. and Benedetti, F. Post-COVID-19 Depressive Symptoms: Epidemiology, Pathophysiology, and Pharmacological Treatment 2022 | Wrong publication type |
| 260. Mehraeen, E. and Oliaei, S. and SeyedAlinaghi, S. and Karimi, A. and Mirzapour, P. and Afsahi, A. M. and Barzegary, A. and Vahedi, F. and Soleymanzadeh, M. and Behnezhad, F. and Javaherian, M. and Zargari, G. and Mirghaderi, S. P. and Noori, T. and Sabatier, J. M. COVID-19 in Pediatrics: A Systematic Review of Current Knowledge and Practice 2022 | Wrong publication type |
| 261. Menon, S. and Bhagat, V. A Review Study on the impact of COVID-19 on Mental Health in the workplace and on working people 2021 | Wrong publication type |
| 262. Mihic-Góngora, L. and Rodriguez-Gonzalez, A. and Velasco, V. and Obispo, B. and Jiménez-Fonseca, P. and Calderon, C. Impact of the COVID-19 pandemic on care and psychological impact on cancer patients 2022 | Wrong publication type |
| 263. Minozzi, S. and Saulle, R. and Amato, L. and Davoli, M. Impact of social distancing for covid-19 on the psychological well-being of youths: A systematic review of the literature 2021 | Wrong publication type |
| 264. Mir, Girão and a, Fern and Berchelli, a and Yamamura, Mellina and Pereira, Sarah Salvador and dos Santos Pereira, Caroline and Teresinha Protti-Zanatta, Simone and Costa, Marceli Karina and Zerbetto, Sonia Regina Psychological distress among nursing professionals during the COVID-19 pandemic: Scoping Review 2021 | Wrong publication type |
| 265. Moradi, Milad and Navab, Elham and Sharifi, Farshad and Namadi, Bahareh and Rahimidoost, Mahziyar The effects of the COVID-19 pandemic on the elderly: A systematic review 2021 | Wrong publication type |
| 266. Morais Da Silva, Francine and Oliveira, Aline. Impacts of the covid-19 pandemic on the mental health and motivation of nursing professionals: an integrative review. 2021 | Wrong publication type |
| 267. Morgan, R. and Tan, H. L. and Oveisi, N. and Memmott, C. and Korzuchowski, A. and Hawkins, K. and Smith, J. Women healthcare workers' experiences during COVID-19 and other crises: A scoping review 2022 | Wrong publication type |
| 268. Moustakopoulou, L. and Adamakidou, T. and Mastrogiannis, D. and Mantoudi, A. and Apostolara, P. and Mantzorou, M. Consequences of older persons' physical and social isolation during the COVID-19 pandemic 2021 | Wrong publication type |
| 269. Moya-Salazar, J. and Nuñez, E. and Jaime-Quispe, A. and Zuñiga, N. and Loaiza-Barboza, I. L. and Balabarca, E. A. and Chicoma-Flores, K. and Cañari, B. and Contreras-Pulache, H. Substance Use in Healthcare Professionals During the COVID-19 Pandemic in Latin America: A Systematic Review and a Call for Reports 2022 | Wrong publication type |
| 270. Murphy, A. and Kirby, A. and Lawlor, A. and Drummond, F. A Systematic Review of the Impact of Covid- 19 on Cancer Patients and Survivors from an Economic, Social and Psychological Perspective 2022 | Wrong publication type |
| 271. Mushtaq, H. and Singh, S. and Mir, M. and Tekin, A. and Singh, R. and Lundeen, J. and VanDevender, K. and Dutt, T. and Khan, S. A. and Surani, S. and Kashyap, R. The Well-Being of Healthcare Workers During the COVID-19 Pandemic: A Narrative Review 2022 | Wrong publication type |
| 272. Nabizadeh, F. and Seyedalhosseini, Z. and Balab and ian, M. and Reza Rostami, M. Psychological outcomes of the COVID-19 pandemic in patients with Parkinson's disease: A systematic review 2022 | Wrong publication type |
| 273. Naff, D. and Williams, S. and Furman-Darby, J. and Yeung, M. The Mental Health Impacts of COVID-19 on PK-12 Students: A Systematic Review of Emerging Literature 2022 | Wrong publication type |
| 274. Naji, L. and Dennis, B. and Morgan, R. L. and Sanger, N. and Worster, A. and Paul, J. and Thabane, L. and Samaan, Z. Investigating and addressing the immediate and long-term consequences of the COVID-19 pandemic on patients with substance use disorders: A scoping review and evidence map protocol 2021 | Wrong publication type |
| 275. Noronha, Flavia Sharlet and Pant, Sanju and Yesodharan, Renjulal and Nayak, Asha K. and Dsa, Rochelle Jane. The Impact of COVID-19 Pandemic on the Developing Nations: Emerging Mental Health Challenges and Interventions 2021 | Wrong publication type |
| 276. O'Reilly, A. and Tibbs, M. and Booth, A. and Doyle, E. and McKeague, B. and Moore, J. A rapid review investigating the potential impact of a pandemic on the mental health of young people aged 12–25 years 2021 | Wrong publication type |
| 277. Oliva-Arocas, A. and Benavente, P. and Ronda, E. and Diaz, E. Health of International Migrant Workers During the COVID-19 Pandemic: A Scoping Review 2022 | Wrong publication type |
| 278. Oliveira, J. M. D. and Butini, L. and Pauletto, P. and Lehmkuhl, K. M. and Stefani, C. M. and Bolan, M. and Guerra, E. and Dick, B. and De Luca Canto, G. and Massignan, C. Mental health effects prevalence in children and adolescents during the COVID-19 pandemic: A systematic review 2022 | Wrong publication type |
| 279. Pai, N. and Vella, S. L. The physical and mental health consequences of social isolation and loneliness in the context of COVID-19 2022 | Wrong publication type |
| 280. Parlapani, Eleni and Holeva, Vasiliki and Nikopoulou, Vasiliki Aliki and Kaprinis, Stergios and Nouskas, Ioannis and Diakogiannis, Ioannis A review on the COVID-19-related psychological impact on older adults: vulnerable or not?2021 | Wrong publication type |
| 281. Patel, U. K. and Abdulqader, M. and Ortiz, J. F. and Patel, A. and Urhoghide, E. and Khurana, M. and Parulekar, A. and Bhriguvanshi, A. and Mistry, A. and Patel, R. and Patel, N. and Kogulavadanan, A. and Shah, S. Long-term neuro-cognitive effects in COVID-19 patients following hospital discharge-a systematic review 2021 | Wrong publication type |
| 282. Peck, J. A. and Porter, T. H. Pandemics and the Impact on Physician Mental Health: A Systematic Review | Wrong publication type |
| 283. Pellino, V. C. and Lovecchio, N. and Puci, M. V. and Marin, L. and Gatti, A. and Pirazzi, A. and Negri, F. and Ferraro, O. E. and oni, M. Effects of the lockdown period on the mental health of elite athletes during the COVID-19 pandemic: a narrative review | Wrong publication type |
| 284. Pfefferbaum, B. Children’s Psychological Reactions to the COVID-19 Pandemic 2021 | Wrong publication type |
| 285. Pfefferbaum, Betty Challenges for Child Mental Health Raised by School Closure and Home Confinement During the COVID-19 Pandemic 2021 | Wrong publication type |
| 286. Porter, B. and Zile, A. and Peryer, G. and Farquhar, M. and erson, K. The impact of providing end-of-life care during a pandemic on the mental health and wellbeing of health and social care staff: Systematic review and meta-synthesis 2021 | Wrong publication type |
| 287. Purnama, A. and Susaldi and Mukhlida, H. Z. and Maulida, H. H. and Purwati, N. H. Mental health in health students during coronavirus disease-19: Systematic review 2021 | Wrong publication type |
| 288. Putri, C. and Arisa, J. and Hananto, J. E. and Hariyanto, T. I. and Kurniawan, A. Psychiatric sequelae in COVID-19 survivors: A narrative review 2021 | Wrong publication type |
| 289. Rad, M. and Rad, M. and Hefazi Torghabeh, L. and Fatemi, N. and Maleki, M. M. and Seifi, Z. Increased nurses' anxiety disorder during the COVID-19 outbreak 2022 | Wrong publication type |
| 290. Rahman, A. A scoping review of COVID-19-related stress coping resources among nurses 2022 | Wrong publication type |
| 291. Raiff, E. M. and D'Antonio, K. M. and Mai, C. and Monk, C. Mental Health in Obstetric Patients and Providers During the COVID-19 Pandemic 2022 | Wrong publication type |
| 292. Raihan, Mohammad Mojammel Hussain Mental health consequences of COVID-19 pandemic on adult population: a systematic review 2021 | Wrong publication type |
| 293. Rajmil, L. and Hjern, A. and Boran, P. and Gunnlaugsson, G. and Kraus De Camargo, O. and Raman, S. Impact of lockdown and school closure on children's health and well-being during the first wave of COVID-19: A narrative review 2021 | Wrong publication type |
| 294. Ramos-Vera, C. and García-Ampudia, L. and Serpa-Barrientos, A. An alternative network analysis in the exploration of mental health states, chronic conditions and COVID-19 2022 | Wrong publication type |
| 295. Renaud-Charest, O. and Lui, L. M. W. and Esk and er, S. and Ceban, F. and Ho, R. and Di Vincenzo, J. D. and Rosenblat, J. D. and Lee, Y. and Subramaniapillai, M. and McIntyre, R. S. Onset and frequency of depression in post-COVID-19 syndrome: A systematic review 2021 | Wrong publication type |
| 296. Rodríguez-Fernández, P. and González-Santos, J. and Santamaría-Peláez, M. and Soto-Cámara, R. and Sánchez-González, E. and González-Bernal, J. J. Psychological effects of home confinement and social distancing derived from covid-19 in the general population—a systematic review 2021 | Wrong publication type |
| 297. Rosales Vaca, K. M. and Cruz Barrientos, O. I. and Girón López, S. and Noriega, S. and More Árias, A. and Guariente, S. M. M. and Zazula, R. Mental health of healthcare workers of Latin American countries: a review of studies published during the first year of COVID-19 pandemic 2022 | Wrong publication type |
| 298. Ruksakulpiwat, S. and Zhou, W. and Phianhasin, L. and Benjasirisan, C. and Fan, Y. and Su, T. and Chiaranai, C.The experience of caregivers of chronically ill patients during the COVID-19: A Systematic Review 2021 | Wrong publication type |
| 299. Russo, M. and Calisi, D. and De Rosa, M. A. and Evangelista, G. and Consoli, S. and Dono, F. and Santilli, M. and Gambi, F. and Onofrj, M. and Di Giannantonio, M. and Parruti, G. and Sensi, S. L. COVID-19 and first manic episodes: a systematic review 2022 | Wrong publication type |
| 300. Robson da Silva, Roni and Andrade da Silva et al. PSYCHOSOCIAL LOAD AND BURNOUT SYNDROME IN HEALTHCARE PROFESSIONALS IN THE FIGHT AGAINST COVID-19 PANDEMIC 2021 | Wrong publication type |
| 301. Robson da Silva, Roni and Barbosa de Moura, Le and ro and da Costa Campos, Eliseu and Pella do Império, João Alberto and Alves Ribeiro, Alex and ro and Alencar, Icaro Ferracini and Ferreira da Silva, Diego Psychological impacts and vulnerabilities of health professionals in the SARS-CoV-2 pandemic 2021 | Wrong publication type |
| 302. Rodrigues da Silva, Roger and Adelmo da Silva Filho, José and Lima de Oliveira, Jessica and Branco Cavalcante de Meneses, Jayana Castelo and Neves de Oliveira, Camila Almeida and Alves Pinto, Antonio Germane Efeitos do isolamento social na pandemia da covid-19 na saúde mental da população 2021 | Wrong publication type |
| 303. Ruta, Federico and Dal Mas, Francesca and Biancuzzi, Helena and Ferrara, Paolo and Della Monica, AnnalisaCovid-19 e salute mentale del personale infermieristico in prima linea: una revisione della letteratura 2021 Not a systematic review or meta-analysis | Wrong publication type |
| 304. Ahmed M. Abbas, Asmaa AbouBakr, Sara Magdy, Alshaima Refai, Yasmin Ismail, Nourhan Mahmoud & Mai E. AbuElmagd (2021) Psychological effect of COVID-19 on medical health-care workers, International Journal of Psychiatry in Clinical Practice, 25:2, 140-141, DOI: 10.1080/13651501.2020.1791903 | Wrong publication type |
| 305. Abdel-Bakky MS, Amin E, Faris TM, Abdellatif AAH. Mental depression: Relation to different disease status, newer treatments and its association with COVID-19 pandemic (Review). Mol Med Rep. 2021 Dec;24(6):839. doi: 10.3892/mmr.2021.12479. Epub 2021 Oct 11. PMID: 34633054; PMCID: PMC8524409. | Wrong publication type |
| 306. Abdelbasset WK, Nambi G, Eid MM, Elkholi SM. Physical activity and mental well-being during COVID-19 pandemic. World J Psychiatry. 2021 Dec 19;11(12):1267-1273. doi: 10.5498/wjp.v11.i12.1267. PMID: 35070776; PMCID: PMC8717035. | Wrong publication type |
| 307. Agarwal, V., L., G. and B.K., S. (2021), "Impact of COVID-19 on the mental health among children in China with specific reference to emotional and behavioral disorders", International Journal of Human Rights in Healthcare, Vol. 14 No. 2, pp. 182-188. https://doi.org/10.1108/IJHRH-05-2020-0035 | Wrong publication type |
| 308. Aknin LB, De Neve JE, Dunn EW, Fancourt DE, Goldberg E, Helliwell JF, Jones SP, Karam E, Layard R, Lyubomirsky S, Rzepa A, Saxena S, Thornton EM, VanderWeele TJ, Whillans AV, Zaki J, Karadag O, Ben Amor Y. Mental Health During the First Year of the COVID-19 Pandemic: A Review and Recommendations for Moving Forward. Perspect Psychol Sci. 2022 Jul;17(4):915-936. doi: 10.1177/17456916211029964. Epub 2022 Jan 19. PMID: 35044275; PMCID: PMC9274782. | Wrong publication type |
| 309. Alanazi TNM, McKenna L, Buck M, Alharbi RJ. Reported effects of the COVID-19 pandemic on the psychological status of emergency healthcare workers: A scoping review. Australas Emerg Care. 2022 Sep;25(3):197-212. doi: 10.1016/j.auec.2021.10.002. Epub 2021 Nov 12. PMID: 34802977; PMCID: PMC8585598. | Wrong publication type |
| 310. Ali E. COVID-19, the Child, & Mental health: A Systematic Review 50th Annual Meeting of the Child Neurology Society. Ann Neurol. 2021 Sep;90 Suppl 26:S1-S173. doi: 10.1002/ana.26177. PMID: 34570386. | Wrong publication type |
| 311. Almeda, N., García-Alonso, C. & Salvador-Carulla, L. Mental health planning at a very early stage of the COVID-19 crisis: a systematic review of online international strategies and recommendations. BMC Psychiatry 21, 43 (2021). https://doi.org/10.1186/s12888-020-03015-y | Wrong publication type |
| 312. AlQaatri, Hamda Ahmed J. H. Increased Psychological Disorders among Pediatric Population during Covid. Middle East Journal of Family Medicine . Dec2021, Vol. 19 Issue 12, p60-61. 2p. | Wrong publication type |
| 313. Ansariniaki, M., Abounoori, M., & Babakhanian, M. (2021). A Systematic Review of Anxiety during Pregnancy in the Period of the COVID-19 Pandemic. International Journal of Pediatrics, 9(11), 14756-14771. doi: 10.22038/ijp.2021.54774.4321 | Wrong publication type |
| 314. Ashraf, S.; Yaqoob, A.. Impact of COVID-19 on Mental Health of Children and Adolescents: A Narrative Review. Journal of Clinical and Diagnostic Research ; 16(4):VE01-VE03, 2022. | Wrong publication type |
| 315. Atheer, F.; Cavaliere, R.; Khajah, A.; Salman, M.; McNee, M.; Duffy, R. The psychological effect of covid-19 on pregnant women. Irish Medical Journal ; 114(8), 2021. | Wrong publication type |
| 316. Bahamdan AS. Review of the Psychological Impact of COVID-19 Pandemic on Healthcare Workers in Saudi Arabia. Risk Manag Healthc Policy. 2021 Oct 1;14:4105-4111. doi: 10.2147/RMHP.S324938. PMID: 34629915; PMCID: PMC8493476. | Wrong publication type |
| 317. Balai MK, Avasthi RD, Va R, Jonwal A. Psychological Impacts among Health Care Personnel during COVID-19 Pandemic: A Systematic Review. J Caring Sci. 2022 Apr 17;11(2):118-125. doi: 10.34172/jcs.2022.14. PMID: 35919274; PMCID: PMC9339130. | Wrong publication type |
| 318. Balakrishnan V, Ng KS, Kaur W, Lee ZL. COVID-19 mental health prevalence and its risk factors in South East Asia. Curr Psychol. 2022 Jan 12:1-16. doi: 10.1007/s12144-021-02556-z. Epub ahead of print. PMID: 35035200; PMCID: PMC8752185. | Wrong publication type |
| 319. Barrett D, Twycross A. Impact of COVID-19 on nursing students' mental health: a systematic review and meta-analysis. Evid Based Nurs. 2022 Jan;25(1):8-9. doi: 10.1136/ebnurs-2021-103500. Epub 2021 Dec 14. PMID: 34907093. | Wrong publication type |
| 320. Bartek N, Peck JL, Garzon D, VanCleve S. Addressing the Clinical Impact of COVID-19 on Pediatric Mental Health. J Pediatr Health Care. 2021 Jul-Aug;35(4):377-386. doi: 10.1016/j.pedhc.2021.03.006. Epub 2021 Mar 24. PMID: 34078570; PMCID: PMC7988467. | Wrong publication type |
| 321. Benzinger P, Kuru S, Keilhauer A, Hoch J, Prestel P, Bauer JM, Wahl HW. Psychosoziale Auswirkungen der Pandemie auf Pflegekräfte und Bewohner von Pflegeheimen sowie deren Angehörige – Ein systematisches Review [Psychosocial effects of the pandemic on staff and residents of nursing homes as well as their relatives-A systematic review]. Z Gerontol Geriatr. 2021 Mar;54(2):141-145. German. doi: 10.1007/s00391-021-01859-x. Epub 2021 Feb 23. PMID: 33624143; PMCID: PMC7901511. | Wrong publication type |
| 322. Bera L, Souchon M, Ladsous A, Colin V, Lopez-Castroman J. Emotional and Behavioral Impact of the COVID-19 Epidemic in Adolescents. Curr Psychiatry Rep. 2022 Jan;24(1):37-46. doi: 10.1007/s11920-022-01313-8. Epub 2022 Feb 1. PMID: 35102536; PMCID: PMC8803571. | Wrong publication type |
| 323.Boden M, Zimmerman L, Azevedo KJ, Ruzek JI, Gala S, Abdel Magid HS, Cohen N, Walser R, Mahtani ND, Hoggatt KJ, McLean CP. Addressing the mental health impact of COVID-19 through population health. Clin Psychol Rev. 2021 Apr;85:102006. doi: 10.1016/j.cpr.2021.102006. Epub 2021 Mar 5. PMID: 33714167; PMCID: PMC7934657. | Wrong publication type |
| 324. Borel M, Xie L, Kapera O, Mihalcea A, Kahn J, Messiah SE. Long-term physical, mental and social health effects of COVID-19 in the pediatric population: a scoping review. World J Pediatr. 2022 Mar;18(3):149-159. doi: 10.1007/s12519-022-00515-7. Epub 2022 Feb 3. PMID: 35118594; PMCID: PMC8812346. | Wrong publication type |
| 325. Bottemanne H, Delaigue F, Lemogne C. SARS-CoV-2 Psychiatric Sequelae: An Urgent Need of Prevention. Front Psychiatry. 2021 Sep 9;12:738696. doi: 10.3389/fpsyt.2021.738696. PMID: 34566731; PMCID: PMC8458863. | Wrong publication type |
| 326. Bottemanne H, Vahdat B, Jouault C, Tibi R and Joly L (2022) Becoming a Mother During COVID-19 Pandemic: How to Protect Maternal Mental Health Against Stress Factors. Front. Psychiatry 12:764207. doi: 10.3389/fpsyt.2021.764207 | Wrong publication type |
| 327. Boulkrane MS, Ilina V, Melchakov R, Arisov M, Fedotova J, Gozzo L, Drago F, Lu W, Sarapultsev A, Tseilikman V, Baranenko D. SARS-Cov-2 Damage on the Nervous System and Mental Health. Curr Neuropharmacol. 2022;20(2):412-431. doi: 10.2174/1570159X19666210629151303. PMID: 34191699; PMCID: PMC9413788. | Wrong publication type |
| 328. Bourmistrova NW, Solomon T, Braude P, Strawbridge R, Carter B. Long-term effects of COVID-19 on mental health: A systematic review. J Affect Disord. 2022 Feb 15;299:118-125. doi: 10.1016/j.jad.2021.11.031. Epub 2021 Nov 16. PMID: 34798148; PMCID: PMC8758130. | Wrong publication type |
| 329. Bowman C, Branjerdporn G, Turner K, Kamara M, Tyagi N, Reyes NJD, Stapelberg NJC. The impact of viral epidemics and pandemics on acute mental health service use: an integrative review. Health Psychol Rev. 2021 Mar;15(1):1-33. doi: 10.1080/17437199.2021.1886864. Epub 2021 Feb 23. PMID: 33550940. | Wrong publication type |
| 330. Brasso C, Bellino S, Blua C, Bozzatello P, Rocca P. The Impact of SARS-CoV-2 Infection on Youth Mental Health: A Narrative Review. Biomedicines. 2022; 10(4):772. https://doi.org/10.3390/biomedicines10040772 | Wrong publication type |
| 331. Brooks SK, Weston D, Greenberg N. Social and psychological impact of the COVID-19 pandemic on people with Parkinson's disease: a scoping review. Public Health. 2021 Oct;199:77-86. doi: 10.1016/j.puhe.2021.08.014. Epub 2021 Sep 1. PMID: 34571441; PMCID: PMC8407946. | Wrong publication type |
| 332. Buselli R, Corsi M, Veltri A, Baldanzi S, Chiumiento M, Lupo ED, Marino R, Necciari G, Caldi F, Foddis R, Guglielmi G, Cristaudo A. Mental health of Health Care Workers (HCWs): a review of organizational interventions put in place by local institutions to cope with new psychosocial challenges resulting from COVID-19. Psychiatry Res. 2021 May;299:113847. doi: 10.1016/j.psychres.2021.113847. Epub 2021 Mar 2. | Wrong publication type |
| 333. Caffo E, Asta L, Scandroglio F. Predictors of mental health worsening among children and adolescents during the coronavirus disease 2019 pandemic. Curr Opin Psychiatry. 2021 Nov 1;34(6):624-630. doi: 10.1097/YCO.0000000000000747. PMID: 34494973; PMCID: PMC8500206. | Wrong publication type |
| 334. Campos Oliveira, Jamille; Pedreira Rodrigues, Jaqueline; Gonçalves Fonseca, Keccya Nunes. Illness and mental health facing the covid-19 pandemic scenario experienced by nursing professionals. Saude Coletiva - Volume 12, Issue 0, pp. 9578-9584. | Wrong publication type |
| 335. Campos-Garzón C, Riquelme-Gallego B, de la Torre-Luque A, Caparrós-González RA. Psychological Impact of the COVID-19 Pandemic on Pregnant Women: A Scoping Review. Behav Sci (Basel). 2021 Dec 16;11(12):181. doi: 10.3390/bs11120181. PMID: 34940116; PMCID: PMC8698569. | Wrong publication type |
| 336. Carbone EA, de Filippis R, Roberti R, Rania M, Destefano L, Russo E, De Sarro G, Segura-Garcia C, De Fazio P. The Mental Health of Caregivers and Their Patients With Dementia During the COVID-19 Pandemic: A Systematic Review. Front Psychol. 2021 Dec 24;12:782833. doi: 10.3389/fpsyg.2021.782833. PMID: 35002872; PMCID: PMC8740146. | Wrong publication type |
| 337. Chandler, A.; Wank, A.; Vanuk, J.; O'Connor, M. F.; Dreifuss, B.; Dreifuss, H.; Ellingson, K.; Kahn, S.; Friedman, S.; Athey, A..ONLINE PSYCHOLOGICAL FIRST AID FOR HEALTHCARE WORKERS: THE HCW HOSTED ICARE MODEL IN RESPONSE TO COVID-19. Psychosomatic Medicine ; 83(7):A41-A41, 2021. | Wrong publication type |
| 338. Chamaa F, Bahmad HF, Darwish B, Kobeissi JM, Hoballah M, Nassif SB, Ghandour Y, Saliba JP, Lawand N, Abou-Kheir W. PTSD in the COVID-19 Era. Curr Neuropharmacol. 2021;19(12):2164-2179. doi: 10.2174/1570159X19666210113152954. PMID: 33441072; PMCID: PMC9185760. | Wrong publication type |
| 339. Chaudhary FA, Fazal A, Ahmad B, Khattak O, Hyder M, Javaid MM, Iqbal A, Issrani R. The Impact of COVID-19 Pandemic on the Psychological Health and Dental Practice of Oral Healthcare Workers: A Scoping Review. Risk Manag Healthc Policy. 2022 Jul 26;15:1421-1431. doi: 10.2147/RMHP.S370125. PMID: 35923553; PMCID: PMC9342663. | Wrong publication type |
| 340. Chen C, Tang J, Wang C, Wen W, Cheng Y, Zhou M, Wu Q, Zhang X, Wang M, Feng Z, Yang D. Meta-analysis of post-traumatic stress disorder and COVID-19 in patients discharged. J Infect. 2022 Jun;84(6):834-872. doi: 10.1016/j.jinf.2022.03.008. Epub 2022 Mar 10. PMID: 35283185; PMCID: PMC8907132. | Wrong publication type |
| 341. Çıkrıkçı Ö, Çıkrıkçı N, Griffiths M. Fear of COVID-19, stress and depression: A meta-analytic test of the mediating role of anxiety. Psychol Psychother. 2022 Dec;95(4):853-874. doi: 10.1111/papt.12406. Epub 2022 Jun 7. PMID: 35670451; PMCID: PMC9348301. | Wrong publication type |
| 342. Clare Vigilar, Maria Veronica, Trotter, Jerry. The Impact of COVID-19 Virtual Schooling on Child and Adolescent Mental Health: Considerations for Force Readiness. J Am Acad Child Adolesc Psychiatry. 2021 Oct; 60(10): S223. | Wrong publication type |
| 343. Clemente-Suárez VJ, Martínez-González MB, Benitez-Agudelo JC, Navarro-Jiménez E, Beltran-Velasco AI, Ruisoto P, Diaz Arroyo E, Laborde-Cárdenas CC, Tornero-Aguilera JF. The Impact of the COVID-19 Pandemic on Mental Disorders. A Critical Review. Int J Environ Res Public Health. 2021 Sep 24;18(19):10041. doi: 10.3390/ijerph181910041. PMID: 34639341; PMCID: PMC8507604. | Wrong publication type |
| 344. Corsi M, Orsini A, Pedrinelli V, Santangelo A, Bertelloni CA, Carli N, Buselli R, Peroni D, Striano P, Dell'Osso L, Carmassi C. PTSD in parents of children with severe diseases: a systematic review to face Covid-19 impact. Ital J Pediatr. 2021 Jan 14;47(1):8. doi: 10.1186/s13052-021-00957-1. PMID: 33446246; PMCID: PMC7807213. | Wrong publication type |
| 345. Cuellar, Bianca, Henderson, Sally, Briggs, Emily. Assessing the impact the Covid-19 pandemic has had on children’s mental health presentations to a tertiary children’s emergency department. Archives of Disease in Childhood ; 106(Suppl 1):A371-A372, 2021. | Wrong publication type |
| 346. Curran E, Nalder L, Koye D, Hocking J, Coulson B, Khalid S, Loi SM, Lautenschlager NT. COVID-19 and mental health: Impact on symptom burden in older people living with mental illness in residential aged care. Australas J Ageing. 2022 Dec;41(4):522-529. doi: 10.1111/ajag.13042. Epub 2022 Feb 7. PMID: 35129267; PMCID: PMC9111336. | Wrong publication type |
| 347. De Kock JH, Latham HA, Cowden RG. The mental health of healthcare workers during the COVID-19 pandemic: a narrative review. Curr Opin Psychiatry. 2022 Sep 1;35(5):311-316. doi: 10.1097/YCO.0000000000000805. Epub 2022 Jul 18. | Wrong publication type |
| 348. De Kock JH, Latham HA, Leslie SJ, Grindle M, Munoz SA, Ellis L, Polson R, O'Malley CM. A rapid review of the impact of COVID-19 on the mental health of healthcare workers: implications for supporting psychological well-being. BMC Public Health. 2021 Jan 9;21(1):104. doi: 10.1186/s12889-020-10070-3. | Wrong publication type |
| 349. Oliveira JMD, Butini L, Pauletto P, Lehmkuhl KM, Stefani CM, Bolan M, Guerra E, Dick B, De Luca Canto G, Massignan C. Mental health effects prevalence in children and adolescents during the COVID-19 pandemic: A systematic review. Worldviews Evid Based Nurs. 2022 Apr;19(2):130-137. doi: 10.1111/wvn.12566. Epub 2022 Mar 1. PMID: 35229967; PMCID: PMC9115455. | Wrong publication type |
| 350. De Picker LJ, Dias MC, Benros ME, Vai B, Branchi I, Benedetti F, Borsini A, Leza JC, Kärkkäinen H, Männikkö M, Pariante CM, Güngör ES, Szczegielniak A, Tamouza R, van der Markt A, Fusar-Poli P, Beezhold J, Leboyer M. Severe mental illness and European COVID-19 vaccination strategies. Lancet Psychiatry. 2021 May;8(5):356-359. doi: 10.1016/S2215-0366(21)00046-8. Epub 2021 Feb 17. Erratum in: Lancet Psychiatry. 2021 Apr;8(4):e12. PMID: 33609450; PMCID: PMC7906735. | Wrong publication type |
| 351. de Sousa Moreira JL, Barbosa SMB, Vieira JG, Chaves NCB, Felix EBG, Feitosa PWG, da Cruz IS, da Silva CGL, Neto MLR. The psychiatric and neuropsychiatric repercussions associated with severe infections of COVID-19 and other coronaviruses. Prog Neuropsychopharmacol Biol Psychiatry. 2021 Mar 2;106:110159. doi: 10.1016/j.pnpbp.2020.110159. Epub 2020 Nov 2. PMID: 33147504; PMCID: PMC7605739. | Wrong publication type |
| 352. Laurent A, Fournier A, Poujol A-L, Deltour V, Lheureux F, Meunier-Beillard N, Loiseau M, Ecarnot F, Rigaud J-P, Binquet C, Quenot J-P. Psychological impact of COVID-19 pandemic on healthcare professionals in intensive care. Méd. Intensive Réa. [Internet]. 2021 Jun. 16 [cited 2023 Jan. 11];30(Hors-série 1):69-74. Available from: https://revue-mir.srlf.org/index.php/mir/article/view/1539 | Wrong publication type |
| 353. Dorri M, Mozafari Bazargany MH, Khodaparast Z, Bahrami S, Seifi Alan M, Rahimi F, Kamipoor Z, Niksima MM, Dehghan H, Rastad H. Psychological problems and reduced health-related quality of life in the COVID-19 survivors. J Affect Disord Rep. 2021 Dec;6:100248. doi: 10.1016/j.jadr.2021.100248. Epub 2021 Oct 7. PMID: 34642678; PMCID: PMC8495058. | Wrong publication type |
| 354. Dos Santos ERR, Silva de Paula JL, Tardieux FM, Costa-E-Silva VN, Lal A, Leite AFB. Association between COVID-19 and anxiety during social isolation: A systematic review. World J Clin Cases. 2021 Sep 6;9(25):7433-7444. doi: 10.12998/wjcc.v9.i25.7433. PMID: 34616809; PMCID: PMC8464456. | Wrong publication type |
| 355. Vanderlind WM, Rabinovitz BB, Miao IY, Oberlin LE, Bueno-Castellano C, Fridman C, Jaywant A, Kanellopoulos D. A systematic review of neuropsychological and psychiatric sequalae of COVID-19: implications for treatment. Curr Opin Psychiatry. 2021 Jul 1;34(4):420-433. doi: 10.1097/YCO.0000000000000713. PMID: 34016818; PMCID: PMC8183238. | Wrong publication type |
| 356. Exner-Cortens D, Baker E, Gray S, Fernandez Conde C, Rivera RR, Van Bavel M, Vezina E, Ambrose A, Pawluk C, Schwartz KD, Arnold PD. School-Based Suicide Risk Assessment Using eHealth for Youth: Systematic Scoping Review. JMIR Ment Health. 2021 Sep 21;8(9):e29454. doi: 10.2196/29454. PMID: 34546178; PMCID: PMC8493464. | Wrong publication type |
| 357. Pandey K, Thurman M, Johnson SD, Acharya A, Johnston M, Klug EA, Olwenyi OA, Rajaiah R, Byrareddy SN. Mental Health Issues During and After COVID-19 Vaccine Era. Brain Res Bull. 2021 Nov;176:161-173. doi: 10.1016/j.brainresbull.2021.08.012. Epub 2021 Sep 3. PMID: 34487856; PMCID: PMC8414813. | Wrong publication type |
| 358. Boden M, Cohen N, Froelich JM, Hoggatt KJ, Abdel Magid HS, Mushiana SS. Mental disorder prevalence among populations impacted by coronavirus pandemics: A multilevel meta-analytic study of COVID-19, MERS & SARS. Gen Hosp Psychiatry. 2021 May-Jun;70:124-133. doi: 10.1016/j.genhosppsych.2021.03.006. Epub 2021 Mar 12. | Wrong publication type |
| 359. Bonati M, Campi R, Segre G. Psychological impact of the quarantine during the COVID-19 pandemic on the general European adult population: a systematic review of the evidence. Epidemiol Psychiatr Sci. 2022 Apr 27;31:e27. doi: 10.1017/S2045796022000051. PMID: 35475479; PMCID: PMC9069583. | Wrong publication type |
| 360. Chen J, Zhang SX, Yin A, Yáñez JA. Mental health symptoms during the COVID-19 pandemic in developing countries: A systematic review and meta-analysis. J Glob Health. 2022 May 23;12:05011. doi: 10.7189/jogh.12.05011. PMID: 35604881; PMCID: PMC9126304. | Wrong publication type |
| 361. Chutiyami M, Cheong AMY, Salihu D, Bello UM, Ndwiga D, Maharaj R, Naidoo K, Kolo MA, Jacob P, Chhina N, Ku TK, Devar L, Pratitha P, Kannan P. COVID-19 Pandemic and Overall Mental Health of Healthcare Professionals Globally: A Meta-Review of Systematic Reviews. Front Psychiatry. 2022 Jan 17;12:804525. doi: 10.3389/fpsyt.2021.804525. PMID: 35111089; PMCID: PMC8801501. | Wrong publication type |
| 362. Medeiros KSd, Ferreira de Paiva LM, Macêdo LTdA, Farias de Souza W, Soares da Silva LA, Sarmento ACA, et al. (2021) Prevalence of Burnout Syndrome and other psychiatric disorders among health professionals during the COVID-19 pandemic: A systematic review and meta-analysis protocol. PLoS ONE 16(12): e0260410. https://doi. org/10.1371/journal.pone.0260410 | Wrong publication type |
| 363. Delanerolle, Gayathri & Mccauley, Mary & Hirsch, Martin & Yutain, Zeng & Cong, Xu & Cavalini, Heitor & Shetty, Ashish & Rathod, Shanaya & Shi, Jian & Hapangama, Dharani & Phiri, Peter. (2022). The prevalence of mental ill-health in women during pregnancy and after childbirth during the Covid-19 pandemic: a Systematic review and Meta-analysis (Preprint). 10.1101/2022.06.13.22276327. | Wrong publication type |
| 364. Dragioti E, Tsartsalis D, Mentis M, Mantzoukas S, Gouva M. Impact of the COVID-19 pandemic on the mental health of hospital staff: An umbrella review of 44 meta-analyses. Int J Nurs Stud. 2022 Jul;131:104272. doi: 10.1016/j.ijnurstu.2022.104272. Epub 2022 Apr 27. PMID: 35576637; PMCID: PMC9045868. | Wrong publication type |
| 365. Elharake JA, Akbar F, Malik AA, Gilliam W, Omer SB. Mental Health Impact of COVID-19 among Children and College Students: A Systematic Review. Child Psychiatry Hum Dev. 2022 Jan 11:1–13. doi: 10.1007/s10578-021-01297-1. Epub ahead of print. PMID: 35013847; PMCID: PMC8747859. | Wrong publication type |
| 366. Şimşir Z, Koç H, Seki T, Griffiths MD. The relationship between fear of COVID-19 and mental health problems: A meta-analysis. Death Stud. 2022;46(3):515-523. doi: 10.1080/07481187.2021.1889097. Epub 2021 Feb 27. PMID: 33641626. | Wrong publication type |
| 367. Shanbehzadeh, S., et al. (2021). "Physical and mental health complications post-COVID-19: Scoping review." Journal of Psychosomatic Research 147: N.PAG-N.PAG. | Wrong publication type |
| 368. Shi, H. R., et al. (2022). "COVID-19 in China: A Rapid Review of the Impacts on the Mental Health of Undergraduate Students." Frontiers in public health 10. | Wrong publication type |
| 369. Stavridou, A., et al. (2022). "Psycho-emotional consequences in pregnant women during the COVID-19 pandemic." Folia Med (Plovdiv) 64(1): 21-26. | Wrong publication type |
| 370. Tng, X. J. J., et al. (2022). "Psychological sequelae within different populations during the COVID-19 pandemic: a rapid review of extant evidence." Singapore Medical Journal 63(5): 229-235. | Wrong publication type |
| 371. Verbiest, M. E. A., et al. (2022). "Health impact of the first and second wave of COVID-19 and related restrictive measures among nursing home residents: a scoping review." BMC Health Serv Res 22(1): 921. | Wrong publication type |
| 372. Walsh, K., et al. (2021). "Narrative review: COVID-19 and pediatric anxiety." J Psychiatr Res 144: 421-426. | Wrong publication type |
| 373. Winwood, J. J., et al. (2021). "Exploring the Social Impacts of the COVID-19 Pandemic on People Living with HIV (PLHIV): A Scoping Review." AIDS Behav 25(12): 4125-4140. | Wrong publication type |
| 374. Zhao, Y., et al. (2022). "COVID-19 and mental health in Australia - a scoping review." BMC PUBLIC HEALTH 22(1): 1200. | Wrong publication type |
| 375. Zhi, J. C., et al. (2022). "[Psychological and behavioral problems in children and adolescents during the coronavirus disease 2019 epidemic: a Scoping review]." Zhongguo Dang Dai Er Ke Za Zhi 24(7): 728-735. | Wrong publication type |
| 376. Saeed, H., et al. (2022). "Anxiety Linked to COVID-19: A Systematic Review Comparing Anxiety Rates in Different Populations." Int J Environ Res Public Health 19(4). | Wrong publication type |
| 377. Sahebi, A., et al. (2021). "The prevalence of anxiety and depression among healthcare workers during the COVID-19 pandemic: An umbrella review of meta-analyses." PROGRESS IN NEURO-PSYCHOPHARMACOLOGY & BIOLOGICAL PSYCHIATRY 107. | Wrong publication type |
| 378. Sahebi, A., et al. (2021). "The Prevalence of Post-traumatic Stress Disorder Among Health Care Workers During the COVID-19 Pandemic: An Umbrella Review and Meta-Analysis." Front Psychiatry 12: 764738. | Wrong publication type |
| 379. Sakti, A. M. T., et al. (2022). "Impact of COVID-19 on School Populations and Associated Factors: A Systematic Review." International Journal of Environmental Research and Public Health 19(7). | Wrong publication type |
| 380. Saladino, V., et al. (2022). "Healthcare Professionals, Post-traumatic Stress Disorder, and COVID-19: A Review of the Literature." Frontiers in Psychiatry 12. | Wrong publication type |
| 381. Samji, H., et al. (2022). "Review: Mental health impacts of the COVID-19 pandemic on children and youth - a systematic review." Child Adolesc Ment Health 27(2): 173-189. | Wrong publication type |
| 382. Sánchez-García, M., et al. (2022). "[Neuropsychiatric symptoms in people living with dementia related to COVID-19 pandemic lockdown. Exploratory systematic review]." Rev Neurol 74(3): 83-92. | Wrong publication type |
| 383. Schou, T. M., et al. (2021). "Psychiatric and neuropsychiatric sequelae of COVID-19—A systematic review." Brain, Behavior, and Immunity 97: 328-348. | Wrong publication type |
| 384. Scortegagna, S. A., et al. (2021). "Mental health in health professionals facing Covid-19: A systematic review." Psicologia: Teoria e Prática 23(1): 1-23. | Wrong publication type |
| 385. Shankar, A., et al. (2021). "The psychological impact of COVID-19 on socially isolated individuals – a systematic review." Mental Health Review Journal 26(3): 247-257. | Wrong publication type |
| 386. Sheila Menon, F. B. S. C. H. and V. Bhagat (2021). "Review of the impact Covid-19 has on the psychosocial factors affecting well-being." Research Journal of Pharmacy and Technology 14(6): 3404-3408. | Wrong publication type |
| 387. Silva, D. F. O., et al. (2021). "Prevalence of anxiety, depression, and stress among teachers during the COVID-19 pandemic a PRISMA-compliant systematic review." Medicine (United States) 100(44). | Wrong publication type |
| 388. Singh, S., et al. (2021). "Impact of COVID-19 Pandemic on Mental Health of General Population and University Students Across the World: A Review." Online Journal of Health & Allied Sciences 20(2): 1-8. | Wrong publication type |
| 389. Śniadach, J., et al. (2021). "Increased Depression and Anxiety Disorders during the COVID-19 Pandemic in Children and Adolescents: A Literature Review." Life (Basel) 11(11). | Wrong publication type |
| 390. Soto-Cámara, R., et al. (2021). "Psychological Impact of the COVID-19 Pandemic on Out-of-Hospital Health Professionals: A Living Systematic Review." J Clin Med 10(23). | Wrong publication type |
| 391. Strasser, M. A., et al. (2022). "COVID-19 news consumption and distress in young people: A systematic review." J Affect Disord 300: 481-491. | Wrong publication type |
| 392. Tashakori-Miyanroudi, M., et al. (2021). "Prevalence of depression, anxiety, and psychological distress in patients with epilepsy during COVID-19: A systematic review." EPILEPSY & BEHAVIOR 125. | Wrong publication type |
| 393. Thakur, B. and M. Pathak (2021). "Burden of Predominant Psychological Reactions Among the Healthcare Workers and General Population During COVID-19 Pandemic Phase: A Systematic Review and Meta-Analysis." INDIAN JOURNAL OF COMMUNITY MEDICINE 46(4): 600-605. | Wrong publication type |
| 394. Thatrimontrichai, A., et al. (2021). "Mental health among healthcare personnel during COVID-19 in Asia: A systematic review." Journal of the Formosan Medical Association 120(6): 1296-1304. | Wrong publication type |
| 395. Theberath, M., et al. (2022). "Effects of COVID-19 pandemic on mental health of children and adolescents: A systematic review of survey studies." SAGE Open Med 10: 20503121221086712. | Wrong publication type |
| 396. Tran, Q. D., et al. (2022). "Depression prevalence in Vietnam during the Covid-19 pandemic: A systematic review and meta-analysis." Ethics Med Public Health 23: 100806. | Wrong publication type |
| 397.Usmani, S., et al. (2021). "Risk Factors for Postpartum Depression During COVID-19 Pandemic: A Systematic Literature Review." Journal of Primary Care & Community Health 12: 21501327211059348. | Wrong publication type |
| 398. Vaca, K. M. R., et al. (2022). "Mental health of healthcare workers of Latin American countries: a review of studies published during the first year of COVID-19 pandemic." Psychiatry Research 311. | Wrong publication type |
| 399. Veazie, S., et al. (2022). "Mental health outcomes of adults hospitalized for COVID-19: A systematic review." J Affect Disord Rep 8: 100312. | Wrong publication type |
| 400. Vescovi, G., et al. (2021). "Parenting, mental health, and Covid-19: A rapid systematic review." Psicologia: Teoria e Prática 23(1): 1-28. | Wrong publication type |
| 401. Viner, R., et al. (2022). "School Closures During Social Lockdown and Mental Health, Health Behaviors, and Well-being Among Children and Adolescents During the First COVID-19 Wave: A Systematic Review." JAMA PEDIATRICS 176(4): 400-409. | Wrong publication type |
| 402. Wall, S. and M. Dempsey (2022). "The effect of COVID-19 lockdowns on women's perinatal mental health: a systematic review." Women Birth. | Wrong publication type |
| 403. Wirkner, J., et al. (2021). "Mental health in times of the COVID-19 pandemic: Current knowledge and implications from a European perspective." European Psychologist 26(4): 310-322. | Wrong publication type |
| 404. Wojtysiak, K. and H. Zielińska-Więczkowska (2022). "Work in stressful conditions in medical emergency system during the COVID-19 pandemic." Med Pr 73(3): 241-250. | Wrong publication type |
| 405. Wu, Y., et al. (2021). "International Students' Mental Health Care in China: A Systematic Review." Healthcare (Basel) 9(12). | Wrong publication type |
| 406. ya, A. and P. Lodha (2022). "Mental health consequences of COVID-19 pandemic among college students and coping approaches adapted by higher education institutions: A scoping review." SSM Ment Health 2: 100122. | Wrong publication type |
| 407. Yahiaoui, S., et al. (2022). "EPH28 Impact of COVID19 on Mental Health - Focus on Depression & Anxiety." Value in Health 25(7): S439. | Wrong publication type |
| 408. Yuan, K., et al. (2022). "A systematic review and meta-analysis on prevalence of and risk factors associated with depression, anxiety and insomnia in infectious diseases, including COVID-19: a call to action." Mol Psychiatry: 1-9. | Wrong publication type |
| 409. Zarghami, A., et al. (2022). "Psychological impacts of COVID-19 pandemic on individuals living with multiple sclerosis: A rapid systematic review." Mult Scler Relat Disord 59: 103562. | Wrong publication type |
| 410. Zeng, N., et al. "A systematic review and meta-analysis of long term physical and mental sequelae of COVID-19 pandemic: call for research priority and action." Molecular Psychiatry. | Wrong publication type |
| 411. Zhang, H., et al. (2022). "Psychological experience of COVID-19 patients: A systematic review and qualitative meta-synthesis." Am J Infect Control 50(7): 809-819. | Wrong publication type |
| 412. Xu, H., et al. (2021). "Psychosocial experiences of frontline nurses working in hospital-based settings during the COVID-19 pandemic - A qualitative systematic review." Int J Nurs Stud Adv 3: 100037. | Wrong publication type |
| 1. Cairns, P., et al., *Interventions for the well-being of healthcare workers during a pandemic or other crisis: scoping review.* BMJ Open. **11**(8): p. e047498. | Wrong Topic |
| 2. Callus, E., et al., *Stress Reduction Techniques for Health Care Providers Dealing With Severe Coronavirus Infections (SARS, MERS, and COVID-19): A Rapid Review.* FRONTIERS IN PSYCHOLOGY. **11**. | Wrong Topic |
| 3. Caponnetto, P., A. Benenati, and M.G. Maglia, *Psychopathological Impact and Resilient Scenarios in Inpatient with Schizophrenia Spectrum Disorders Related to Covid Physical Distancing Policies: A Systematic Review.* Behav Sci (Basel). **11**(4). | Wrong Topic |
| 4. Ceban, F., et al., *Association between Mood Disorders and Risk of COVID-19 Infection, Hospitalization, and Death: A Systematic Review and Meta-analysis.* JAMA Psychiatry, 2021. | Wrong Topic |
| 5. Choi, K.R., et al., *Adolescent Psychiatric Emergencies Precipitated by the COVID-19 Pandemic.* JOURNAL OF PSYCHOSOCIAL NURSING AND MENTAL HEALTH SERVICES. **59**(7): p. 17-21. | Wrong Topic |
| 6. Clemente-Suarez, V.J., et al., *Impact of COVID-19 Pandemic in Public Mental Health: An Extensive Narrative Review.* SUSTAINABILITY. **13**(6). | Wrong Topic |
| 7. Couturier, J., et al., *The COVID-19 pandemic and eating disorders in children, adolescents, and emerging adults: virtual care recommendations from the Canadian consensus panel during COVID-19 and beyond.* J Eat Disord. **9**(1): p. 46. | Wrong Topic |
| 8. Damiano, R.F., et al., *Mental health interventions following COVID-19 and other coronavirus infections: a systematic review of current recommendations and meta-analysis of randomized controlled trials.* Revista brasileira de psiquiatria (Sao Paulo, Brazil : 1999), 2021. | Wrong Topic |
| 9. David, E., et al., *COVID-19 Pandemic Support Programs for Healthcare Workers and Implications for Occupational Mental Health: A Narrative Review.* Psychiatr Q. | Wrong Topic |
| 10. Diaz, A., R. Baweja, and J.K. Bonatakis, *Global health disparities in vulnerable populations of psychiatric patients during the COVID-19 pandemic.* World J Psychiatry. **11**(4): p. 94-108. | Wrong Topic |
| 11. Ding, H., et al., *Effects of non-drug interventions on depression, anxiety and sleep in COVID-19 patients: A systematic review and meta-analysis.* European Review for Medical and Pharmacological Sciences, 2021. **25**(3): p. 1087-1096. | Wrong Topic |
| 12. Drissi, N., et al., *A Systematic Literature Review on e-Mental Health Solutions to Assist Health Care Workers During COVID-19.* Telemedicine journal and e-health : the official journal of the American Telemedicine Association, 2021. **27**(6): p. 594-602. | Wrong Topic |
| 13. Dube, J.P., et al., *Suicide behaviors during the COVID-19 pandemic: A meta-analysis of 54 studies.* PSYCHIATRY RESEARCH. **301**. | Wrong Topic |
| 14. Ellison, K.S., et al., *Telehealth and autism prior to and in the age of covid-19: A systematic and critical review of the last decade.* Clinical Child and Family Psychology Review, 2021. | Wrong Topic |
| 15. erlind, W.M., et al., *A systematic review of neuropsychological and psychiatric sequalae of COVID-19: implications for treatment.* Current Opinion in Psychiatry, 2021. **34**(4): p. 420-433. | Wrong Topic |
| 16. Fond, G., et al., *Association Between Mental Health Disorders and Mortality Among Patients With COVID-19 in 7 Countries A Systematic Review and Meta-analysis.* JAMA PSYCHIATRY. | Wrong Topic |
| 17. Fornaro, M., et al., *Implications of the COVID-19 pandemic for people with bipolar disorders: A scoping review.* Journal of Affective Disorders, 2021. **295**: p. 740-751. | Wrong Topic |
| 18. Havsteen-Franklin, D., et al., *Arts-Based Interventions for Professionals in Caring Roles During and After Crisis: A Systematic Review of the Literature.* FRONTIERS IN PSYCHOLOGY. **11**. | Wrong Topic |
| 19. Hossain, M.I., et al., *Psychosocial stress and trauma during the COVID-19 pandemic: Evidence from Bangladesh.* ASIAN SOCIAL WORK AND POLICY REVIEW. **15**(2): p. 145-159. | Wrong Topic |
| 20. John, A., et al., *The impact of the COVID-19 pandemic on self-harm and suicidal behaviour: A living systematic review.* F1000Research, 2020. **9**. | Wrong Topic |
| 21. Kahil, K., et al., *Suicide during COVID-19 and other major international respiratory outbreaks: A systematic review.* Asian Journal of Psychiatry, 2021. **56**. | Wrong Topic |
| 22. Karaoulanis, S.E. and N.G. Christodoulou, *Do patients with schizophrenia have higher infection and mortality rates due to COVID-19? A systematic review.* Psychiatrike = Psychiatriki, 2021. | Wrong Topic |
| 23. Leahy, D. and F. McNicholas, *Systematic review of effectiveness and satisfaction evaluation in child and adolescent mental health services in Ireland.* Irish Journal of Psychological Medicine, 2021. | Wrong Topic |
| 24. Lemieux, A.J., et al., *Management of COVID-19 for Persons with Mental Illness in Secure Units: A Rapid International Review to Inform Practice in Quebec.* VICTIMS & OFFENDERS. **15**(7): p. 1337-1360. | Wrong Topic |
| 25. Mamun, M.A., *Suicide and Suicidal Behaviors in the Context of COVID-19 Pandemic in Bangladesh: A Systematic Review.* Psychol Res Behav Manag, 2021. **14**: p. 695-704. | Wrong Topic |
| 26. Marra, D.E., et al., *Validity of teleneuropsychology for older adults in response to COVID-19: A systematic and critical review.* Clin Neuropsychol. **34**(7): p. 1411-1452. | Wrong Topic |
| 27. McLean, S.A., et al., *Exploring the efficacy of telehealth for family therapy through systematic, meta-analytic, and qualitative evidence.* Clinical Child and Family Psychology Review, 2021. | Wrong Topic |
| 28. Moreira, W.C., et al., *Mental health interventions implemented in the COVID-19 pandemic: what is the evidence?* Rev Bras Enferm, 2021. **74**: p. e20200635. | Wrong Topic |
| 29. Moss, S.J., et al., *Restricted visitation policies in acute care settings during the COVID-19 pandemic: a scoping review.* Critical Care, 2021. **25**(1): p. 1-12. | Wrong Topic |
| 30. Noone, C., et al., *Video calls for reducing social isolation and loneliness in older people: a rapid review.* Cochrane Database of Systematic Reviews, 2020(5). | Wrong Topic |
| 31. Orsolini, L., et al., *A Systematic Review on TeleMental Health in Youth Mental Health: Focus on Anxiety, Depression and Obsessive-Compulsive Disorder.* Medicina (Kaunas). **57**(8). | Wrong Topic |
| 32. Ostuzzi, G., et al., *Pharmacological treatment of hyperactive delirium in people with COVID-19: rethinking conventional approaches.* Therapeutic Advances in Psychopharmacology, 2020. **10**. | Wrong Topic |
| 33. Paz, L.E.S., et al., *COVID-19: the importance of physical therapy in the recovery of workers' health.* Rev Bras Med Trab. **19**(1): p. 94-106. | Wrong Topic |
| 34. Pilowsky, J.K., R. Elliott, and M.A. Roche, *Pre-existing mental health disorders in patients admitted to the intensive care unit: A systematic review and meta-analysis of prevalence.* J Adv Nurs. **77**(5): p. 2214-2227. | Wrong Topic |
| 35. Porter, B., et al., *The impact of providing end-of-life care during a pandemic on the mental health and wellbeing of health and social care staff: Systematic review and meta-synthesis.* Social Science & Medicine, 2021. **287**: p. N.PAG-N.PAG. | Wrong Topic |
| 36. Puyat, J.H., et al., *A rapid review of home-based activities that can promote mental wellness during the COVID-19 pandemic.* PLoS ONE, 2020. **15**(12). | Wrong Topic |
| 37. Rauschenberg, C., et al., *Evidence Synthesis of Digital Interventions to Mitigate the Negative Impact of the COVID-19 Pandemic on Public Mental Health: Rapid Meta-review.* Journal of Medical Internet Research, 2021. **23**(3): p. N.PAG-N.PAG. | Wrong Topic |
| 38. Rogers, J.P., et al., *Suicide, self-harm and thoughts of suicide or self-harm in infectious disease epidemics: A systematic review and meta-analysis.* Epidemiology and Psychiatric Sciences, 2021. | Wrong Topic |
| 39. Sabe, M., et al., *Toxicity of psychotropic drugs in patients with COVID-19: A systematic review.* General Hospital Psychiatry, 2021. **70**: p. 1-9. | Wrong Topic |
| 40. Safieh, J., et al., *Interventions to Optimise Mental Health Outcomes During the COVID-19 Pandemic: A Scoping Review.* INTERNATIONAL JOURNAL OF MENTAL HEALTH AND ADDICTION. | Wrong Topic |
| 41. Santos de Sousa Júnior, B., et al., *PANDEMIA DO CORONAVÍRUS: ESTRATÉGIAS AMENIZADORAS DO ESTRESSE OCUPACIONAL EM TRABALHADORES DA SAÚDE.* Enfermagem em Foco, 2020. **11**: p. 148-154. | Wrong Topic |
| 42. Sharifi, V. and A. Mohammadjafari, *Screening for mental health problems in epidemics: Is it justifiable?* Archives of Iranian Medicine, 2021. **24**(8): p. 643-650. | Wrong Topic |
| 43. Sharma, M., et al., *Impact of COVID-19 pandemic on sleep in children and adolescents: a systematic review and meta-analysis.* Sleep Medicine, 2021. **84**: p. 259-267. | Wrong Topic |
| 44. Shatri, H., et al., *Psychotherapy for Healthcare Provider During COVID-19 Pandemic: An Evidence Based Clinical Review.* Acta medica Indonesiana, 2021. **53**(2): p. 225-232. | Wrong Topic |
| 45. Siegel, A., et al., *Barriers, benefits and interventions for improving the delivery of telemental health services during the coronavirus disease 2019 pandemic: a systematic review.* Current Opinion in Psychiatry, 2021. **34**(4): p. 434-443. | Wrong Topic |
| 46. Simon, N., et al., *Internet‐based cognitive and behavioural therapies for post‐traumatic stress disorder (PTSD) in adults.* Cochrane Database of Systematic Reviews, 2021(5). | Wrong Topic |
| 47. Simonelli, G., et al., *Sleep in times of crises: A scoping review in the early days of the COVID-19 crisis.* Sleep Medicine Reviews, 2021. **60**. | Wrong Topic |
| 48. Simonetti, A., et al., *Neuropsychiatric Symptoms in Elderly With Dementia During COVID-19 Pandemic: Definition, Treatment, and Future Directions.* Frontiers in Psychiatry, 2020. **11**. | Wrong Topic |
| 49. Smith, V., et al., *Care bundles for improving outcomes in patients with COVID‐19 or related conditions in intensive care – a rapid scoping review.* Cochrane Database of Systematic Reviews, 2020(12). | Wrong Topic |
| 50. Soklaridis, S., et al., *Mental health interventions and supports during COVID- 19 and other medical pandemics: A rapid systematic review of the evidence.* General Hospital Psychiatry, 2020. **66**: p. 133-146. | Wrong Topic |
| 51. Soltany, A., et al., *A scoping review of the impact of COVID-19 pandemic on surgical practice.* Annals of Medicine and Surgery, 2020. **57**: p. 24-36. | Wrong Topic |
| 52. Stockwell, S., et al., *Changes in physical activity and sedentary behaviours from before to during the COVID-19 pandemic lockdown: a systematic review.* BMJ Open Sport Exerc Med, 2021. **7**(1): p. e000960. | Wrong Topic |
| 53. Strudwick, G., et al., *Digital interventions to support population mental health in canada during the covid-19 pandemic: Rapid review.* JMIR Mental Health, 2021. **8**(3). | Wrong Topic |
| 54. Suárez-González, A., et al., *The effect of COVID-19 isolation measures on the cognition and mental health of people living with dementia: A rapid systematic review of one year of quantitative evidence.* EClinicalMedicine, 2021. **39**. | Wrong Topic |
| 55. Suleiman-Martos, N., et al., *Prevalence and Predictors of Burnout in Midwives: A Systematic Review and Meta-Analysis.* Int J Environ Res Public Health. **17**(2). | Wrong Topic |
| 56. Sun, X., et al., *Effect of intensive care unit diary on incidence of posttraumatic stress disorder, anxiety, and depression of adult intensive care unit survivors: A systematic review and meta-analysis.* J Adv Nurs. **77**(7): p. 2929-2941. | Wrong Topic |
| 57. Sun, Z., et al., *Psychological Interventions for Healthcare Providers With PTSD in Life-Threatening Pandemic: Systematic Review and Meta-Analysis.* Frontiers in Psychiatry, 2021. **12**. | Wrong Topic |
| 58. Suresh, R., A. Alam, and Z. Karkossa, *Using Peer Support to Strengthen Mental Health During the COVID-19 Pandemic: A Review.* Frontiers in Psychiatry, 2021. **12**. | Wrong Topic |
| 59. Taylor, C.B., et al., *Current state of scientific evidence on Internet-based interventions for the treatment of depression, anxiety, eating disorders and substance abuse: an overview of systematic reviews and meta-analyses.* Eur J Public Health. **31**(31): p. i3-i10. | Wrong Topic |
| 60. Thomas, N., et al., *Review of the current empirical literature on using videoconferencing to deliver individual psychotherapies to adults with mental health problems.* Psychology & Psychotherapy: Theory, Research & Practice, 2021. **94**(3): p. 854-883. | Wrong Topic |
| 61. Toubasi, A.A., et al., *A meta-analysis: The mortality and severity of COVID-19 among patients with mental disorders.* Psychiatry Research, 2021. **299**. | Wrong Topic |
| 62. Treacy, S., et al., *Repurposing psychological interventions for healthcare workers during COVID-19.* BMC Proceedings, 2021. **15**. | Wrong Topic |
| 63. Treanor, C.J., et al., *Acceptability of Computerized Cognitive Behavioral Therapy for Adults: Umbrella Review.* JMIR Ment Health. **8**(7): p. e23091. | Wrong Topic |
| 64. Ueafuea, K., et al., *Potential Applications of Mobile and Wearable Devices for Psychological Support During the COVID-19 Pandemic: A Review.* IEEE SENSORS JOURNAL. **21**(6): p. 7162-7178. | Wrong Topic |
| 65. Vai, B., et al., *Mental disorders and risk of COVID-19-related mortality, hospitalisation, and intensive care unit admission: a systematic review and meta-analysis.* LANCET PSYCHIATRY. **8**(9): p. 797-812. | Wrong Topic |
| 66. Vaishali, K., et al., *Coping strategies for obese individuals with obstructive sleep apnea during COVID-19 pandemic: A narrative review.* Obesity Medicine, 2021. **22**. | Wrong Topic |
| 67. van Veenendaal, N.R., et al., *Supporting parents as essential care partners in neonatal units during the SARS-CoV-2 pandemic.* Acta Paediatrica, International Journal of Paediatrics, 2021. **110**(7): p. 2008-2022. | Wrong Topic |
| 68. Velana, M. and G. Rinkenauer, *Individual-Level Interventions for Decreasing Job-Related Stress and Enhancing Coping Strategies Among Nurses: A Systematic Review.* Front Psychol, 2021. **12**: p. 708696. | Wrong Topic |
| 69. Vigo, D., et al., *Estimating the Prevalence of Mental and Substance Use Disorders: A Systematic Approach to Triangulating Available Data to Inform Health Systems Planning:: Estimer la prévalence des troubles mentaux et des troubles liés à une substance: une approche systématique de la triangulation des données disponibles pour éclairer la planification du système de santé.* Can J Psychiatry: p. 7067437211006872. | Wrong Topic |
| 70. Violant-Holz, V., et al., *Psychological Health and Physical Activity Levels during the COVID-19 Pandemic: A Systematic Review.* INTERNATIONAL JOURNAL OF ENVIRONMENTAL RESEARCH AND PUBLIC HEALTH. **17**(24). | Wrong Topic |
| 71. Vitger, T., et al., *Digital Shared Decision-Making Interventions in Mental Healthcare: A Systematic Review and Meta-Analysis.* Front Psychiatry, 2021. **12**: p. 691251. | Wrong Topic |
| 72. von Hagen, A., et al., *Efficacy of Remote as Compared to In-Person School Psychological Services: A Rapid Systematic Evidence Review.* JOURNAL OF EDUCATIONAL AND PSYCHOLOGICAL CONSULTATION. | Wrong Topic |
| 73. Wang, L., et al., *Clinical manifestations and evidence of neurological involvement in 2019 novel coronavirus SARS-CoV-2: a systematic review and meta-analysis.* Journal of Neurology, 2020. **267**(10): p. 2777-2789. | Wrong Topic |
| 74. Waring, S. and S. Giles, *Rapid Evidence Assessment of Mental Health Outcomes of Pandemics for Health Care Workers: Implications for the Covid-19 Pandemic.* Front Public Health, 2021. **9**: p. 629236. | Wrong Topic |
| 75. Waseem, S., et al., *The global burden of trauma during the COVID-19 pandemic: A scoping review.* Journal of Clinical Orthopaedics and Trauma, 2021. **12**(1): p. 200-207. | Wrong Topic |
| 76. Williams, C.Y.K., et al., *Interventions to reduce social isolation and loneliness during COVID-19 physical distancing measures: A rapid systematic review.* PLoS ONE, 2021. **16**(2). | Wrong Topic |
| 77. Wingert, A., et al., *Risk factors for severity of COVID-19: A rapid review to inform vaccine prioritisation in Canada.* BMJ Open, 2021. **11**(5). | Wrong Topic |
| 78. Wolf, S., et al., *Is Physical Activity Associated with Less Depression and Anxiety During the COVID-19 Pandemic? A Rapid Systematic Review.* Sports Medicine, 2021. **51**(8): p. 1771-1783. | Wrong Topic |
| 79. Xia, L., et al., *Prevalence of Sleep Disturbances and Sleep Quality in Chinese Healthcare Workers During the COVID-19 Pandemic: A Systematic Review and Meta-Analysis.* Frontiers in Psychiatry, 2021. **12**. | Wrong Topic |
| 80. Yang, Y., et al., *Comparative Effectiveness of Multiple Psychological Interventions for Psychological Crisis in People Affected by Coronavirus Disease 2019: A Bayesian Network Meta-Analysis.* FRONTIERS IN PSYCHOLOGY. **12**. | Wrong Topic |
| 81. Yen-Hao Chu, I., et al., *Social consequences of mass quarantine during epidemics: A systematic review with implications for the COVID-19 response.* Journal of Travel Medicine, 2020. **27**(7). | Wrong Topic |
| 82. Yong, S.J., *Long COVID or post-COVID-19 syndrome: putative pathophysiology, risk factors, and treatments.* Infectious Diseases, 2021. **53**(10): p. 737-754. | Wrong Topic |
| 83. Yue, J.-L., et al., *Mental health services for infectious disease outbreaks including COVID-19: a rapid systematic review.* Psychological Medicine, 2020. **50**(15): p. 2498-2513. | Wrong Topic |
| 84. Zaccari, V., et al., *Narrative Review of COVID-19 Impact on Obsessive-Compulsive Disorder in Child, Adolescent and Adult Clinical Populations.* Frontiers in Psychiatry, 2021. **12**. | Wrong Topic |
| 85. Zaçe, D., et al., *Interventions to address mental health issues in healthcare workers during infectious disease outbreaks: A systematic review.* Journal of Psychiatric Research, 2021. **136**: p. 319-333. | Wrong Topic |
| 86. Zapata-Ospina, J.P., et al., *Mental health interventions for college and university students during the COVID-19 pandemic: a critical synthesis of the literature.* Revista Colombiana de Psiquiatria, 2021. **50**(3): p. 199-213. | Wrong Topic |
| 87. Zhang, L., et al., *[An analysis of global research on SARS-CoV-2].* Sheng Wu Yi Xue Gong Cheng Xue Za Zhi. **37**(2): p. 236-245. | Wrong Topic |
| 88. Zhang, M. and H.E. Smith, *Digital Tools to Ameliorate Psychological Symptoms Associated With COVID-19: Scoping Review.* Journal of Medical Internet Research, 2020. **22**(8): p. N.PAG-N.PAG. | Wrong Topic |
| 89. Zortea, T.C., et al., *The Impact of Infectious Disease-Related Public Health Emergencies on Suicide, Suicidal Behavior, and Suicidal Thoughts.* Crisis: The Journal of Crisis Intervention & Suicide Prevention, 2020: p. 1-14. | Wrong Topic |
| 1. Kuroda, N. and T. Kubota, *Psychological impact of the COVID-19 pandemic for patients with epilepsy: A systematic review and meta-analysis.* Epilepsy Behav. **124**: p. 108340. | Not retrievable |
| 1. Danet, A.D., *Impacto psicol?gico de la COVID-19 en profesionales sanitarios de primera l?nea en el ?mbito occidental. Una revisi?n sistem?tica.* MEDICINA CLINICA. **156**(9): p. 449-458. | Duplicate |
| 2. Muehlschlegel, P.A., et al., *Learning from previous lockdown measures and minimising harmful biopsychosocial consequences as they end: A systematic review.* JOURNAL OF GLOBAL HEALTH, 2021. **11**. | Duplicate |
| 3. Röhr, S., et al., *Psychosoziale Folgen von Quarantänemaßnahmen bei schwerwiegenden Coronavirus-Ausbrüchen: ein Rapid Review = Psychosocial impact of quarantine measures during serious coronavirus outbreaks: A rapid review.* Psychiatrische Praxis, 2020. **47**(4): p. 179-189. | Duplicate |
| 4. Zhang, S. X. and J. Chen (2021). "Scientific evidence on mental health in key regions under the COVID-19 pandemic - meta-analytical evidence from Africa, Asia, China, Eastern Europe, Latin America, South Asia, Southeast Asia, and Spain." Eur J Psychotraumatol 12(1): 2001192. | Duplicate |
| 1. Monteleone, A. M. and Cascino, G. and Barone, E. and Carfagno, M. and Monteleone, P. COVID-19 Pandemic and Eating Disorders: What Can We Learn About Psychopathology and Treatment? A Systematic Review 2021 | Wrong population |
| 2. Radfar, Amir and Caceres, Maria Mercedes Ferreira and Sosa, Juan Pablo and Filip, Irina Overcoming the Challenges of the Mental Health Care System in United States in the Aftermath of COVID-19 2021 | Wrong population |
| 1. Liu, X. and Wang, G. and Zhang, J. and Wang, S. Prevalence of depression and anxiety among health care workers in designated hospitals during the COVID-19 epidemic: A meta-analysis 2021 | Foreign language |
| 1. Al Maqbali, M., M. Al Sinani, and B. Al-Lenjawi, *Prevalence of stress, depression, anxiety and sleep disturbance among nurses during the COVID-19 pandemic: A systematic review and meta-analysis.* Journal of Psychosomatic Research, 2021. **141**: p. N.PAG-N.PAG. | E-publication 2020 |
| 2. Arora, T., et al., *The prevalence of psychological consequences of COVID-19: A systematic review and meta-analysis of observational studies.* JOURNAL OF HEALTH PSYCHOLOGY. | E-publication 2020 |
| 3. Batra, K., et al., *Investigating the psychological impact of COVID-19 among healthcare workers: A meta-analysis.* International Journal of Environmental Research and Public Health, 2020. **17**(23): p. 1-33. | E-publication 2020 |
| 4. Bueno-Notivol, J., et al., *Prevalence of depression during the COVID-19 outbreak: A meta-analysis of community-based studies.* INTERNATIONAL JOURNAL OF CLINICAL AND HEALTH PSYCHOLOGY. **21**(1). | E-publication 2020 |
| 5. Cénat, J.M., et al., *Prevalence of symptoms of depression, anxiety, insomnia, posttraumatic stress disorder, and psychological distress among populations affected by the COVID-19 pandemic: A systematic review and meta-analysis.* Psychiatry Res. **295**: p. 113599. | E-publication 2020 |
| 6. Cooke, J.E., et al., *Prevalence of posttraumatic and general psychological stress during COVID-19: A rapid review and meta-analysis.* Psychiatry Research, 2020. **292**. | E-publication 2020 |
| 7. da Silva, F.C.T. and M.L.R. Neto, *Psychiatric symptomatology associated with depression, anxiety, distress, and insomnia in health professionals working in patients affected by COVID-19: A systematic review with meta-analysis.* Progress in Neuro-Psychopharmacology and Biological Psychiatry, 2021. **104**. | E-publication 2020 |
| 8. da Silva, F.C.T. and M.L.R. Neto, *Psychological effects caused by the COVID-19 pandemic in health professionals: A systematic review with meta-analysis.* Progress in Neuro-Psychopharmacology and Biological Psychiatry, 2021. **104**. | E-publication 2020 |
| 9. de Pablo, G.S., et al., *Impact of coronavirus syndromes on physical and mental health of health care workers: Systematic review and meta-analysis.* JOURNAL OF AFFECTIVE DISORDERS. **275**: p. 48-57. | E-publication 2020 |
| 10. Deng, J., et al., *The prevalence of depression, anxiety, and sleep disturbances in COVID-19 patients: a meta-analysis*. 2021. p. 90-111. | E-publication 2020 |
| 11. Fan, S., et al., *Psychological effects caused by COVID-19 pandemic on pregnant women: A systematic review with meta-analysis.* ASIAN JOURNAL OF PSYCHIATRY. **56**. | E-publication 2020 |
| 12. Hessami, K., et al., *COVID-19 pandemic and maternal mental health: a systematic review and meta-analysis.* JOURNAL OF MATERNAL-FETAL & NEONATAL MEDICINE. | E-publication 2020 |
| 13. Krishnamoorthy, Y., et al., *Prevalence of psychological morbidities among general population, healthcare workers and COVID-19 patients amidst the COVID-19 pandemic: A systematic review and meta-analysis.* Psychiatry Research, 2020. **293**. | E-publication 2020 |
| 14. Lasheras, I., et al., *Prevalence of anxiety in medical students during the covid-19 pandemic: A rapid systematic review with meta-analysis.* International Journal of Environmental Research and Public Health, 2020. **17**(18): p. 1-12. | E-publication 2020 |
| 15. Luo, M., et al., *The psychological and mental impact of coronavirus disease 2019 (COVID-19) on medical staff and general public—A systematic review and meta-analysis.* Psychiatry Research, 2020. **291**. | E-publication 2020 |
| 16. Neelam, K., et al., *Pandemics and pre-existing mental illness: A systematic review and meta-analysis.* Brain, Behavior, and Immunity - Health, 2021. **10**. | E-publication 2020 |
| 17. Pan, R., L. Zhang, and J. Pan, *The anxiety status of chinese medical workers during the epidemic of COVID-19: A meta-analysis.* Psychiatry Investigation, 2020. **17**(5): p. 475-480. | E-publication 2020 |
| 18. Pappa, S., et al., *Prevalence of depression, anxiety, and insomnia among healthcare workers during the COVID-19 pandemic: A systematic review and meta-analysis.* Brain, Behavior, and Immunity, 2020. **88**: p. 901-907. | E-publication 2020 |
| 19. Ren, X., et al., *Mental Health During the Covid-19 Outbreak in China: a Meta-Analysis.* Psychiatric Quarterly, 2020. **91**(4): p. 1033-1045. | E-publication 2020 |
| 20. Rogers, J.P., et al., *Psychiatric and neuropsychiatric presentations associated with severe coronavirus infections: a systematic review and meta-analysis with comparison to the COVID-19 pandemic.* The Lancet Psychiatry, 2020. **7**(7): p. 611-627. | E-publication 2020 |
| 21. Salari, N., et al., *Prevalence of stress, anxiety, depression among the general population during the COVID-19 pandemic: a systematic review and meta-analysis.* Globalization & Health, 2020. **16**(1): p. 1-11. | E-publication 2020 |
| 22. Salari, N., et al., *The prevalence of stress, anxiety and depression within front-line healthcare workers caring for COVID-19 patients: a systematic review and meta-regression.* Human Resources for Health, 2020. **18**(1): p. 1-14. | E-publication 2020 |
| 23. Salazar de Pablo, G., et al., *Impact of coronavirus syndromes on physical and mental health of health care workers: Systematic review and meta-analysis.* Journal of Affective Disorders, 2020. **275**: p. 48-57. | E-publication 2020 |
| 24. Santabárbara, J., et al., *Prevalence of anxiety in the COVID-19 pandemic: An updated meta-analysis of community-based studies.* Progress in Neuro-Psychopharmacology and Biological Psychiatry, 2021. **109**. | E-publication 2020 |
| 25. Serrano-Ripoll, M.J., et al., *Impact of viral epidemic outbreaks on mental health of healthcare workers: a rapid systematic review and meta-analysis.* JOURNAL OF AFFECTIVE DISORDERS. **277**: p. 347-357. | E-publication 2020 |
| 26. Sheraton, M., et al., *Psychological effects of the COVID 19 pandemic on healthcare workers globally: A systematic review.* Psychiatry Research, 2020. **292**. | E-publication 2020 |
| 27. Silva, D.F.O., et al., *Prevalence of anxiety among health professionals in times of COVID-19: a systematic review with meta-analysis.* CIENCIA & SAUDE COLETIVA. **26**(2): p. 693-710. | E-publication 2020 |
| 28. Sun, F., et al., *A systematic review involving 11,187 participants evaluating the impact of covid-19 on anxiety and depression in pregnant women.* Journal of Psychosomatic Obstetrics & Gynecology, 2020. | E-publication 2020 |
| 29. Tng, X.J.J., Q.H. Chew, and K. Sim, *Psychological sequelae within different populations during the COVID-19 pandemic: a rapid review of extant evidence.* Singapore medical journal, 2020. | E-publication 2020 |
| 30. Vindegaard, N. and M.E. Benros, *COVID-19 pandemic and mental health consequences: Systematic review of the current evidence.* Brain, Behavior, and Immunity, 2020. **89**: p. 531-542. | E-publication 2020 |
| 31. Vizheh, M., et al., *The mental health of healthcare workers in the COVID-19 pandemic: A systematic review.* Journal of Diabetes and Metabolic Disorders, 2020. **19**(2): p. 1967-1978. | E-publication 2020 |
| 32. Wang, Y., M.P. Kala, and T.H. Jafar, *Factors associated with psychological distress during the coronavirus disease 2019 (COVID- 19) pandemic on the predominantly general population: A systematic review and metaanalysis.* PLoS ONE, 2020. **15**(12). | E-publication 2020 |
| 33. Wu, T.C., et al., *Prevalence of mental health problems during the COVID-19 pandemic: A systematic review and meta-analysis.* JOURNAL OF AFFECTIVE DISORDERS. **281**: p. 91-98. | E-publication 2020 |
| 34. Yan, H., Y. Ding, and W. Guo, *Mental Health of Pregnant and Postpartum Women During the Coronavirus Disease 2019 Pandemic: A Systematic Review and Meta-Analysis.* Front Psychol, 2020. **11**: p. 617001. | E-publication 2020 |
| 35. Al Maqbali M, Al Sinani M, Al-Lenjawi B. Prevalence of stress, depression, anxiety and sleep disturbance among nurses during the COVID-19 pandemic: A systematic review and meta-analysis. J Psychosom Res. 2021 Feb;141:110343. doi: 10.1016/j.jpsychores.2020.110343. Epub 2020 Dec 17. PMID: 33360329; PMCID: PMC7831768. | E-publication 2020 |
| 36. Bueno-Notivol J, Gracia-García P, Olaya B, Lasheras I, López-Antón R, Santabárbara J. Prevalence of depression during the COVID-19 outbreak: A meta-analysis of community-based studies. Int J Clin Health Psychol. 2021 Jan-Apr;21(1):100196. doi: 10.1016/j.ijchp.2020.07.007. Epub 2020 Aug 31. PMID: 32904715; PMCID: PMC7458054. | E-publication 2020 |
| 37. Santabárbara, J., et al. (2021). "Prevalence of anxiety in the COVID-19 pandemic: An updated meta-analysis of community-based studies." PROGRESS IN NEURO-PSYCHOPHARMACOLOGY & BIOLOGICAL PSYCHIATRY 109. | E-publication 2020 |
| 38. Silva, D. F. O., et al. (2021). "Prevalence of anxiety among health professionals in times of COVID-19: A systematic review with meta-analysis." Ciencia e Saude Coletiva 26(2): 693-710. | E-publication 2020 |
| 39. Sun, F., et al. (2021). "A systematic review involving 11,187 participants evaluating the impact of COVID-19 on anxiety and depression in pregnant women." Journal of Psychosomatic Obstetrics & Gynecology 42(2): 91-99. | E-publication 2020 |
| 40. Wu, T., et al. (2021). "Prevalence of mental health problems during the COVID-19 pandemic: A systematic review and meta-analysis." JOURNAL OF AFFECTIVE DISORDERS 281: 91-98. | E-publication 2020 |

### **Table C**. Appendix AMSTAR 2 ratings

| **AMSTAR item:** | **1** | **2*** | **3** | **4*** | **5** | **6** | **7*** | **8** | **9*** | **10** | **11*** | **12** | **13*** | **14** | **15*** | **16** | **Critical** | **Total** |
| --- | --- | --- | --- | --- | --- | --- | --- | --- | --- | --- | --- | --- | --- | --- | --- | --- | --- | --- |
| Abdulla 2021 (1) | PY | Y | Y | PY | N | N | N | PY | Y | N | Y | Y | Y | Y | N | Y | 3,5 | 9,5 |
| Adrianto 2022 (2) | Y | N | Y | PY | Y | Y | N | PY | N | N | Y | N | N | Y | Y | Y | 2,5 | 9 |
| Adibi 2021 (3) | Y | PY | N | PY | Y | Y | N | N | N | N | N | Y | Y | N | Y | Y | 3 | 8 |
| Afridi 2022 (4) | PY | N | N | PY | Y | Y | N | PY | Y | N | Y | N | N | Y | N | Y | 2,5 | 7,5 |
| Alzahrani 2021 (5) | Y | N | Y | PY | N | N | N | PY | N | N | Y | N | N | N | Y | Y | 2,5 | 6 |
| Arora, 2022 (6) | Y | N | Y | PY | Y | Y | N | PY | N | N | Y | N | N | Y | N | N | 1,5 | 7 |
| Aymerich, 2021 (7) | Y | Y | Y | PY | Y | Y | Y | PY | Y | N | Y | N | N | Y | N | Y | 4,5 | 11 |
| Ayubi 2021 (8) | Y | N | N | PY | N | N | N | PY | N | N | Y | N | N | N | N | Y | 0,5 | 4,5 |
| Bello 2022 (9) | Y | Y | Y | PY | Y | Y | N | N | N | N | Y | N | N | N | N | Y | 2,5 | 7,5 |
| Balakrishnan 2022 (10) | Y | Y | Y | PY | N | N | N | PY | Y | N | Y | N | N | Y | N | Y | 3,5 | 8 |
| Bareeqa 2021 (11) | Y | PY | N | Y | Y | Y | N | PY | PY | N | Y | N | N | Y | N | Y | 3 | 8,5 |
| Batra 2021 (12) | Y | PY | N | N | Y | Y | N | N | PY | N | Y | N* | Y | Y | Y | Y | 5 | 9 |
| Bussières 2021 (13) | PY | N | Y | PY | N | N | N | PY | N | N | Y | N | N | N | Y | Y | 2,5 | 5,5 |
| Carvalho 2022 (14) | Y | N | N | N | N | N | N | N | N | N | Y | N | N | Y | Y | Y | 2 | 5 |
| Castaldelli-Maia 2021 (15) | Y | PY | N | PY | Y | Y | N | Y | PY | N | Y | N | N | Y | N | Y | 2,5 | 8,5 |
| Cenat 2021 (16) | Y | Y | Y | N | Y | N | N | PY | N | N | Y | N | N | Y | Y | Y | 3 | 8,5 |
| Cenat 2022 (17) | Y | Y | Y | PY | Y | Y | N | PY | Y | N | Y | Y | Y | Y | N | Y | 4,5 | 11 |
| Cevik 2022 (18) | Y | Y | Y | PY | Y | Y | N | PY | N | N | Y | N | N | N | N | Y | 2,5 | 8 |
| Chai 2021 (19) | Y | Y | Y | PY | N | N | N | PY | N | N | Y | N | N | Y | N | Y | 2,5 | 7 |
| Chang 2021 (20) | Y | N | N | N | Y | Y | N | PY | PY | N | Y | N* | N | Y | Y | Y | 2,5 | 8 |
| Chekole and Abate 2021 (21) | Y | PY | Y | N | Y | Y | N | Y | PY | N | Y | N | Y | Y | Y | Y | 4 | 11 |
| Chen Farah 2021 (22) | Y | Y | Y | PY | Y | Y | N | N | N | N | Y | N | N | N | N | Y | 2 | 7,5 |
| Chen Yang 2022 (23) | PY | N | N | PY | Y | Y | N | PY | N | N | Y | N | N | Y | Y | Y | 2,5 | 7,5 |
| Chen Li 2022 (24) | PY | N | Y | PY | Y | Y | N | PY | N | N | Y | N | N | N | N | Y | 1,5 | 6,5 |
| Ching 2021 (25) | Y | Y | Y | PY | Y | Y | N | N | Y | N | Y | N | N | Y | Y | Y | 4,5 | 10,5 |
| Dal Santo 2021 (26) | Y | Y | Y | Y | Y | Y | Y | Y | PY | N | Y | N | N | N | N | Y | 4,5 | 10,5 |
| da Silva 2021 (27) | PY | N | Y | PY | N | N | N | PY | N | N | Y | N | N | N | N | Y | 1,5 | 4,5 |
| Demissie 2021 (28) | Y | N | Y | PY | N | Y | N | PY | PY | N | Y | Y | N | Y | Y | Y | 3 | 9,5 |
| Deng 2021 (29) | Y | Y | Y | PY | Y | Y | N | PY | PY | N | Y | Y | Y | Y | Y | Y | 5 | 12,5 |
| Deng 2021 (30) | Y | Y | Y | Y | Y | Y | N | PY | PY | N | Y | N | N | Y | Y | Y | 4,5 | 11 |
| de Sousa 2021 (31) | Y | Y | Y | PY | N | N | N | PY | N | N | Y | N | N | N | Y | Y | 3,5 | 7 |
| Delanerolle 2022 (32) | Y | Y | Y | PY | N | N | N | PY | Y | N | Y | N | N | N | Y | Y | 4,5 | 8 |
| Dong 2021 (33) | Y | Y | Y | N | Y | Y | N | Y | PY | N | N | N | N | Y | Y | Y | 2,5 | 9,5 |
| Dong 2021 (34) | Y | PY | Y | PY | Y | Y | N | Y | PY | N | N | N | N | N | Y | Y | 2,5 | 10 |
| Dragioti 2022 (35) | PY | Y | Y | PY | Y | Y | N | PY | N | N | Y | N | N | Y | Y | N | 3,5 | 8,5 |
| Dutta 2021 (36) | Y | Y | Y | PY | Y | Y | N | PY | PY | N | Y | N | N | Y | N | Y | 3 | 9,5 |
| Ebrahim 2022 (37) | Y | N | Y | PY | Y | Y | N | PY | Y | N | Y | N | N | Y | Y | Y | 3,5 | 10 |
| El-Qushayri 2021 (38) | Y | N | N | Y | Y | N | N | PY | PY | N | Y | N | N | Y | N | Y | 2,5 | 7 |
| Fan 2021 (39) | Y | N | N | N | N | N | N | N | PY | N | N | N | N | N | Y | Y | 1,5 | 3,5 |
| Fang Ji 2022 (40) | Y | PY | N | PY | Y | Y | N | Y | PY | N | Y | N | Y | Y | Y | Y | 6 | 12 |
| Ghahramani 2022 (41) | Y | PY | N | N | Y | N | N | Y | PY | N | Y | Y | Y | Y | N | Y | 4 | 10 |
| Ghazanfarpour 2021 (42) | Y | N | N | PY | N | Y | N | Y | Y | N | Y | N | N | N | Y | Y | 3,5 | 7,5 |
| Guo 2021 (43) | Y | N | N | N | N | Y | N | PY | PY | N | Y | N | N | Y | Y | Y | 2,5 | 7 |
| Halemani 2021 (44) | Y | PY | N | N | Y | Y | N | PY | PY | N | Y | Y | Y | N | Y | N | 5 | 10 |
| Hao 2021 (45) | Y | Y | N | Y | Y | Y | N | Y | PY | N | Y | N | N | Y | Y | Y | 4 | 10,5 |
| Hosen 2021 (46) | Y | N | N | N | N | Y | N | Y | PY | N | Y | N | N | Y | Y | Y | 3 | 8 |
| Hossain 2021 (47) | Y | N | N | N | N | Y | N | PY | PY | N | Y | Y | N | Y | Y | Y | 2,5 | 8 |
| Hu 2022 (48) | Y | PY | N | N | N | N | N | PY | PY | N | Y | Y | Y | Y | Y | Y | 5 | 10 |
| Huang 2022 (49) | Y | PY | N | PY | N | Y | N | PY | PY | N | Y | Y | Y | Y | Y | Y | 6 | 12 |
| Jia 2022 (50) | Y | PY | N | N | Y | Y | N | Y | PY | N | Y | Y | Y | Y | Y | Y | 5 | 12 |
| Johns 2021 (51) | Y | PY | N | N | Y | Y | N | Y | PY | N | Y | Y | Y | Y | Y | Y | 5 | 12 |
| Pashazadeh Kan 2021 (52) | PY | Y | N | PY | Y | Y | N | N | N | N | Y | N | N | Y | Y | Y | 3,5 | 8 |
| Khraisat 2022 (53) | Y | N | N | N | N | N | N | Y | PY | N | Y | Y | Y | Y | Y | N | 4 | 8 |
| Knox 2022 (54) | Y | N | N | N | Y | N | N | Y | PY | N | Y | Y | Y | N | Y | Y | 4 | 9 |
| Kunzler 2021 (55) | Y | Y | N | PY | Y | Y | N | Y | PY | N | Y | Y | N | Y | N | Y | 2,5 | 10 |
| Kuroda 2021 (56) | Y | N | N | N | Y | N | N | Y | PY | N | Y | N | N | Y | Y | Y | 3 | 8 |
| Lee 2021 (57) | Y | PY | N | N | N | N | N | N | PY | N | Y | N | Y | N | Y | Y | 4 | 6 |
| Lee 2022 (58) | Y | PY | N | P Y | N | Y | N | PY | PY | N | N | N | N | N | N | Y | 3 | 7 |
| Li 2021 (59) | Y | Y | N | N | Y | N | N | Y | N | N | Y | N | Y | Y | Y | Y | 4 | 9 |
| Li 2021 (60) | Y | PY | N | N | Y | Y | N | Y | Y | N | Y | Y | Y | Y | N | Y | 3,5 | 10,5 |
| Li 2021 (61) | Y | PY | N | PY | Y | Y | N | N | PY | N | Y | N* | Y | Y | Y | Y | 4,5 | 9,5 |
| Liyanage 2022 (62) | Y | N | N | N | Y | N | N | PY | PY | N | Y | Y | N | Y | Y | Y | 2,5 | 8 |
| Liu 2021 (63) | Y | PY | Y | PY | N | Y | N | PY | PY | N | Y | Y | Y | Y | N | Y | 3,5 | 10 |
| Liu 2021 (64) | Y | N | N | PY | Y | Y | N | N | PY | N | Y | N | N | N | N | N | 2 | 5 |
| Liu 2021 (65) | Y | PY | Y | PY | N | Y | N | PY | PY | N | Y | Y | Y | Y | N | Y | 4,5 | 10 |
| Luo 2021 (66) | Y | Y | Y | PY | Y | Y | N | Y | PY | N | Y | Y | Y | Y | Y | Y | 5 | 13 |
| Ma 2021 (67) | Y | Y | N | N | Y | Y | N | Y | PY | N | Y | N | N | Y | Y | Y | 3,5 | 9,5 |
| Ma 2022 (68) | Y | N | N | N | N | Y | N | PY | PY | N | Y | Y | N | Y | Y | Y | 2,5 | 8 |
| Makwana 2022 (69) | Y | N | N | N | N | Y | N | PY | N | N | Y | N | N | Y | N | Y | 1 | 5,5 |
| Mahmud 2021 (70) | Y | N | Y | N | Y | Y | N | Y | Y | N | Y | N | N | Y | Y | Y | 3 | 10 |
| Marvaldi 2021 (71) | Y | N | N | PY | Y | Y | N | PY | PY | N | Y | Y | Y | Y | Y | Y | 4 | 10,5 |
| Mulyadi 2021 (72) | Y | N | N | N | Y | Y | N | N | PY | N | Y | Y | N | Y | Y | Y | 2,5 | 8,5 |
| Nagarajan 2022 (73) | Y | Y | N | N | Y | N | N | PY | PY | N | Y | Y | N | Y | Y | Y | 3,5 | 9 |
| Natarajan 2022 (74) | Y | Y | N | N | Y | Y | N | N | PY | N | Y | Y | N | Y | Y | Y | 3,5 | 9,5 |
| Necho 2021 (75) | Y | N | N | N | N | Y | N | N | **N** | N | Y | N | N | N | Y | Y | 2 | 5 |
| Nochaiwong 2021 (76) | N | Y | N | Y | Y | Y | N | PY | N | N | Y | Y | Y | Y | Y | Y | 5 | 10,5 |
| Norhayati 2021 (77) | Y | Y | N | N | N | N | N | N | **PY** | N | **Y** | N | N | N | N | Y | 2,5 | 5 |
| Olaya 2021 (78) | Y | N | N | N | Y | N | N | Y | **PY** | N | **Y** | N | N | Y | N | Y | 1,5 | 6 |
| Ozamiz-Etxebarria 2021 (79) | Y | N | N | N | Y | N | N | Y | **PY** | N | **Y** | N | N | N | Y | Y | 2,5 | 7 |
| Panda 2021 (80) | PY | N | N | PY | Y | Y | N | PY | Y | N | Y | N | N | N | Y | Y | 3,5 | 7,5 |
| Pappa 2022 (81) | Y | Y | N | PY | Y | Y | N | PY | PY | N | Y | Y | Y | Y | Y | Y | 5 | 12,5 |
| Phiri 2021 (82) | Y | Y | N | N | Y | Y | N | PY | PY | N | Y | N | N | Y | Y | Y | 3,5 | 9 |
| Prati 2021 (83) | Y | PY | Y | N | Y | N | N | N | N | N | Y | N | N | Y | N | Y | 1,5 | 7 |
| Premraj 2022 (84) | Y | Y | N | N | Y | Y | N | N | PY | N | Y | N | N | Y | N | Y | 3.5 | 7,5 |
| Qi 2022 (85) | Y | Y | N | N | Y | Y | N | Y | Y | N | Y | N | N | Y | Y | Y | 4 | 10 |
| Qiu 2021 (86) | Y | Y | N | N | Y | Y | N | N | PY | N | Y | Y | N | Y | Y | Y | 3 | 9,5 |
| Qiu 2021 (87) | Y | Y | N | N | Y | N | N | PY | N | N | Y | Y | N | Y | Y | Y | 3 | 8,5 |
| Racine 2021 (88) | Y | Y | N | N | Y | Y | N | Y | N | N | Y | Y | N | N | N | Y | 2 | 8 |
| Racine 2022 (89) | Y | Y | N | PY | Y | Y | N | PY | PY | N | Y | Y | Y | Y | Y | Y | 5 | 11,5 |
| Rezaei-Hachesu 2022 (90) | Y | N | N | N | Y | Y | N | Y | N | N | Y | N | N | Y | N | Y | 1 | 7 |
| Robinson 2021 (91) | Y | Y | N | Y | N | N | N | PY | PY | N | Y | Y | Y | Y | Y | Y | 5,5 | 10 |
| Raoofi 2021 (92) | Y | Y | N | N | Y | Y | N | N | **N** | N | Y | Y | N | N | N | Y | 2 | 7 |
| Shorey 2021 (93) | Y | N | N | N | Y | Y | N | Y | PY | N | Y | N | N | Y | Y | Y | 3,5 | 8,5 |
| Salehi 2021 (94) | Y | PY | N | Y | Y | Y | N | PY | N | N | Y | N | N | N | Y | Y | 3,5 | 8 |
| Santabárbara 2021 (95) | Y | PY | N | Y | Y | N | N | Y | PY | N | Y | N | N | N | Y | Y | 4 | 8 |
| Santabarbara 2021 (96) | Y | N | N | N | N | N | N | Y | PY | N | Y | Y | Y | Y | Y | Y | 3,5 | 8,5 |
| Santabarbara 2021 (97) | Y | N | N | Y | N | N | N | Y | PY | N | Y | Y | N | N | Y | Y | 3,5 | 7,5 |
| Santabarbara 2021 (98) | Y | N | N | Y | N | N | N | Y | PY | N | Y | Y | Y | Y | Y | Y | 4 | 9 |
| Santomouro 2021 (99) | Y | PY | Y | Y | Y | Y | N | PY | PY | N | Y | Y | Y | N | N | Y | 4 | 10,5 |
| Safi-Keykalah (100) | Y | PY | N | N | Y | Y | N | PY | PY | N | Y | N | N | Y | Y | Y | 2,5 | 8 |
| Saragih 2021 (101) | Y | Y | N | N | N | Y | N | Y | PY | N | N | N | N | N | N | Y | 1,5 | 5,5 |
| Schafer 2022 (102) | N | N | N | N | N | N | N | N | N | N | N | N | N | N | Y | Y | 1 | 2 |
| Scharma 2022 (103) | Y | N | N | PY | N | N | N | N | PY | N | Y | Y | Y | Y | Y | Y | 4 | 8 |
| Sideli 2021 (104) | Y | N | N | N | N | N | N | Y | PY | N | Y | Y | Y | Y | Y | Y | 3,5 | 8,5 |
| Singh 2021 (105) | Y | N | N | N | N | N | N | Y | PY | N | Y | N | N | N | Y | N | 3 | 4,5 |
| Slusarska 2022 (106) | Y | PY | N | N | Y | Y | N | Y | PY | N | Y | N | Y | Y | Y | Y | 4 | 10 |
| Sun 2021 (107) | Y | N | N | N | Y | Y | N | Y | PY | N | Y | N | N | N | N | Y | 1,5 | 6,5 |
| Tomfohr-Madsen 2021 (108) | Y | Y | N | N | Y | N | N | PY | PY | N | Y | N | N | Y | Y | Y | 3,5 | 8 |
| Varghese 2021 (109) | Y | PY | Y | N | Y | N | N | PY | Y | N | Y | N | N | Y | Y | Y | 3,5 | 9 |
| Wang 2021 (110) | Y | N | N | PY | N | N | N | PY | N | N | Y | N | N | N | Y | Y | 2,5 | 5 |
| Xie 2021 (111) | Y | N | N | N | Y | Y | N | PY | PY | N | Y | N | N | Y | Y | Y | 2,5 | 8 |
| Xiong 2022 (112) | Y | Y | N | Y | Y | Y | N | N | PY | N | Y | Y | N | Y | Y | Y | 4,5 | 10,5 |
| Yan 2021 (113) | PY | Y | Y | Y | Y | N | N | PY | Y | N | Y | N | N | Y | N | Y | 4 | 9 |
| Yan 2022 (114) | Y | Y | N | PY | Y | Y | N | N | PY | N | Y | N | N | Y | Y | Y | 4 | 9 |
| Yang (115) | Y | N | N | N | Y | Y | N | PY | PY | N | Y | N | N | Y | Y | Y | 2,5 | 8 |
| Yunitri (116) | Y | N | N | Y | N | N | N | Y | PY | N | Y | Y | Y | Y | Y | Y | 4,5 | 9,5 |
| Zhang 2021 (117) | Y | Y | Y | PY | Y | Y | N | PY | PY | N | Y | N | N | N | Y | Y | 4 | 9,5 |
| Zhang 2021 (118) | Y | N | Y | PY | Y | N | N | PY | PY | N | Y | Y | Y | Y | Y | Y | 4 | 10,5 |
| Zhang, Chen 2021 (119) | Y | PY | N | N | Y | Y | N | N | PY | N | Y | Y | N | Y | Y | Y | 3 | 9 |
| Zhang, Batra 2021 (120) | Y | PY | N | N | Y | Y | N | N | PY | N | Y | Y | N | Y | Y | Y | 3 | 9 |
| Zhang, Miller (121) | Y | PY | N | N | Y | Y | N | N | PY | N | Y | Y | N | Y | Y | Y | 3 | 9 |
| Zhao 2021 (122) | Y | Y | Y | N | Y | Y | N | PY | PY | N | Y | Y | Y | Y | Y | Y | 4,5 | 12 |
| Zhu 2021 (123) | Y | PY | N | PY | Y | Y | N | PY | PY | N | Y | Y | Y | Y | Y | Y | 4,5 | 11 |

*Note*: * Critical AMSTAR-2 items; total number of ‘yes’ or ‘partial yes’ on critical AMSTAR-2 items; Total = total score of 0 (No), 0,5 (P Yes), 1 (Yes) on all AMSTAR-2 items. The AMSTAR-2 has 16 items for meta-analyses of interventions (non-randomized and randomized controlled trials), but these were slightly modified for meta-analyses of observational studies. **Item 1:** Did the research questions and inclusion criteria for the review include the components of PICO? Modified interpretation: ‘Intervention’ was interpreted as exposure factor (i.e. COVID-19 pandemic) and ‘Comparator’ as no exposure factor (i.e. pre-pandemic). Comparator not applicable for cross sectional studies. **Item 2*:** Did the report of the review contain an explicit statement that the review methods were established prior to conduct of the review and did the report justify any significant deviations from the protocol? Interpretation: rated as ‘No’ if there is no mention of a protocol in text (including acknowledgement section); strictly establishing whether the paper is reporting a type of a written pre-established method of review. If a protocol is mentioned but authors provide no exact location it is a partial ‘yes’. **Item 3:** Did the review authors explain their selection of the study designs for inclusion in the review? Interpretation: assess if study designs listed in the protocol were assessed and whether a clear description of the inclusion of the studies based on study designs was provided and handled correctly statistically (e.g. separate reporting on different study-design types). **Item 4*:** Did the review authors use a comprehensive literature search strategy? **Item 5:** Did the review authors perform study selection in duplicate? **Item 6:** Did the review authors perform data extraction in duplicate? **Item 7*:** Did the review authors provide a list of excluded studies and justify the exclusions? Interpretation: usually a ‘No’ due to strictness of the check boxes (i.e. reasons for exclusion must be very detailed with reference to each specified excluded publication at the stage of papers screened full text). **Item 8:** Did the review authors describe the included studies in adequate detail? **Item 9*:** Did the review authors use a satisfactory technique for assessing the risk of bias (RoB) in individual studies that were included in the review? Modified interpretation: original items listed were disregarded and replaced. For ‘P yes’ employed tool must have assessed all of the following domains: selection, outcome assessment, analysis and for full ‘yes’, the employed tool must have assessed all of the following domains: selection, outcome, assessment, analysis, selective reporting, conflict of interest. In the case of ad-hoc modified tools for which review authors do not provide a clear list of items/domains this item was rated as ‘No’. **Item 10:** Did the review authors report on the sources of funding for the studies included in the review? Interpretation: the meta-analysis had to have looked into the funding sources of each study it assessed. **Item 11*:** If meta-analysis was justified did the review authors use appropriate methods for statistical combination of results? Modified interpretation: original list disregarded, instead all of the following must have been performed: used a random effect meta-analysis, explored the heterogeneity by either subgroup analysis OR meta-regression. **Item 12:** If meta-analysis was performed did the review authors assess the potential impact of RoB in individual studies on the results of the meta-analysis or other evidence synthesis? Interpretation: ‘Yes’ if reviewers only included low risk of bias studies; if not reviewers should have assessed the impact of risk of bias on the results. **Item 13*:** Did the review authors account for RoB in individual studies when interpreting/ discussing the results of the review? Interpretation: ‘Yes’ if the meta-analysis was done in Low Risk Of Bias studies alone and if not, authors should have performed analyses to account for the possible bias. **Item 14:** Did the review authors provide a satisfactory explanation for, and discussion of, any heterogeneity observed in the results of the review? **Item 15*:** If they performed quantitative synthesis did the review authors carry out an adequate investigation of publication bias (small study bias) and discuss its likely impact on the results of the review? Interpretation: reporting and discussing heterogeneity and whether they have conducted appropriate analyses. Only mentioning this was not satisfactory. **Item 16:** Did the review authors report any potential sources of conflict of interest, including any funding they received for conducting the review?

### References

1. Abdulla EK, Velladath SU, Varghese A, Anju M. Depression and anxiety associated with COVID- 19 pandemic among healthcare professionals in India- A systematic review and meta-analysis. Clin Epidemiol Glob Health. 2021;12:100888. <https://doi.org/10.1016/j.cegh.2021.100888>. PMID: [34751253](https://pubmed.ncbi.nlm.nih.gov/34751253/)

2. Adrianto N, Caesarlia J, Pajala FB. Depression in pregnant and postpartum women during COVID-19 pandemic: systematic review and meta-analysis. Obstet Gynecol Sci. 2022; 65(4):287–302. <https://doi.org/10.5468/ogs.21265>. PMID: [35754363](https://pubmed.ncbi.nlm.nih.gov/35754363/)

3. Adibi A, Golitaleb M, Farrahi-Ashtiani I, Pirani D, Yousefi K, Jamshidbeigi Y, et al. The Prevalence of Generalized Anxiety Disorder Among Health Care Workers During the COVID-19 Pandemic: A Systematic Review and Meta-Analysis. Front Psychiatry. 2021; 12:658846. <https://doi.org/10.3389/fpsyt.2021.658846>. PMID: [34135784](https://pubmed.ncbi.nlm.nih.gov/34135784/)

4. Afridi MZ, Akhtar P, Khan MN. A Systematic Review and Meta-Analysis of Prevalence of Depressive Symptoms among Healthcare Workers in Pakistan during Covid Pandemic. Pakistan Journal of Medical & Health Sciences. 2022; 16(02):2–5. <https://doi.org/10.53350/pjmhs221622>.

5. Alzahrani F, Alshahrani NZ, Abu Sabah A, Zarbah A, Abu Sabah S, Mamun MA. Prevalence and factors associated with mental health problems in Saudi general population during the coronavirus disease 2019 pandemic: A systematic review and meta-analysis. Psych J. 2022; 11(1):18–29. <https://doi.org/10.1002/pchj.516>. PMID: [34986503](https://pubmed.ncbi.nlm.nih.gov/34986503/)

6. Arora T, Grey I, Östlundh L, Lam KBH, Omar OM, Arnone D. The prevalence of psychological consequences of COVID-19: A systematic review and meta-analysis of observational studies. J Health Psychol. 2022;27(4):805–824. <https://doi.org/10.1177/1359105320966639>. PMID: [33118376](https://pubmed.ncbi.nlm.nih.gov/33118376/)

7. Aymerich C, Pedruzo B, Pérez JL, Laborda M, Herrero J, Blanco J, et al. COVID-19 pandemic effects on health worker’s mental health: Systematic review and meta-analysis. Eur Psychiatry. 2022; 65(1):e10. <https://doi.org/10.1192/j.eurpsy.2022.1>. PMID: 35060458

8. Ayubi E, Bashirian S, Khazaei S. Depression and Anxiety Among Patients with Cancer During COVID-19 Pandemic: A Systematic Review and Meta-analysis. J Gastrointest Cancer. 2021; 52(2):499-507. <https://doi.org/10.1007/s12029-021-00643-9>. PMID: [33950368](https://pubmed.ncbi.nlm.nih.gov/33950368/)

9. Bello UM, Kannan P, Chutiyami M, Salihu D, Cheong AMY, Miller T, et al. Prevalence of Anxiety and Depression Among the General Population in Africa During the COVID-19 Pandemic: A Systematic Review and Meta-Analysis. Front Public Health. 2022; 10:814981. <https://doi.org/10.3389/fpubh.2022.814981>. PMID: [35655463](https://pubmed.ncbi.nlm.nih.gov/35655463/)

10. Balakrishnan V, Ng KS, Kaur W, Govaichelvan K, Lee ZL. COVID-19 depression and its risk factors in Asia Pacific – A systematic review and meta-analysis. J Affect Disord. 2022; 298:47–56. <https://doi.org/10.1016/j.jad.2021.11.048>. PMID: [34801606](https://pubmed.ncbi.nlm.nih.gov/34801606/)

11. Bareeqa SB, Ahmed SI, Samar SS, Yasin W, Zehra S, Monese GM, et al. Prevalence of depression, anxiety and stress in china during COVID-19 pandemic: A systematic review with meta-analysis. Int J Psychiatry Med . 2021; 56(4):210–27. <https://doi.org/10.1177/0091217420978005>. PMID: [33243029](https://pubmed.ncbi.nlm.nih.gov/33243029/)

12. Batra K, Sharma M, Batra R, Singh TP, Schvaneveldt N. Assessing the Psychological Impact of COVID-19 among College Students: An Evidence of 15 Countries. Healthcare (Basel). 2021; 9(2):222. <https://doi.org/10.3390/healthcare9020222>. PMID: [33671363](https://pubmed.ncbi.nlm.nih.gov/33671363/)

13. Bussières EL, Malboeuf-Hurtubise C, Meilleur A, Mastine T, Hérault E, Chadi N, et al. Consequences of the COVID-19 Pandemic on Children’s Mental Health: A Meta-Analysis. Front Psychiatry. 2021;12:2125. <https://doi.org/10.3389/fpsyt.2021.691659>. PMID: [34925080](https://pubmed.ncbi.nlm.nih.gov/34925080/)

14. Carvalho PO, Hülsdünker T, Carson F. The impact of the COVID-19 lockdown on european students’ negative emotional symptoms: A systematic review and meta-analysis. Behav Sci (Basel); 2022; 12(1):3. <https://doi.org/10.3390/bs12010003>. PMID: [35049614](https://pubmed.ncbi.nlm.nih.gov/35049614/)

15. Castaldelli-Maia JM, Marziali ME, Lu Z, Martins SS. Investigating the effect of national government physical distancing measures on depression and anxiety during the COVID-19 pandemic through meta-analysis and meta-regression. 2021; 51(6):881-893. <https://doi.org/10.1017/s0033291721000933>. PMID: [33648613](https://pubmed.ncbi.nlm.nih.gov/33648613/)

16. Cénat JM, Blais-Rochette C, Kokou-Kpolou CK, Noorishad PG, Mukunzi JN, McIntee SE, et al. Prevalence of symptoms of depression, anxiety, insomnia, posttraumatic stress disorder, and psychological distress among populations affected by the COVID-19 pandemic: A systematic review and meta-analysis. Psychiatry Res. 2021; 295:113599. <https://doi.org/10.1016/j.psychres.2020.113599>. PMID: [33285346](https://pubmed.ncbi.nlm.nih.gov/33285346/)

17. Cénat JM, Farahi SMMM, Dalexis RD, Darius WP, Bekarkhanechi FM, Poisson H, et al. The global evolution of mental health problems during the COVID-19 pandemic: A systematic review and meta-analysis of longitudinal studies. J Affect Disord. 2022; 315:70-95. <https://doi.org/10.1016/j.jad.2022.07.011>. PMID: [35842064](https://pubmed.ncbi.nlm.nih.gov/35842064/).

18. Cevik A, Onat Koroglu C, Karacam Z, Gokyildiz Surucu S, Alan S. Effects of the Covid-19 Pandemic on the Prevalence of Insomnia, Anxiety, and Depression During Pregnancy: A Systematic Review and Meta-Analysis. Clin Nurs Res. 2022; 31(8):1405–1421. <https://doi.org/10.1177/10547738221112748>. PMID: [35912813](https://pubmed.ncbi.nlm.nih.gov/35912813/)

19. Chai J, Xu H, An N, Zhang P, Liu F, He S, et al. The Prevalence of Mental Problems for Chinese Children and Adolescents During COVID-19 in China: A Systematic Review and Meta-Analysis. Front Pediatr. 2021; 9:661796. <https://doi.org/10.3389/fped.2021.661796>. PMID: [34692601](https://pubmed.ncbi.nlm.nih.gov/34692601/)

20. Chang JJ, Ji Y, Li YH, Pan HF, Su PY. Prevalence of anxiety symptom and depressive symptom among college students during COVID-19 pandemic: A meta-analysis. J Affect Disord . 2021 ; 292:242–54. <https://doi.org/10.1016/j.jad.2021.05.109>. PMID: [34134022](https://pubmed.ncbi.nlm.nih.gov/34134022/)

21. Chekole YA, Abate SM. Global prevalence and determinants of mental health disorders during the COVID-19 pandemic: A systematic review and meta-analysis. Ann Med Surg (Lond). 2021; 68:102634. <https://doi.org/10.1016/j.amsu.2021.102634>. PMID: [34386226](https://pubmed.ncbi.nlm.nih.gov/34386226/)

22. Chen J, Farah N, Dong RK, Chen RZ, Xu W, Yin J, et al. Mental health during the covid-19 crisis in Africa: A systematic review and meta-analysis. Int J Environ Res Public Health. 2021; 18(20):10604. <https://doi.org/10.3390/ijerph182010604>. PMID: [34682357](https://pubmed.ncbi.nlm.nih.gov/34682357/)

23. Chen J, Yang K, Cao Y, Du Y, Wang N, Qu M. Depressive Symptoms Among Children and Adolescents in China During the Coronavirus Disease-19 Epidemic: A Systematic Review and Meta-Analysis. Front Psychiatry. 2022; 13:870346. <https://doi.org/10.3389/fpsyt.2022.870346>. PMID: [35463512](https://pubmed.ncbi.nlm.nih.gov/35463512/)

24. Chen Q, Li W, Xiong J, Zheng X. Prevalence and Risk Factors Associated with Postpartum Depression during the COVID-19 Pandemic: A Literature Review and Meta-Analysis. Int J Environ Res Public Health. 2022; 16;19(4):2219. <https://doi.org/10.3390/ijerph19042219>. PMID: [35206407](https://pubmed.ncbi.nlm.nih.gov/35206407/)

25. Ching SM, Ng KY, Lee KW, Yee A, Lim PY, Ranita H, et al. Psychological distress among healthcare providers during COVID-19 in Asia: Systematic review and meta-analysis. PLoS One. 2021; 16(10):e0257983. <https://doi.org/10.1371/journal.pone.0257983>. PMID: [34648526](https://pubmed.ncbi.nlm.nih.gov/34648526/).

26. Dal Santo T, Sun Y, Wu Y, He C, Wang Y, Jiang X, et al. Systematic review of mental health symptom changes by sex or gender in early-COVID-19 compared to pre-pandemic. Sci Rep. 2022; 12(1):11417. <https://doi.org/10.1038/s41598-022-14746-1>. PMID: [35794116](https://pubmed.ncbi.nlm.nih.gov/35794116/)

27. da Silva FCT, Neto MLR. Psychological effects caused by the COVID-19 pandemic in health professionals: A systematic review with meta-analysis. Biol Psychiatry. 2021; 104:110062. <https://doi.org/10.1016/j.pnpbp.2020.110062>. PMID: [32771337](https://pubmed.ncbi.nlm.nih.gov/32771337/)

28. Demissie DB, Bitew ZW. Mental health effect of COVID-19 pandemic among women who are pregnant and/or lactating: A systematic review and meta-analysis. SAGE Open Med . 2021 ; 9:205031212110261. <https://doi.org/10.1177/20503121211026195>. PMID: [34262762](https://pubmed.ncbi.nlm.nih.gov/34262762/)

29. Deng Y, Chen Y, Zhang B. Different prevalence trend of depression and anxiety among healthcare workers and general public before and after the peak of COVID-19 occurred in China: A meta-analysis. Asian J Psychiatr. 2021; 56:102547. <https://doi.org/10.1016/j.ajp.2021.102547>. PMID: [33465749](https://pubmed.ncbi.nlm.nih.gov/33465749/)

30. Deng J, Zhou F, Hou W, Silver Z, Wong CY, Chang O, et al. The prevalence of depressive symptoms, anxiety symptoms and sleep disturbance in higher education students during the COVID-19 pandemic: A systematic review and meta-analysis. Psychiatry Res. 2021; 301:113863. <https://doi.org/10.1016/j.psychres.2021.113863> PMID: [33984824](https://pubmed.ncbi.nlm.nih.gov/33984824/)

31. de Sousa Júnior GM, Tavares VD de O, de Meiroz Grilo MLP, Coelho MLG, Lima-Araújo GL de, Schuch FB, et al. Mental Health in COVID-19 Pandemic: A Meta-Review of Prevalence Meta-Analyses. Front Psychol. 2021; 12:703838. <https://doi.org/10.3389/fpsyg.2021.703838>. PMID: [34621212](https://pubmed.ncbi.nlm.nih.gov/34621212/).

32. Delanerolle G, Zeng Y, Shi JQ, Yeng X, Goodison W, Shetty A, et al. Mental health impact of the Middle East respiratory syndrome, SARS, and COVID-19: A comparative systematic review and meta-analysis. World J Psychiatry. 2022; 12(5):739–765. <https://doi.org/10.5498/wjp.v12.i5.739>. PMID: [35663292](https://pubmed.ncbi.nlm.nih.gov/35663292/)

33. Dong F, Liu HL, Yang M, Lu CL, Dai N, Zhang Y, et al. Immediate Psychosocial Impact on Healthcare Workers During COVID-19 Pandemic in China: A Systematic Review and Meta-Analysis. Front Psychol. 2021; 12:645460. <https://doi.org/10.3389/fpsyg.2021.645460>. PMID: [34122233](https://pubmed.ncbi.nlm.nih.gov/34122233/)

34. Dong F, Liu H liang, Dai N, Yang M, Liu J ping. A living systematic review of the psychological problems in people suffering from COVID-19. J Affect Disord. 2021; 292:172–88. <https://doi>.org/10.1016/j.jad.2021.05.060. PMID: [34126309](https://pubmed.ncbi.nlm.nih.gov/34126309/)

35. Dragioti E, Li H, Tsitsas G, Lee KH, Choi J, Kim J, et al. A large-scale meta-analytic atlas of mental health problems prevalence during the COVID-19 early pandemic. J Med Virol. 2022; 94(5):1935–1949. <https://doi.org/10.1002/jmv.27549>. PMID: [34958144](https://pubmed.ncbi.nlm.nih.gov/34958144/)

36. Dutta A, Sharma A, Torres-Castro R, Pachori H, Mishra S. Mental health outcomes among health-care workers dealing with COVID-19/severe acute respiratory syndrome coronavirus 2 pandemic: A systematic review and meta-analysis. Indian J Psychiatry . 2021; 63(4):335-347. <https://doi.org/10.4103/psychiatry.indianjpsychiatry_1029_20>. PMID: [34456346](https://pubmed.ncbi.nlm.nih.gov/34456346/)

37. Ebrahim AH, Dhahi A, Husain MA, Jahrami H. The Psychological Well-Being of University Students amidst COVID-19 Pandemic Scoping review, systematic review and meta-analysis. Sultan Qaboos Univ Med J. 2022; 22(2):179–197.<https://doi.org/10.18295/squmj.6.2021.081>. PMID: [35673293](https://pubmed.ncbi.nlm.nih.gov/35673293/)

38. El-Qushayri AE, Dahy A, Reda A, Mahmoud MA, Mageed SA, Kamel AMA, et al. A closer look at the high burden of psychiatric disorders among healthcare workers in Egypt during the COVID-19 pandemic. Epidemiol Health. 2021;. 43:e2021045. <https://doi.org/10.4178/epih.e2021045>. PMID: [34265893](https://pubmed.ncbi.nlm.nih.gov/34265893/)

39. Fan FC, Zhang SY, Cheng Y. Incidence of psychological illness after coronavirus outbreak: a meta-analysis study. J Epidemiol Community Health. 2021;75(9):836–42. <https://doi>.org/10.1136/jech-2020-21592. PMID: [33632722](https://pubmed.ncbi.nlm.nih.gov/33632722/)

40. Fang Y, Ji B, Liu Y, Zhang J, Liu Q, Ge Y, et al. The prevalence of psychological stress in student populations during the COVID-19 epidemic: a systematic review and meta-analysis. Sci Rep. 2022; 12(1):12118. <https://doi.org/10.1038/s41598-022-16328-7>. PMID: [35840641](https://pubmed.ncbi.nlm.nih.gov/35840641/).

41. Ghahramani S, Lankarani KB, Yousefi M, Heydari K, Shahabi S, Azmand S. A Systematic Review and Meta-Analysis of Burnout Among Healthcare Workers During COVID-19. Front Psychiatry. 2021; 12:758849. <https://doi.org/10.3389/fpsyt.2021.758849>. PMID: [34858231](https://pubmed.ncbi.nlm.nih.gov/34858231/)

42. Ghazanfarpour M, Bahrami F, Rashidi Fakari F, Ashrafinia F, Babakhanian M, Dordeh M, et al. Prevalence of anxiety and depression among pregnant women during the COVID-19 pandemic: a meta-analysis. J Psychosom Obstet Gynaecol. 2022; 43(3):315-326. <https://doi.org/10.1080/0167482x.2021.1929162>. PMID: [34165032](https://pubmed.ncbi.nlm.nih.gov/34165032/).

43. Guo S, Kaminga AC, Xiong J. Depression and Coping Styles of College Students in China During COVID-19 Pandemic: A Systemic Review and Meta-Analysis. Front Public Health. 2021; 9:613321. <https://doi.org/10.3389/fpubh.2021.613321>. PMID: [34307268](https://pubmed.ncbi.nlm.nih.gov/34307268/)

44. Halemani K, Issac A, Mishra P, Dhiraaj S, Mavinatop S. Prevalence of Anxiety, Depression, Stress, and Insomnia among Healthcare Workers during Covid-19: A Systematic Review and Meta-Analysis. Nursing Journal of India. 2021; CXII(06):269–277. <https://doi.org/10.48029/NJI.2021.CXII605>

45. Hao Q, Wang D, Xie M, Tang Y, Dou Y, Zhu L, et al. Prevalence and Risk Factors of Mental Health Problems Among Healthcare Workers During the COVID-19 Pandemic: A Systematic Review and Meta-Analysis. Front Psychiatry. 2021; 12:567381. <https://doi.org/10.3389/fpsyt.2021.567381>. PMID: [34211406](https://pubmed.ncbi.nlm.nih.gov/34211406/)

46. Hosen I, al-Mamun F, Mamun MA. Prevalence and risk factors of the symptoms of depression, anxiety, and stress during the COVID-19 pandemic in Bangladesh: a systematic review and meta-analysis. Glob Ment Health (Camb). 2021; 8:e47. <https://doi.org/10.1017/gmh.2021.49>. PMID: [35145709](https://pubmed.ncbi.nlm.nih.gov/35145709/)

47. Hossain MM, Rahman M, Trisha NF, Tasnim S, Nuzhath T, Hasan NT, et al. Prevalence of anxiety and depression in South Asia during COVID-19: A systematic review and meta-analysis. Heliyon. 2021; 7(4):e06677 ; <https://doi.org/10.1016/j.heliyon.2021.e06677>. PMID: [33898819](https://pubmed.ncbi.nlm.nih.gov/33898819/)

48. Hu N, Deng H, Yang H, Wang C, Cui Y, Chen J, et al. The pooled prevalence of the mental problems of Chinese medical staff during the COVID-19 outbreak: A meta-analysis. J Affect Disord. 2022; 303:323–30. <https://doi.org/10.1016/j.jad.2022.02.045>. PMID: [35183620](file:///C:\Users\awn233\AppData\Roaming\Microsoft\Word\35183620)

49. Huang G, Chu H, Chen R, Liu D, Banda KJ, O’Brien AP, et al. Prevalence of depression, anxiety, and stress among first responders for medical emergencies during COVID-19 pandemic: A meta-analysis. J Glob Health. 2022;12:05028. <https://doi.org/10.7189/jogh.12.05028>. PMID: [35871411](https://pubmed.ncbi.nlm.nih.gov/35871411/)

50. Jia Q, Qu Y, Sun H, Huo H, Yin H, You D. Mental Health Among Medical Students During COVID-19: A Systematic Review and Meta-Analysis. Front Psychol. 2022; 13:846789. <https://doi.org/10.3389/fpsyg.2022.846789>. PMID: [35619776](https://pubmed.ncbi.nlm.nih.gov/35619776/)

51. Johns G, Samuel V, Freemantle L, Lewis J, Waddington L. The global prevalence of depression and anxiety among doctors during the covid-19 pandemic: Systematic review and meta-analysis. J Affect Disord. 2022; 298:431–441. <https://doi.org/10.1016/j.jad.2021.11.026>. PMID: [34785264](https://pubmed.ncbi.nlm.nih.gov/34785264/)

52. Pashazadeh Kan F, Raoofi S, Rafiei S, Khani S, Hosseinifard H, Tajik F, et al. A systematic review of the prevalence of anxiety among the general population during the COVID-19 pandemic. J Affect Disord. 2021; 293:391-398. <https://doi.org/10.1016/j.jad.2021.06.073>. PMID: [34246947](https://pubmed.ncbi.nlm.nih.gov/34246947/)

53. Khraisat BR, Al-Jeady AM, Alqatawneh DA, Toubasi AA, AlRyalat SA. The prevalence of mental health outcomes among eating disorder patients during the COVID-19 pandemic: A meta-analysis. Clin Nutr ESPEN. 2022; 48:141–147. <https://doi.org/10.1016/j.clnesp.2022.01.033>. PMID: [35331484](https://pubmed.ncbi.nlm.nih.gov/35331484/)

54. Knox L, Karantzas GC, Romano D, Feeney JA, Simpson JA. One year on: What we have learned about the psychological effects of COVID-19 social restrictions: A meta-analysis. Curr Opin Psychol. 2022; 46:101315. <https://doi.org/10.1016/j.copsyc.2022.101315>. PMID: [35398753](https://pubmed.ncbi.nlm.nih.gov/35398753/)

55. Kunzler AM, Röthke N, Günthner L, Stoffers-Winterling J, Tüscher O, Coenen M, et al. Mental burden and its risk and protective factors during the early phase of the SARS-CoV-2 pandemic: systematic review and meta-analyses. Global Health. 2021; 17(1):1–29. <https://doi.org/10.1186/s12992-021-00670-y>. PMID: [33781283](https://pubmed.ncbi.nlm.nih.gov/33781283/)

56. Kuroda N, Kubota T. Psychological impact of the COVID-19 pandemic for patients with epilepsy: A systematic review and meta-analysis. Epilepsy Behav. 2021; 124:108340. <https://doi.org/10.1016/j.yebeh.2021.108340>. PMID: [34600283](https://pubmed.ncbi.nlm.nih.gov/34600283/)

57. Lee Y, Lui LMW, Chen-Li D, Liao Y, Mansur RB, Brietzke E, et al. Government response moderates the mental health impact of COVID-19: A systematic review and meta-analysis of depression outcomes across countries. J Affect Disord. 2021; 290:364-377. <https://doi.org/10.1016/j.jad.2021.04.050>. PMID: [34052584](https://pubmed.ncbi.nlm.nih.gov/34052584/)

58. Lee KW, Ang CS, Lim SH, Siau CS, Ong LTD, Ching SM, et al. Prevalence of mental health conditions among people living with HIV during the COVID-19 pandemic: A rapid systematic review and meta-analysis. HIV Med. 2022; 23(9):990–1001. <https://doi.org/10.1111/hiv.13299>. PMID: [35304829](https://pubmed.ncbi.nlm.nih.gov/35304829/)

59. Li Y, Scherer N, Felix L, Kuper H. Prevalence of depression, anxiety and post-traumatic stress disorder in health care workers during the COVID-19 pandemic: A systematic review and meta-analysis. PLoS One . 2021 ;16(3):e0246454. <https://doi.org/10.1371/journal.pone.0246454>. PMID: [33690641](https://pubmed.ncbi.nlm.nih.gov/33690641/)

60. Li W, Zhang H, Zhang C, Luo J, Wang H, Wu H, et al. The Prevalence of Psychological Status During the COVID-19 Epidemic in China: A Systemic Review and Meta-Analysis. Front Psychol. 2021; 12:614964. <https://doi.org/10.3389/fpsyg.2021.614964>. PMID: [34017278](https://pubmed.ncbi.nlm.nih.gov/34017278/)

61. Li Y, Wang A, Wu Y, Han N, Huang H. Impact of the COVID-19 Pandemic on the Mental Health of College Students: A Systematic Review and Meta-Analysis. Front Psychol. 2021; 12:669119. <https://doi.org/10.3389/fpsyg.2021.669119>. PMID: [34335381](https://pubmed.ncbi.nlm.nih.gov/34335381/)

62. Liyanage S, Saqib K, Khan AF, Thobani TR, Tang WC, Chiarot CB, et al. Prevalence of anxiety in university students during the covid-19 pandemic: A systematic review. Int J Environ Res Public Health. 2022; 19(1):62. <https://doi.org/10.3390/ijerph19010062>. PMID: [35010323](https://pubmed.ncbi.nlm.nih.gov/35010323/)

63. Liu X, Zhu M, Zhang R, Zhang J, Zhang C, Liu P, et al. Public mental health problems during COVID-19 pandemic: a large-scale meta-analysis of the evidence. Transl Psychiatry. 2021; 11(1):384. <https://doi.org/10.1038/s41398-021-01501-9>. PMID: [34244469](https://pubmed.ncbi.nlm.nih.gov/34244469/)

64. Liu X, Wang G, Zhang J, Wang S. Prevalence of depression and anxiety among health care workers in designated hospitals during the COVID-19 epidemic: A meta-analysis. Chinese Journal of Evidence-Based Medicine. 2021; 21(9):1035-1042. Available from: <https://pesquisa.bvsalud.org/global-literature-on-novel-coronavirus-2019-ncov/resource/pt/covidwho-1449172?lang=en>.

65. Liu C, Pan W, Li L, Li B, Ren Y, Ma X. Prevalence of depression, anxiety, and insomnia symptoms among patients with COVID-19: A meta-analysis of quality effects model. J Psychosom Res. 2021; 147:110516. <https://doi.org/10.1016/j.jpsychores.2021.110516>. PMID: [34023580](https://pubmed.ncbi.nlm.nih.gov/34023580/)

66. Luo W, Zhong BL, Chiu HFK. Prevalence of depressive symptoms among Chinese university students amid the COVID-19 pandemic: A systematic review and meta-analysis. Epidemiol Psychiatr Sci. 2021 ;30:e31. <https://doi.org/10.1017/s2045796021000202>. PMID: [33766163](https://pubmed.ncbi.nlm.nih.gov/33766163/)

67. Ma L, Mazidi M, Li K, Li Y, Chen S, Kirwan R, et al. Prevalence of mental health problems among children and adolescents during the COVID-19 pandemic: A systematic review and meta-analysis. J Affect Disord. 2021;293:78–89. <https://doi.org/10.1016/j.jad.2021.06.021>. PMID: [34174475](https://pubmed.ncbi.nlm.nih.gov/34174475/)

68. Ma K, Liang L, Chutiyami M, Nicoll S, Khaerudin T, Ha X van. COVID-19 pandemic-related anxiety, stress, and depression among teachers: A systematic review and meta-analysis. Work. 2022; 73(1):3–27. <https://doi.org/10.3233/wor-220062>. PMID: [35527618](https://pubmed.ncbi.nlm.nih.gov/35527618/)

69. Makwana K, Jain S, Makwana A, Rathod NM. Prevalence of depression among Indian medical students in COVID-19 pandemic – A meta-analysis. Natl J Physiol Pharm Pharmacol. 2022; 12(7):916–916. <http://dx.doi.org/10.5455/njppp.2022.12.052331202210062022>

70. Mahmud S, Hossain S, Muyeed A, Islam MM, Mohsin M. The global prevalence of depression, anxiety, stress, and, insomnia and its changes among health professionals during COVID-19 pandemic: A rapid systematic review and meta-analysis. Heliyon. 2021;7(7):e07393. <https://doi.org/10.1016/j.heliyon.2021.e07393>. PMID: [34278018](https://pubmed.ncbi.nlm.nih.gov/34278018/)

71. Marvaldi M, Mallet J, Dubertret C, Moro MR, Guessoum SB. Anxiety, depression, trauma-related, and sleep disorders among healthcare workers during the COVID-19 pandemic: A systematic review and meta-analysis. Neurosci Biobehav Rev. 2021 Jul 1;126:252–64.

72. Mulyadi M, Tonapa SI, Luneto S, Lin WT, Lee BO. Prevalence of mental health problems and sleep disturbances in nursing students during the COVID-19 pandemic: A systematic review and meta-analysis. Nurse Educ Pract. 2021; 57:103228. <https://doi.org/10.1016/j.nepr.2021.103228>. PMID: [34653783](https://pubmed.ncbi.nlm.nih.gov/34653783/)

73. Nagarajan R, Krishnamoorthy Y, Basavarachar V, Dakshinamoorthy R. Prevalence of post-traumatic stress disorder among survivors of severe COVID-19 infections: A systematic review and meta-analysis. J Affect Disord; 2022 299:52-59. <https://doi.org/10.1016/j.jad.2021.11.040>. PMID: [34800571](https://pubmed.ncbi.nlm.nih.gov/34800571/)

74. Natarajan A, Shetty A, Delanerolle G, Zeng Y, Zhang Y, Raymont V, et al. A systematic review and meta-analysis of Long COVID symptoms. medRxiv. 2022 [cited 2022 Dec 12]. 2022.03.08.22272091 [Preprint]. Available from: <https://doi.org/10.1101/2022.03.08.22272091>

75. Necho M, Tsehay M, Birkie M, Biset G, Tadesse E. Prevalence of anxiety, depression, and psychological distress among the general population during the COVID-19 pandemic: A systematic review and meta-analysis. Int J Soc Psychiatry. 2021 Nov;67(7):892-906. <https://doi.org/10.1177/00207640211003121>. PMID: [33794717](https://pubmed.ncbi.nlm.nih.gov/33794717/)

76. Nochaiwong S, Ruengorn C, Thavorn K, Hutton B, Awiphan R, Phosuya C, et al. Global prevalence of mental health issues among the general population during the coronavirus disease-2019 pandemic: a systematic review and meta-analysis. Sci Rep. 2021 ;11(1): 10173. <https://doi.org/10.1038/s41598-021-89700-8>. PMID: [33986414](https://pubmed.ncbi.nlm.nih.gov/33986414/)

77. Norhayati MN, Yusof RC, Azman MY. Prevalence of psychological impacts on healthcare providers during COVID-19 pandemic in Asia. Vol. 18, International Journal of Environmental Research and Public Health. 2021; 18(17):9157 <https://doi.org/10.3390/ijerph18179157>. PMID: [34501747](https://pubmed.ncbi.nlm.nih.gov/34501747/)

78. Olaya B, Pérez-Moreno M, Bueno-Notivol J, Gracia-García P, Lasheras I, Santabárbara J. Prevalence of depression among healthcare workers during the covid-19 outbreak: A systematic review and meta-analysis. J Clin Med. 2021; 10(15):3406. <https://doi.org/10.3390/jcm10153406>. PMID: [34362188](https://pubmed.ncbi.nlm.nih.gov/34362188/)

79. Ozamiz-Etxebarria N, Mondragon NI, Bueno-Notivol J, Pérez-Moreno M, Santabárbara J. Prevalence of anxiety, depression, and stress among teachers during the covid-19 pandemic: A rapid systematic review with meta-analysis. Brain Sci. 2021; 11(9):1172 . <https://doi.org/10.3390/brainsci11091172>. PMID: [34573192](https://pubmed.ncbi.nlm.nih.gov/34573192/)

80. Panda PK, Gupta J, Chowdhury SR, Kumar R, Meena AK, Madaan P, et al. Psychological and Behavioral Impact of Lockdown and Quarantine Measures for COVID-19 Pandemic on Children, Adolescents and Caregivers: A Systematic Review and Meta-Analysis. J Trop Pediatr. 2021; 67(1):fmaa122. <https://doi.org/10.1093/tropej/fmaa122>. PMID: [33367907](https://pubmed.ncbi.nlm.nih.gov/33367907/)

81. Pappa S, Chen J, Barnett J, Chang A, Dong RK, Xu W, et al. A systematic review and meta-analysis of the mental health symptoms during the Covid-19 pandemic in Southeast Asia. Vol. 76, Psychiatry and Clinical Neurosciences. 2022; 76(2):41-50. <https://doi.org/10.1111/pcn.13306>. PMID: [34704305](https://pubmed.ncbi.nlm.nih.gov/34704305/)

82. Phiri P, Ramakrishnan R, Rathod S, Elliot K, Thayanandan T, Sandle N, et al. An evaluation of the mental health impact of SARS-CoV-2 on patients, general public and healthcare professionals: A systematic review and meta-analysis. EClinicalMedicine. 2021; 34:100806. <https://doi.org/10.1016/j.eclinm.2021.100806>. PMID: [33842872](https://pubmed.ncbi.nlm.nih.gov/33842872/)

83. Prati G, Mancini AD. The psychological impact of COVID-19 pandemic lockdowns: A review and meta-analysis of longitudinal studies and natural experiments. Psychol Med. 2021; 51(2):201-211. <https://doi.org/10.1017/s0033291721000015>. PMID: [33436130](https://pubmed.ncbi.nlm.nih.gov/33436130/)

84. Premraj L, Kannapadi N v., Briggs J, Seal SM, Battaglini D, Fanning J, et al. Mid and long-term neurological and neuropsychiatric manifestations of post-COVID-19 syndrome: A meta-analysis. J Neurol Sci. 2022; 434:120162. <https://doi.org/10.1016/j.jns.2022.120162>. PMID: [35121209](https://pubmed.ncbi.nlm.nih.gov/35121209/)

85. Qi G, Yuan P, Qi M, Hu X, Shi S, Shi X. Influencing Factors of High PTSD Among Medical Staff During COVID-19: Evidences From Both Meta-analysis and Subgroup Analysis. Saf Health Work. 2022;13(3):269–278. <https://doi.org/10.1016/j.shaw.2022.06.003>. PMID: [35784492](https://pubmed.ncbi.nlm.nih.gov/35784492/)

86. Qiu D, Li Y, Li L, He J, Ouyang F, Xiao S. Prevalence of post-traumatic stress symptoms among people influenced by coronavirus disease 2019 outbreak: A meta-analysis. Eur Psychiatry. 2021; 64(1):e30. <https://doi.org/10.1192/j.eurpsy.2021.24>. PMID: [33843547](https://pubmed.ncbi.nlm.nih.gov/33843547/)

87. Qiu D, Li Y, Li L, He J, Ouyang F, Xiao S. Infectious Disease Outbreak and Post-Traumatic Stress Symptoms: A Systematic Review and Meta-Analysis. Front Psychol. 2021; 12:668784. <https://doi.org/10.3389/fpsyg.2021.668784>. PMID: [34421723](https://pubmed.ncbi.nlm.nih.gov/34421723/)

88. Racine N, McArthur BA, Cooke JE, Eirich R, Zhu J, Madigan S. Global Prevalence of Depressive and Anxiety Symptoms in Children and Adolescents during COVID-19: A Meta-analysis. JAMA Pediatr. 2021;175(11):1142–1150. <https://doi.org/10.1001/jamapediatrics.2021.2482>. PMID: [34369987](https://pubmed.ncbi.nlm.nih.gov/34369987/)

89. Racine N, Eirich R, Cooke J, Zhu J, Pador P, Dunnewold N, et al. When the Bough Breaks: A systematic review and meta-analysis of mental health symptoms in mothers of young children during the COVID-19 pandemic. Infant Ment Health J. 2022; 43(1):36–54. <https://doi.org/10.1002/imhj.21959>. PMID: [34962649](https://pubmed.ncbi.nlm.nih.gov/34962649/)

90. Rezaei-Hachesu V, Fe’li SN, Maajani K, Hokmabadi R, Golbabaei F. The Global Prevalence of Anxiety, Depression, and Insomnia among Healthcare Workers during the Covid-19 Pandemic: A Systematic Review and Meta-Analysis. Journal of Occupational Health and Epidemiology. 2022; 11(1):48–66. Available from: <https://doi.org/10.52547/johe.11.1.48>

91. Robinson E, Sutin AR, Daly M, Jones A. A systematic review and meta-analysis of longitudinal cohort studies comparing mental health before versus during the COVID-19 pandemic in 2020. J Affect Disord; 2022; 296:567-576.. <https://doi.org/10.1016/j.jad.2021.09.098>. PMID: [34600966](https://pubmed.ncbi.nlm.nih.gov/34600966/)

92. Raoofi S, Pashazadeh Kan F, Rafiei S, Khani S, Hosseinifard H, Tajik F, et al. Anxiety during the COVID-19 pandemic in hospital staff: systematic review plus meta-analysis. BMJ Support Palliat Care. 2021; bmjspcare-2021-003125. <https://doi.org/10.1136/bmjspcare-2021-003125>. PMID: [34312187](https://pubmed.ncbi.nlm.nih.gov/34312187/)

93. Shorey SY, Ng ED, Chee CYI. Anxiety and depressive symptoms of women in the perinatal period during the COVID-19 pandemic: A systematic review and meta-analysis. Scand J Public Health . 2021;49(7):730–740. <https://doi.org/10.1177/14034948211011793>. PMID: [33966511](https://pubmed.ncbi.nlm.nih.gov/33966511/)

94. Salehi M, Amanat M, Mohammadi M, Salmanian M, Rezaei N, Saghazadeh A, et al. The prevalence of post-traumatic stress disorder related symptoms in Coronavirus outbreaks: A systematic-review and meta-analysis. J Affect Disord. 2021; 282:527–538. <https://doi.org/10.1016/j.jad.2020.12.188>. PMID: [33433382](https://pubmed.ncbi.nlm.nih.gov/33433382/)

95. Santabárbara J, Bueno-Notivol J, Lipnicki DM, Olaya B, Pérez-Moreno M, Gracia-García P, et al. Prevalence of anxiety in health care professionals during the COVID-19 pandemic: A rapid systematic review (on published articles in Medline) with meta-analysis. Prog Neuropsychopharmacol Biol Psychiatry. 2021; 107:110244. <https://doi.org/10.1016/j.pnpbp.2021.110244>. PMID: [33453320](https://pubmed.ncbi.nlm.nih.gov/33453320/)

96. Santabarbara J, Idoiaga N, Ozamiz-Etxebarria N, Bueno-Notivol J. Prevalence of anxiety in dental students during the covid-19 outbreak: A meta-analysis. Vol. 18, International Journal of Environmental Research and Public Health. Int J Environ Res Public Health; 2021. 18(20):10978. <https://doi.org/10.3390/ijerph182010978>. PMID: [34682726](https://pubmed.ncbi.nlm.nih.gov/34682726/)

97. Santabárbara J, Ozamiz-Etxebarria N, Idoiaga N, Olaya B, Bueno-Novitol J. Meta-analysis of prevalence of depression in dental students during covid-19 pandemic. Vol. 57, Medicina (Lithuania). 2021; 57(11):1278. <https://doi.org/10.3390/medicina57111278>. PMID: [34833496](https://pubmed.ncbi.nlm.nih.gov/34833496/)

98. Santabárbara J, Olaya B, Bueno-Notivol J, Pérez-Moreno M, Gracia-García P, Ozamiz-Etxebarria N, et al. Prevalence of depression among medical students during the COVID-19 pandemic. A systematic review and meta-analysis. Rev Med Chil. 2021; 149(11):1579-1588. <https://doi.org/10.4067/s0034-98872021001101579>. PMID: [35735320](https://pubmed.ncbi.nlm.nih.gov/35735320/).

99. Santomauro DF, Mantilla Herrera AM, Shadid J, Zheng P, Ashbaugh C, Pigott DM, et al. Global prevalence and burden of depressive and anxiety disorders in 204 countries and territories in 2020 due to the COVID-19 pandemic. Lancet. 2021; 398(10312):1700-1712. <https://doi.org/10.1016/s0140-6736(21)02143-7>. PMID: [34634250](https://pubmed.ncbi.nlm.nih.gov/34634250/)

100. Safi-Keykaleh M, Aliakbari F, Safarpour H, Safari M, Tahernejad A, Sheikhbardsiri H, et al. Prevalence of postpartum depression in women amid the COVID-19 pandemic: A systematic review and meta-analysis. Int J Gynaecol Obstet. 2022; 157(2):240-247. <https://doi.org/10.1002/ijgo.14129>. PMID: [35122433](https://pubmed.ncbi.nlm.nih.gov/35122433/).

101. Saragih ID, Tonapa SI, Saragih IS, Advani S, Batubara SO, Suarilah I, et al. Global prevalence of mental health problems among healthcare workers during the Covid-19 pandemic: A systematic review and meta-analysis.International Journal of Nursing Studies. Int J Nurs Stud; 2021; 121:104002 <https://doi.org/10.1016/j.ijnurstu.2021.104002>. PMID: [34271460](https://pubmed.ncbi.nlm.nih.gov/34271460/)

102. Schafer KM, Lieberman A, Sever AC, Joiner T. Prevalence rates of anxiety, depressive, and eating pathology symptoms between the pre- and peri-COVID-19 eras: A meta-analysis. J Affect Disord. 2022; 298:364–372. <https://doi.org/10.1016/j.jad.2021.10.115>. PMID: [34740748](https://pubmed.ncbi.nlm.nih.gov/35122433/)

103. Sharma S, Joseph J, Dhandapani M, Varghese A, Radha K, Mathews E, et al. COVID-19 and psychological distress among the general population of India: Meta-Analysis of observational studies. Indian J Community Med. 2022; 47(2):160-165. <https://doi.org/10.4103/ijcm.ijcm_1365_21>. PMID: [36034249](https://pubmed.ncbi.nlm.nih.gov/36034249/)

104. Sideli L, lo Coco G, Bonfanti RC, Borsarini B, Fortunato L, Sechi C, et al. Effects of COVID-19 lockdown on eating disorders and obesity: A systematic review and meta-analysis . Eur Eat Disord Rev. 2021; 29(6): 826–841. <https://doi.org/10.1002/erv.2861>. PMID: [34460991](https://pubmed.ncbi.nlm.nih.gov/34460991/)

105. Singh RK, Bajpai R, Kaswan P. COVID-19 pandemic and psychological wellbeing among health care workers and general population: A systematic-review and meta-analysis of the current evidence from India. Clin Epidemiol Glob Health. 2021; 11:100737. <https://doi.org/10.1016/j.cegh.2021.100737>. PMID: [33898866](https://pubmed.ncbi.nlm.nih.gov/33898866/)

106. Ślusarska B, Nowicki GJ, Niedorys-Karczmarczyk B, Chrzan-Rodak A. Prevalence of Depression and Anxiety in Nurses during the First Eleven Months of the COVID-19 Pandemic: A Systematic Review and Meta-Analysis. Int J Environ Res Public Health. 2022; 19(3):1154. <https://doi.org/10.3390/ijerph19031154>. PMID: [35162183](https://pubmed.ncbi.nlm.nih.gov/35162183/)

107. Sun P, Wang M, Song T, Wu Y, Luo J, Chen L, et al. The Psychological Impact of COVID-19 Pandemic on Health Care Workers: A Systematic Review and Meta-Analysis. Front Psychol. 2021; 12:626547. <https://doi.org/10.3389/fpsyg.2021.626547>. PMID: [34305703](https://pubmed.ncbi.nlm.nih.gov/34305703/)

108. Tomfohr-Madsen LM, Racine N, Giesbrecht GF, Lebel C, Madigan S. Depression and anxiety in pregnancy during COVID-19: A rapid review and meta-analysis. Psychiatry Res. 2021; 300:113912. <https://doi.org/10.1016/j.psychres.2021.113912>. PMID: [33836471](https://pubmed.ncbi.nlm.nih.gov/33836471/)

109. Varghese A, George G, Kondaguli S v., Naser AY, Khakha DC, Chatterji R. Decline in the mental health of nurses across the globe during COVID-19: A systematic review and meta-analysis. J Glob Health. 2021; 10;11:05009. <https://doi.org/10.7189/jogh.11.05009>. PMID: [33884193](file:///C:\Users\awn233\AppData\Roaming\Microsoft\Word\33884193)

110. Wang C, Wen W, Zhang H, Ni J, Jiang J, Cheng Y, et al. Anxiety, depression, and stress prevalence among college students during the COVID-19 pandemic: A systematic review and meta-analysis. J Am Coll Health. 2021; 1–8. <https://doi.org/10.1080/07448481.2021.1960849>. PMID: [34469261](https://pubmed.ncbi.nlm.nih.gov/34469261/)

111. Xie Q, Liu XB, Xu YM, Zhong BL. Understanding the psychiatric symptoms of COVID-19: a meta-analysis of studies assessing psychiatric symptoms in Chinese patients with and survivors of COVID-19 and SARS by using the Symptom Checklist-90-Revised. Translational Psychiatry. 2021; 11(1):290. <https://doi.org/10.1038/s41398-021-01416-5>. PMID: [34001863](https://pubmed.ncbi.nlm.nih.gov/34001863/)

112. Xiong N, Fritzsche K, Pan Y, Löhlein J, Leonhart R. The psychological impact of COVID-19 on Chinese healthcare workers: a systematic review and meta-analysis. Soc Psychiatry Psychiatr Epidemiol. 2022; 57(8):1515-1529. <https://doi.org/10.1007/s00127-022-02264-4>. PMID: [35325261](https://pubmed.ncbi.nlm.nih.gov/35325261/)

113. Yan H, Ding Y, Guo W. Mental Health of Medical Staff during the Coronavirus Disease 2019 Pandemic: A Systematic Review and Meta-Analysis. Psychosom Med. 2021; 83(4):387–396. <https://doi.org/10.1097/psy.0000000000000922>. PMID: [33818054](https://pubmed.ncbi.nlm.nih.gov/33818054/)

114. Yan Y, Du X, Lai L, Ren Z, Li H. Prevalence of depressive and anxiety symptoms among Chinese older adults during the COVID-19 pandemic: A systematic review and meta-analysis. J Geriatr Psychiatry Neurol. 2022; 35(2):182–95. <https://doi.org/10.1177/08919887221078556>. PMID: [35245999](https://pubmed.ncbi.nlm.nih.gov/35245999/)

115. Yang F, Wen J, Huang N, Riem MME, Lodder P, Guo J. Prevalence and related factors of child posttraumatic stress disorder during COVID-19 pandemic: A systematic review and meta-analysis. Eur Psychiatry. 2022; 65(1):e37. <https://doi.org/10.1192/j.eurpsy.2022.31>. PMID: [35726735](PMID:%2035726735)

116. Yunitri N, Chu H, Kang XL. Global prevalence and associated risk factors of posttraumatic stress disorder during COVID-19 pandemic: A meta-analysis. Int J Nurs Stud. 2022; 126:104136. <https://doi.org/10.1016/j.ijnurstu.2021.104136>. PMID: [34856503](https://pubmed.ncbi.nlm.nih.gov/34856503/)

117. Zhang H, Li W, Li H, Zhang C, Luo J, Zhu Y, et al. Prevalence and dynamic features of psychological issues among Chinese healthcare workers during the COVID-19 pandemic: A systematic review and cumulative meta-analysis. Gen Psychiatr. 2021; 34(3):e100344. <https://doi.org/10.1136/gpsych-2020-100344>. PMID: [34192242](https://pubmed.ncbi.nlm.nih.gov/34192242/)

118. Zhang L, Pan R, Cai Y, Pan J. The prevalence of post-traumatic stress disorder in the general population during the COVID-19 pandemic: A systematic review and single-arm meta-analysis. Psychiatry Investig. 2021;18(5):426–433. <https://doi.org/10.30773/pi.2020.0458>. PMID: [33910325](https://pubmed.ncbi.nlm.nih.gov/33910325/)

119. Zhang SX, Chen RZ, Xu W, Yin A, Dong RK, Chen BZ, et al. A Systematic Review and Meta-Analysis of Symptoms of Anxiety, Depression, and Insomnia in Spain in the COVID-19 Crisis. Int J Environ Res Public Health. 2022; 19(2):1018. <https://doi.org/10.3390/ijerph19021018>. PMID: [35055841](https://pubmed.ncbi.nlm.nih.gov/35055841/)

120. Zhang SX, Batra K, Xu W, Liu T, Dong RK, Yin A, et al. Mental disorder symptoms during the COVID-19 pandemic in Latin America - a systematic review and meta-analysis. Epidemiol Psychiatr Sci. 2022; 31:e23. <https://doi.org/10.1017/s2045796021000767>. PMID: [35438066](https://pubmed.ncbi.nlm.nih.gov/35438066/)

121. Zhang SX, Miller SO, Xu W, Yin A, Chen BZ, Delios A, et al. Meta-analytic evidence of depression and anxiety in Eastern Europe during the COVID-19 pandemic. Eur J Psychotraumatol. 2022; 13(1):2000132. <https://doi.org/10.1080/20008198.2021.2000132>. PMID: [35186214](https://pubmed.ncbi.nlm.nih.gov/35186214/)

122. Zhao YJ, Jin Y, Rao WW, Li W, Zhao N, Cheung T, et al. The prevalence of psychiatric comorbidities during the SARS and COVID-19 epidemics: a systematic review and meta-analysis of observational studies. J Affect Disord. 2021;287:145–57. <https://doi.org/10.1016/j.jad.2021.03.016>. PMID: [33799032](https://pubmed.ncbi.nlm.nih.gov/33799032/)

123. Zhu J, Racine N, Xie EB, Park J, Watt J, Eirich R, et al. Post-secondary Student Mental Health During COVID-19: A Meta-Analysis. Front Psychiatry. 2021; 12:777251. <https://doi.org/10.3389/fpsyt.2021.777251>. PMID: [34955924](https://europepmc.org/article/pmc/pmc8709535)
