# Supplementary material for: COVID-19 and common mental health symptoms in the early phase of the pandemic: An umbrella review of the evidence
Source: PLoS Med. 2023 Apr 25;20(4):e1004206. doi: 10.1371/journal.pmed.1004206 (PMC10129001; doi:10.1371/journal.pmed.1004206)
Supplement: S2 Text — Table A. Pooled prevalence rates and changes in depression symptoms, with heterogeneity scores and main subgroup findings. Table B. Pooled prevalence rates and changes in anxiety symptoms, with heterogeneity scores and main subgroup findings. Table C. Pooled prevalence rates and changes in PTSD symptoms, with heterogeneity scores and main subgroup findings. Fig 1. PRISMA flow chart initial search December 31, 2019 until October 6, 2021. Fig 2. PRISMA flow chart updated search October 7, 2021 until August 12, 2022. (DOCX) [file pmed.1004206.s003.docx]

**Contents**

[**Table A.** Pooled prevalence rates and changes in depression, heterogeneity and subgroup or meta-regression findings. 2](#_Toc128933072)

[**Table B.** Pooled prevalence rates anxiety, heterogeneity and subgroup or meta-regression findings. 9](#_Toc128933073)

[**Table C.** Pooled prevalence rates PTSD, heterogeneity and subgroup or meta-regression findings. 16](#_Toc128933074)

[**Figure 1**: PRISMA flow chart initial search Dec. 31^st^, 2019 until Oct. 6^th^, 2021 19](#_Toc128933075)

[**Figure 2**: PRISMA flow chart updated search Oct. 7^th^, 2021 until August 12^th^, 2022. 20](#_Toc128933076)

[**References** 21](#_Toc128933077)

## **Table A.** Pooled prevalence rates and changes in depression, heterogeneity and subgroup or meta-regression findings.

| **Study** | **K / n depression** | **Pooled prevalence / change (95% CI)** | **I^2^ (%)** | **Subgroup- or meta-regression results depression prevalence** |
| --- | --- | --- | --- | --- |
| **General Population** | | | | |
| Alzahrani 2022 (1) | 10/NR | 30 (22 to 38) | 99.5 | Differences in pooled prevalence for BDI, DASS-21, HADS, and PHQ-9 was 59%, 25%, 37% , and 26%. |
| Arora 2022 (2) | 11/NR | 22 (13 to 33) | 99.7 | Subgroup analysis: no effect of region, type of workers, population, timing of COVID and month of publication on prevalence. |
| Bareeqa 2021 (3) | 15/49,656 | 26.9 (20.0 to 34.3) | NR | Higher in females; no large difference between scale (PHQ-9; SDS). |
| Balakrishnan 2022 (4) | 33/130481 | 34 (27 to 42) | 99.8 | No significant differences in depression levels were observed between the timelines, cohorts (HCW, GP, Students) and regions. |
| Bello 2022 (5) | 42/33805 | 48.0 (39.0 to 57.0) | 99.5 | Highest prevalence was reported for studies conducted in Egypt (67%), whereas studies conducted in Nigeria (31%) and Kenya (31%) reported the lowest pooled prevalence rates. |
| Castaldelli-Maia 2021 (6) | NR/193137 | 21.3 (19.0 to 23.6) | 99.4 | Across populations: higher when more previous depression, older data, in other regions than Asia. |
| Cenat 2021 (7) | 28/NR | 17 (13 to 22) | 99.6 | No differences were found between countries. |
| Cenat (8) | 26 / NR | 26.8 (23.7 to 29.9) | 99.9 | Sign. higher prevalence in North America in comparison with Europe, Latin America and Asia-Pacific. Significant difference across measures used. Meta-regression: prevalence rate of depression in females is positively associated with the overall pooled prevalence of depression. Measures were also sign associated with prevalence. |
| Cenat (8) | 14 / NR | 2.7 (1.7 to 3.6)^5^ | 97.8 | Pooled prevalence depression across all time-points: sig. differences underlying **the measures and regions** and a higher depression rate was reported in the waves conducted in North America (d = 4.76) and in waves that used the Zung Self-Rating Depression (d = 7.46). Meta-regression showed that months (B = −0.93) are associated with the pooled effect size of depression. |
| Chen, Farah, 2021 (9) | 24/12688 | 45 (38 to 53) | 99.4 | Subgroups based on population, frontline HCWs vs. general HCWs vs. medical students vs. GP; on symptom severity, mild vs. moderate vs. severe; on region, sub-Saharan Africa v. North Africa; on study quality, medium quality vs. high quality; and on data collection time, higher first six months vs. after six months. |
| Dal Santo 2022 (10) | 4 / 4475 | 0.01 (-0.1 to 0.3)^1^ | 96.0 | NR |
| Deng 2021 (11) | NR/16094 | 26.0 (17.0 to 36.0) | 99.5 | Prevalence increased over time in varying degrees. |
| De Sousa 2021 (12) | 18/NR | 26.7 (22.3 to 31.6) | 99.8 | Between-population subgroup analyses were performed (GP vs. HCW) for depression. No differences were found. |
| Delanerolle 2022 (13) | NR/NR | 27.6 (23.4 to 32.5) | 99.8 | NR |
| Dragioti 2022 (14) | 201/NR | 20 (18 to 21) | 99 | Females higher prevalence rates in almost all examined mental health problems; younger strata of the population (students) had higher prevalence rates of depressive symptoms. Depression symptoms more prevalent in low‐/middle‐income countries. |
| Fan 2021 (15) | 108/515452 | 27.0 (25.0 to 29.0) | 99.6 | NR |
| Hosen 2021 (16) | 6/NR | 41 (33 to 50) | 98.46 | Age and gender were found to be significant predictors of mental health problems in a large number of studies; level of education in some studies. |
| Hossain 2021 (17) | NR/NR | 34.1 (28.9 to 39.4) | 99.4 | Higher in females; related to instrument used; and in studies with high risk of bias. |
|  |  |  |  |  |
| Knox 2022 (18) | 27/NR | 0.83 (0.30 to 1.37)^5^ | 99.88 | Moderator/subgroup analyses: type and length of social restriction, WHO region, age, pre-existing physical or mental health vulnerabilities. Depression was significantly higher in people exposed to strict compared to moderate social restrictions, in European regions, in people who reported no pre-existing physical or mental health conditions, and among people under age 18 or between 31 and 59 years. |
| Kunzler 2021 (19) | 25/60213 | 0.67 (0.07 to 1.27)^1^ | 100 | Higher for specific assessment tools and older comparative data. |
| Lee 2021 (20) | 114/640037 | 21.4 (19.3 to 23.5) | 100 | Depression moderated by stringent government response with lockdown. |
| X. Liu 2021 (21) | 23 / NR | 25.3 (20.4 to 32.0) | NR | Higher in females. |
| Necho 2021 (22) | 8 / NR | 34.3 (18.4 to 50.2) | 94.6 | Prevalence differed according to scale used. |
| Nochaiwong 2021 (23) | 75/ 280607 | 28.0 (25.0 to 31.2) | 99.7 | Prevalence associated with WHO region, female gender inequality index; hospital beds per 10,000 people, surveyed country (China vs. non-China). |
| Pappa 2022 (24) | 3/NR | 16.0 (6.0 to 29.0) | 99.7 | NR |
| Phiri 2021 (25) | 66 / NR | 22.6 (20.0 to 25.1) | 99.8 | Prevalence greater under age ≤30 years. |
| Prati and Mancini (26) | 10 / NR | 0.15 (0.01 to 0.30)^2^ | 94.0 | No significant moderation effects (age, gender, continent, COVID-19 death rate, days of lockdown, publication status or study design). |
| Robinson 2022 (27) | 35 / NR | 0.22 (0.13 to 0.29)^3^ | 95.0 | Largest changes among studies that sampled participants in the early stages of the pandemic. Country level factors (e.g. number of COVID cases) and age, gender and region did not explain heterogeneity in analyses of depression symptoms. |
| Santomauro 2021 (28) | 46 / NR | 27.6 (25.1 to 30.3)^6^ | NR | Daily SARS-CoV-2 infection rates and reductions in human mobility were associated with increased prevalence of major depressive disorder symptoms. Females were affected more by the pandemic than males for major depressive disorder and younger age groups were more affected than older age groups. |
| Schafer 2022 (29) | 13/36 | 18.3 (13.5 to 24.3) | NR | No significant differences in prevalence according to age, gender or race. |
| Singh 2021 (30) | 2 / NR | 24.9 (22.0 to 27.8) | NR | Too few studies. |
| Yan et al 2022 (31) | 7/10,229 | 26 (16 to 40) | 99.5 | No significant differences were found between older adults from different regions and between prevalence estimated in different phases of the pandemic. |
| Zhang, Chen 2022 (32) | NR/NR | 20 (16 to 25) | 84- 99 | Studies with high quality reported lower prevalence of mental health symptoms. |
| Zhang, Batra 2022 (33) | 49 / 139 559 | 33 (28 to 37) | NR | Prevalence rates higher in South America than in Central America and higher among frontline HCWs and students than among the general population and general HCWs. |
| Zhang, Miller 2022 (34) | NR /  NR | 20 (13 to 27) | NR | Prevalence rates bit higher in EU Eastern European countries than non-EU eastern European countries. |
| Zhao 2021 (35) | 10 / 20644 | 23.2 (16.6 to 31.4) | 99.4 | Higher in females and in studies reporting higher quality scores. |
| **Health Care Workers** | | | | |
| Abdulla 2021 (36) | 5/5796 | 41.90 (29.17 to 54.64) | 99 | NR |
| Afridi 2022 (37) | 10/12507 | 25.5 (19.5 to 32.5) | 95.8 | NR |
| Aymerich 2022 (38) | 160/210762 | 33 (28 to 38) | 99.9 | NR |
| Bareeqa 2021 (3) | 8/10267 | 31.5 (20.7 to 43.5) | 99.3 | NR |
| Balakrishnan 2022 (4) | 23/29470 | 34 (29 to 39) | 99.4 | No significant differences in depression levels were observed between the timelines, cohorts (HCW, GP, Students) and regions |
| Castaldelli-Maia 2021 (6) | 16/NR | 19.1 (14.7 to 23.5) | 98.2 | Across populations: higher when more previous depression, older data,  in other regions than Asia and Europe. |
| Cenat 2021 (7) | 18/NR | 14 (11 to 17) | 98.2 | No differences were found between countries |
| Chekole and Abate 2021 (39) | NR | 29.5 (15.3 to 43.7) | 99.7 | Prevalence highest among students followed by patients. |
| Ching et al., 2021 (40) | 98/103628 | 37.5 (33.8 to 41.3) | 99.5 | Women and nurses were at increased risk of having depression or anxiety |
| Y. Deng 2021 (11) | 20 / 11438 | 31.0 (25.0 to 37.0) | 98.4 | High between study heterogeneity likely due to inconsistent scales and cutoffs. Higher prevalence of depression before the COVID-19 peak than after (Feb, 8, 2020). |
| De Sousa, 2021(12) | 18/NR | 27.1 (23.1 to 31.4) | 99.9 | Between-population subgroup analyses were performed (GP vs. HCW) for depression and anxiety but not PTSD. No differences were found |
| Da Silva, 2021 (41) | 7/7,102 | 33 (24 to 45) | 99.5 | NR |
| Delanerolle 2022 (13) | NR/NR | 27.7 (23.2 to 33.1) | 99.8 | NR |
| F. Dong 2021 (42) | 18 / NR | 31.1 (24.5 to 37.7) | 92.2 | High heterogeneity: small sample capacity, non-random sampling, different scales. |
| Dragioti (14) | 106/NR | 23 (19 to 28) | 98 | Females higher prevalence rates in almost all examined mental health problems; younger strata of the population (students) had higher prevalence rates of depressive symptoms. Depression symptoms more prevalent in low‐/middle‐income countries. |
| Dutta 2021 (43) | 30 / 37655 | 32.4 (25.9 to 39.3) | 99.0 | High heterogeneity for assessment tools. |
| El-Qushayri 2021 (44) | 5 / NR | 65.5 (46.9 to 80.3) | 98.0 | High heterogeneity; lower in some groups of disease severity. |
| Fan 2021 (15) | 23 / NR | 32.3 (NR) | NR | NR |
| Ghahramani 2022 (45) | 5/NR | DASS 36 (12 to 70) | 99.3 | Pooled prevalence of depression was not highly varied across different questionnaires. |
|  | 2/NR | PHQ-2 30 (8 to 66) | 99.5 | Anxiety more prevalent in physicians, nurses and older people, and in studies conducted in China. |
|  | 11/NR | PHQ-9 36 (24 to 50) | 99.5 | A significant relationship was revealed between age and sample size and mental health outcomes. |
| Halemani 2021 (46) | 12/3315 | 34 (23 to 45) | 97.61 | Doctors |
|  | 13/4167 | 42 (32 to 52) | 97.60 | Nurses. Studies had low risk and acceptable heterogeneity between studies. |
| Hao 2021 (47) | 14 / NR | 24.1 (16.2 to 32.1) | 99 | Female and second line workers higher depression and frontline workers higher depression severity. |
| Hosen 2021 (16) | 4/NR | 41 (35 to 46) | 62.10 | Age and gender were found to be significant predictors of mental health problems in a large number of studies; education in some studies. |
| Hossain 2021 (17) | NR | 29.9 (23.9 to 36.2) | 98.1 | Prevalence higher when more females in samples and when studies had high risk of bias; prevalence rates varied across commonly used scales. |
| Hu 2022 (48) | 56/89390 | 29 (23 to 35) | 100 | Subgroup analyses: the working type (first-line vs non-first-line medical staff) is an important factor in the mental problems of medical staff. Meta-regression analyses: mean age and sex had no significant effect on depression as predictors. |
| Johns 2021 (49) | 26/31447 | 21 (16 to 25) | 98.93 | Subgroup analyses: region, GDP per capita (significant), doctors per 100K. Between-group heterogeneity was not significant when analysed by measure, severity threshold, survey timeframe, or risk of bias. |
| Kunzler 2021 (19) | 7 / 2226 | -0.16 (−0.59 to 0.26)^1^ | 97.0 | Specific assessment tools and older comparative data associated with increased mental burden. Level of COVID-19 patient contact did not affect the mental health impact. |
| Li 2021 (50) | 55 / NR | 21.7 (18.3 to 25.2) | 99.3 | NR |
| Liu 2021 (51) | 17 / NR | 31.0 (25.0 to 37.0) | 95.0 | NR |
| Liu 2021 (21) | 20 / NR | 25.8 (20.4 to 31.0) | NR | NR |
| Mahmud 2021 (52) | 69 / 144649 | 37.1 (31.8 to 42.4) | 99.8 | High heterogeneity and prevalence rates for depression associated with later data collection and lower sample size. |
| Marvaldi 2021 (53) | 25/68 030 | 31.1 (25.7 to 36.8) | 99.5 | High heterogeneity for depression not associated with high quality criteria study, gender, location, type of scale or location. |
| Norhayati 2021 (54) | 60/132308 | 34.6 (30.9 to 38.4) | 100 | Western Asia, frontline healthcare providers, females, and nurses showed highest prevalence of depression. |
| Olaya 2021 (55) | 46/NR | 24 (20 to 28) |  | HCW overall (significant heterogeneity between prevalence rates in nurses (25%), doctors (24% and frontline healthcare workers (43%); prevalence of depression was lower in studies using the DASS-21, those carried out in China, studies using convenience sampling methods and those of high methodological quality (Table |
| Pappa 2022 (24) | 4/NR | 14 (5 to 25) | 98.5 | Frontline HCW: NR |
|  | 11/ NR | 15 (10 to 21) | 97.5 | General HCW: NR |
| Phiri 2021 (25) | 66 / NR | 23.4 (20.6 to 26.3) | 99.5 | Not much difference in the prevalence by study quality among healthcare professionals. |
| Rezaei-Hachesu, 2022 | 7/1362 | 35 (19 to 55) | 99.01 | Pooled prevalence rate of depression by severity subgroups was obtained as 25% in mild depression, 20% in moderate depression, 12% in severe depression, and 7% in very severe depression. The pooled prevalence rate of depression was calculated as 46% in nurses and as 17% among all HCWs. |
| Saragih 2021 (56) | 30 / NR | 37 (29 to 45) | 99.7 | NR |
| Singh 2021 (30) | 8 / NR | 32.7 (24.6 to 41.3) | 93.3 | NR |
| Ślusarska (57) | 18/39430 | 22 (15 to 30) | 99.7 | Subgroup analysis by tool: PHQ-9 (23%) not sign. higher than other tools (21%). Meta-regression: prevalence related with gender but not with marital status. Subgroup analysis by area: Asia 18% while other continents 31%. Subgroup analysis by position: frontline 19% versus mixed 25%. Not significant. |
| Sun 2021 (58) | 39 / NR | 36 (31 to 41) | 99.6 | NR |
| Varghese 2021 (59) | 17 / NR | 32 (21 to 44) | 99.4 | NR |
| Xiong et al 2022 (60) | 13/48,621 | 15 (13 to 16) | NR | The prevalence of moderate to severe depression was higher in nurses than technicians or others and higher in HCW from Wuhan than those from other provinces in China. Only the differences between locations could partly account for the heterogeneity. |
| Yan 2021 (61) | 20 / 21038 | 27 (20 to 34) | 99.0 | Lower prevalence in Wuhan than other survey sites and lower in frontline versus non-frontline staff. |
| Zhang 2021 (62) | 18 / 18818 | 26.2 (20.8 to 32.5) | 98.6 | NR |
| Zhao 2021 (35) | 11/ 11922 | 23.9 (15 to 35.9) | 99.3 | Depression significantly associated with higher proportion of females and higher quality-studies. |
| **Patients (COVID-19/other)** |  |  |  |  |
| Ayubi 2021 (63) | 15 / NR | 37 (27 to 47) | 99 | Cancer patients. NR |
| Castaldelli-Maia 2021 (6) | 6 / NR | 22.2 (12.6 to 31.8) | 96.6 | Mixed patients. Prevalence higher when previous depression; older study data; patient studies; other regions than Asia and Europe. |
| Chekole and Abate 2021 (39) | 3 / NR | 28.4 (0.87 to 55.8) | 99.5 | Covid patients. NR |
| Dong 2021 (64) | 27 / 6200 | 37.7 (29.3 to 46.2) | 98.3 | Covid patients. Higher prevalence in more clinical severe COVID-19 patients and according to time of data collection during first half year of pandemic. |
| Dragioti (14) | 29/NR | 28 (21 to 36) | 96 | Patients Covid-19: Females had higher prevalence rates in almost all examined mental health problems. Estimates of almost all examined mental health problems were higher in people affected by COVID‐19 infection or who had close contact with COVID‐19‐ infected people. |
| Dragioti (14) | 4/NR | 37 (24 to 51) | NR | Patient (somatic): females had higher prevalence rates in almost all examined mental health problems inc dep. |
| Fan 2021 (15) | 13 | 18.6 (11.2 to 29.4) | 99.6 | Covid patients. NR |
| Khraisat 2022 (65) | 5/837 | 55 (21 to 86) | 99 | Patients with eating disorders. |
| Kuroda 2021 (66) | 18/6252 | 31 (23 to 39) | 97 | Patients with epilepsy. Subgroup analyses: questionnaire, continents, income country, early/late pandemic phases. |
| Kunzler 2021 (19) | 7 / 2138 vs 24444 | 0.48 (-0.08 to 1.04) ^1^ | 98.0 | Mixed patients (covid, physical and mental disorders). Specific assessment tools and older comparative data associated with increased mental burden. |
| Lee K 2022 (67) | 4/950 | 17 (4 to 30) | 97.7 | People living with HIV. |
| Liu 2021 (68) | 20 / NR | 38 (25 to 51) | 98.0 | Higher prevalence in females, in acute SARS-CoV-2 patients, in those with mild symptom severity, small difference in prevalence between high vs low quality. Heterogeneity largely unexplained: variance in prevalence due to screening tool. |
| Liu 2021 (21) | 4 / NR | 55.4 (32.8 to 76.0) | 98 | Higher prevalence in COVID-19 patients (particularly confirmed) compared to other groups (e.g. students), lower in China. Additionally, different measurement tools resulted in varying results. |
| Natarajan 2022 (69) | 6/NR | 22.4 (10.22 to 49.35) | 97.8 | Long COVID patients. NR |
| Premraj, 2022 (70) | 8/480 | 16.7 (9.7 to 23.7) | 97.63 | Depression was more frequently reported in the community than in patients hospitalised during their acute phase of COVID-19 (27% vs 12%). Depression prevalence higher when assessed at or beyond six months (long-term) than when assessed between three and six months (mid-term). Patients hospitalised during acute COVID-19 less likely to develop depression 3 months (or more) after COVID-19 onset than those who remained non-hospitalised throughout their acute infection. Depression approximately two times more prevalent in cohorts with >20% of participants admitted to an ICU (during acute COVID-19) as in cohorts in which <20% underwent ICU admission. |
| Sideli 2021 (71) | 4 / NR | 55 (39 to 70) | 91.6 | Higher symptoms in individuals with self-reported compared to clinical patients with eating-disorders. |
|  | 3 / NR | 51 (18 to 85) | 99.0 | NR |
| Xie 2021 (72) | 10 / 1655 | 2.08^4^ (1.7 to 2.5) | 99.9 | Acute COVID-19 stage: NR |
|  | 2 / 30 | 1.66^4^ (0.8 to 2.5) | 97.0 | Early COVID-19 recovery stage: NR |
| Yan et al 2022 (31) | 5/137 | 27 (14 to 48) | 75 | Older adults COVID-19: heterogeneity in estimated prevalence but no significant difference between studies using different criteria or between patients from different regions. Although the prevalence of depressive symptoms for older Covid-19 patients was higher during Phase 2 (March-April 2020) of the pandemic than in Phase 3 (May-August 2020) and in Phase 1 (January-February 2020), only a marginally significant difference was found. |
|  | 2/669 | 61 (31 to 84) | 97.5 | Older adults with COVID 19 and with chronic disease not reported because the number of studies (k=2) was too small to conduct quantitative subgroup analyses. |
| **Pregnant women** |  |  |  |  |
| Adrianto 2022 (73) |  |  |  |  |
| Pregnant subsample | 36/NR | 31.4 (27 to 36) | 99.5 | NR |
| Postnatal subsample | 19/NR | 27.6 (22 to 34) | 98.4 | NR |
| Chen 2022 (74) | 8/6,480 | 34 (21 to 46) | 98 | NR |
| Cevik 2022 (75) | 26 / NR (before)  4 / NR (during) | 23.1 (15.4 to 33.2)  40.4 (33.5 to 47.7) | NR | Moderator variable depression prevalence ‘before versus during the pandemic’ was not statistically significant. |
| Dragioti 2022 (14) | 7/NR | 34 (28 to 40) | 99 | Females had higher prevalence rates in almost all examined mental health problems. Depression more prevalent in low‐/middle‐income countries. |
| Tomfohr-Madsen 2021 (76) | 37 / 47,677 | 25.6 (21.8 to 29.9) | 98.6 | NR |
| Safi-Keykaleh (77) | 24 / NR | 28 (23 to 33) | 98.5 | Prevalence of PPD significantly different according to cut-off score of Edinburgh Postpartum Depression Scale. |
| Demissie 2021 (78) | 14 / 18335 | 27 (9 to 45) | 99.3 | Subgroup analyses gave null results |
| Ghazanfarpour 2021 (79) | 11 / NR | 25.1 (18 to 33) | 97 | NR for depression |
| Shorey 2021 (80)  Pregnant subsample | 13 / 14802 | 27 (20 to 33) | 99 | Prevalence estimates depressive symptoms not significantly different when stratified by geographical regions. |
| Perinatal subsample | 4 / 1819 | 27 (14 to 40) | 97 |  |
| Postnatal subsample | 5/4845 | 17 (10 to 24) | 96 |  |
| **Young people** |  |  |  |  |
| **Students** |  |  |  |  |
| Balakrishnan 2022 (4) | 15/36423 | 34 (25 to 42) | 99,8 | No significant differences in depression levels were observed between the timelines, cohorts (HCW, GP, Students) and regions |
| **Batra** (81) | 14 / 61,392 | 31.2 (19.7 to 45.6) | 99.8 | Higher prevalence of depression in lower quality studies, in areas other than Asia/China, in females, in assessment tools like PHQ. |
| Castaldelli-Maia 2021 (6) | 11 / 3841 | 25.9 (17.6 to 34.2) | 99.7 | Prevalence higher when previous depression; older study data; patient studies; other regions than Asia and Europe. |
| Chang 2021 (82) | NR/ 135,018 | 34 (27 to 41) | 99.9 | Prevalence differed among countries, and higher pooled depressive symptom prevalence of females. |
| Chekole 2021 (39) | 2 / NR | 50.2 (-2.6 to 103) | 99.8 | NR |
| Deng 2021 (83) | 52 / 1,277,755 | 34 (30 to 38) | 100 | Differences in prevalence estimates between validated versus unvalidated scales, different screening tools and cutoff values used and positive correlations pooled depressive symptoms  and study date and mean age. |
| Ebrahim 2022 (84) | 12/23,927 | 23.2 (15.7 to 32.9) | 99.5 | NR |
| Fang 2022 (85) | 75/1005228 | 32 (28 to 37) | 100 | Pooled prevalence of depression in the student population was influenced by gender, the period of the epidemic, the region, the stage of education, the student’s major, and the instrument used in the evaluation. The prevalence of depression was higher among females 36.0% than males in the student population. Prevalence of depressive symptoms higher in undergraduate and postgraduate students than in primary school and middle school students. In addition, non-medical students had higher prevalence of depression than medical students. |
| Guo 2021 (86) | 10 / 24234 | 25 (17 to 33) | 99 | Mild depression: no significant difference between genders. |
| Hosen 2021 (16) | 6/NR | 65 (53 to 76) | 96.11 | Age and gender were found to be significant predictors of mental health problems in a large number of studies; education in some studies. |
| Jia 2022 (87) | 31/29036 | 38 (31 to 45) | 99 | Subgroup analyses: differences in assessment tool, country, gender. |
| Li 2021 (88) | 63317/18 | 39 (27 to 51) | 99.9 | Higher prevalence in non-Chinese vs. Chinese students and in surveys conducted after March 1 compared with surveys before March 1, 2020. |
| Luo 2021 (89) | 84 /1292811 | 26 (23.3 to 28.9) | 99 | Higher prevalence in studies conducted during the late stage of the COVID-19 outbreak than during the early stage, in studies with more females, in studies with specific screening tools or lower cut-offs for depression. |
| Oliveira Carvalho 2022 (90) | 13/NR | 63 (52 to 73) | 99 | Seven studies used the PHQ-9 obtaining a higher pooled prevalence rate 69% (95% CI 56-82), than the other four studies that used other questionnaires 48% (95%CI 25-78). |
| Pappa 2022 (24) | 1/NR | 23 (10 to 39) | NR | NR |
| Santabarbara (2021) (91) | 11 | 31 (23 to 40) | 98.0 | Meta-regression: gender, age, response rate, methodological quality and sampling methods not significant. Asia/China lower prevalence than other continents/countries. Higher prevalence PHQ-9 than DASS-21. |
| Santabarbara (2021) (92) | 13 | 37 (26 to 49) | 98.3 | Higher prevalence of depression for studies conducted in Asia (40%) vs. Europe or America (29%). Higher prevalence of depression for studies using the PHQ-9 compared to those using the DASS or HADS and those using random or cluster sampling methods compared to those using convenience sampling method. |
| Makwana 2022 (93) | 6 / 3248 | 38 (27 to 49) | 97 | NR |
| Mulyadi 2021 (94) | 8/8175 | 52 (40 to 63) | 98 | NR |
| Wang 2021 (95) | 19 / > 83,699 | 37 (32 to 42) | 99.9 | Higher prevalence in non-Chinese versus Chinese students. |
| Zhu 2021 (96) | 126/ NR (total 1,732,456) | 30.6 (27.4 to 34) | 99.9 | Significant moderators geographical region and month of data collection. Clinical depression lower in studies East Asia compared to all other regions and for every month increase a 0.16% increase in depression prevalence. Age, sex, type of student, level of training, or study quality were no significant moderators for the prevalence of depression symptoms among students during the COVID-19 pandemic. |
| **Children and adolescents** |  |  |  |  |
| Chen 2022 (97) | 13/41,729 | 28.6 (21.7 to 35.5) | 99.6 | Pooled prevalences were higher for PHQ-9 than DSRSC and for studies with primary school students than studies excluding primary school students. |
| Chai 2021 (98) | 9/NR | 22 (16 to 30) | 99 | Gender (percentages of boys higher – lower prevalence) had an effect on symptoms. |
| Ma 2021 (99) | 12 / NR | 29 (17 to 40) | 99.9 | Higher in adolescents and females compared to children and males, respectively. |
| Panda 2021 (100) | 11/ 21 330 | 42.3 (39.4 to 45.7) | 36 | NR |
| Racine 2021 (101) | 26 / 79305 | 25.2 (21 to 30) | 99.5 | Higher in studies collected later in the pandemic and in girls; higher symptoms in older children. |
| **Caregivers** |  |  |  |  |
| Dragioti 2022 (14) | 5/NR | 21 (12 to 31) | 99 | Females had higher prevalence rates in almost all examined mental health problems. Prevalence estimates of almost all examined mental health problems were higher in people who had close contact with COVID‐19‐ infected people such as caregivers. |
| Racine 2022 (102) | 16/6520 | 26.9 (21.3 to 33.4) | 96.52 | Mothers of young children: prevalence of clinically significant depression was higher in studies from Europe and North America compared to studies from South America, the Middle East, or Asia (*p* < .001). Prevalence rates varied as a function of maternal age with depressive symptoms being higher in studies with older mothers. Studies that had higher percentages of individuals who were racially minoritized had lower depressive symptom prevalence rates. |
| Panda 2021 (100) | 3/ 257 | 27.4 (19.4 to 35.8) | 34 | NR |
| **Working population** |  |  |  |  |
| Dragioti (14) | 19/NR | 22 (13 to 31) | 99 | Females had higher prevalence rates in almost all examined mental health problems; younger strata of the population had higher prevalence rates of depressive symptoms. |
| Huang 2022 (103) | 14/7504 | 31 (21 to 41) | 97 | Subgroup analyses first responders: type of interview, study quality, sample size, and study type were significant moderator variables while country status, setting, and assessment tool were not. Prevalence of depression was 37% for paramedics, 28% for emergency medical personnel, and 22% for police. For symptoms severity: 67% for mild depression, 24% for moderate depression, and 16% for severe depression. Meta-regression: marital status was significantly associated with depression, while gender and being a first responder were not. |
| Ozamiz-Extebarria 2021 (104) | 3 / 2577 | 19 (15 to 24) | 83.7 | Teachers. No subgroups analyses due to insufficient studies available. |
| Ma 2022 (105) | 15 / NR | 59.9 (43.4 to 74.4) | 98.96 | Teachers. NR |

^1^ Standardized Mean Difference (SMD); ^2^ Hedges’g; ^3^ Standardized Mean Change (SMC); ^4^Pooled mean (SCL-90-R), ^5^Cohen’s d; ^6^Percentage change in cases globally during vs. pre-pandemic; C = comparison; K = number of studies included;; n = total number of participants.

## **Table B.** Pooled prevalence rates anxiety, heterogeneity and subgroup or meta-regression findings.

| **Study** | **K / n** | **Pooled prevalence / change (95% CI)** | **Pooled prevalence / change (95% CI)** | **I^2^ (%)** | **Subgroup- or meta-regression results** |  |
| --- | --- | --- | --- | --- | --- | --- |
| General Population |  |  |  |  |  |  |
| Alzahrani 2022 (1) | 11/NR | 20 (16 to 24) | 20 (16 to 24) | 99.3 | Difference in pooled prevalence for DASS-21 and GAD-7 was 19% and 20% respectively. Another study used different assessment tool. |  |
| Arora 2022 (2) | 13/NR | 28 (21 to 36) | 28 (21-36) | 99.5 | Pooled estimates for subgroups based on region, type of workers, population, timing of COVID and month of publication showed no evidence of an effect of any of these factors upon prevalence. |  |
| Bello 2022 (5) | 45/31300 | 47.0 (40.0 to 54.0) | 47.0 (40.0 to 54.0) | 99.1 | The highest prevalence was reported for studies conducted in Egypt (56%, 95% CI: 38–73%, n = 6), whereas the lowest prevalence was reported for studies in Libya. |  |
| Bareeqa 2021 (3) | 17/57311 | 21.8 (16.9 to 27.1) | 21.8 (16.9 to 27.1) | 99.5 | Higher in females. Prevalence differed according to scale used. |  |
| Castaldelli-Maia 2021 (6) | NR/191519 | 24.0 (21.0 to 27.1) | 24.0 (21.0 to 27.1) | 99.7 | Higher prevalence when closure of public transportation and in other regions than Asia. |  |
| Cenat 2021 (7) | 31/NR | 15 (11 to 20) | 15 (11 to 20) | 99.7 | NR |  |
| Cenat 2022 (8) | 22 / NR | 25.0 (20.7 to 29.3) | 25.0 (20.7-29.3) | 99.6 | Higher pooled prevalence anxiety across all time-points in North America in comparison to Europe, Latin America (and Asia-Pacific). A significant difference between regions were found. Also significant group differences across types of measures (STAI higher prevalence compared to others). Meta-regression: pooled prevalence rate is associated with female prevalence rate. |  |
|  | 15 / NR | 2.52 (1.7 to 3.4)^5^ | 2.52 (1.7 to 3.4)^5^ | 98.0 | Higher effect sizes in studies conducted in North America (d = 4.85), and in studies that used the EST-Q2 (d = 6.09). Meta-regression showed that age at baseline and measures are associated with the pooled effect size of anxiety. |  |
| Chekole & Abate 2021 (39) | 25/78191 | 29.9 (25.3 to 34.6) | 29.9 (25.3 to 34.6) | 99.6 | Prevalence highest among students followed by patients. |  |
| Chen 2021 (9) | 27/NR | 39 (32 to 46) | 39 (32 – 46) | 99.4 | Pooled estimates for subgroups based on population, frontline HCWs vs. general HCWs vs. medical students vs. GP; on symptom severity, mild vs. moderate vs. severe; on region, sub-Saharan Africa v. North Africa; on study quality, medium quality vs. high quality; and on data collection time, first six months vs. after the first six months. |  |
| Dal Santo 2022 (10) | 4 / 4,344 | 0.15 (0.07 to 0.22)^1^ | 0.15 (0.07–0.22)^1^ | 3 | Worsened more for females then for males over time. |  |
| Y. Deng 2021 (11) | NR/16386 | 22.0 (15.0 to 30.0) | 22.0 (15.0 to 30.0) | 99.3 | Prevalence increased over time in varying degrees. Higher prevalence of anxiety after peak. |  |
| De Sousa, 2021(12) | 18/NR | 28.33 (22.1-35.5) | 28.33 (22.1-35.5) | 100 | Between-population subgroup analyses were performed (GP vs. HCW) for depression and anxiety but not PTSD. No differences were found. |  |
| Delanerolle 2022 (13) | NR/NR | 21.8 (17.9 to 25.1) | 21.8 (17.88- 25.09) | 99.8 | NR |  |
| Dragioti (14) | 200/NR | 21 (19 to 23) | 21 (19-23) | 99 | Females had higher prevalence rates in almost all examined mental health problems; younger strata of the population (college students) had higher prevalence rates of anxiety. Adults had higher fear. Prevalence estimates of almost all examined mental health problems were higher in people affected by COVID‐19 infection or who had close contact with COVID‐19‐infected people. Finally, anxiety, more prevalent in low‐/middle‐income compared to high income countries. |  |
| Fan 2021 (15) | 109/127 703 | 28.0 (25.0 to 32.0) | 28.0 (25.0 to 32.0) | 99,8 | NR |  |
| Hosen 2021 (16) | 6/NR | 40 (36 to 45) | 40 (36 to 45) | 88.10 | Age and gender were found to be significant predictors of mental health problems in a large number of studies; education in some studies. |  |
| Hossain 2021 (17) | NR/NR | 40.7 (31.6 to 50.1) | 40.7 (31.6 to 50.1) | 99.1 | Higher in females; related to instruments used; and in studies with high risk of bias. |  |
| Knox 2022 (18) | 19/NR | 0.26 (-0.04 to 0.56) | 0.26 (-0.04 to 0.56) | 99.55 | Moderator/subgroup analyses: type and length of social restriction, WHO region, age, pre-existing physical or mental health vulnerabilities. Anxiety was significantly higher for those exposed to low compared to moderate social restrictions. |  |
| Kunzler 2021 (19) | 25/60213 | 0.40 (0.15 to 0.65)^1^ | 0.40 (0.15 to 0.65)^1^ | 99.0 | Higher when similar sample sizes were compared (during- to pre pandemic). |  |
| W. Li et al., 2021 (106) | 53/348754 | 29.6 (19.7 to 39.5) | 29.6 (19.7 to 39.5) | 99.9 | NR |  |
| X. Liu 2021 (21) | 23/NR | 29.5 (25.2 to 34.3) | 29.5 (25.2 to 34.3) | 98.0 | Higher in females. |  |
| Necho 2021 (22) | 8/NR | 38.1 (18.3 to 58.0) | 38.1 (18.3 to 58.0) | 96.0 | Prevalence higher in non-Chinese studies; prevalence differed according to assessment tool |  |
| Nochaiwong 2021 (23) | 75/284813 | 26.9 (24.0 to 30.0) | 26.9 (24.0 to 30.0) | 99.7 | Prevalence associated with WHO region, female gender inequality; hospital beds 10,000 people. |  |
| Kan 2021 (107) | 89/NR | 27.3 (23.7 to 31.2) | 27.3 (23.7 to 31.2) | NR | Subgroup analyses anxiety: gender (higher for female compared to male), age (higher in older adults), continents/WHO regions (higher in Africa followed by USA, Europe, and Asia lowest), occupation (higher in non-medical workers) and in pregnant women compared to general population and students. |  |
|  |  |  |  |  |  |  |
| Pappa 2022 (24) | 9/NR | 31 (20 to 44) | 31 (20–44) | 99.7 | NR |  |
| Phiri 2021 (25) | 69/NR | 22.4 (19.8 to 25.0) | 22.4 (19.8 to 25.0) | 99.9 | Heterogeneity by country of study; good (vs fair) quality slightly lower prevalence. |  |
| Prati and Mancini 2021 (26) | 9/NR | 0.17 (0.07 to 0.27)^2^ | 0.17 (0.07 to 0.27)^2^ | NR | No significant moderation effects for age, gender, continent, COVID-19 death rate, days of lockdown, publication status or study design. |  |
| Robinson 2022 (27) | 34/NR | 0.13 (-0.19 to 0.23)^3^ | 0.13 (-0.19 to 0.23)^3^ | 96.2 | Largest changes in studies with participants in early stages of pandemic. Country level factors and age, gender and region did not explain could not explain heterogeneity in primary studies of anxiety. |  |
| Santomauro 2021 (28) | 34/NR | 27.6 (25.1 to 30.3)^6^ | 27.6 (25.1 to 30.3)^6^ | NR | Daily SARS-CoV-2 infection rates and reductions in human mobility were associated with increased prevalence of major depressive disorder symptoms. Females were affected more by the pandemic than males for major depressive disorder and younger age groups were more affected than older age groups. |  |
| Sharma 2022 (108) | 8 / 7165 | 15.0 (9.8 to 20.1) | 15.0 (9.8–20.1) | 98.6 | Anxiety symptoms based on DASS-21 reported here. Pooled prevalence of psychological distress was significantly lower based on DASS‑21 measurements as compared to those with studies other scales. |  |
| Zhang, Chen 2022 (32) | NR/NR | 20 (16 to 25) | 20 (16–25) | 84- 99 | Studies with high quality reported lower prevalence of mental health symptoms. |  |
| Zhang, Batra 2022 (33) | NR/NR | 34 (29 to 40) | 34 (29–40) | NR | Prevalence rates higher in South America than in Central America and higher among frontline HCWs and students than among the general population and general HCWs. |  |
| Zhang, Miller (2022) (34) | NR/NR | 22 (15 to 31) | 22 (15–31) | NR | High quality reported lower prevalence of clinically significant symptoms of mental health symptoms. |  |
| Yan 2022 (31) | 9/9908 | 23 (12 to 38) | 23 (12-38) | 99.5 | Older adults: subgroup analyses showed significant difference among samples collected in different phases of the pandemic. Anxiety among older adults from the general public was highest during the Phase 2 (March-April 2020) of the pandemic, followed by Phase 1 (January-February 2020) and Phase 3 (May-August 2020). Region was not a significant source of heterogeneity. |  |
| Zhao 2021 (35) | 10/20599 | 21.2 (16.6 to 26.7) | 21.2 (16.6 to 26.7) | 98.7 | Higher in females and in studies reporting higher quality scores. |  |
| **Health care workers** |  |  |  |  |  |  |
| Abdulla 2021 (36) | 10/3059 | 42.9 (30.3 to 55.5) | 42.9 (30.3- 55.5) | 98.00 | NR |  |
|  |  |  |  |  |  |  |
| Adibi 2021 (109) | 19/21866 | 30.5 (25.6 to 34.4) | 30.5 (25.6-34.4) | 98.4 | High heterogeneity may reflect large differences in sample sizes |  |
| Aymerich 2021 (38) | 179/206513 | 42 (35 to 48) | 42 (35–48) | 99.9 | NR |  |
| Bareeqa 2021 (3) | 10267/8 | 23.7 (16.8 to 31.3) | 23.7 (16.8-31.3) | 98.5 | NR |  |
| Castaldelli-Maia 2021 (6) | 23/NR | 15.9 (12.2 to 20.3) | 15.9 (12.2 - 20.3) | 98.9 | Associated with anxiety: closure of public transportation. |  |
| Cenat, Blais-Rochette 2021 (7) | 23/NR | 15 (12 to 19) | 15 (12 to 19) | 99.5 | NR |  |
| Chekole & Abate 2021 (39) | NR | 30.2 (20.5 to 39.9) | 30.2 (20.5-39.9) | 99.5 | Anxiety was highest among students followed by patients. |  |
| Ching 2021 (40) | 117/99639 | 39.7 (34.3 to 45.1) | 39.7 (34.3-45.1) | 98 | Women and nurses were at increased risk of having depression or anxiety |  |
| Da Silva 2021 (41) | 7/7,102 | 36 (19 to 58) | 36 (19-58) | 98.8 | NR |  |
| Y. Deng 2021 (11) | 22/11438 | 40.0 (33.0 to 46.0) | 40.0 (33.0-46.0) | 98.6 | High inherent between study heterogeneity probably due to the inconsistent scales and cutoff |  |
| De Sousa 2021 (12) | 18/NR | 27.5 (23.8 to 31.6) | 27.5 (23.78-31.55) | 99.9 | Between-population subgroup analyses were performed (GP vs. HCW) for depression and anxiety but not PTSD. No differences were found |  |
| Delanerolle 2022 (13) | NR/NR | 22.35 (23.2 to 33.1) | 22.35 (23.22- 33.08) | 99.8 | NR |  |
| Dong 2021 (42) | 22/NR | 34.4 (29.5 to 39.4) | 34.4 (29.5–39.4) | 98.8 | High heterogeneity: small sample capacity, non-random sampling, different scales. |  |
| Dragioti 2022 (14) | 103/NR | 24 (21 to 35) | 24 (21-35) | 98 | Females higher prevalence rates in almost all examined mental health problems; younger strata of the population (students) had higher prevalence rates of anxiety symptoms. Anxiety symptoms more prevalent in low‐/middle‐income countries. |  |
| Dutta 2021 (43) | 31/23472 | 32.5 (26.4 to 39.0) | 32.5 (26.4–39.0) | 99.0 | High heterogeneity: differences in the sample, assessment methods, cutoff value, disease severity. |  |
| El-Qushayri 2021 (44) | 4/NR | 71.8 (49.4 to 86.9) | 71.8 (49.4-86.9) | NR | High heterogeneity; lower in some groups of disease severity. |  |
| Ghahramani 2021 (45) | 5/NR | DASS 47 (22 to 74) | DASS 47.0 (22-74) | 99.1 | Anxiety levels differed depending on the study tool. |  |
|  | 3/NR | SAS 24 (12 to 41) | SAS 24 (12 to 41) | 96.4 | Subgroup analyses: anxiety more prevalent in physicians, nurses and older people, and in studies conducted in China. |  |
|  | 15/NR | GAD-7 36 (29 to 43) | GAD-7 36 (29- 43) | 98.9 | Meta-regression: a significant relationship was revealed between age and sample size (p<0.001) and mental health outcomes. |  |
| Halemani 2021 (46) | 12/3315 | 34 (26 to 42) | 34 (26 to 42) | 95.26 | Doctors. |  |
|  | 13/4167 | 42 (33 to 50) | 42 (33 to 50) | 97.42 | Nurses. Nurses being in close contact with Covid patients had a greater chance to experience anxiety. |  |
| Hosen 2021 (16) | 4/NR | 52 (37 to 71) | 52 (37 to 71) | 96.51 |  |  |
| Hao 2021 (47) | 16/NR | 28.6 (22.4 to 36.4) | 28.6 (22.4–36.4) | 99.0 | Females and frontline workers higher anxiety; second line workers lower anxiety. |  |
| Hossain 2021 (17) | NR | 43.6 (33.1 to 54.5) | 43.6 (33.1–54.5) | 99.1 | Anxiety prevalence: higher when percentage of females higher; prevalence rates varied across commonly used scales and higher prevalence when studies had high compared to low risk of bias. |  |
| Hu 2022 (48) | 63/76998 | 27 (20 to 35) | 27 (20 to 35) | 100 | Subgroup analyses: the working type (first-line vs non-first-line medical staff) is an important factor in the mental problems of medical staff. Meta-regression analyses: as predictors, mean age and sex had no significant effect on anxiety. |  |
| Johns 2022 (49) | 30/33281 | 26 (20 to 32) | 26 (20 to 32) | 99.19 | Subgroup analyses: GDP per capita, region, doctors per 100K (significant). Between-group heterogeneity was statistically significant when analysed by measure, severity threshold, and survey timeframe, but not by risk of bias. |  |
| **Kunzler 2021** (19) | **13/5508** | **-0.08 (−0.7** to **0.5)^1^** | **-0.08 (−0.66-0.49)^1^** | **99.0** | **Specific assessment tools, older comparative data (published ≥5 years ago) associated with increased mental burden. Level of COVID-19 patient contact did not affect the mental health impact. Evidence for elevated symptoms of anxiety if similar sample sizes were compared (of during- to pre pandemic studies).** |  |
| Li 2021 (50) | 57/NR | 22.1 (18.2 to 26.3) | 22.1 (18.2-26.3) | 99.4 | NR |  |
| Liu 2021 (51) | 20/NR | 44.0 (34 to 53) | 44.0 (34- 53) | 95.0 | NR |  |
| Liu 2021 (21) | 32/NR | 32.7 (27.9 to 38.2) | 32.7 (27.9 - 38.2) | NR | NR |  |
| Mahmud 2021 (52) | 75/147435 | 41.4 (36.2 to 46.5) | 41.4 (36.2–46.5) | 99.8 | High heterogeneity for anxiety associated with later data collection and lower sample size. |  |
| Marvaldi 2021 (53) | 22/51942 | 30.0 (24.2 to 37.0) | 30.0 (24.2-37.0) | 99.5 | High heterogeneity  for anxiety not associated with high quality criteria study, gender, location, type of scale or  location. |  |
| Norhayati 2021 (54) | 68/124925 | 34.8 (30.8 to 38.8) | 34.8 (30.8-38.8) | 100 | Highest prevalence of anxiety Western Asia and in non-frontline healthcare providers, females and nurses. |  |
| Pappa 2022 (24) | 5/NR | 23 (13 to 34) | 23 (13–34) | 98.1 | Frontline HCW: NR |  |
|  | 15/NR | 18 (12 to 80) | 18 (12–80) | 98.1 | General HCW: NR |  |
| Phiri 2021 (25) | 69/NR | 21.9 (18.7 to 25) | 21.9 (18.7-25) | 99.7 | Lower prevalence of anxiety among healthcare professionals for good quality studies. |  |
| Raoofi 2021 (110) | 46/61551 | 26.1 (19.0 to 34.6) | 26.1 (19.0 34.6) | NR | Higher prevalence among women, medical students and medical technicians.  Positive significant relationship between prevalence of anxiety among HCWs and age. |  |
| Rezaei-Hachesu, 2022 (111) | 7/2321 | 42 (25 to 59) | 42 (25-59) | 98.93 | The pooled prevalence rate of anxiety by severity subgroups was calculated as 24% in mild anxiety, 19% in moderate anxiety, 13% in severe anxiety, and 11% in very severe anxiety. The pooled prevalence rate of anxiety was 54% among nurses and 29% among all medical staff. |  |
| Santabárbara 2021 (112) | 59/NR | 25 (21 to 29) | 25 (21-29) | 99.1 | Heterogeneity due to diagnostic tool, country (China vs non-China), sampling method (convenience vs other), quality rating. |  |
| Saragih 2021 (56) | 34/NR | 40 (29 to 52) | 40 (29-52) | 99.9 | NR |  |
| Singh 2021 (30) | 6/NR | 34.1 (26.3 to 42.3) | 34.1 (26.3– 42.3) | 97.1 | NR |  |
| Ślusarska (57) | 22/23 | 29 (18 to 40) | 29 (18–40) | 99.9 | Subgroup analysis by tool: GAD-7 22% other 57%. Subgroup analysis by area: Asia 25%vs other 40%. Subgroup analysis by position: frontline 31% vs mixed 23%. Meta-regression: female (p = 0.04) related to prevalence of anxiety but not to marital status (p = 0.38). |  |
| Sun 2021 (58) | 44/NR | 37 (31 to 42) | 37 (31–42) | 99.9 | NR |  |
| Varghese 2021 (52)  Xiong et al 2022 (60) | 21/NR  18/34,793 | 33 (24 to 43)  17 (13 to 21) | 33 (24-43)  17 (13-21) | 99.4  NR | NR  The prevalence of moderate to severe anxiety was higher in frontline than non-frontline participants and higher in HCW from Wuhan than from other cities in the Hubei province and other provinces in China. The difference between positions (frontline versus non-frontline) and locations could partly account for the large heterogeneity within the whole sample. |  |
| Yan 2021 (61) | 29/15704 | 41 (35 to 47) | 41 (35-47) | 98.0 | Lower prevalence locations other than Wuhan and higher in medical than non-medical staff and  frontline versus non-frontline staff. |  |
| Zhang 2021 (62) | 23/21447 | 27 (21.2 to 33.7) | 27 (21.2- 33.7) | 99.0 | NR |  |
| Zhao 2021 (35) | 14/13020 | 23.2 (17.1 to 30.8) | 23.2 (17.1- 30.8) | 98,7 | Higher prevalence anxiety in higher quality reviews. |  |
| **Patients (COVID-19/other)** |  |  |  |  |  |  |
| Ayubi 2021 (63) | 17/NR | 38.0 (31.0 to 46.0) | 38.0 (31.0 – 46.0) | 99.1 | Cancer patients. NR |  |
| Chekole 2021 (39) | 3/NR | 34.7 (27.0 to 42.5) | 34.7 (26.95-42.5) | NR | Covid patients. NR |  |
| Dong 2021 (64) | 27/5,144 | 39.6 (30.8 to 48.5) | 39.6 (30.8-48.5) | 98.2 | Heterogeneity due to populations, sampling methods and scales across studies. Higher prevalence in more clinical severe COVID-19 patients and time of data collection during pandemic. |  |
|  |  |  |  |  |  |  |
| Dragioti, 2022 (14) |  |  |  |  |  |  |
| Covid Patients | 24/NR | 29 (18 to 42) | 29 (18-42) | 98 | Prevalence estimates of almost all examined mental health problems including anxiety were higher in people affected by COVID‐19. Finally, anxiety more prevalent in low‐/middle‐income countries. |  |
| Somatic Disorders | 7/NR | 31 (21 to 41) | 31 (21−41) | NR |  |  |
|  |  |  |  |  |  |  |
| Khraisat 2022 (65) | 8/2444 | 64 (48 to 79) | 64 (48 to 79) | 98 | Patients with eating disorders |  |
| Kunzler 2021 (19) | 6 / 1845 vs 12458 | 0.31 (-0.07 to 0.69) ^1^ | 0.31 (-0.07 to 0.69) ^1^ | 93.0 | Specific assessment tools and older comparative data associated with increased mental burden. |  |
| Kuroda 2021 (66) | 19/6461 | 39 (31 to 47) | 39 (31 to 47) | 97 | Patients with epilepsy. Subgroup analyses: questionnaire, continents, income country, early/late pandemic phases. |  |
| Lee K 2022 (67) | 5/938 | 23 (12 to 34) | 23 (12 to 34) | 94.89 | People living with HIV (PLHIV) |  |
| Liu 2021 (68) | 19 / NR | 38 (24 to 52) | 38 (24–52) | 98.0 | Higher prevalence in females, in acute SARS-CoV-2 patients, in those with mild symptom severity, small difference in prevalence between high vs low quality. Heterogeneity largely unexplained: variance in prevalence due to screening tool. |  |
| Liu 2021 (21) | 5 / NR | 63.9 (29.6 to 88.2) | 63.9 (29.6 - 88.2) | 98.0 | Higher prevalence in confirmed patients than other groups but lower in China than in other countries and significant variance across different scales. |  |
| Kan 2021 (107) | 14/NR | 40 (30 to 50) | 40 (30 to 50) | NR | Covid-19 patients. Subgroup analyses: gender (higher for female), age (inverse relationship), continents/WHO regions (higher in Europe) |  |
|  | 5/NR | 39 (25 to 50) | 39 (25 to 50) | 95.87 | Other patients (not covid-19) [Subgroup analysis from general population] |  |
| Natarajan 2022 (69) | 7/NR | 27.8 (16.6 to 46.5) | 27.8 (16.56 - 46.53) | 96.2 | Long COVID patients. |  |
| Premraj, 2022 (70) | 8/598 | 22.97 (14.2 to 31.8) | 22.97 (14.19-31.75) | 98.01 | Anxiety was more frequently reported in the community than in patients hospitalised during their acute phase of COVID-19 (31% vs. 16%); anxiety was substantially increased in prevalence when assessed at or beyond six months (long-term) than when assessed between three and six months (mid-term). Patients hospitalised during acute COVID-19 less likely developed anxiety >3 months after COVID-19 onset than those who remained non-hospitalised throughout their acute infection. Anxiety approximately three times more prevalent in cohorts with >20% of participants admitted to an ICU as in cohorts in which <20% underwent ICU admission. |  |
| Sideli 2021 (71) | 4 / NR | 50 (42 to 57) | 50 (42-57) | 62.6 | Eating disorder patients. NR |  |
|  | 3 / NR | 51 (21 to 81) | 51 (21-81) | 99.3 | Morbid obesity patients: NR |  |
| Xie 2021 (72) | 11 / 1675 | 2.23^4^ (1.9 to 2.6) | 2.23^4^ (1.9-2.6) | 99.7 | Acute COVID-19 stage: NR |  |
|  | 2 / 30 | 1.67^4^ (1.0 to 2.4) | 1.67^4^ (1.0-2.4) | 94.8 | Early COVID-19 recovery stage: NR |  |
| Yan et al 2022 | 4/97 | 14 (9 to 23) | 14 (9-23) | 0 | Older adults COVID-19: No significant heterogeneity; subgroup analysis showed that neither assessment criteria nor time phases of the pandemic can account for the heterogeneity. |  |
| **Perinatal women** |  |  |  |  |  |  |
| Tomfohr-Madsen 2021 (76) | 34/ 42,773 | 30.5 (22.6 to 39.8) | 30.5 (22.6-39.8) | 98.4 | NR |  |
| Cevik 2022 (75) | 10 / NR (before)  6 / NR (during) | 25.9 (16.2 to 38.7)  17.2 (11.2 to 25.5) | 25.9 (16.2–38.7)  17.2 (11.2–25.5) | NR | Moderator variable anxiety ‘before and during the pandemic’ was not statistically significant. |  |
| Demissie 2021 (78) | 16 / 1627 | 33.0 (50.0 to 61.0) | 33.0 (50.0-61.0) | NR | Subgroup analyses gave null results |  |
| Dragioti (14) | 7/NR | 34 (28 to 40) | 34 (28-40) | 99 | Females had higher prevalence rates in almost all examined mental health problems. Anxiety more prevalent in low‐/middle‐income compared to high-income countries. |  |
| Ghazanfarpour 2021 (79) | 11 / NR | 18.7 (6.0 to 36.0) | 18.7 (6.0–36.0) | 99 | Continent subgroup analysis: prevalence of anxiety was higher in Western countries than in Asia countries |  |
| Kan 2021 (107) | 11/NR | 34 (21 to 50) | 34 (21 to 50) | 99.32 | [Subgroup analysis from general population] |  |
| Shorey 2021 (80) |  |  |  |  |  |  |
| Prenatal subsample | 12 / 8547 | 40 (27.0 to 52) | 40 (27.0 to 52) | 100 | Prevalence highest in Europe and North America, lowest in Asia. |  |
| Perinatal subsample | 4 / 1819 | 50 (24.0 to 75) | 50 (24.0 to 75) | 100 |  |  |
| **Students** |  |  |  |  |  |  |
| Batra 2021 (81) | 20/84.097 | 34.4 (29.5 to 39.7) | 34.4 (29.5- 39.7) | 99.8 | Higher prevalence of anxiety in lower quality studies, in areas other than Asia/China, in females, in assessment tools other than GAD. |  |
| Chang 2021 (82) | 16/ 144,010 | 31 (23 to 39) | 31 (23-39) | 99.9 | Prevalence of anxiety symptoms differed among countries. |  |
| Chekole & Abate 2021 (39) | 2 / NR | 43.6 (-11.6 to 98.8) | 43.6 (-11.6-98.8) | 99.8 | Prevalence of anxiety was highest among students followed by patients. |  |
| Deng 2021 (83) | 69/1,094,240 | 32 (26 to 38) | 32 (26-38) | 100 | Significant positive correlations between study date and pooled anxiety symptoms. |  |
| Ebrahim, 2022 (84) | 9/22,357 | 29.1 (20.9 to 39.0) | 29.1 (20.9-39.0) | 99.3 | NR |  |
| Fang 2022 (85) | 93/2048035 | 28 (24 to 32) | 28 (24 to 32) | 100 | Pooled prevalence of anxiety in the student population was influenced by gender, the period of the epidemic, the region, the stage of education, the student’s major, and the instrument used in the evaluation. The prevalence anxiety higher among females than males in the student population. For students at different educational levels, the prevalence of anxiety symptoms were higher in undergraduate and postgraduate students than in primary school and middle school students. In addition, non-medical students had higher prevalence of anxiety than medical students. |  |
| Hosen 2021 (16) | 7/NR | 52 (37 to 58) | 52 (37 to 58) | 99.72 | Age and gender were found to be significant predictors of mental health problems in a large number of studies; education in some studies. |  |
| Jia 2022 (87) | 37/34285 | 34 (27 to 41) | 34 (27 to 41) | 99 | Subgroup analyses: differences in assessment tool, country, gender. |  |
| Li 2021 (88) | 20 / 73912 | 36 (26 to 46) | 36 (26- 46) | 99.9 | Higher prevalence in non-Chinese students (versus Chinese students) and after versus before March 1, 2020. |  |
| Liyanage 2022 (113) | 36 / NR | 41 (34 to 49) | 41 (34–49) | 100 | Anxiety prevalence lowest in Asia (33%), in Europe 51 and highest in USA (56%). A subgroup analysis: prevalence of anxiety in females 43% compared to males 39%. |  |
| Kan 2021 (107) | 23/NR | 31 (22 to 41) | 31 (22 to 41) | 99.43 | [Subgroup analysis from general population] |  |
| Mulyadi 2021 (94) | 10/9554 | 32 (24 to 42) | 32 (24–42) | 98 | NR |  |
| Oliveira Carvalho 2022 (90) | 10/NR | 55 (45 to 64) | 55 (45-64) | 99 | The pooled prevalence for studies using GAD-7 questionnaires was higher than compared to the other assessment tools with 57% and 51%, respectively. |  |
| Pappa 2022 (24) | 3/NR | 18 (8 to 32) | 18 (8–32) | 99.4 | NR |  |
| Santabarbara (114) | 15/15 | 35 (26 to 45) | 35 (26-45) | 98.4 | Meta-regression: women, mean age at baseline, response rate, methodological quality not significantly explaining prevalence differences between studies. Sampling method no sign. moderator; only region sign. with lower prevalence in Europe (21%) compared to America (39%) and Asia: 37% and instruments (GAD-7 31%) lower prevalence then DASS-21 36% and SAS 57%. |  |
| Wang 2021 (95) | 19 / > 83,699 | 29 (19 to 25) | 29 (19-25) | 99.8 | Higher prevalence in non-Chinese versus Chinese students. |  |
| Zhu et al (96) | 144/ NR (total 1,732,456) | 28.2 (24.6 to 32.1) | 28.2 (24.6-32.1) | 99.9 | Significant moderators geographical region and month of data collection. Clinical anxiety lower in studies East Asia compared to all other regions and for every month increase a  0.18% increase in anxiety prevalence. Age, sex, type of student, level of training, or study quality were no significant moderators for the prevalence of clinically significant anxiety symptoms. |  |
| **Children and adolescents** |  |  |  |  |  |  |
| Chai 2021 (98) | NR/NR | 25 (20 to 32) | 25 (20 – 32) | 99 | Gender (percentages of boys higher – lower prevalence) had an effect on symptoms. | |
| Ma 2021 (99) | 13 / NR | 26 (16 to 35) | 26 (16-35) | 99.9 | Higher in adolescents and females compared to children and males, respectively. |  |
| Racine 2021 (101) | 25 / 70707 | 21 (17 to 24) | 21 (17-24) | 99.3 | Prevalence rates of clinically elevated anxiety was higher in females, in European countries compared with East Asian countries and in poorer quality studies. |  |
| Panda 2021 (100) | 11/ 21 330 | 34.5 (33.8 to 35.1) | 34.5 (33.8 - 35.1) | 45 | NR |  |
| **University and schoolteachers** |  |  |  |  |  |  |
| Ozamiz-Etxebarria 2021 (104) | 6/ 91498 | 17 (9 to 28) | 17 (9-28) | 99 | Non-significant higher prevalence according to study locations (other continents (22%); Asian (14%)) and in school- versus university teachers |  |
| Ma 2022 (105) | 12 / Nr | 36.3 (28.5 to 44.9) | 36.3 (28.5–44.9) | 99.74 | NR |  |
| **Caregivers** |  |  |  |  |  |  |
| Dragioti, 2022 (14) | 7/NR | 42 (24 to 60) | 42 (24- 60) | 99 | Prevalence estimates of almost all examined mental health problems were higher in people who had close contact with COVID‐19‐infected people (such as in caregivers). |  |
| Panda 2021 (100) | 3/ 257 | 52.3 (42.4 to 60.7) | 52.3 (42.4–60.7) | 61 | NR |  |
| Racine 2021 (102) | 8/5286 | 42 (26.7 to 58.8) | 42 (26.7- 58.8) | 99.1 | Mothers of young children: prevalence of clinical anxiety higher in studies of poorer quality compared better quality studies and higher in studies from Europe and North America compared to studies from South America, the Middle East, or Asia. Maternal age: anxiety symptoms higher in studies with older mothers. More highly educated mothers exhibiting higher clinically significant symptoms of anxiety. |  |
| **Working population** |  |  |  |  |  |  |
| Dragioti 2022 (14) | 14/NR | 8.0 (3.0 to 14.0) | 8.0 (3.0- 14.0) | 99 | Females had higher prevalence rates in almost all examined mental health problems and younger strata of the population (college students) had higher prevalence rates of anxiety and adults had higher fear. Finally, anxiety more prevalent in low‐/middle‐income countries. |  |
| Huang 2022 (103) | 16/7795 | 0.32 (0.20 to 0.44) | 0.32 (0.20 to 0.44) | 98 | Subgroup analyses: type of interview, study quality, and sample size were significant moderator variables while study type, country status, setting, and assessment tool were not. Prevalence of anxiety was 38% for paramedics, 28% for EMS personnel, and 19% for police. For symptoms severity: 60% for mild anxiety, 27% for moderate anxiety, and 14% for severe anxiety. Meta-regression: marital status was significantly associated with anxiety, while gender and being a first responder were not. |  |

^1^Standardized Mean Difference (SMD);^2^ Hedges’g; ^3^ Standardized Mean Change (SMC); ^4^Pooled mean (SCL-90-R); ^5^Pooled effect size (on mean differences);

C = comparison; K = number of studies included; n = total number of participants.

## **Table C.** Pooled prevalence rates PTSD, heterogeneity and subgroup or meta-regression findings.

| **Study** | **K / n**  **PTSD** | **Pooled prevalence % (95% CI)** | **I^2^ (%)** | **Subgroup- or meta-regression results** |
| --- | --- | --- | --- | --- |
| **General Population** |  |  |  |  |
| Aymerich, 2021 (38) | 39/58995 | 32 (26 to 37) | 99.9 | The meta-regression results reveal **fewer symptoms in the posttraumatic domain in Asia** than in other continents. |
| Arora, 2022 (2) | 4/NR | 33 (0 to 86) | 99.8 | Pooled estimates for subgroups based on region, type of workers, population, timing of COVID and month of publication showed no evidence of an effect of any of these factors upon prevalence. |
| Cenat 2022 (8) | 8 / NR | 17.5 (14.1 to 20.9) | 95.5 | Pooled prevalence across all time-points. North America higher prevalence of PTSD in comparison with studies in Europe (statistically significant). Significant difference between the type of measures (COVID-19 TSRD highest prevalence compared to e.g. PCL-C and IES-R). Meta-regression: region negatively associated with the PTSD prevalence. |
| De Sousa, 2021 (12) | 18/NR | 19.6 (15.5 to 24.4) | 99.5 | Between-population subgroup analyses were not performed for PTSD. |
| Delanerolle et al., 2022 (13) | NR/NR | 24.8 (15 to 41.2) | 99.8 | NR |
| Dragioti 2022 (14) | 13/NR | 18 (11 to 25) | 99 | Females had higher prevalence rates in almost all examined mental health problems. Adults had higher PTSD symptomatology and fear. Prevalence estimates of almost all examined mental health problems were higher in people affected by COVID‐19 infection or who had close contact with COVID‐19‐ infected people. Finally, PTSD symptoms were more prevalent in low‐/middle‐income compared to high income countries. |
| Fan 2021 (15) | 14/51 721 | 15.7 (11 to 24) | 99.7 | NR |
| X. Liu 2021 (21) | 6/NR | 9.3 (4.0 to 19.8) | 99.8 | NR |
| Nochaiwong 2021 (23) | 28/56447 | 24.1 (17.0 to 32.0) | NR | Prevalence of PTSD symptoms associated with risk of bias. |
| Phiri 2021 (25) | 19/NR | 23.2 (10.5 to 35.9) | 100 | Prevalence of PTSD higher among ≤ 30 years compared to >30 years but lower in China. |
| Qiu 2021 (115) | 76/33,810 | 28.3 (23.0 to 34.3) | 99.7 | No significant sources of heterogeneity. Significantly higher prevalence according to gender (males), and age (older) and sample size (smaller); other factors such as assessment tool (IES) and quality score differed non-significantly. |
| Qiu 2021 (116) | 51/78,459 | 24.4 (18.5 to 30.5) | 99.8 | Meta-regression analysis did not identify major contributors of heterogeneity. Subgroup analyses showed differences according to region of study (higher in eastern Mediterranean region), assessment tool, survey time after outbreak (closer to outbreak – higher) and quality (highest quality scores higher prevalence). |
| Salehi 2021 (117) | 12/11217 | 9.0 (5.0 to 16.0) | 97.8 | Location of study was a significant source of heterogeneity. |
| Yunitri 2022 (118) | 36 /91890 | 17.3 (12.2 to 24.0) | 99.7 | Subgroup analyses: studies that used Clinician-Administered PTSD Scale for DSM-5 and nurses, HCWs in Covid-19 units had highest prevalence. Age >65 years had lower PTSD prevalence compared to adults. Study’s continent (Europe higher than Asia) and assessment tools as significant moderators while age, gender, marital status, and educational level countries’ GDP, total case, and death case were not. |
| Zhang 2021 (119) | 10/73547 | 15.0 (14.0 to 25.0) | 100 | NR |
| Zhao 2021 (35) | 5/3015 | 19.2 (4.6 to 54.2) | 99.6 | Higher prevalence PTS symptoms in higher quality reviews. No significant differences in prevalence between different sex, education levels and marital status. |
| **Health Care Workers** |  |  |  |  |
| Cenat, Blais-Rochette, 2021 (7) | 4/NR | 22 (1 to 60) | 99.7 | NR |
| Delanerolle et al., 2022 (13) | NR/NR | 25.2 (16.6 to 38.1) | 99.6 | NR |
| Dong 2021 (42) | 9 / NR | 29.1 (24.3 to 33.8) | NR | High heterogeneity due to small sample capacity, non-random sampling, different scales . |
| Dragioti, 2022 (14) | 19/NR | 27 (21 to 35) | 97 | Females had higher prevalence rates in almost all examined mental health problems. Adults had higher PTSD symptomatology and fear. |
| Fan 2021 (15) | 3 / NR | 19.0 (6.4 to 44.8) | 99.7 | Incidence of PTSD in HCW is lower than that in SARS and MERS;. The level of PTSD is higher in the general public than that of HCW |
| Ghahramani 2022 (45) | 7/NR | 37 (19 to 59) | 99.7 | IES-R Subgroup analyses: anxiety more prevalent in physicians, nurses and older people, and in studies conducted in China. Meta-regression: a significant relationship was revealed between age and sample size (p<0.001) and mental health outcomes. |
| Li 2021 (50) | 9 / NR | 21.5 (10.5 to 34.9) | 99.7 | Moderating role of the measurement tool |
| Liu 2021 (21) | 5 / NR | 30.6 (9.1 to 65.9) | NR | High between-study heterogeneity, not adequately explained by the meta-regression |
| Marvaldi 2021 (53) | 17 / 29084 | 31.2 (21.2 to 43.1) | 99.7 | No factor explained high heterogeneity (quality criteria, female or nurse proportion, location, scale, sample size) |
| Norhayati 2021 (54) | 9 / 23012 | 15.3 (11.4 to 19.2) | 99.0 | High heterogeneity except for females. Highest PTSD symptom prevalence in frontline healthcare providers, females, and nurses. |
| Phiri 2021 (25) | 19 / NR | 25.0 (18.9 to 31.2) | 99.8 | High heterogeneity not explained by sub-group analyses by mean/median age, country (insufficient number of studies), measurement tool. No differences in PTSD prevalence according to China only studies. |
| Qiu 2021 (115) | 26 / NR | 29.22 (21.10 to 38.94) | 99.5 | No significant moderators of heterogeneity. Significantly higher prevalence according to gender (males), and age (older) and sample size (smaller); other factors such as assessment tool (IES) and quality score differed non-significantly. |
| Qiu 2021 (116) | 27/34842 | 29.6 (21.7 to 39.0) | 95.5 | Mortality rate of disease was a significant moderator for heterogeneity. Subgroup analyses: higher prevalence according to type of disease (MERS), mortality rate, region of study (European region), assessment tool, and when survey closer to outbreak. |
| Qi, 2022 (120) | 14/4611 | 31 (21 to 40) | 98.3 | Doctors: Asian 0.44 higher than Caucasian 0.26; differences in prevalence rates depending on instrument (IES-R vs PCL-5 and PCL-C). |
|  | 27/16280 | 38 (30 to 45) | 99.2 | Nurses: Asian 0.37 higher than Caucasian 0.34; differences in prevalence rates depending on instrument IES-R highest PCL-C lowest. |
| Saragih 2021 (56) | 7 / NR | 49 (22 to 75) | 99.9 | NR |
| Varghese 2021 (59) | 3 / 638 | 18.6 (4.8 to 38.3) | 96.4 | NR |
| Xiong et al 2022 (60) | 7/28,148 | 27 (16 to 38) | 99.8 | The prevalence of moderate to severe PTSS was higher in frontline than non-frontline HCW, higher in female than male HCW, higher in nurses than technicians or others, and higher in HCW from Wuhan than those from other provinces in China. The difference between gender occupations and locations could partly account for the large heterogeneity. |
| Yan 2021 (61) | 2 / 435 | 38 (34 to 43) | 96.0 | NR |
| Yunitri 2022 (118) | 24 / 27670 | 17.2 (11.8 to 24.5) | 99.3 | Subgroup analyses: nurses, HCWs in Covid-19 units had highest prevalence. Age >65 years had lower PTSD prevalence compared to adults. |
| Zhao 2021 (35) | 5 / 4327 | 28 (9.5 to 59.0) | 99,6 | Higher prevalence PTSD in higher quality reviews. Across all populations: no significant differences in prevalence of PTSS for sex, education levels, marital status, population. |
| **Patients (COVID-19/other)** |  |  |  |  |
| Dong 2021 (64) | 10 / 1982 | 41.5 (9.3 to 73.7) | 99.8 | COVID-19 patients: a significant degree of heterogeneity in terms of populations, sampling methods and scales across studies. Higher prevalence in more clinical severe COVID-19 patients and time of data collection during pandemic. |
| Dragioti, 2022 (14) | 2/NR | 94 (94 to 96) | NR | COVID-19 patients: females, adults, people affected by COVID‐19 infection had higher PTSD symptomatology and fear and PTSD symptoms were more prevalent in low‐/middle‐income countries. |
| Nagarajan 2022 (121) | 13 / 1093 | 16 (9 to 23) | 87.9 | Severe COVID-19 patients: difference in prevalence estimate between the regions was statistically significant. |
| Qiu 2021 (115)  Suspected COVID-19 | 3 /NR | 24.5 (17 to 33.9) | 75.8 | Meta-regression analysis did not identify significant sources of the heterogeneity in PTSD symptom prevalence (e.g. gender, age, tool) after controlling for other factors. |
| COVID-19 patients | 5 / NR | 36.3 (8.9 to 77) | 98.9 |  |
| Qiu 2021 (116) |  |  |  |  |
| Suspected infectious disease | 3/525 | 25 (18.1 to 34.7) | 74.5 | No significant contributor of heterogeneity. |
| Confirmed infectious disease | 15/ 2666 | 28.8 (18.5 to 44.9) | 98.6 | No significant contributor of heterogeneity. A significant difference in prevalence between studies with different quality scores. |
| Yunitri 2022 (118) | 11/ 2951 | 15.4 (10.6 to 22.0) | 94.3 | Patients/survivors COVID-19: meta-regression analysis found age as the only statistically significant moderator with those in the elderly group ( > 65 years old) had lower PTSD prevalence during the COVID-19 pandemic compared to adults. |
| **Young people** |  |  |  |  |
| **Students** |  |  |  |  |
| Batra 2021 (81) | 3 / 4242 | 29.8 (3.0 to 85.4) | 99.8 | NR |
| **Children and adolescents** |  |  |  |  |
| Ma 2021 (99) | 2 / NR | 48 (-25 to 121) | 100 | No subgroup analyses were conducted in PTSD due to small number of studies. |
| Yang et al 2022 (122) | 10/17,385 | 28.2 (19.5 to 36.8) | 99.7 | Subgroup analyses region of study populations: prevalence of post PTSD was 19.6% in the Chinese children, 50.8% in American and 50.1% in Italian children. A meta-regression indicated significant differences between study regions in the estimated child PTSD prevalence (p<0.001). |
| **Bussieres (2022) (123)** | **21 / 10,425** | **0.28 (0.15 to 0.41)^1^** | **98.0** | **Worse negative general mental health during public health and social measures in children in Europe (3 times worse) than in Asia and when child instead of parent was informant.** |

^1^  Hedges’ g negative general mental health.

## **Figure 1**: PRISMA flow chart initial search Dec. 31^st^, 2019 until Oct. 6^th^, 2021

Records identified from:

PubMed: 21.876

Embase: 10.242

PsycINFO: 688

CINAHL: 1.811

Web of Science: 11.361

Cochrane Reviews: 306

**Total: 46.284**

Records removed *before screening*:

Duplicate records removed

(n = 14725)

**Identification**

Records screened

(n = 31 559)

Records excluded on Title/Abstract

(n = 30 620)

**Screening**

Reports excluded

No systematic review or meta-analysis

(n = 362)

Reports screened full-text

(n = 939)

Reports excluded (n=519)

Reasons:

1) Wrong outcome (n=158)

2) Wrong study duration (n=35)

3) Wrong publication type (n=199)

4) Wrong topic (n=89)

5) Unretrievable (n=1)

6) Duplicates (n=3)

7) E-publication 2020 (n=34)

Reports assessed for eligibility*

(n = 577)

Included reports (n = 58) with meta-analyses in the following samples:

General Populations (n=21)

Healthcare Workers (n=32)

Vulnerable Groups (n=27)

**Included**

*From:*  Page MJ, McKenzie JE, Bossuyt PM, Boutron I, Hoffmann TC, Mulrow CD, et al. The PRISMA 2020 statement: an updated guideline for reporting systematic reviews. BMJ 2021;372:n71. doi: 10.1136/bmj.n71

## **Figure 2**: PRISMA flow chart updated search Oct. 7^th^, 2021 until August 12^th^, 2022.

Records identified from search 2:

PubMed: 12.204

Embase: 9160

PsycINFO: 2373

CINAHL: 1361

Web of Science: 6229

Cochrane Reviews: 147

**Total: n = 31474**

Records removed *before screening*:

Duplicate records removed

(n = 9588)

**Identification**

Records screened after removal of duplicates

(n = 21886)

Records excluded based

on title and abstract

(n = 21423)

**Screening**

Reports excluded

No systematic review or meta-analysis

(n = 136)

Articles (full-text) assessed

(n = 463)

Reports excluded (n= 262)

Reasons:

1) Wrong outcome (n= 32)

2) Wrong study duration (n= 6)

3) Wrong publication type (n= 214)

4) Wrong topic/population (n = 2)

5) Unretrievable/language (n =1)

6) Duplicates (n= 1)

7) E-publication 2020 (n= 6)

Reports assessed for eligibility*

(n = 327)

Included reports (n = 65) with meta-analyses

**Included**

*From:*  Page MJ, McKenzie JE, Bossuyt PM, Boutron I, Hoffmann TC, Mulrow CD, et al. The PRISMA 2020 statement: an updated guideline for reporting systematic reviews. BMJ 2021;372:n71. doi: 10.1136/bmj.n71

## **References**

1. Alzahrani F, Alshahrani NZ, Abu Sabah A, Zarbah A, Abu Sabah S, Mamun MA. Prevalence and factors associated with mental health problems in Saudi general population during the coronavirus disease 2019 pandemic: A systematic review and meta-analysis. Psych J. 2022; 11(1):18–29. <https://doi.org/10.1002/pchj.516>. PMID: [34986503](https://pubmed.ncbi.nlm.nih.gov/34986503/)

2. Arora T, Grey I, Östlundh L, Lam KBH, Omar OM, Arnone D. The prevalence of psychological consequences of COVID-19: A systematic review and meta-analysis of observational studies. J Health Psychol. 2022;27(4):805–824. <https://doi.org/10.1177/1359105320966639>. PMID: [33118376](https://pubmed.ncbi.nlm.nih.gov/33118376/)

3. Bareeqa SB, Ahmed SI, Samar SS, Yasin W, Zehra S, Monese GM, et al. Prevalence of depression, anxiety and stress in china during COVID-19 pandemic: A systematic review with meta-analysis. Int J Psychiatry Med . 2021; 56(4):210–27. <https://doi.org/10.1177/0091217420978005>. PMID: [33243029](https://pubmed.ncbi.nlm.nih.gov/33243029/)

4. Balakrishnan V, Ng KS, Kaur W, Govaichelvan K, Lee ZL. COVID-19 depression and its risk factors in Asia Pacific – A systematic review and meta-analysis. J Affect Disord. 2022; 298:47–56. <https://doi.org/10.1016/j.jad.2021.11.048>. PMID: [34801606](https://pubmed.ncbi.nlm.nih.gov/34801606/)

5. Bello UM, Kannan P, Chutiyami M, Salihu D, Cheong AMY, Miller T, et al. Prevalence of Anxiety and Depression Among the General Population in Africa During the COVID-19 Pandemic: A Systematic Review and Meta-Analysis. Front Public Health. 2022; 10:814981. <https://doi.org/10.3389/fpubh.2022.814981>. PMID: [35655463](https://pubmed.ncbi.nlm.nih.gov/35655463/)

6. Castaldelli-Maia JM, Marziali ME, Lu Z, Martins SS. Investigating the effect of national government physical distancing measures on depression and anxiety during the COVID-19 pandemic through meta-analysis and meta-regression. 2021; 51(6):881-893. <https://doi.org/10.1017/s0033291721000933>. PMID: [33648613](https://pubmed.ncbi.nlm.nih.gov/33648613/)

7. Cénat JM, Blais-Rochette C, Kokou-Kpolou CK, Noorishad PG, Mukunzi JN, McIntee SE, et al. Prevalence of symptoms of depression, anxiety, insomnia, posttraumatic stress disorder, and psychological distress among populations affected by the COVID-19 pandemic: A systematic review and meta-analysis. Psychiatry Res. 2021; 295:113599. <https://doi.org/10.1016/j.psychres.2020.113599>. PMID: [33285346](https://pubmed.ncbi.nlm.nih.gov/33285346/)

8. Cénat JM, Farahi SMMM, Dalexis RD, Darius WP, Bekarkhanechi FM, Poisson H, et al. The global evolution of mental health problems during the COVID-19 pandemic: A systematic review and meta-analysis of longitudinal studies. J Affect Disord. 2022; 315:70-95. <https://doi.org/10.1016/j.jad.2022.07.011>. PMID: [35842064](https://pubmed.ncbi.nlm.nih.gov/35842064/).

9. Chen J, Farah N, Dong RK, Chen RZ, Xu W, Yin J, et al. Mental health during the covid-19 crisis in Africa: A systematic review and meta-analysis. Int J Environ Res Public Health. 2021; 18(20):10604. <https://doi.org/10.3390/ijerph182010604>. PMID: [34682357](https://pubmed.ncbi.nlm.nih.gov/34682357/)

10. Dal Santo T, Sun Y, Wu Y, He C, Wang Y, Jiang X, et al. Systematic review of mental health symptom changes by sex or gender in early-COVID-19 compared to pre-pandemic. Sci Rep. 2022; 12(1):11417. <https://doi.org/10.1038/s41598-022-14746-1>. PMID: [35794116](https://pubmed.ncbi.nlm.nih.gov/35794116/)

11. Deng Y, Chen Y, Zhang B. Different prevalence trend of depression and anxiety among healthcare workers and general public before and after the peak of COVID-19 occurred in China: A meta-analysis. Asian J Psychiatr. 2021; 56:102547. <https://doi.org/10.1016/j.ajp.2021.102547>. PMID: [33465749](https://pubmed.ncbi.nlm.nih.gov/33465749/)

12. de Sousa Júnior GM, Tavares VD de O, de Meiroz Grilo MLP, Coelho MLG, Lima-Araújo GL de, Schuch FB, et al. Mental Health in COVID-19 Pandemic: A Meta-Review of Prevalence Meta-Analyses. Front Psychol. 2021; 12:703838. <https://doi.org/10.3389/fpsyg.2021.703838>. PMID: [34621212](https://pubmed.ncbi.nlm.nih.gov/34621212/).

13. Delanerolle G, Zeng Y, Shi JQ, Yeng X, Goodison W, Shetty A, et al. Mental health impact of the Middle East respiratory syndrome, SARS, and COVID-19: A comparative systematic review and meta-analysis. World J Psychiatry. 2022; 12(5):739–765. <https://doi.org/10.5498/wjp.v12.i5.739>. PMID: [35663292](https://pubmed.ncbi.nlm.nih.gov/35663292/)

14. Dragioti E, Li H, Tsitsas G, Lee KH, Choi J, Kim J, et al. A large-scale meta-analytic atlas of mental health problems prevalence during the COVID-19 early pandemic. J Med Virol. 2022; 94(5):1935–1949. <https://doi.org/10.1002/jmv.27549>. PMID: [34958144](https://pubmed.ncbi.nlm.nih.gov/34958144/)

15. Fan FC, Zhang SY, Cheng Y. Incidence of psychological illness after coronavirus outbreak: a meta-analysis study. J Epidemiol Community Health. 2021;75(9):836–42. <https://doi>.org/10.1136/jech-2020-21592. PMID: [33632722](https://pubmed.ncbi.nlm.nih.gov/33632722/)

16. Hosen I, al-Mamun F, Mamun MA. Prevalence and risk factors of the symptoms of depression, anxiety, and stress during the COVID-19 pandemic in Bangladesh: a systematic review and meta-analysis. Glob Ment Health (Camb). 2021; 8:e47. <https://doi.org/10.1017/gmh.2021.49>. PMID: [35145709](https://pubmed.ncbi.nlm.nih.gov/35145709/)

17. Hossain MM, Rahman M, Trisha NF, Tasnim S, Nuzhath T, Hasan NT, et al. Prevalence of anxiety and depression in South Asia during COVID-19: A systematic review and meta-analysis. Heliyon. 2021; 7(4):e06677 ; <https://doi.org/10.1016/j.heliyon.2021.e06677>. PMID: [33898819](https://pubmed.ncbi.nlm.nih.gov/33898819/)

18. Knox L, Karantzas GC, Romano D, Feeney JA, Simpson JA. One year on: What we have learned about the psychological effects of COVID-19 social restrictions: A meta-analysis. Curr Opin Psychol. 2022; 46:101315. <https://doi.org/10.1016/j.copsyc.2022.101315>. PMID: [35398753](https://pubmed.ncbi.nlm.nih.gov/35398753/)

19. Kunzler AM, Röthke N, Günthner L, Stoffers-Winterling J, Tüscher O, Coenen M, et al. Mental burden and its risk and protective factors during the early phase of the SARS-CoV-2 pandemic: systematic review and meta-analyses. Global Health. 2021; 17(1):1–29. <https://doi.org/10.1186/s12992-021-00670-y>. PMID: [33781283](https://pubmed.ncbi.nlm.nih.gov/33781283/)

20. Lee Y, Lui LMW, Chen-Li D, Liao Y, Mansur RB, Brietzke E, et al. Government response moderates the mental health impact of COVID-19: A systematic review and meta-analysis of depression outcomes across countries. J Affect Disord. 2021; 290:364-377. <https://doi.org/10.1016/j.jad.2021.04.050>. PMID: [34052584](https://pubmed.ncbi.nlm.nih.gov/34052584/)

21. Liu X, Zhu M, Zhang R, Zhang J, Zhang C, Liu P, et al. Public mental health problems during COVID-19 pandemic: a large-scale meta-analysis of the evidence. Transl Psychiatry. 2021; 11(1):384. <https://doi.org/10.1038/s41398-021-01501-9>. PMID: [34244469](https://pubmed.ncbi.nlm.nih.gov/34244469/)

22. Necho M, Tsehay M, Birkie M, Biset G, Tadesse E. Prevalence of anxiety, depression, and psychological distress among the general population during the COVID-19 pandemic: A systematic review and meta-analysis. Int J Soc Psychiatry. 2021 Nov;67(7):892-906. <https://doi.org/10.1177/00207640211003121>. PMID: [33794717](https://pubmed.ncbi.nlm.nih.gov/33794717/)

23. Nochaiwong S, Ruengorn C, Thavorn K, Hutton B, Awiphan R, Phosuya C, et al. Global prevalence of mental health issues among the general population during the coronavirus disease-2019 pandemic: a systematic review and meta-analysis. Sci Rep. 2021 ;11(1): 10173. <https://doi.org/10.1038/s41598-021-89700-8>. PMID: [33986414](https://pubmed.ncbi.nlm.nih.gov/33986414/)

24. Pappa S, Chen J, Barnett J, Chang A, Dong RK, Xu W, et al. A systematic review and meta-analysis of the mental health symptoms during the Covid-19 pandemic in Southeast Asia. Vol. 76, Psychiatry and Clinical Neurosciences. 2022; 76(2):41-50. <https://doi.org/10.1111/pcn.13306>. PMID: [34704305](https://pubmed.ncbi.nlm.nih.gov/34704305/)

25. Phiri P, Ramakrishnan R, Rathod S, Elliot K, Thayanandan T, Sandle N, et al. An evaluation of the mental health impact of SARS-CoV-2 on patients, general public and healthcare professionals: A systematic review and meta-analysis. EClinicalMedicine. 2021; 34:100806. <https://doi.org/10.1016/j.eclinm.2021.100806>. PMID: [33842872](https://pubmed.ncbi.nlm.nih.gov/33842872/)

26. Prati G, Mancini AD. The psychological impact of COVID-19 pandemic lockdowns: A review and meta-analysis of longitudinal studies and natural experiments. Psychol Med. 2021; 51(2):201-211. <https://doi.org/10.1017/s0033291721000015>. PMID: [33436130](https://pubmed.ncbi.nlm.nih.gov/33436130/)

27. Robinson E, Sutin AR, Daly M, Jones A. A systematic review and meta-analysis of longitudinal cohort studies comparing mental health before versus during the COVID-19 pandemic in 2020. J Affect Disord; 2022; 296:567-576.. <https://doi.org/10.1016/j.jad.2021.09.098>. PMID: [34600966](https://pubmed.ncbi.nlm.nih.gov/34600966/)

28. Santomauro DF, Mantilla Herrera AM, Shadid J, Zheng P, Ashbaugh C, Pigott DM, et al. Global prevalence and burden of depressive and anxiety disorders in 204 countries and territories in 2020 due to the COVID-19 pandemic. Lancet. 2021; 398(10312):1700-1712. <https://doi.org/10.1016/s0140-6736(21)02143-7>. PMID: [34634250](https://pubmed.ncbi.nlm.nih.gov/34634250/)

29. Schafer KM, Lieberman A, Sever AC, Joiner T. Prevalence rates of anxiety, depressive, and eating pathology symptoms between the pre- and peri-COVID-19 eras: A meta-analysis. J Affect Disord. 2022; 298:364–372. <https://doi.org/10.1016/j.jad.2021.10.115>. PMID: [34740748](https://pubmed.ncbi.nlm.nih.gov/35122433/)

30. Singh RK, Bajpai R, Kaswan P. COVID-19 pandemic and psychological wellbeing among health care workers and general population: A systematic-review and meta-analysis of the current evidence from India. Clin Epidemiol Glob Health. 2021; 11:100737. <https://doi.org/10.1016/j.cegh.2021.100737>. PMID: [33898866](https://pubmed.ncbi.nlm.nih.gov/33898866/)

31. Yan Y, Du X, Lai L, Ren Z, Li H. Prevalence of depressive and anxiety symptoms among Chinese older adults during the COVID-19 pandemic: A systematic review and meta-analysis. J Geriatr Psychiatry Neurol. 2022; 35(2):182–95. <https://doi.org/10.1177/08919887221078556>. PMID: [35245999](https://pubmed.ncbi.nlm.nih.gov/35245999/)

32. Zhang SX, Chen RZ, Xu W, Yin A, Dong RK, Chen BZ, et al. A Systematic Review and Meta-Analysis of Symptoms of Anxiety, Depression, and Insomnia in Spain in the COVID-19 Crisis. Int J Environ Res Public Health. 2022; 19(2):1018. <https://doi.org/10.3390/ijerph19021018>. PMID: [35055841](https://pubmed.ncbi.nlm.nih.gov/35055841/)

33. Zhang SX, Batra K, Xu W, Liu T, Dong RK, Yin A, et al. Mental disorder symptoms during the COVID-19 pandemic in Latin America - a systematic review and meta-analysis. Epidemiol Psychiatr Sci. 2022; 31:e23. <https://doi.org/10.1017/s2045796021000767>. PMID: [35438066](https://pubmed.ncbi.nlm.nih.gov/35438066/)

34. Zhang SX, Miller SO, Xu W, Yin A, Chen BZ, Delios A, et al. Meta-analytic evidence of depression and anxiety in Eastern Europe during the COVID-19 pandemic. Eur J Psychotraumatol. 2022; 13(1):2000132. <https://doi.org/10.1080/20008198.2021.2000132>. PMID: [35186214](https://pubmed.ncbi.nlm.nih.gov/35186214/)

35. Zhao YJ, Jin Y, Rao WW, Li W, Zhao N, Cheung T, et al. The prevalence of psychiatric comorbidities during the SARS and COVID-19 epidemics: a systematic review and meta-analysis of observational studies. J Affect Disord. 2021;287:145–57. <https://doi.org/10.1016/j.jad.2021.03.016>. PMID: [33799032](https://pubmed.ncbi.nlm.nih.gov/33799032/)

36. Abdulla EK, Velladath SU, Varghese A, Anju M. Depression and anxiety associated with COVID- 19 pandemic among healthcare professionals in India- A systematic review and meta-analysis. Clin Epidemiol Glob Health. 2021;12:100888. <https://doi.org/10.1016/j.cegh.2021.100888>. PMID: [34751253](https://pubmed.ncbi.nlm.nih.gov/34751253/)

37. Afridi MZ, Akhtar P, Khan MN. A Systematic Review and Meta-Analysis of Prevalence of Depressive Symptoms among Healthcare Workers in Pakistan during Covid Pandemic. Pakistan Journal of Medical & Health Sciences. 2022; 16(02):2–5. <https://doi.org/10.53350/pjmhs221622>.

38. Aymerich C, Pedruzo B, Pérez JL, Laborda M, Herrero J, Blanco J, et al. COVID-19 pandemic effects on health worker’s mental health: Systematic review and meta-analysis. Eur Psychiatry. 2022; 65(1):e10. <https://doi.org/10.1192/j.eurpsy.2022.1>. PMID: 35060458

39. Chekole YA, Abate SM. Global prevalence and determinants of mental health disorders during the COVID-19 pandemic: A systematic review and meta-analysis. Ann Med Surg (Lond). 2021; 68:102634. <https://doi.org/10.1016/j.amsu.2021.102634>. PMID: [34386226](https://pubmed.ncbi.nlm.nih.gov/34386226/)

40. Ching SM, Ng KY, Lee KW, Yee A, Lim PY, Ranita H, et al. Psychological distress among healthcare providers during COVID-19 in Asia: Systematic review and meta-analysis. PLoS One. 2021; 16(10):e0257983. <https://doi.org/10.1371/journal.pone.0257983>. PMID: [34648526](https://pubmed.ncbi.nlm.nih.gov/34648526/).

41. da Silva FCT, Neto MLR. Psychological effects caused by the COVID-19 pandemic in health professionals: A systematic review with meta-analysis. Biol Psychiatry. 2021; 104:110062. <https://doi.org/10.1016/j.pnpbp.2020.110062>. PMID: [32771337](https://pubmed.ncbi.nlm.nih.gov/32771337/)

42. Dong F, Liu HL, Yang M, Lu CL, Dai N, Zhang Y, et al. Immediate Psychosocial Impact on Healthcare Workers During COVID-19 Pandemic in China: A Systematic Review and Meta-Analysis. Front Psychol. 2021; 12:645460. <https://doi.org/10.3389/fpsyg.2021.645460>. PMID: [34122233](https://pubmed.ncbi.nlm.nih.gov/34122233/)

43. Dutta A, Sharma A, Torres-Castro R, Pachori H, Mishra S. Mental health outcomes among health-care workers dealing with COVID-19/severe acute respiratory syndrome coronavirus 2 pandemic: A systematic review and meta-analysis. Indian J Psychiatry . 2021; 63(4):335-347. <https://doi.org/10.4103/psychiatry.indianjpsychiatry_1029_20>. PMID: [34456346](https://pubmed.ncbi.nlm.nih.gov/34456346/)

44. El-Qushayri AE, Dahy A, Reda A, Mahmoud MA, Mageed SA, Kamel AMA, et al. A closer look at the high burden of psychiatric disorders among healthcare workers in Egypt during the COVID-19 pandemic. Epidemiol Health. 2021;. 43:e2021045. <https://doi.org/10.4178/epih.e2021045>. PMID: [34265893](https://pubmed.ncbi.nlm.nih.gov/34265893/)

45. Ghahramani S, Lankarani KB, Yousefi M, Heydari K, Shahabi S, Azmand S. A Systematic Review and Meta-Analysis of Burnout Among Healthcare Workers During COVID-19. Front Psychiatry. 2021; 12:758849. <https://doi.org/10.3389/fpsyt.2021.758849>. PMID: [34858231](https://pubmed.ncbi.nlm.nih.gov/34858231/)

46. Halemani K, Issac A, Mishra P, Dhiraaj S, Mavinatop S. Prevalence of Anxiety, Depression, Stress, and Insomnia among Healthcare Workers during Covid-19: A Systematic Review and Meta-Analysis. Nursing Journal of India. 2021; CXII(06):269–277. <https://doi.org/10.48029/NJI.2021.CXII605>

47. Hao Q, Wang D, Xie M, Tang Y, Dou Y, Zhu L, et al. Prevalence and Risk Factors of Mental Health Problems Among Healthcare Workers During the COVID-19 Pandemic: A Systematic Review and Meta-Analysis. Front Psychiatry. 2021; 12:567381. <https://doi.org/10.3389/fpsyt.2021.567381>. PMID: [34211406](https://pubmed.ncbi.nlm.nih.gov/34211406/)

48. Hu N, Deng H, Yang H, Wang C, Cui Y, Chen J, et al. The pooled prevalence of the mental problems of Chinese medical staff during the COVID-19 outbreak: A meta-analysis. J Affect Disord. 2022; 303:323–30. <https://doi.org/10.1016/j.jad.2022.02.045>. PMID: [35183620](file:///C:\\Users\\awn233\\AppData\\Roaming\\Microsoft\\Word\\35183620)

49. Johns G, Samuel V, Freemantle L, Lewis J, Waddington L. The global prevalence of depression and anxiety among doctors during the covid-19 pandemic: Systematic review and meta-analysis. J Affect Disord. 2022; 298:431–441. <https://doi.org/10.1016/j.jad.2021.11.026>. PMID: [34785264](https://pubmed.ncbi.nlm.nih.gov/34785264/)

50. Li Y, Scherer N, Felix L, Kuper H. Prevalence of depression, anxiety and post-traumatic stress disorder in health care workers during the COVID-19 pandemic: A systematic review and meta-analysis. PLoS One . 2021 ;16(3):e0246454. <https://doi.org/10.1371/journal.pone.0246454>. PMID: [33690641](https://pubmed.ncbi.nlm.nih.gov/33690641/)

51. Liu X, Wang G, Zhang J, Wang S. Prevalence of depression and anxiety among health care workers in designated hospitals during the COVID-19 epidemic: A meta-analysis. Chinese Journal of Evidence-Based Medicine. 2021; 21(9):1035-1042. Available from: <https://pesquisa.bvsalud.org/global-literature-on-novel-coronavirus-2019-ncov/resource/pt/covidwho-1449172?lang=en>.

52. Mahmud S, Hossain S, Muyeed A, Islam MM, Mohsin M. The global prevalence of depression, anxiety, stress, and, insomnia and its changes among health professionals during COVID-19 pandemic: A rapid systematic review and meta-analysis. Heliyon. 2021;7(7):e07393. <https://doi.org/10.1016/j.heliyon.2021.e07393>. PMID: [34278018](https://pubmed.ncbi.nlm.nih.gov/34278018/)

53. Marvaldi M, Mallet J, Dubertret C, Moro MR, Guessoum SB. Anxiety, depression, trauma-related, and sleep disorders among healthcare workers during the COVID-19 pandemic: A systematic review and meta-analysis. Neurosci Biobehav Rev. 2021; 126:252–64. <https://doi.org/10.1016/j.neubiorev.2021.03.024>. PMID: [33774085](https://pubmed.ncbi.nlm.nih.gov/33774085/)

54. Norhayati MN, Yusof RC, Azman MY. Prevalence of psychological impacts on healthcare providers during COVID-19 pandemic in Asia. Vol. 18, International Journal of Environmental Research and Public Health. 2021; 18(17):9157 <https://doi.org/10.3390/ijerph18179157>. PMID: [34501747](https://pubmed.ncbi.nlm.nih.gov/34501747/)

55. Olaya B, Pérez-Moreno M, Bueno-Notivol J, Gracia-García P, Lasheras I, Santabárbara J. Prevalence of depression among healthcare workers during the covid-19 outbreak: A systematic review and meta-analysis. J Clin Med. 2021; 10(15):3406. <https://doi.org/10.3390/jcm10153406>. PMID: [34362188](https://pubmed.ncbi.nlm.nih.gov/34362188/)

56. Saragih ID, Tonapa SI, Saragih IS, Advani S, Batubara SO, Suarilah I, et al. Global prevalence of mental health problems among healthcare workers during the Covid-19 pandemic: A systematic review and meta-analysis.International Journal of Nursing Studies. Int J Nurs Stud; 2021; 121:104002 <https://doi.org/10.1016/j.ijnurstu.2021.104002>. PMID: [34271460](https://pubmed.ncbi.nlm.nih.gov/34271460/)

57. Ślusarska B, Nowicki GJ, Niedorys-Karczmarczyk B, Chrzan-Rodak A. Prevalence of Depression and Anxiety in Nurses during the First Eleven Months of the COVID-19 Pandemic: A Systematic Review and Meta-Analysis. Int J Environ Res Public Health. 2022; 19(3):1154. <https://doi.org/10.3390/ijerph19031154>. PMID: [35162183](https://pubmed.ncbi.nlm.nih.gov/35162183/)

58. Sun P, Wang M, Song T, Wu Y, Luo J, Chen L, et al. The Psychological Impact of COVID-19 Pandemic on Health Care Workers: A Systematic Review and Meta-Analysis. Front Psychol. 2021; 12:626547. <https://doi.org/10.3389/fpsyg.2021.626547>. PMID: [34305703](https://pubmed.ncbi.nlm.nih.gov/34305703/)

59. Varghese A, George G, Kondaguli S v., Naser AY, Khakha DC, Chatterji R. Decline in the mental health of nurses across the globe during COVID-19: A systematic review and meta-analysis. J Glob Health. 2021; 10;11:05009. <https://doi.org/10.7189/jogh.11.05009>. PMID: [33884193](file:///C:\\Users\\awn233\\AppData\\Roaming\\Microsoft\\Word\\33884193)

60. Xiong N, Fritzsche K, Pan Y, Löhlein J, Leonhart R. The psychological impact of COVID-19 on Chinese healthcare workers: a systematic review and meta-analysis. Soc Psychiatry Psychiatr Epidemiol. 2022; 57(8):1515-1529. <https://doi.org/10.1007/s00127-022-02264-4>. PMID: [35325261](https://pubmed.ncbi.nlm.nih.gov/35325261/)

61. Yan H, Ding Y, Guo W. Mental Health of Medical Staff during the Coronavirus Disease 2019 Pandemic: A Systematic Review and Meta-Analysis. Psychosom Med. 2021; 83(4):387–396. <https://doi.org/10.1097/psy.0000000000000922>. PMID: 33818054

62. Zhang H, Li W, Li H, Zhang C, Luo J, Zhu Y, et al. Prevalence and dynamic features of psychological issues among Chinese healthcare workers during the COVID-19 pandemic: A systematic review and cumulative meta-analysis. Gen Psychiatr. 2021; 34(3):e100344. <https://doi.org/10.1136/gpsych-2020-100344>. PMID: [34192242](https://pubmed.ncbi.nlm.nih.gov/34192242/)

63. Ayubi E, Bashirian S, Khazaei S. Depression and Anxiety Among Patients with Cancer During COVID-19 Pandemic: A Systematic Review and Meta-analysis. J Gastrointest Cancer. 2021; 52(2):499-507. <https://doi.org/10.1007/s12029-021-00643-9>. PMID: [33950368](https://pubmed.ncbi.nlm.nih.gov/33950368/)

64. Dong F, Liu H liang, Dai N, Yang M, Liu J ping. A living systematic review of the psychological problems in people suffering from COVID-19. J Affect Disord. 2021; 292:172–88. <https://doi>.org/10.1016/j.jad.2021.05.060. PMID: [34126309](https://pubmed.ncbi.nlm.nih.gov/34126309/)

65. Khraisat BR, Al-Jeady AM, Alqatawneh DA, Toubasi AA, AlRyalat SA. The prevalence of mental health outcomes among eating disorder patients during the COVID-19 pandemic: A meta-analysis. Clin Nutr ESPEN. 2022; 48:141–147. <https://doi.org/10.1016/j.clnesp.2022.01.033>. PMID: [35331484](https://pubmed.ncbi.nlm.nih.gov/35331484/)

66. Kuroda N, Kubota T. Psychological impact of the COVID-19 pandemic for patients with epilepsy: A systematic review and meta-analysis. Epilepsy Behav. 2021; 124:108340. <https://doi.org/10.1016/j.yebeh.2021.108340>. PMID: [34600283](https://pubmed.ncbi.nlm.nih.gov/34600283/)

67. Lee KW, Ang CS, Lim SH, Siau CS, Ong LTD, Ching SM, et al. Prevalence of mental health conditions among people living with HIV during the COVID-19 pandemic: A rapid systematic review and meta-analysis. HIV Med. 2022; 23(9):990–1001. <https://doi.org/10.1111/hiv.13299>. PMID: [35304829](https://pubmed.ncbi.nlm.nih.gov/35304829/)

68. Liu C, Pan W, Li L, Li B, Ren Y, Ma X. Prevalence of depression, anxiety, and insomnia symptoms among patients with COVID-19: A meta-analysis of quality effects model. J Psychosom Res. 2021; 147:110516. <https://doi.org/10.1016/j.jpsychores.2021.110516>. PMID: [34023580](https://pubmed.ncbi.nlm.nih.gov/34023580/)

69. Natarajan A, Shetty A, Delanerolle G, Zeng Y, Zhang Y, Raymont V, et al. A systematic review and meta-analysis of Long COVID symptoms. medRxiv. 2022 [cited 2022 Dec 12]. 2022.03.08.22272091 [Preprint]. Available from: <https://doi.org/10.1101/2022.03.08.22272091>

70. Premraj L, Kannapadi N v., Briggs J, Seal SM, Battaglini D, Fanning J, et al. Mid and long-term neurological and neuropsychiatric manifestations of post-COVID-19 syndrome: A meta-analysis. J Neurol Sci. 2022; 434:120162. <https://doi.org/10.1016/j.jns.2022.120162>. PMID: [35121209](https://pubmed.ncbi.nlm.nih.gov/35121209/)

71. Sideli L, lo Coco G, Bonfanti RC, Borsarini B, Fortunato L, Sechi C, et al. Effects of COVID-19 lockdown on eating disorders and obesity: A systematic review and meta-analysis . Eur Eat Disord Rev. 2021; 29(6): 826–841. <https://doi.org/10.1002/erv.2861>. PMID: [34460991](https://pubmed.ncbi.nlm.nih.gov/34460991/)

72. Xie Q, Liu XB, Xu YM, Zhong BL. Understanding the psychiatric symptoms of COVID-19: a meta-analysis of studies assessing psychiatric symptoms in Chinese patients with and survivors of COVID-19 and SARS by using the Symptom Checklist-90-Revised. Translational Psychiatry. 2021; 11(1):290. <https://doi.org/10.1038/s41398-021-01416-5>. PMID: [34001863](https://pubmed.ncbi.nlm.nih.gov/34001863/)

73. Adrianto N, Caesarlia J, Pajala FB. Depression in pregnant and postpartum women during COVID-19 pandemic: systematic review and meta-analysis. Obstet Gynecol Sci. 2022; 65(4):287–302. <https://doi.org/10.5468/ogs.21265>. PMID: [35754363](https://pubmed.ncbi.nlm.nih.gov/35754363/)

74. Chen Q, Li W, Xiong J, Zheng X. Prevalence and Risk Factors Associated with Postpartum Depression during the COVID-19 Pandemic: A Literature Review and Meta-Analysis. Int J Environ Res Public Health. 2022; 16;19(4):2219. <https://doi.org/10.3390/ijerph19042219>. PMID: [35206407](https://pubmed.ncbi.nlm.nih.gov/35206407/)

75. Cevik A, Onat Koroglu C, Karacam Z, Gokyildiz Surucu S, Alan S. Effects of the Covid-19 Pandemic on the Prevalence of Insomnia, Anxiety, and Depression During Pregnancy: A Systematic Review and Meta-Analysis. Clin Nurs Res. 2022; 31(8):1405–1421. <https://doi.org/10.1177/10547738221112748>. PMID: [35912813](https://pubmed.ncbi.nlm.nih.gov/35912813/)

76. Tomfohr-Madsen LM, Racine N, Giesbrecht GF, Lebel C, Madigan S. Depression and anxiety in pregnancy during COVID-19: A rapid review and meta-analysis. Psychiatry Res. 2021; 300:113912. <https://doi.org/10.1016/j.psychres.2021.113912>. PMID: [33836471](https://pubmed.ncbi.nlm.nih.gov/33836471/)

77. Safi-Keykaleh M, Aliakbari F, Safarpour H, Safari M, Tahernejad A, Sheikhbardsiri H, et al. Prevalence of postpartum depression in women amid the COVID-19 pandemic: A systematic review and meta-analysis. Int J Gynaecol Obstet. 2022; 157(2):240-247. <https://doi.org/10.1002/ijgo.14129>. PMID: [35122433](https://pubmed.ncbi.nlm.nih.gov/35122433/).

78. Demissie DB, Bitew ZW. Mental health effect of COVID-19 pandemic among women who are pregnant and/or lactating: A systematic review and meta-analysis. SAGE Open Med . 2021 ; 9:205031212110261. <https://doi.org/10.1177/20503121211026195>. PMID: [34262762](https://pubmed.ncbi.nlm.nih.gov/34262762/)

79. Ghazanfarpour M, Bahrami F, Rashidi Fakari F, Ashrafinia F, Babakhanian M, Dordeh M, et al. Prevalence of anxiety and depression among pregnant women during the COVID-19 pandemic: a meta-analysis. J Psychosom Obstet Gynaecol. 2022; 43(3):315-326. <https://doi.org/10.1080/0167482x.2021.1929162>. PMID: [34165032](https://pubmed.ncbi.nlm.nih.gov/34165032/).

80. Shorey SY, Ng ED, Chee CYI. Anxiety and depressive symptoms of women in the perinatal period during the COVID-19 pandemic: A systematic review and meta-analysis. Scand J Public Health . 2021;49(7):730–740. <https://doi.org/10.1177/14034948211011793>. PMID: [33966511](https://pubmed.ncbi.nlm.nih.gov/33966511/)

81. Batra K, Sharma M, Batra R, Singh TP, Schvaneveldt N. Assessing the Psychological Impact of COVID-19 among College Students: An Evidence of 15 Countries. Healthcare (Basel). 2021; 9(2):222. <https://doi.org/10.3390/healthcare9020222>. PMID: [33671363](https://pubmed.ncbi.nlm.nih.gov/33671363/)

82. Chang JJ, Ji Y, Li YH, Pan HF, Su PY. Prevalence of anxiety symptom and depressive symptom among college students during COVID-19 pandemic: A meta-analysis. J Affect Disord . 2021 ; 292:242–54. <https://doi.org/10.1016/j.jad.2021.05.109>. PMID: [34134022](https://pubmed.ncbi.nlm.nih.gov/34134022/)

83. Deng J, Zhou F, Hou W, Silver Z, Wong CY, Chang O, et al. The prevalence of depressive symptoms, anxiety symptoms and sleep disturbance in higher education students during the COVID-19 pandemic: A systematic review and meta-analysis. Psychiatry Res. 2021; 301:113863. <https://doi.org/10.1016/j.psychres.2021.113863> PMID: [33984824](https://pubmed.ncbi.nlm.nih.gov/33984824/)

84. Ebrahim AH, Dhahi A, Husain MA, Jahrami H. The Psychological Well-Being of University Students amidst COVID-19 Pandemic Scoping review, systematic review and meta-analysis. Sultan Qaboos Univ Med J. 2022; 22(2):179–197.<https://doi.org/10.18295/squmj.6.2021.081>. PMID: [35673293](https://pubmed.ncbi.nlm.nih.gov/35673293/)

85. Fang Y, Ji B, Liu Y, Zhang J, Liu Q, Ge Y, et al. The prevalence of psychological stress in student populations during the COVID-19 epidemic: a systematic review and meta-analysis. Sci Rep. 2022; 12(1):12118. <https://doi.org/10.1038/s41598-022-16328-7>. PMID: [35840641](https://pubmed.ncbi.nlm.nih.gov/35840641/).

86. Guo S, Kaminga AC, Xiong J. Depression and Coping Styles of College Students in China During COVID-19 Pandemic: A Systemic Review and Meta-Analysis. Front Public Health. 2021; 9:613321. <https://doi.org/10.3389/fpubh.2021.613321>. PMID: [34307268](https://pubmed.ncbi.nlm.nih.gov/34307268/)

87. Jia Q, Qu Y, Sun H, Huo H, Yin H, You D. Mental Health Among Medical Students During COVID-19: A Systematic Review and Meta-Analysis. Front Psychol. 2022; 13:846789. <https://doi.org/10.3389/fpsyg.2022.846789>. PMID: [35619776](https://pubmed.ncbi.nlm.nih.gov/35619776/)

88. Li Y, Wang A, Wu Y, Han N, Huang H. Impact of the COVID-19 Pandemic on the Mental Health of College Students: A Systematic Review and Meta-Analysis. Front Psychol. 2021; 12:669119. <https://doi.org/10.3389/fpsyg.2021.669119>. PMID: [34335381](https://pubmed.ncbi.nlm.nih.gov/34335381/)

89. Luo W, Zhong BL, Chiu HFK. Prevalence of depressive symptoms among Chinese university students amid the COVID-19 pandemic: A systematic review and meta-analysis. Epidemiol Psychiatr Sci. 2021 ;30:e31. <https://doi.org/10.1017/s2045796021000202>. PMID: [33766163](https://pubmed.ncbi.nlm.nih.gov/33766163/)

90. Carvalho PO, Hülsdünker T, Carson F. The impact of the COVID-19 lockdown on european students’ negative emotional symptoms: A systematic review and meta-analysis. Behav Sci (Basel); 2022; 12(1):3. <https://doi.org/10.3390/bs12010003>. PMID: [35049614](https://pubmed.ncbi.nlm.nih.gov/35049614/)

91. Santabárbara J, Olaya B, Bueno-Notivol J, Pérez-Moreno M, Gracia-García P, Ozamiz-Etxebarria N, et al. Prevalence of depression among medical students during the COVID-19 pandemic. A systematic review and meta-analysis. 2021; 149(11):1579-1588. <https://doi.org/10.4067/s0034-98872021001101579>. PMID: [35735320](https://pubmed.ncbi.nlm.nih.gov/35735320/).

92. Santabárbara J, Ozamiz-Etxebarria N, Idoiaga N, Olaya B, Bueno-Novitol J. Meta-analysis of prevalence of depression in dental students during covid-19 pandemic. Vol. 57, Medicina (Lithuania). 2021; 57(11):1278. <https://doi.org/10.3390/medicina57111278>. PMID: [34833496](https://pubmed.ncbi.nlm.nih.gov/34833496/)

93. Makwana K, Jain S, Makwana A, Rathod NM. Prevalence of depression among Indian medical students in COVID-19 pandemic – A meta-analysis. Natl J Physiol Pharm Pharmacol. 2022; 12(7):916–916. <http://dx.doi.org/10.5455/njppp.2022.12.052331202210062022>

94. Mulyadi M, Tonapa SI, Luneto S, Lin WT, Lee BO. Prevalence of mental health problems and sleep disturbances in nursing students during the COVID-19 pandemic: A systematic review and meta-analysis. Nurse Educ Pract. 2021; 57:103228. <https://doi.org/10.1016/j.nepr.2021.103228>. PMID: [34653783](https://pubmed.ncbi.nlm.nih.gov/34653783/)

95. Wang C, Wen W, Zhang H, Ni J, Jiang J, Cheng Y, et al. Anxiety, depression, and stress prevalence among college students during the COVID-19 pandemic: A systematic review and meta-analysis. J Am Coll Health. 2021; 1–8. <https://doi.org/10.1080/07448481.2021.1960849>. PMID: [34469261](https://pubmed.ncbi.nlm.nih.gov/34469261/)

96. Zhu J, Racine N, Xie EB, Park J, Watt J, Eirich R, et al. Post-secondary Student Mental Health During COVID-19: A Meta-Analysis. Front Psychiatry. 2021; 12:777251. <https://doi.org/10.3389/fpsyt.2021.777251>. PMID: [34955924](https://europepmc.org/article/pmc/pmc8709535)

97. Chen J, Yang K, Cao Y, Du Y, Wang N, Qu M. Depressive Symptoms Among Children and Adolescents in China During the Coronavirus Disease-19 Epidemic: A Systematic Review and Meta-Analysis. Front Psychiatry. 2022; 13:870346. <https://doi.org/10.3389/fpsyt.2022.870346>. PMID: [35463512](https://pubmed.ncbi.nlm.nih.gov/35463512/)

98. Chai J, Xu H, An N, Zhang P, Liu F, He S, et al. The Prevalence of Mental Problems for Chinese Children and Adolescents During COVID-19 in China: A Systematic Review and Meta-Analysis. Front Pediatr. 2021; 9:661796. <https://doi.org/10.3389/fped.2021.661796>. PMID: [34692601](https://pubmed.ncbi.nlm.nih.gov/34692601/)

99. Ma L, Mazidi M, Li K, Li Y, Chen S, Kirwan R, et al. Prevalence of mental health problems among children and adolescents during the COVID-19 pandemic: A systematic review and meta-analysis. J Affect Disord. 2021;293:78–89. <https://doi.org/10.1016/j.jad.2021.06.021>. PMID: [34174475](https://pubmed.ncbi.nlm.nih.gov/34174475/)

100. Panda PK, Gupta J, Chowdhury SR, Kumar R, Meena AK, Madaan P, et al. Psychological and Behavioral Impact of Lockdown and Quarantine Measures for COVID-19 Pandemic on Children, Adolescents and Caregivers: A Systematic Review and Meta-Analysis. J Trop Pediatr. 2021; 67(1):fmaa122. <https://doi.org/10.1093/tropej/fmaa122>. PMID: [33367907](https://pubmed.ncbi.nlm.nih.gov/33367907/)

101. Racine N, McArthur BA, Cooke JE, Eirich R, Zhu J, Madigan S. Global Prevalence of Depressive and Anxiety Symptoms in Children and Adolescents during COVID-19: A Meta-analysis. JAMA Pediatr. 2021;175(11):1142–1150. <https://doi.org/10.1001/jamapediatrics.2021.2482>. PMID: [34369987](https://pubmed.ncbi.nlm.nih.gov/34369987/)

102. Racine N, Eirich R, Cooke J, Zhu J, Pador P, Dunnewold N, et al. When the Bough Breaks: A systematic review and meta-analysis of mental health symptoms in mothers of young children during the COVID-19 pandemic. Infant Ment Health J. 2022; 43(1):36–54. <https://doi.org/10.1002/imhj.21959>. PMID: [34962649](https://pubmed.ncbi.nlm.nih.gov/34962649/)

103. Huang G, Chu H, Chen R, Liu D, Banda KJ, O’Brien AP, et al. Prevalence of depression, anxiety, and stress among first responders for medical emergencies during COVID-19 pandemic: A meta-analysis. J Glob Health. 2022;12:05028. <https://doi.org/10.7189/jogh.12.05028>. PMID: [35871411](https://pubmed.ncbi.nlm.nih.gov/35871411/)

104. Ozamiz-Etxebarria N, Mondragon NI, Bueno-Notivol J, Pérez-Moreno M, Santabárbara J. Prevalence of anxiety, depression, and stress among teachers during the covid-19 pandemic: A rapid systematic review with meta-analysis. Brain Sci. 2021; 11(9):1172 . <https://doi.org/10.3390/brainsci11091172>. PMID: [34573192](https://pubmed.ncbi.nlm.nih.gov/34573192/)

105. Ma K, Liang L, Chutiyami M, Nicoll S, Khaerudin T, Ha X van. COVID-19 pandemic-related anxiety, stress, and depression among teachers: A systematic review and meta-analysis. Work. 2022; 73(1):3–27. <https://doi.org/10.3233/wor-220062>. PMID: [35527618](https://pubmed.ncbi.nlm.nih.gov/35527618/)

106. Li W, Zhang H, Zhang C, Luo J, Wang H, Wu H, et al. The Prevalence of Psychological Status During the COVID-19 Epidemic in China: A Systemic Review and Meta-Analysis. Front Psychol. 2021; 12:614964. <https://doi.org/10.3389/fpsyg.2021.614964>. PMID: [34017278](https://pubmed.ncbi.nlm.nih.gov/34017278/)

107. Pashazadeh Kan F, Raoofi S, Rafiei S, Khani S, Hosseinifard H, Tajik F, et al. A systematic review of the prevalence of anxiety among the general population during the COVID-19 pandemic. J Affect Disord. 2021; 293:391-398. <https://doi.org/10.1016/j.jad.2021.06.073>. PMID: [34246947](https://pubmed.ncbi.nlm.nih.gov/34246947/)

108. Sharma S, Joseph J, Dhandapani M, Varghese A, Radha K, Mathews E, et al. COVID-19 and psychological distress among the general population of India: Meta-Analysis of observational studies. Indian J Community Med. 2022; 47(2):160-165. <https://doi.org/10.4103/ijcm.ijcm_1365_21>. PMID: [36034249](https://pubmed.ncbi.nlm.nih.gov/36034249/)

109. Adibi A, Golitaleb M, Farrahi-Ashtiani I, Pirani D, Yousefi K, Jamshidbeigi Y, et al. The Prevalence of Generalized Anxiety Disorder Among Health Care Workers During the COVID-19 Pandemic: A Systematic Review and Meta-Analysis. Front Psychiatry. 2021; 12:658846. <https://doi.org/10.3389/fpsyt.2021.658846>. PMID: [34135784](https://pubmed.ncbi.nlm.nih.gov/34135784/)

110. Raoofi S, Pashazadeh Kan F, Rafiei S, Khani S, Hosseinifard H, Tajik F, et al. Anxiety during the COVID-19 pandemic in hospital staff: systematic review plus meta-analysis. BMJ Support Palliat Care. 2021; bmjspcare-2021-003125. <https://doi.org/10.1136/bmjspcare-2021-003125>. PMID: [34312187](https://pubmed.ncbi.nlm.nih.gov/34312187/)

111. Rezaei-Hachesu V, Fe’li SN, Maajani K, Hokmabadi R, Golbabaei F. The Global Prevalence of Anxiety, Depression, and Insomnia among Healthcare Workers during the Covid-19 Pandemic: A Systematic Review and Meta-Analysis. Journal of Occupational Health and Epidemiology. 2022; 11(1):48–66. Available from: <https://doi.org/10.52547/johe.11.1.48>

112. Santabárbara J, Bueno-Notivol J, Lipnicki DM, Olaya B, Pérez-Moreno M, Gracia-García P, et al. Prevalence of anxiety in health care professionals during the COVID-19 pandemic: A rapid systematic review (on published articles in Medline) with meta-analysis. Prog Neuropsychopharmacol Biol Psychiatry. 2021; 107:110244. <https://doi.org/10.1016/j.pnpbp.2021.110244>. PMID: [33453320](https://pubmed.ncbi.nlm.nih.gov/33453320/)

113. Liyanage S, Saqib K, Khan AF, Thobani TR, Tang WC, Chiarot CB, et al. Prevalence of anxiety in university students during the covid-19 pandemic: A systematic review. Int J Environ Res Public Health. 2022; 19(1):62. <https://doi.org/10.3390/ijerph19010062>. PMID: [35010323](https://pubmed.ncbi.nlm.nih.gov/35010323/)

114. Santabarbara J, Idoiaga N, Ozamiz-Etxebarria N, Bueno-Notivol J. Prevalence of anxiety in dental students during the covid-19 outbreak: A meta-analysis. Vol. 18, International Journal of Environmental Research and Public Health. Int J Environ Res Public Health; 2021. 18(20):10978. <https://doi.org/10.3390/ijerph182010978>. PMID: [34682726](https://pubmed.ncbi.nlm.nih.gov/34682726/)

115. Qiu D, Li Y, Li L, He J, Ouyang F, Xiao S. Prevalence of post-traumatic stress symptoms among people influenced by coronavirus disease 2019 outbreak: A meta-analysis. Eur Psychiatry. 2021; 64(1):e30. <https://doi.org/10.1192/j.eurpsy.2021.24>. PMID: [33843547](https://pubmed.ncbi.nlm.nih.gov/33843547/)

116. Qiu D, Li Y, Li L, He J, Ouyang F, Xiao S. Infectious Disease Outbreak and Post-Traumatic Stress Symptoms: A Systematic Review and Meta-Analysis. Front Psychol. 2021; 12:668784. <https://doi.org/10.3389/fpsyg.2021.668784>. PMID: [34421723](https://pubmed.ncbi.nlm.nih.gov/34421723/)

117. Salehi M, Amanat M, Mohammadi M, Salmanian M, Rezaei N, Saghazadeh A, et al. The prevalence of post-traumatic stress disorder related symptoms in Coronavirus outbreaks: A systematic-review and meta-analysis. J Affect Disord. 2021; 282:527–538. <https://doi.org/10.1016/j.jad.2020.12.188>. PMID: [33433382](https://pubmed.ncbi.nlm.nih.gov/33433382/)

118. Yunitri N, Chu H, Kang XL. Global prevalence and associated risk factors of posttraumatic stress disorder during COVID-19 pandemic: A meta-analysis. Int J Nurs Stud. 2022; 126:104136. <https://doi.org/10.1016/j.ijnurstu.2021.104136>. PMID: [34856503](https://pubmed.ncbi.nlm.nih.gov/34856503/)

119. Zhang L, Pan R, Cai Y, Pan J. The prevalence of post-traumatic stress disorder in the general population during the COVID-19 pandemic: A systematic review and single-arm meta-analysis. Psychiatry Investig. 2021;18(5):426–433. <https://doi.org/10.30773/pi.2020.0458>. PMID: [33910325](https://pubmed.ncbi.nlm.nih.gov/33910325/)

120. Qi G, Yuan P, Qi M, Hu X, Shi S, Shi X. Influencing Factors of High PTSD Among Medical Staff During COVID-19: Evidences From Both Meta-analysis and Subgroup Analysis. Saf Health Work. 2022;13(3):269–278. <https://doi.org/10.1016/j.shaw.2022.06.003>. PMID: [35784492](https://pubmed.ncbi.nlm.nih.gov/35784492/)

121. Nagarajan R, Krishnamoorthy Y, Basavarachar V, Dakshinamoorthy R. Prevalence of post-traumatic stress disorder among survivors of severe COVID-19 infections: A systematic review and meta-analysis. J Affect Disord; 2022 299:52-59. <https://doi.org/10.1016/j.jad.2021.11.040>. PMID: [34800571](https://pubmed.ncbi.nlm.nih.gov/34800571/)

122. Yang F, Wen J, Huang N, Riem MME, Lodder P, Guo J. Prevalence and related factors of child posttraumatic stress disorder during COVID-19 pandemic: A systematic review and meta-analysis. Eur Psychiatry. 2022; 65(1):e37. <https://doi.org/10.1192/j.eurpsy.2022.31>. PMID: [35726735](PMID:%2035726735)

123. Bussières EL, Malboeuf-Hurtubise C, Meilleur A, Mastine T, Hérault E, Chadi N, et al. Consequences of the COVID-19 Pandemic on Children’s Mental Health: A Meta-Analysis. Front Psychiatry. 2021;12:2125. <https://doi.org/10.3389/fpsyt.2021.691659>. PMID: [34925080](https://pubmed.ncbi.nlm.nih.gov/34925080/)
