# Supplementary material for: COVID-19 and common mental health symptoms in the early phase of the pandemic: An umbrella review of the evidence
Source: PLoS Med. 2023 Apr 25;20(4):e1004206. doi: 10.1371/journal.pmed.1004206 (PMC10129001; doi:10.1371/journal.pmed.1004206)
Supplement: Attachment — Submitted filename: Response_reviewers_revision.docx [file pmed.1004206.s004.docx]

**Reviewer #1:** **This is an interesting umbrella review on the impact of COVID-19 on common mental health symptoms in the early phase of the pandemic. However, there are a few issues needing attention.**

**1) The main result of an increased prevalence of mental health symptoms during the pandemic compared to before is well known and widely studied and reported.** **Many other studies even went on to later phase of the pandemic until 2022 and provided further insight of mental health problems in the populations throughout the pandemic. Now the question is what's novelty of this study? Any new insight not being discovered before?**

Authors’ response: We thank the reviewer for these interesting comments in terms of whether the evidence presented in our paper is up to date and novel. In terms of novelty and inclusion of studies until 2022, as mentioned in our reply to the editor, we revised the paper in such a way that it now also includes reviews published in 2022. These reviews include individual studies published in later phases of the pandemic up till the first months of 2022. Thus, our umbrella review extends the evidence base regarding the mental health effects of the pandemic presented in single reviews and therefore firmly consolidates the overall evidence on mental health consequences of the pandemic.

One review with meta-analyses of fluctuations of mental health during the pandemic was identified by our updated search (Cénat et al., 2022). However, since no longitudinal study-data with pre-pandemic assessment was included in the Cénat et al 2022 review, this paper fell outside the scope of our umbrella review and we therefore did not include it. We however presented the cross-sectional prevalence rates from this review in the supplemental file and this review was rated with AMSTAR2. In the Discussion we do refer to this review of Cénat et al., 2022 and to other individual during pandemic longitudinal studies as well by stating that:

Discussion, Lines 341 – 349:

*“We also conclude, in line with the pooled longitudinal data in the reviews of Cénat et al and Salanti et al [92,148], that largest increases of mental health symptoms such as depression took place in the early phases of the pandemic [10] and during periods of social restrictions [113]. In May-July 2020, the SMD for depression was however still increased and only marginally lower than in March-April 2020 [10] and this is not in line with the continuous decline in anxiety and depression reported by Cenát et al [92] and Salanti et al [148].* *An explanation may be that the more recent reviews that included studies with longer follow-up times were able to capture long-term trends showing prolonged deterioration of symptoms during the pandemic [92,148],* *specifically among more vulnerable individuals, such as females and young people [7,152].*

**2) This umbrella review approach claims to address the issues like inconsistencies, biases and gaps of knowledge. Have the authors achieved all these in the paper and given better and more consistent results? Particularly the very high heterogeneity score is throughout all the pooled analyses, which means there are still inconsistencies and heterogeneities among the pooled studies. In short, have we seen the inconsistency and bias reduced by this umbrella review quantatively and by what measure?**

Authors’ response: This is an important comment and we acknowledge that our umbrella review does not provide a quantitative measure or metric that reduces the high amount of heterogeneity extracted from the reviews. We however did not calculate the summary average effect using the random-effects robust variance estimation method across all meta-analyses which also made further quantification of heterogeneity between reviews of studies impossible. With our umbrella review we however try to provide a transparent overview of the enormous number of reviews of individual studies in terms of impact of COVID-19 on the most common mental health outcomes (i.e., prevalence rates of above cut-off scores of symptoms of depression, anxiety and PTSD and mean changes/differences during- compared to pre-pandemic or pre-lockdown). Teams of researchers/research-assistants have independently assessed the methodological quality of each review and we particularly highlight and qualitatively summarize the findings from reviews pooling longitudinal data in the main text of the paper, and included the heterogeneity scores and synthesized subgroup- or moderator analyses of each review in the Supplemental file S2 Text. Following the steps of Fusar-Poli and Radua for umbrella reviews (2018), we thus do provide heterogeneity score of each individual review. According to these authors, qualitative umbrella reviews should also provide tables or detailed forest plots of results and tables for each judgment criterion on methodological quality, which we do indeed provide. We however acknowledge that it is a limitation that we have not quantitatively assessed the pooled data of prevalence rates nor did we calculate summary statistics of the high heterogeneity across meta-analyses.

In the introduction and in the abstract, we re-wrote several lines in a way that it is clear that we used a qualitative umbrella review approach and made this approach more in line with the aims:

Introduction, see Lines 76 to 83:

*“A critical evaluation of the current research evidence is needed to correctly inform the global mental health response to mitigate (future) disruptions and to adapt research strategies and implementation of interventions to address COVID-19 related mental health problems in populations where needed most, such as in young people [13,14]. We aimed to provide an overview of the evidence base on common mental health disorder symptoms during the pandemic, ideally compared to pre-pandemic periods, using an umbrella review approach. With this qualitative approach, inconsistencies and gaps of knowledge in the evidence may be recognized [15–18].”*

Abstract, Line 5:

*“… we qualitatively summarized…”*

Furthermore, we now added this as an important limitation in the Discussion, lines 433-435:

*“**First, because we summarized the pooled prevalence rates and standardized mean changes and the heterogeneity metrics and subgroup analyses from each individual meta-analysis qualitatively, quantitative reduction of inconsistencies or biases across reviews was lacking.”*

Finally, in our revised Discussion, we now better emphasize the inconsistencies across reviews with meta-analyses of longitudinal studies by theoretically explaining these inconsistencies in several places throughout the Discussion, for example in:

Lines 357-362: *“…. This is in line with the longitudinal study of Aknin et al and the meta-analysis of Salanti et al [148,151] but in contrast with other reviews of longitudinal data [10,59]. The Bayesian meta-regression and meta-analysis approaches in the Santomauro et al and Salanti et al reviews that use additional informative data (e.g. from cross-sectional samples or during pandemic longitudinal data) are more powerful and this may explain the differences with other reviews of longitudinal data with conventional random-effects meta-analysis.”*

**3) Public health and social measures (PHSMs). What exactly are PHSMs? Have the authors considered the impact of different lock down measures and durations on mental health symptoms such as strict lockdown in China and relatively soft lockdown in UK? How about financial support in each country to relieve the mental health symptoms? These are just a few factors. Without detailed subgroup analyses on these important factors, the pooled results are unlikely to be consistent and less biased.**

Authors’ response: Public health and social measures (PHSMs) are measures or actions by individuals, institutions, communities, local and national governments and international bodies to slow or stop the spread of an infectious disease, such as COVID-19. It is a definition used by the World Health Organization (WHO). We closely collaborated with the WHO during the course of the development of the umbrella review and we used this terminology in the scientific brief we developed (<https://www.who.int/publications/i/item/WHO-2019-nCoV-Sci_Brief-Mental_health-2022.1>). However, we can imagine in the scientific field, it is not so common to use the acronym PHSM and we therefore changed the text throughout the paper accordingly in several places throughout the text.

For example in Introduction:

Line 49: *“….as well as an indirect psychological* *impact through implementation of public health measures and social restrictions and its longer-term socio-economic consequences [3].”*

Indeed, factors related to the lockdowns, public health measures and social restrictions including financial support did differ across countries. With our updated search we found additional evidence of the effect of strict versus moderate or low social restrictions on for example depression symptoms (Knox et al., 2021) and integrated these in our revised paper. However, many reviews including studies from multiple countries and continents were not adequately examining these factors in subgroup analyses to explain the high heterogeneity. This is a limitation to our findings and we agree that we could have emphasized that better in the Discussion.

We therefore added the following in in the Discussion:

Lines 317-322:

*“Similar discrepancies were found in terms of country-level or COVID-19 exposure factors with some meta-analyses showing no evidence that factors such as continent, COVID-19 case/death rate, or economic situation or support explained the heterogeneity between longitudinal studies of general populations [10,59], while others found that region (e.g. Europe vs. China), decreased human mobility and higher daily SARS-CoV-2 infection rates were associated with depression and anxiety symptoms during the pandemic or during social restrictions [5,113].”*

Lines 362-366:

*“Still, heterogeneity in most reviews was high and largely unexplained. This may be due to the sparse assessment of multiple COVID-19 exposure or individual-level factors such as economic support or situation on mental health symptoms in longitudinal studies. Also, COVID-19 exposure factors such as numbers of cases and stringency of the measures are strongly correlated over time in terms of their influence on mental health outcomes and difficult to disentangle [5,10].* *This issue with the COVID-19 exposure factors calls for a more integrative approach to examine the inter-relatedness of several social-, economic-, and behavioral factors that may explain the impact of public health measures and social restrictions during pandemics on mental health [113,152]. “*

In relation to the high heterogeneity that remained largely unexplained, we also added the following research recommendation in the Discussion in Lines 418-423:

*Second, individual-level socio-economic and pandemic exposure factors including time as continuous factor (instead of estimations based on time-intervals or averages [5,59]) should be assessed more comprehensively and accurately. Third, accurate exposure trackers such as the OXFORD COVID-19 Government Response Stringency and Google mobility indices should be linked to longitudinal within-person data from probabilistic or national representative samples [11].*

**4) Table 2 is a bit confusing. Should it be during pandemic vs before rather than before vs during pandemic? So far, it looks like there are more depression and anxiety before pandemic.**

Authors’ response: Thank you for your attention paid to Table 2, we agree it is confusing and have adjusted the wording accordingly in all relevant places in the Table and in the text into: “During- vs pre-pandemic”.

**Reviewer #2:** **Many thanks for your kind invitation to review the manuscript titled: ''Impact of COVID-19 on common mental health symptoms in the early phase of the pandemic: an umbrella review of the evidence''. This is a well-designed and well executed umbrella review of the literature and I would like to commend the authors for their work. Despite its numerous limitations, I believe it will make a significant contribution to the literature and shed some light in the relatively 'muddy' and often contradictory findings around the impact of COVID-19 on mental health worldwide. I only suggest some minor points for revision:**

1. **The finding that healthcare workers' mental health was not greatly affected when comparing pre- and during the pandemic needs to be further interpreted. It would be important if the authors suggested some explanations for this (?unexpected) phenomenon.**

Authors’ response: We do agree with the reviewer that this finding is unexpected. Please note that no meta-analyses with longitudinal during- versus pre-pandemic data in healthcare workers were retrieved by our two searches. We have now made this more explicit in the Results section:

Line 244-245: *“No meta-analyses of longitudinal studies in healthcare workers were available.”*

Furthermore, in the Discussion we now add further explanations by adding the following information in lines 378 to 388:

*“**Furthermore, in healthcare workers and in a mixed patient population, the during pandemic mental health symptoms were not significantly higher compared to matched pre-pandemic data [43].* *It needs to be emphasized here that longitudinal within-person during- and pre-pandemic data were lacking* *among health workers and mixed-patient populations, impeding strong conclusions regarding the pandemic-related changes in mental health in these groups. However, subgroup-findings of cross-sectional prevalence rates cautiously suggested that pandemic exposure factors (e.g. COVID-19 mortality rate, region) negatively affected specific workers and patients (i.e., females, nurses, younger workers or more severely infected patients). This is in line with recent individual longitudinal studies in these populations, showing that anxiety among healthcare workers including females and young was increased when exposure rates were highest, particularly shortly before a lockdown phase [7,154,155].”*

2. **The same stands for people with pre-existing mental health problems. It would be important if the authors expanded on the potential reasons of why this group was not particularly affected by Covid-19 (e.g. the 'pulling together' phenomenon has been suggested for the non-increase in serious mental illness during the initial phases of the pandemic).**

Authors’ response: That mental-health of people with mental disorders did not deteriorate during the initial phases of the pandemic may be explained by several phenomena. Indeed, one of these might be the often found observation that large scale crises may have a positive effect on social cohesion and being less self-focused in turn improving mental wellbeing with e.g., reducing suicidality rates, at least in the early post-impact phase (Travis-Lumer et al., 2021). Another explanation may be a ceiling effect, in which the amount of symptoms in people with pre-existing mental disorders is already so great that the impact of COVID-19 caused milder elevations for them than for others or that there is great individual variation with some even benefiting from the lockdown in terms of reduced (social) pressures.

We therefore added the following information in the Discussion and refer to the paper of Travis-Lumer et al. (2021):

Lines 391-401:

*However, that patients with mental disorders showed no such deterioration [10], seems inconsistent with findings indicating greater vulnerability in people with a history of mental disorders at least on the longer run during the pandemic [7]. This inconsistency may be explained by several phenomena. One is that pandemic mental health indicators like suicidality, although outside the scope of this review, tend to decrease in the initial phase of a disaster when people are less self-focused but may rise again when the situation is normalized and more long-term negative (socio-economic) consequences become apparent [156]. This inconsistency may also be explained by great individual variation among people with mental disorders, with a pandemic- or lockdown related increase of anxiety and depression symptoms for some but a beneficial effect for others (e.g. due to reduced social pressures). A ceiling effect may play a role as well, with symptoms already being that high that large increases cannot be expected [157].*

**I was wondering whether there was any data collected or any comment that could be made on suicidality of the groups investigated?**

Authors’ response: The impact of COVID-19 on suicidality has been included in our WHO scientific brief based on a living systematic review and update performed by colleagues Prof. David Gunnell and Prof. Ann John. A separate focus on suicidality was however outside the scope of the current umbrella review. However, as mentioned above, we related to suicidality in our review by providing an additional explanation and reference to the longitudinal study of Travis-Lumer on the trajectory of suicidality during the pandemic (see our response to issue 2, Reviewer #2).

3. **Finally, although the authors indicate that one of the study's aims is 'to identify targets for clinical- and policy interventions' , this is not done adequately in my opinion. It is suggested that the authors add a separate, distinct, small paragraph on policy interventions and future implications of their study's findings**

Authors’ response: We thank the reviewer for this suggestion and have now added a paragraph on implications of our umbrella review by adding the following paragraph in the Discussion:

Lines 414 – 428:

*“The findings presented here have implications for mental health researchers, policy makers and public health professionals involved in current and future public health crises. First, more powerful meta-analysis methods with e.g., Bayesian approaches, individual participant data from multiple countries and linear and non-linear assessments of change, should be employed to more accurately plot mental health trajectories. Second, individual-level socio-economic and pandemic exposure factors including time as continuous factor (instead of estimations based on time-intervals or averages [5,59]) should be assessed more comprehensively and accurately. Third, accurate exposure trackers such as the OXFORD COVID-19 Government Response Stringency and Google mobility indices should be linked to longitudinal within-person data from probabilistic or national representative samples [11]. Finally, policy interventions could be developed in such a way that it includes repeated and systematic data collection in vulnerable groups, for example through population panels. Since a digital- and tele-mental health revolution unfolded during the pandemic [159], these developments may be incorporated in remote mental health care interventions making monitoring and prevention- and treatment scalable and cost-effective socio-economic approach for vulnerable individuals [160].”*

**Reviewer #3:** Thank you for the opportunity to review this umbrella review. This study represents an important contribution to the literature. I have only a few comments:

1) **The authors are entirely correct in my view to prioritise reviews with meta-analyses focused on longitudinal data or studies with comparative pre-pandemic prevalence data. However, the rationale for prioritising this type of study needs to be better motivated in the introduction, expanding on the point that "cross-sectional primary studies [...] do not allow any causal inferences to the pandemic". For instance****, highlighting that high prevalence estimates during the pandemic are not meaningful in the absence of comparative data collected with methodological consistency (most crucially sampling), the importance of probability-based sampling when generating prevalence estimates (e.g. Pierce, M., McManus, S., Jessop, C., John, A., Hotopf, M., Ford, T., ... & Abel, K. M. (2020). Says who? The significance of sampling in mental health surveys during COVID-19. The Lancet Psychiatry, 7(7), 567-568.), and the advantages of examining the same people over time when representative data is unavailable or the comparability of sampling is compromised. Essentially including a paragraph motivating why the review views studies with comparative pre-pandemic data as at lower risk of bias.**

Authors’ response: We thank the reviewer for these important adjustments for our paragraph on the methodological weaknesses that make our prioritization for the reviews with longitudinal within-person data more transparent. We do agree that the fact that some studies on the mental health impact of COVID-19 may have used a longitudinal design, may not rule out the risk of bias, particularly in terms of sampling bias. We therefore, in the Introduction re-wrote this paragraph and now refer to the relevant publication of Pierce et al., 2020.

Added in the Introduction lines 71 to 78:

*“ Importantly, most individual studies have cross-sectional designs and lack pre- to during pandemic longitudinal data, which makes causal inferences to the pandemic difficult. Furthermore, even when multiple during- and pre-pandemic assessments have been performed in surveys, respondents often come from non-representative convenience samples while a probability sampling approach is lacking [11]. Hence, assessments of mental health outcomes are often not based on structured clinical interviews or validated questionnaires with established cut-offs and methodological quality or risk of bias assessment of primary studies is often lacking.”*

2) **Please amend the below point in the introduction. Sun et al. include convenience samples in their 'general population' samples (e.g. Benz et al, Katz et al.) and I don't see subgroup analyses of 'population-based' studies. There are also only a handful of general population studies examined in Sun et al. When a larger number of longitudinal studies with representative sampling are examined an increase in mental health symptoms in the initial stages of the pandemic is found (see Robinson et al. review Table 2** [**https://doi.org/10.1016/j.jad.2021.09.098**](https://doi.org/10.1016/j.jad.2021.09.098)**). "Although findings from population-based studies in the initial stages of the pandemic indicate that most people were resilient and did not experience increases in distress [4]": Sun Y, Wu Y, Bonardi O, Krishnan A, He C, Boruff JT, et al. Comparison of Mental Health Symptoms prior to and during COVID-19: Evidence from a Living Systematic Review and Metaanalysis. [cited 23 Jun 2022]. doi:10.1101/2021.05.10.21256920**

Authors’ response: We agree with this reviewer that referring to the living systematic review of Sun et al., was not correct here. We actually meant to refer here to the Pierce et al., (2021) study with probabilistic sampling approach as an example for early studies that showed distinct mental health latent class trajectories with pre- and early pandemic indicating that across the first 6 months of the pandemic, the majority (76%) actually showed a resilient trajectory and 12% only showed initial deterioration of mental health but recovered to pre-pandemic levels later on in the first year of the pandemic while the remaining 12% was characterized by poorer mental health throughout. These findings are in line with the sentence above and we have corrected the reference [4].

See lines 50-51, Introduction:

*“Although findings from population-based studies in the initial stages of the pandemic indicate that most people were resilient and did not experience increases in distress [4],…”*

**3) When discussing results from Table 2 it may make more sense to highlight the design of the studies included (exclusively longitudinal studies vs. estimates including comparisons to different pre-pandemic samples) as this may be a key factor in explaining discrepancies between estimates (e.g. point estimates of .67 vs. .23 differ substantially below with no indication how the studies they are based on differ in their design). Currently, how these findings are discussed seems contradictory in places (e.g. the prevalence of anxiety was higher in the pandemic by 0.40 SMD but not significant over time, SMD = 0.05 in May-July) but is most likely a function of the types of studies estimates are based on.**

Authors’ response: We agree that the presentation of the results in Tables 2 and in the Results text could have been made more accessible to readers. For example, in our original version of the paper, the findings from meta-analyses of studies with longitudinal designs are presented together with those from pairwise meta-analyses from during pandemic cross-sectional studies with matched pre-pandemic cross-sectional studies. In the revised version we present these findings separately now and in Table 2 we have added a column with the designs included in each meta-analysis to further improve the interpretability. For anxiety in the general population we have changed the text as well:

See for example lines 205-209:

*“Anxiety disorder symptoms were also higher during- compared to pre-pandemic periods (SMC 0.12, 95% CI 0.02 to 0.23) [10]; 25.6% increase, 95% CI: 23.2 to 28.0 [5]) as were anxiety and posttraumatic stress symptoms when pooled together (Hedges’ g: 0.18, 95% CI: 0.07 to 0.27) [59]. Anxiety symptoms during pandemic were also increased compared to pre-pandemic data (SMD: 0.40, 95% CI:0.15 to 0.65) [43].”*

4) **Discussion - increases in mental health symptoms during the public health and social measures need to be contextualised in relation to studies explicitly estimating the link between COVID-19 policy stringency and mental health measures which have found this link to be small - Aknin, L. B., Andretti, B., Goldszmidt, R., Helliwell, J. F., Petherick, A., De Neve, J. E., ... & Zaki, J. (2022). Policy stringency and mental health during the COVID-19 pandemic: a longitudinal analysis of data from 15 countries. The Lancet Public Health, 7(5), e417-e426.**

Authors’ response: In our revised Discussion section, attention is now paid to the link between COVID-19 exposure factors such as the stringency index and mental health outcomes. With our updated search we included the review of Knox et al., (2021), estimating the impact of several levels of strictness of social restrictions on overall mental health and depression and anxiety symptoms and the review of Santomauro et al. (2021) also finding a relation between reduced mobility and increased mental health problems. We discuss these findings in relation to the reviews not finding such relations with COVID-19 exposure factors in subgroup analyses (Robinson et al., 2021 and Prati and Manincini 2021).

Discussion, Lines 366-370:

*“Also, COVID-19 exposure factors such as numbers of cases and stringency of the measures are strongly correlated over time in terms of their influence on mental health outcomes and difficult to disentangle [5,10]. This issue with the COVID-19 exposure factors calls for a more integrative approach to examine the inter-relatedness of several social-, economic-, and behavioral factors that may explain the impact of public health measures and social restrictions during pandemics on mental health [113,152].”*

**5) Discussion -please note where the below result can be found in the results section:**

**"Meta-regression analyses showed higher prevalence rates of mental health symptoms predominantly in early pandemic as well as an increasing trend between January to September 2020"**

Authors’ response: Because our Discussion was thoroughly revised, we no longer include this sentence and findings in a similar way but explain the discrepancy in fluctuations over time during the pandemic thereby referring to the correct source (see our feedback to the first comment of #Reviewer 1).

6) **The authors may be over-interpreting trends in cross-sectional estimates which is a concern particularly given the point on this data not allowing causal inferences made in the introduction. For instance, many studies have found age-differences in distress and common mental health symptoms to be typical immediately prior to the pandemic (e.g. for the UK Daly, M., Sutin, A. R., & Robinson, E. (2020). Longitudinal changes in mental health and the COVID-19 pandemic: Evidence from the UK Household Longitudinal Study. Psychological medicine, 1-10.).**

**The fact that there are also age differences in mental health symptoms in (cross-sectional) data collected during the pandemic may say nothing about the effect of the pandemic. Also, an increasing trend in cross-sectional prevalence estimates could be driven by differences in sample composition or representativeness over time. Can the authors address this point?**

Authors’ response: We thank the reviewer for this important feedback. We agree that we should be more careful when interpreting findings of higher prevalence rates derived from subgroup or moderator analyses of cross-sectional study-data. Because of a general scarcity of reviews of longitudinal studies in some specific populations we do however report the findings from the meta-analyses of cross-sectional studies. To make the information from the Tables D, E and F in the Supplemental file more accessible, we have added Table 3 in the revised version that summarizes the ranges in prevalence rates from meta-analyses of cross-sectional studies in all populations. Although we made some cautionary remarks in the original version of the paper, we have added more cautionary remarks throughout the text for example when interpreting the findings of subgroup analyses from cross-sectional pooled data.

Introduction, lines 58 to 64:

*“A large number of cross-sectional and longitudinal studies on the mental health effects of COVID-19 across the general population and vulnerable groups has been published. These studies have been integrated in numerous systematic reviews and meta-analyses and suggest a significant negative effect of the pandemic on mental health, mainly by presenting pooled prevalence rates from cross-sectional studies. However, the few reviews of longitudinal pooled data show less pronounced increases or are contradictory in terms of impact of the pandemic, for example on subgroups [8–10].”*

Discussion, lines 323 to 329:

*“Pooled cross-sectional data from general- and specific populations and healthcare workers indicated wide ranges of prevalence rates. High heterogeneity was often explained by assessment tools and cut-offs used, sampling procedures and quality of reviews. Also, higher rates were found in females, in certain regions, in acute or clinically severe COVID-19 patients, in frontline versus non-frontline healthcare workers and related to some COVID-19 exposure factors such as mortality rate. However, these findings need to be interpreted with caution and no causal inferences can be made due to the cross-sectional designs of the studies pooled in these meta-analyses.”*

Instead, in the revised paper we emphasize additional findings from recent reviews of longitudinal studies in terms of age- and gender differences.

Lines 316 – 321:

*“Although the high heterogeneity between studies with pre- and during pandemic data could not be explained by differences in age and gender in some reviews [10,59], other reviews did show that mental health of females and younger age groups including children between 5 and 13 years was more affected by the pandemic or by social restrictions [5,43,89,98,113].”*

Lines 370 to 372:

*“That females and younger age groups experienced a larger deterioration in mental health during the pandemic is a rather consistent finding across reviews presented here and in line with recent longitudinal studies with national representative or probabilistic samples [4,147].”*

**7) Please include page/line numbers.**

These have been included in the revised paper now.

**8) Concluding section summarises the trends identified v. well.**

**Reviewer #4:**

1. **This review is a major undertaking, and the approach is very thorough. It is striking just how many individual reviews were published within a few months of the onset of the pandemic. 577 reviews passed initial selection procedures, although this was whittled down to 58. Given that COVID-19 was unknown in most countries until 2020, this is an astonishing number. One has the impression that almost as many reviews as primary articles were published over this period. The authors rightly point out the importance of studies that included pre-pandemic data so that change in mental health could be examined.**

Authors’ response: Thank you. In the revised version a similar number of reviews was added by updating the search period up to half of August 2022.

2.	**The main findings are summaries of the meta-analyses and selective presentations of individual findings. They show considerable heterogeneity. It could be made clearer how some conclusions are drawn. For example, the conclusion that healthcare workers showed no rise in anxiety and depression during the early months of the pandemic appears to be based on a single meta-analysis (Kunzler et al, 2021), but tables D and E list numerous other reviews that drew different conclusions. This is only one comparison, but needs clarification.**

Authors’ response: We agree that the conclusion of no rise in anxiety and depression in healthcare workers is primarily based on one review paper comparing during pandemic symptoms to pre-pandemic symptoms in matched studies with healthcare workers. Indeed numerous other reviews with pooled prevalence rates from cross-sectional studies have been included in Tables D, E and F (S2 Text). However, the conclusions of these latter reviews are biased because of design issues emphasized both in the Introduction and Discussion (see our responses to comment 6 of #Reviewer 3 and to comment 1 of #Reviewer2). We now more clearly distinguish these differences in results from reviews with meta-analyses of longitudinal data versus those of cross-sectional data.

We adapted the following information in the Results section, Lines 244-249:

***“Health care workers.*** *No meta-analyses of longitudinal studies in healthcare workers were available. Based on during pandemic prevalence data and pre-pandemic comparative data, no significant differences in symptoms of depression and anxiety were found (SMD: -0.16, 95% CI: -0.59 to 0.26 and SMD: -0.08, 95% CI: -0.66 to 0.49, respectively, Table 2) and rates were not affected by COVID-19 patient contact [43]. As shown in Table 3, apart from some outliers, pooled cross-sectional prevalence of above cut-off depression-, anxiety- and PTSD- symptom levels during pandemic ranged …”*

**3.	Some clarification is also needed of the term 'young people'. Do the authors mean young adults, or does this group include children? Was there a lower age limit on the studies considered?**

Authors’ response: We agree with the reviewer that the term ‘young people’ may be confusing. We therefore chose to more explicitly define subgroups of young people as either children and adolescents or students in the Results section and Tables (by changing the heading of these subsections accordingly) and in the Discussion. We now more closely adhere to the source reviews in terminology. For example, Robinson et al distinguish mental health changes in samples of child/adolescents from changes in samples of (university) students). There was no lower age limit accept when source reviews used one such as the pooled longitudinal data in children between ages 5 to 13 in the Bussieres et al. review.

Results, lines 279-285:

***“Students.*** *A non-significant small increase from pre- to during pandemic in mental health problems (including anxiety and depression) was found for university students (SMC: 0.13, 95% CI: -0.01 to 0.27) [10]. Apart from outliers, cross-sectional prevalence rates of above cut-off depression and anxiety symptoms in students ranged from 23% to 39% and from 28 to 44%, respectively and prevalence for above cut-off PTSD level was 30% (Table 3). Prevalence rates were higher in certain regions (e.g., non-China)[71,109,130], in females [28,104], and for specific assessment tools [32,90,109].*

Results, lines 286 to 290:

***“Children and adolescents.*** *During- compared to pre-pandemic mental health disorder symptoms in children and adolescents were not significantly increased in a meta-analysis of exclusively longitudinal data (SMC: 0.11, 95% CI: -0.03 to 0.26) [10] but in children between 5 and 13 years of age symptoms were significantly higher when based on longitudinal and cross-sectional compared to retrospective pre-pandemic data ([89] SMC 0.28; 95% CI 0.15 to 0.41) (Table 2).*

4.	**One of the authors' conclusions is that people with pre-existing physical health conditions were at increased risk for mental ill-health. It is not clear from the description here whether this category included people with COVID-19 infection, or only those with a long-term condition unrelated to the virus. I am also confused by the conclusion that people with pre-existing physical health conditions had a higher prevalence, but that 'patients (including those with COVID-19 infections)' did not. Surely these 'patients' had pre-existing health conditions?**

Authors’ response: In the Results section, Table 2, the new Table 3 and the Tables D, E and F in the Supplemental file S2 Text, we have disentangled the findings for each group separately when possible. For example, the Robinson et al., review examined the increase in people with pre-existing physical- and mental health disorders separately. With pre-existing, we literally mean ‘existing before the COVID-19 pandemic’ and these do not include COVID-19 patients. However, we do agree that there is some confusion since in the review of Kunzler et al., (2021), changes of during pandemic prevalence rates were compared to pre-pandemic prevalence rates were assessed in ‘any patients’ and this included both people with a mental- or somatic- disorders (e.g. eating disorder, or cancer and COVID-19 patients). If this is the case, we now more explicitly state that in the Results.

Results section; we changed the heading of this sub-section into:

Line 259:

***“People with pre-existing physical- or mental health disorders, or people infected with COVID-19”***

Results section, Lines 266 to 269:

*“In a mixed population of patients with mental, physical or COVID-19 diseases [43], depression and anxiety symptom levels were higher but not significantly different from those in matched studies with pre-pandemic prevalence data (SMD: 0.48, 95% CI:-0.08 to 1.04; SMD: 0.31, 95% CI:-0.07 to 0.69 respectively) [43] (Table 2).”*

5.	**The authors emphasize that mental health was poorer during periods of PHSMs, and give the impression that PHSMs contributed to this rise. There are two issues here that are not adequately taken into account. First, the primary source for this finding (the meta-analysis by Prati & Mancini, 2021) did not compare periods during the COVID-19 pandemic when PHSMs were in force and not in force, but compared mental health pre-pandemic vs pandemic. Thus their conclusion is really that mental health was poorer during the pandemic compared with before. The way to evaluate the impact of PHSMs is to compare months during the pandemic that had higher or lower restrictions in the relevant country (using something like the Oxford Stringency index). This has been done in more recent studies beyond the scope of the reviews included here, though I'm not aware of meta-analyses in this area. Second, PHSMs typically coincide with periods of greater COVID-19 infection in the population, and this itself likely leads to increases in anxiety and depression. I don't think it is possible to infer that PHSMs themselves are responsible for any changes in mental health from the findings of present study; the Abstract and Conclusions should be modified accordingly.**

Authors’ response: We thank the reviewer for raising these two important comments. In response to the first issue, we indeed acknowledge that although authors of reviews (such as Prati & Mancini, and the newly added reviews of Knox et al and Bussieres et al) emphasize that they examined the effect of lockdowns or social restrictions relative to periods of no lockdown or less restrictions, we need to be clear that they based their findings on pooled data from longitudinal designs with a combination of during- and pre-pandemic assessments. It may help readers that we now added a column with the designs of the individual studies included (as suggested by Reviewer #3).

In response to the second issue, we agree that the Oxford stringency index per month and country should ideally be used to assess the impact of the restrictions on mental health. We relate to this issue in relation to the included findings from Santomauro et al and Knox et al. in the Discussion. We also refer to findings from a recent meta-analysis (Salanti et al., Nov. 2022) of the trajectory of mental health symptoms during the first year of the pandemic. This review showed a significant worsening of depression and anxiety symptoms in the first 2 months of the pandemic (SMD -0.39) in line with our umbrella review, and also found that COVID-19 exposure such as stringency measures were indeed strongly related to surges in COVID cases. In the Santomauro review, meta-regression the COVID-19 infection rate and decreased mobility were also highly correlated with COVID-19 mortality rate. So we agree with the reviewer that the social restrictions and public health measures alone cannot account for the increase of mental health problems in the general population but that these are intertwined with the burden and stress related to the increase of COVID-cases and mortality rate.

We changed the Discussion text, lines 362 to 370:

*“Still, heterogeneity in most reviews was high and largely unexplained. This may be due to the sparse assessment of multiple COVID-19 exposure or individual-level factors such as economic support or situation on mental health symptoms in longitudinal studies. Also, COVID-19 exposure factors such as numbers of cases and stringency of the measures are strongly correlated over time in terms of their influence on mental health outcomes and difficult to disentangle [5,10]. This issue with the COVID-19 exposure factors calls for a more integrative approach to examine the inter-relatedness of several social-, economic-, and behavioral factors that may explain the impact of public health measures and social restrictions during pandemics on mental health [113,152].”*

Abstract, Conclusions:

Lines 36 – 39:

*“Explanatory individual-level-, COVID-19 exposure- and time-course factors were scarce and showed inconsistencies across reviews. Policy and research recommendations based on these findings are the use of repeated assessments of mental health in population panels inclusive of vulnerable individuals to respond to current and future health crises that likely impact mental health.”*

6.	Finally, the authors recognize that the reviews included in their coverage are limited to the early months of the pandemic globally. No mention is made of changes in the incidence of COVID infection, the likely impact of vaccination, and the course of the pandemic in 2021 and 2022. How important are the changes in mental health detailed here in the long run? The authors plead for more research particularly in low and middle income countries, but how relevant will new studies be at this stage, given the trajectory of the pandemic? A stronger argument needs to be made as to why these findings are not only of historical interest, but matter now.

Authors’ response: In our revision we have now related to findings of fluctuations of symptoms in relation to COVID-19 exposure factors in various places throughout the Discussion (please see our replies and additions to the other reviewers above). We do agree though that in our conclusions we might also relate to the current situation of the COVID-19 pandemic and therefore we added the following information:

Discussion, lines 466 to 472:

*“**At the time of writing this article, the COVID-19 pandemic in most regions worldwide has subsided and data collection of COVID-19 cases and deaths has been discontinued. In some areas however, COVID-19 infection rates are still elevated, may increase again or new variants may keep emerging. The implications mentioned in our review, in terms of research and clinical- and policy interventions, such as the implementation of scalable and widely accessible (remote) psychological interventions, are thus still timely and may offer more sound and definite policy directives to mitigate the impact of global public health disasters on mental health.”*
