# Supplementary material for: COVID-19 and common mental health symptoms in the early phase of the pandemic: An umbrella review of the evidence
Source: PLoS Med. 2023 Apr 25;20(4):e1004206. doi: 10.1371/journal.pmed.1004206 (PMC10129001; doi:10.1371/journal.pmed.1004206)
Supplement: Attachment — Submitted filename: Author summary_PLOS medicine_CD.docx [file pmed.1004206.s005.docx]

**Research in context**

**Why was this study done**

- The COVID-19 pandemic has been one of the greatest global public health challenges of the last century and has impacted multiple aspects of health and public life.
- An adverse association between the pandemic and global mental health was expected and many research projects have been rapidly developed to assess this.
- There is uncertainty about the degree and extent of the associations between the pandemic and its associated measures and mental health.

**What did the researchers do and find?**

- We synthesized evidence from 123 systematic reviews of individual studies on symptoms of the common mental disorders, including depression, anxiety and PTSD symptoms, in general- and specific populations and in healthcare workers.
- Only seven of the reviews identified compared differences in mental health outcomes during the COVID-19 pandemic or lockdowns to periods before the pandemic or periods with minimal restrictions. Another 116 reviews provided combined data on during pandemic prevalence rates of elevated levels of mental health outcomes.
- Mental health and particularly mood of the general population slightly deteriorated in the first half year of the pandemic and symptom increases were associated with periods of public health measures and social restrictions. Also, people with pre-existing physical (but not mental) health conditions, females, and young people were more significantly impacted in the first year of the pandemic.
- The available reviews showed large and often unexplained variation in mental health impact of the pandemic and in prevalence rates between individual studies. In some reviews methodological-, individual-level and COVID-19 exposure factors such as assessment tool used, age, gender and COVID-19 infection rate explained part of the variation while in others this was not the case.
- Most systematic reviews meta-analyses were of relatively low methodological quality.

**What do these findings mean?**

- This umbrella review could help clinicians, researchers and policy makers to better understand the current evidence on the association between the COVID-19 pandemic and mental health, particularly in specific vulnerable subpopulations.
- The interpretability of the included systematic reviews was limited by the great variation in prevalence rates and associations between studies and because of the scarcity of longitudinal data.
- Policy makers and researchers should address common pitfalls of research designs prior to (digital) implementation of systematic mental health assessments in future population panels.
