# Supplementary material for: COVID-19 and common mental health symptoms in the early phase of the pandemic: An umbrella review of the evidence
Source: PLoS Med. 2023 Apr 25;20(4):e1004206. doi: 10.1371/journal.pmed.1004206 (PMC10129001; doi:10.1371/journal.pmed.1004206)
Supplement: Attachment — Submitted filename: Response_reviewers_revision.docx [file pmed.1004206.s006.docx]

**Comments from Reviewers:**

**Reviewer #1: Thanks authors for their great effort to improve the manuscript. The authors have comprehensively addressed all my concerns. I am satisfied with the response and revision. No further issues needing attention.**

We thank the reviewer for re-reading the manuscript and for assessing the improvements we made to our paper.

**Reviewer #3: All my comments have been addressed. The article has been strengthened substantially with the update of the review and introduction of additional discussion content. There were some small typos (e.g. line 65 no 'the' at start, line 70 'hence' does not work here, lines 457-459 are repeated in 466-468) the authors will need to proof read the paper well prior to publication.**

We thank the reviewer for re-reading the manuscript and for assessing the improvements we made to our paper. Thank you for also flagging the small typos and the noticed repetition in the final Conclusion paragraph. We solved these issues accordingly and will properly proof read the paper before publication.

In current line 93*: Added ‘The main difficulty…’.*

In current line 99: *‘Hence, ‘ has been deleted.*

The lines 493-504 in the current revision have been deleted. Please see our response to a similar remark to the Conclusions paragraph from the editors.

**Reviewer #4: The authors should be congratulated on the hard work they have put into this revision. I am satisfied with the amendments made, and do not have any further specific recommendations for revision. However, I must say that the Abstract is a pretty dense and quite difficult read, and this may reduce the impact of the paper.**

Authors’ response: We thank the reviewer for re-reading the manuscript and for assessing the improvements we made to our paper. We have taken the remarks concerning our Abstract seriously and have made a few textual changes that hopefully improved the accessibility of the text for readers.
